# Taxonomic Structure and Wing Pattern Evolution in the *Parnassius mnemosyne* Species Complex (Lepidoptera, Papilionidae) †

**DOI:** 10.3390/insects14120942

**Published:** 2023-12-12

**Authors:** Vladimir A. Lukhtanov, Evgeny V. Zakharov

**Affiliations:** 1 Department of Karyosystematics, Zoological Institute, Russian Academy of Sciences, Universitetskaya Nab. 1, 199034 Saint-Petersburg, Russia; 2 Centre for Biodiversity Genomics, Department of Integrative Biology, College of Biological Sciences, University of Guelph, Guelph, ON N1G 2W1, Canada; zakharov@uoguelph.ca

**Keywords:** biodiversity, *COI*, DNA barcoding, insects, Lepidoptera, *Parnassius*, *Driopa*, taxonomy, mitochondrial DNA, sphragis, reinforcement

## Abstract

**Simple Summary:**

The butterfly genus *Parnassius* attracts the attention of numerous researchers. However, species and populations from Central and Western Asia remain understudied compared to taxa from Western Europe and East Asia. In our study, using the analysis of DNA barcodes and morphology (wing color, male genitalia, and sphragis shape in females), we substantiate the species status for *P. nubilosus* **stat. nov**. from Turkmenistan and NE Iran. We demonstrate that the *P. mnemosyne* group includes three morphologically similar species, *P. mnemosyne* (Western Eurasia), *P. turatii* (southwestern Europe), and *P. nubilosus*, as well as *P. ariadne* (Altai). The latter species differs from the rest of the group in the presence of red spots on the wings. We also suggest that morphological evolution within the genus involved processes of loss and reappearance of red wing spotting. The pattern of these processes is consistent with the reinforcement theory, which predicts a higher level of divergence between sympatric and allopatric populations and species.

**Abstract:**

In our study, using the analysis of DNA barcodes and morphology (wing color, male genitalia, and female sphragis shape), we show that the group of species close to *P. mnemosyne* comprises the western and eastern phylogenetic lineages. The eastern lineage includes *P. stubbendorfii*, *P. glacialis,* and *P. hoenei*. The western lineage includes three morphologically similar species: *P. mnemosyne* (Western Eurasia), *P. turatii* (southwestern Europe), and *P. nubilosus* **stat. nov**. (Turkmenistan and NE Iran), as well as the morphologically differentiated *P. ariadne* (Altai). The latter species differs from the rest of the group in the presence of red spots on the wings. *Parnassius mnemosyne* s.s. is represented by four differentiated mitochondrial clusters that show clear association with specific geographic regions. We propose to interpret them as subspecies: *P. mnemosyne mnemosyne* (Central and Eastern Europe, N Caucasus, N Turkey), *P. mnemosyne adolphi* (the Middle East), *P. mnemosyne falsa* (Tian Shan), and *P. mnemosyne gigantea* (Gissar-Alai in Central Asia). We demonstrate that in *P. ariadne,* the red spots on the wing evolved as a reversion to the ancestral wing pattern. This reversion is observed in Altai, where the distribution areas of the western lineage, represented by *P. ariadne*, and the eastern lineage, represented by *P. stubbendorfii*, overlap. These two species hybridize in Altai, and we hypothesize that the color change in *P. ariadne* is the result of reinforcement of prezygotic isolation in the contact zone. The lectotype of *Parnassius mnemosyne* var. *nubilosus* Christoph, 1873, is designated.

## 1. Introduction

Butterflies of the genus *Parnassius* Latreille, 1804, are characterized by a unique type of wing pattern consisting of a milky white background, a combination of black spots and translucent areas, and large, bright red eye-shaped spots [1,2]. Another morphological feature of the genus is that fertilized females carry a mating plug [2], known in the literature as sphragis (plural: sphragides) [3]. It is a firm structure originating from male accessory glands, fixed to the ventral side of the female’s abdomen following insemination, where it blocks the ostium bursae [3,4]. Within the genus *Parnassius*, sphragides are extremely diverse in size and shape [5]. Despite significant individual variation [6,7], the sphragides are species-specific and represent a reliable character for species identification [2,5].

The genus attracts the attention of taxonomists due to the complexity of its evolutionary history and classification [1,2,8,9,10,11,12,13,14,15,16,17]. It includes a large number of species protected by international, national, and regional laws [18,19,20,21]. It is a model system for studying issues of ecology [22,23,24], biogeography [11,12,25], and insect–plant relationships [26]. It is not surprising, therefore, that significant progress has been made in the study of the evolutionary history and taxonomy of the genus, particularly in the phylogeny reconstruction at the level of subgenera and major species groups [2,8,9,10,11,12,13,15,16,17,27]. There is much less clarity on issues relating to the finer taxonomic structure of the genus at the species level. Especially many unresolved questions remain in the taxonomy of species complexes distributed in the little-studied regions of Central Asia, for example, in species that are close to *P. delphius* Eversmann, 1843, and *P. staudingeri* Bang-Haas, 1882 [28].

Oddly enough, similar problems exist for the species living in the much more studied regions of Western Europe and the Asian Far East. An example of such a poorly studied complex is a group of taxa close to *P. mnemosyne* (Linnaeus, 1758). This group is included in the subgenus *Driopa* Korshunov, 1988, of the genus *Parnassius* [13,17]. A morphological feature of the *P. mnemosyne* species complex is the complete absence of red ocelli on the wings [29]. This complex comprises the western and eastern phylogenetic lineages. The western lineage, whose range occupies the western part of Eurasia from the Pyrenees to the Trans-Urals and Central Asia, is known to be represented by the species *P. mnemosyne* and *P. turatii* Fruhstorfer, 1908 [30,31]. The eastern lineage, whose range occupies the eastern part of Eurasia from Altai to the Japanese Islands, is represented by the species *P. stubbendorfii* Ménétriés, 1849; *P. glacialis* Butler, 1866; and *P. hoenei* Schweitzer, 1912 [15]. It has recently been shown that this complex should also include the local Altai species *P. ariadne* (Lederer, 1853) [13,15], which differs sharply from other species of the group by the presence of red ocelli on the hind wings, thus representing a condition characteristic of the genus *Parnassius* as a whole.

The Western European and Far Eastern populations of the *P. mnemosyne* group have been the subject of several phylogeographic studies based on the analysis of mitochondrial genes [8,13,32,33], mitochondrial and nuclear markers [24], mitochondrial genes and allozymes [34], and whole-genome resequencing [21]. However, populations of this group from Eastern Europe, Central Asia, and Altai remain almost completely unstudied.

In our study, we close this gap by presenting data on mitochondrial DNA barcodes, wing patterns, and sphragis shape for 189 specimens from the western lineage of the *P. mnemosyne* species complex from eastern Europe, the Caucasus, Transcaucasia, the Levant, Iran, and Central Asia, and 11 specimens from *P. ariadne* from Kazakhstan and Russia.

As a result of the research, we

(1) Demonstrate that the western lineage of the *P. mnemosyne* species complex is a group consisting of three morphologically similar species: *P. mnemosyne* s.s. (Western Eurasia), *P. turatii* (southwestern Europe), and *P. nubilosus* Christoph, 1873, **stat. nov.** (Turkmenistan, NE Iran), and the morphologically highly differentiated species *P. ariadne* (Altai).

(2) Designate the lectotype of the taxon *P. mnemosyne* var. *nubilosus* Christoph, 1873.

(3) Show that *P. mnemosyne* s.s. is represented by four differentiated mitochondrial clusters that demonstrate clear connections to certain geographical regions. We propose to interpret them as subspecies: *P. mnemosyne mnemosyne* (Central and Eastern Europe, Northern Caucasus, and Northern Turkey), *P. mnemosyne adolphi* Bryk, 1911 (the Middle East, Transcaucasus), *P. mnemosyne falsa* Pagenstecher, 1911 (Tian Shan), and *P mnemosyne gigantea* Staudinger, 1886 (Gissaro-Alai in Central Asia). Alternatively, these four clusters may be interpreted as four subspecies groups or as four closely related allopatric species.

(4) Show that the red ocelli on the wings of *P. ariadne* arose secondarily as a reversion to the ancient wing color pattern characteristic of the genus *Parnassius* as a whole and hypothesize that the red spots arose in *P. ariadne* as a result of reinforcement of prezygotic reproductive isolation in the zone of secondary contact between the western and eastern lineages of the *P. mnemosyne* species complex.

## 2. Materials and Methods

### 2.1. Samples

Standard mitochondrial DNA barcodes (658 bp fragments of the *cytochrome c oxidase subunit I* gene) were obtained for 189 dried samples of the western lineage of the *P. mnemosyne* species complex of *P. mnemosyne*, eleven samples of *P. ariadne*, and three samples of *P. nordmanni* (Appendix A). The specimens of *P. mnemosyne* were collected in the European part of Russia, Turkey, Iran, Israel, Georgia, Armenia, Azerbaijan, Kazakhstan, Kyrgyzstan, Uzbekistan, and Tajikistan (Appendix A). The collecting sites cover nearly all the known distribution regions of *P. mnemosyne* in the eastern half of its distribution range. None of the specimens were subjected to any chemical treatment before desiccation. The climate of the regions ensured quick drying of specimens, which were stored at room temperature (18–25 °C) for 5–20 years. The specimens examined are deposited in the Zoological Institute of the Russian Academy of Sciences and the research collection of B. Khramov (St. Petersburg, Russia). Full details of the voucher samples are presented in Table S1 in Supplementary Material S1.

### 2.2. COI Amplification and Sequencing

DNA was extracted with standard protocols [35] from single legs removed from dried voucher specimens prior to their rehydration for spreading. All the voucher specimens are now identified with labels that include the butterfly field numbers (Table S1 in Supplementary Material S1). Additionally, unique Process IDs automatically generated by the BOLD system have been added to each specimen record. For the majority of samples (175 specimens), the primers LepF1 (5_-ATTCAACCAATCATAAAGATATTGG-3_) and LepR1 (5_-TAAACTTCTGGATGTCCAAAAAATCA-3_) amplified the target 658-bp fragment of *COI*. For the rest of the samples, most of which were 20 or more years old, we amplified shorter overlapping fragments by using the primer combination MLepF1 (5_-GCTTTCCCACGAATAAATAATA- 3_)-LepR1 (407-bp amplicon) and MLepR1 (5_-CCTGTTCCAGCTCCATTTTC-3_)-LepF1 (311-bp amplicon). Sequences were obtained by using ABI 3730XL sequencers (Applied Biosystems).

### 2.3. Sequence Analysis

Sequences were edited to remove ambiguous base calls and primer sequences and assembled by using SEQUENCHER (Gene Codes, Ann Arbor, MI, USA) (https://www.genecodes.com/sequencher-features, accessed on 18 October 2023). Sequences were then aligned using CLUSTAL W (Conway Institute, University College Dublin, Ireland) [36] software and manually edited. Sequence information was entered in the Barcode of Life Database (BOLD, https://www.boldsystems.org/, accessed on 18 October 2023) along with an image and collateral information for each voucher specimen. The detailed specimen records and sequence information, including trace files, are available on BOLD in the project LOWAM or in the dataset DS-MNEMOSYN. All sequences have been submitted to GenBank (Appendix A).

A comparison of the obtained *COI* barcodes revealed 38 unique haplotypes within the five studied species (Appendix A). The data matrix for subsequent phylogenetic analysis also included the mitochondrial haplotypes known for *P. mnemosyne* and *P. turatii* from Western Europe and Asia Minor, as well as the known haplotypes of other species of the subgenus *Driopa* [13,32,37,38,39,40,41,42]. *Parnassius orleans* Oberthür, 1890, is known to be the sister group to all other species of the subgenus *Driopa* [13,15,17], so it was chosen as the outgroup for rooting the tree. The final alignment of the analyzed samples (file in FASTA format) is presented in Supplementary Material S2.

The Bayesian analysis of the matrix was performed using the program MrBayes3.2 [43] as previously described [44,45]. A GTR substitution model with gamma-distributed rate variation across sites and a proportion of invariable sites was specified before running the program for 10,000,000 generations with default settings. The first 2500 trees (out of 10000) were discarded prior to computing a consensus phylogeny and posterior probabilities. The consensus of the obtained trees was visualized using FigTree 1.4.4 (http://tree.bio.ed.ac.uk/software/), accessed on 18 October 2023).

To create the haplotype network, sequences with missing nucleotides were removed from the alignment. The ends of the sequences were truncated so that all sequences were the same length, resulting in a 632-bp alignment. The maximum parsimony haplotype network was constructed using TCS v. 1.21 [46]) and visualized using the program tcsBU tool [47]. The minimum *COI* p-distances (%) between the taxa of the subgenus *Driopa* were calculated using the MEGA 11 program [48].

### 2.4. Morphology Analysis

All samples that were used for barcode analysis were also used for morphological studies. Additionally, the samples from the collection of the Zoological Institute (St. Petersburg, Russia) were inspected. This collection includes several thousand individuals of the subgenus *Driopa*, including two syntypes of *P. nubilosus* and the samples of *P. nubilosus* collected relatively recently by V. Dubatolov in Kopetdagh (Turkmenistan). Photographs of butterflies and sphragides were taken with a Nikon D810 digital camera (Nikon Corporation, Minato City, Tokyo, Japan)) equipped with a Nikon AF-S Micro Nikkor 105 mm lens, using the built-in flash as a lighting source.

For genitalia preparation, adult abdomens were soaked in hot (90 °C) 10% KOH for 3–10 min. Then, they were transferred to water, and the genitalia were carefully extracted and macerated under a stereomicroscope with the help of a pair of preparation needles or with the help of a needle and a watchmaker’s tweezer. Once cleansed of all unwanted elements, they were transferred and stored in glycerin. Cleansed genital armatures were handled, studied, and photographed while immersed in glycerin, free from pressure due to mounting and, therefore, free from the ensuing distortion. Photographs of genitalia were taken with a Leica M205C binocular microscope (Leica Microsystems, Wetzlar, Germany) equipped with a Leica DFC495 digital camera and processed using the Leica Application Suite v.4.5.0 software.

To reconstruct the probabilities of ancestral states, a Bayesian approach was used, as implemented in the program MrBayes3.2 [43]. The states studied were coded as 0 (absence of red spots) and 1 (presence of red spots). These states were implemented into a matrix of molecular features. The probability of ancestral states for each node was calculated separately. The command block used to analyze ancestral states is given in Supplementary Material S3.

## 3. Results

### 3.1. Phylogenetic Analysis

**Figure 1:**
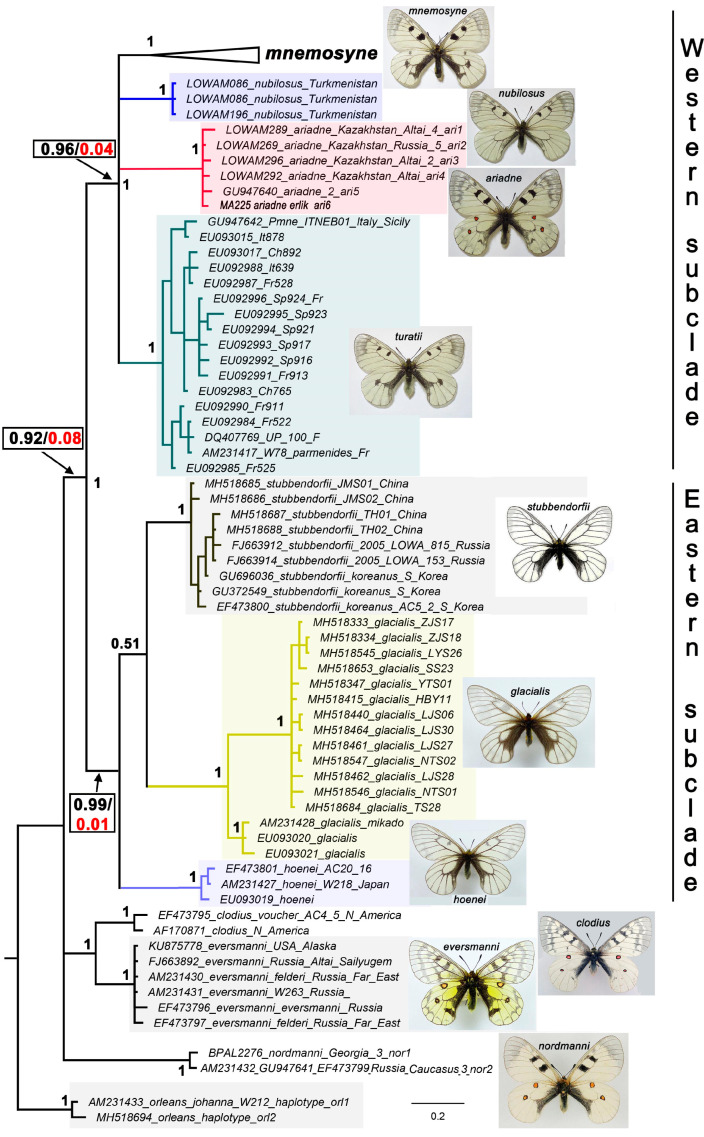
The Bayesian tree of the *Parnassius* (*Driopa*) species based on analysis of the mitochondrial *COI* barcodes. Numbers at nodes indicate Bayesian posterior probabilities (BPP) (higher than 0.5). Black/red values in the rectangles show the probabilities of the ancestral states “absence of red spots”/“presence of red spots”. *Parnassius ariadne erlik* is shown in bold. The number before the name of the haplogroup means the number of identified specimens with this haplogroup. The species clusters are highlighted in different colors.

Within the subgenus *Parnassius* (*Driopa*) (excluding *P. orleans*), Bayesian analysis identified three highly supported (in all cases, posterior probability = 1) major clades (Figure 1). The first clade is represented by the species *P. nordmanni* Ménétriés, 1850. The second clade is represented by the species *P. eversmanni* Ménétriés, 1850 *+ P. clodius* Ménétriés, 1855. The third clade is represented by species of the *P. mnemosyne* group, that is, species lacking red spots on the wings. The third clade also included *P. ariadne*, a species with red spots. The third clade is divided into two highly supported subclades, which can be designated as the western and eastern subclades (Figure 1).

The red-spotted species *P. ariadne* was found to be deeply nested within the western subclade. Within the western subclade, four main lineages were identified. These are the lineages of (1) *P. mnemosyne*, (2) *P. nubilosus* **stat. nov**., (3) *P. ariadne*, and (4) *P. turatii*. The phylogenetic relationships between these lineages were not solved, most likely because the “barcode” sequences of mtDNA were insufficient to uncover the finer phylogenetic relations.

Within the eastern subclade, three main lineages were identified, which correspond to the species *P. stubbendorfii*, *P. glacialis,* and *P. hoenei*. Within the species *P. glacialis*, two sublineages were found, which correspond to mainland (China) and island (Japan) populations.

**Figure 2:**
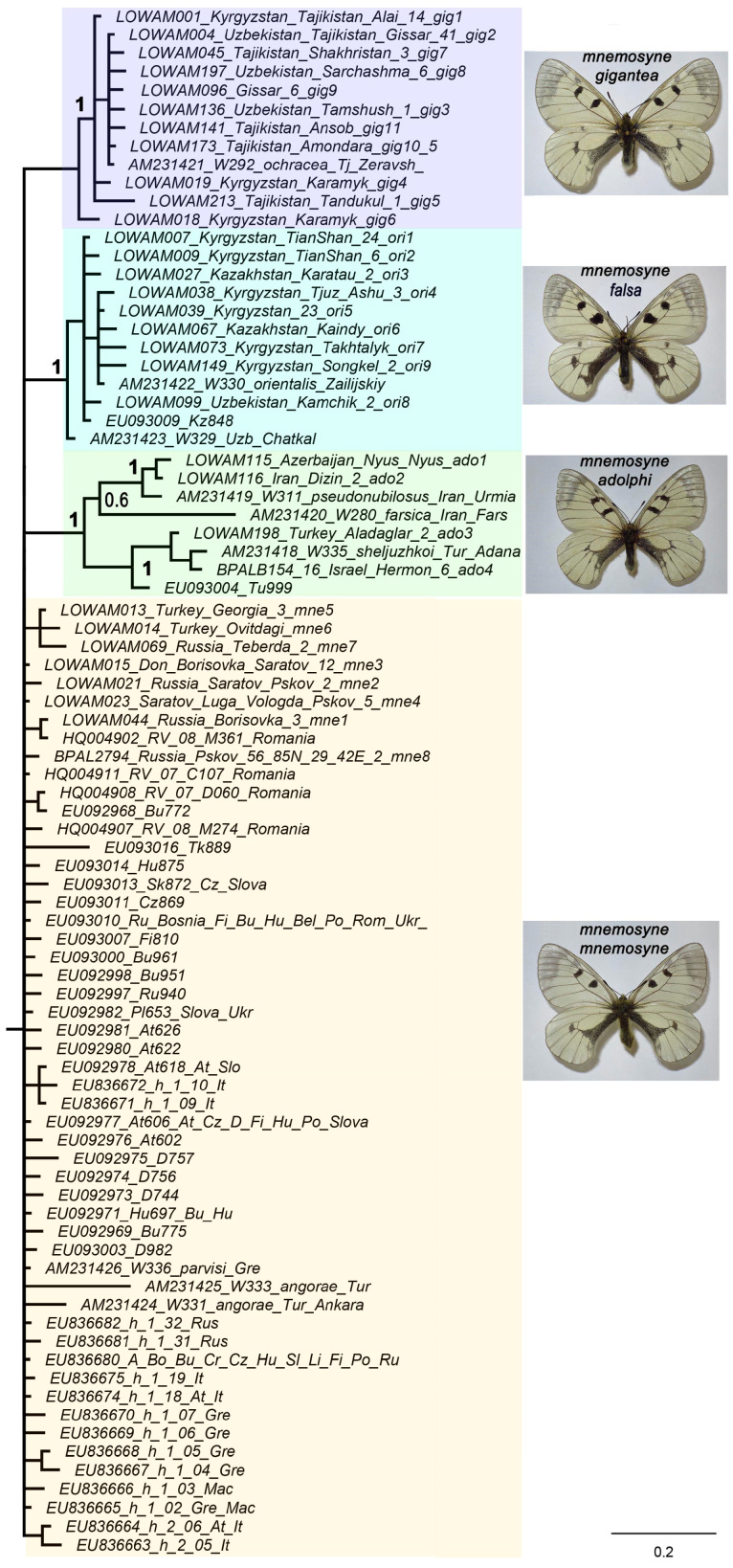
A part of the Bayesian tree of the *Parnassius* (*Driopa*) species (Figure 1) showing the structure of *P. mnemosyne* sensu stricto. The tree is based on an analysis of the mitochondrial *COI* barcodes. Numbers at nodes indicate Bayesian posterior probabilities (BPP) (higher than 0.5). The number before the name of the haplogroup means the number of identified specimens with this haplogroup. The subspecies clusters are highlighted in different colors.

**Figure 3:**
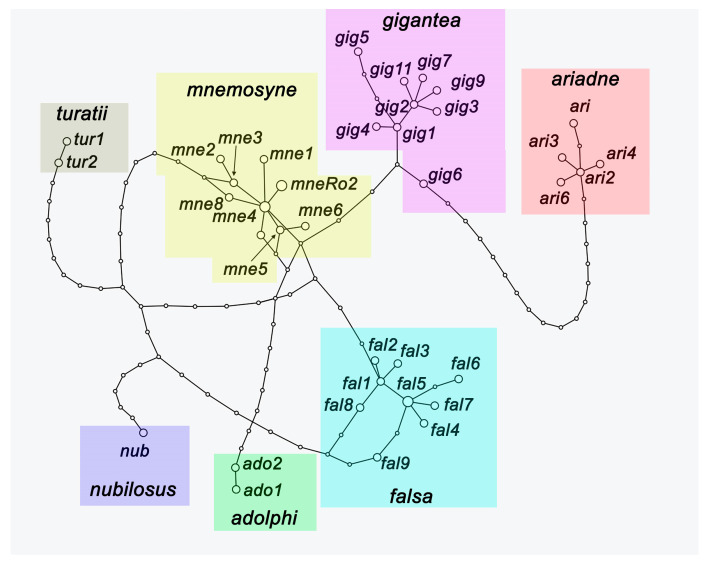
The TCS haplotype network of the samples of the western lineage of the *P. mnemosyne* species complex. The species and subspecies clusters are highlighted in different colors. Geographic data for the studied haplotypes are presented in Appendix A.

Within the species *P. mnemosyne* (Figure 2), three highly supported sublineages were identified, which are designated as *P. mnemosyne adolphi* (the Middle East), *P. mnemosyne falsa* (Tian Shan), and *P. mnemosyne gigantea* (Gissar-Alai in Central Asia). The haplotypes of *P. mnemosyne mnemosyne* from East Europe, NW Turkey, and Caucasus were found to form a basal polytomy on the tree. In the TCS haplotype network, members of this polytomy were found as a separate compact group of similar haplotypes (Figure 3).

**Figure 4:**
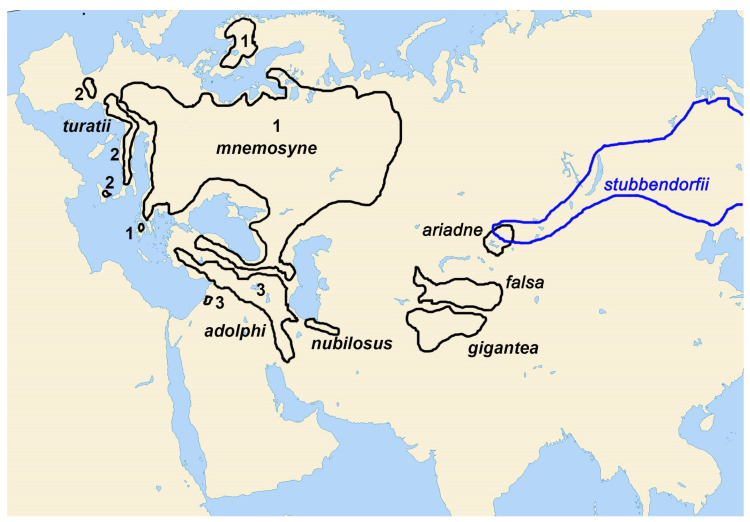
Schematic representation of the ranges of *P. mnemosyne*, *P. turatii*, *P. nubilosus* **stat. nov**., and *P. ariadne*. The western part of the range of *P. stubbendorfii* is also shown in blue to demonstrate overlap with the distribution area of *P. ariadne*. Number 1 indicates geographic isolates of *P. mnemosyne mnemosyne*. Number 2 indicates geographic isolates of *P. turatii*. Number 3 indicates geographic isolates of *P. mnemosyne adolphi*.

All the detected lineages, both major (Figure 1) and secondary lineages (Figure 2), were found to show a clear connection with certain geographical regions (Figure 4): *P. turatii* with southwestern Europe, *P. mnemosyne* with Western Eurasia, *P. nubilosus* **stat. nov**. with Turkmenistan and NE Iran, *P. ariadne* with Altai and Saur-Tarbagatai Mts, *P. mnemosyne mnemosyne* with Central and Eastern Europe, N Caucasus and N Turkey, *P. mnemosyne adolphi* with the Middle East, *P. mnemosyne falsa* with Tian Shan, and *P. mnemosyne gigantea* with Gissar-Alai region in Central Asia.

The minimum *COI* p-distances between the species of the subgenus *Driopa* are shown in Table 1.

### 3.2. Morphology

**Figure 5:**
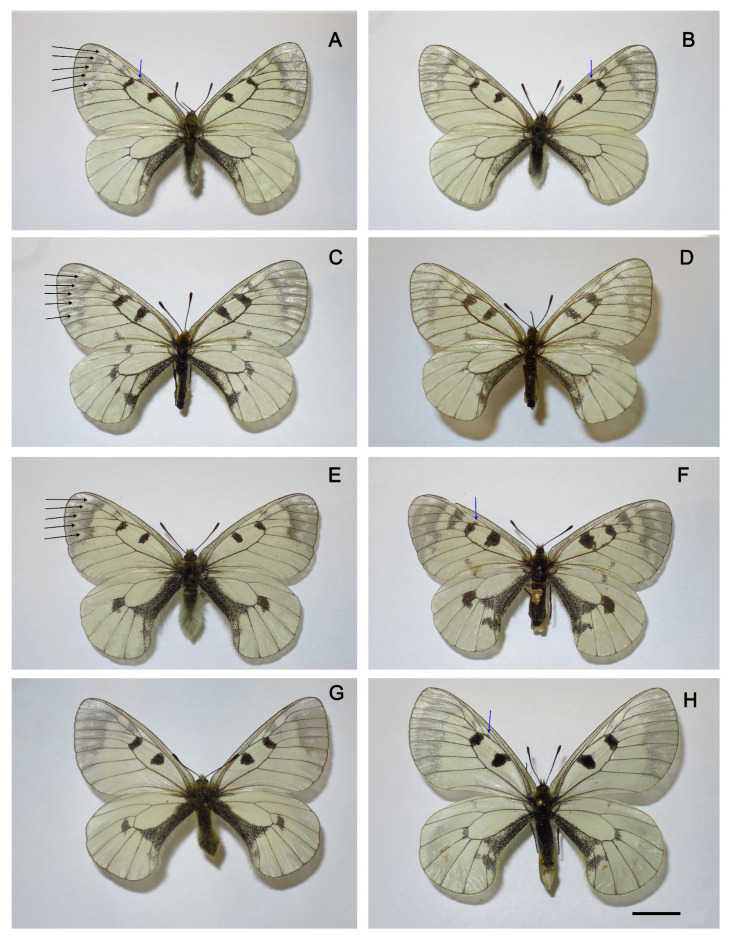
*Parnassius nubilosus* (**A**–**D**), *P. turatii* (**E**,**F**), and *P. mnemosyne mnemosyne* (**G**,**H**). The white band in the apical transparent part of the forewings is shown by black arrows. The additional small black streak on the discal cell of the forewing is shown by the blue arrow. All specimens are from the collection of the. Zoological Institute, St. Petersburg. Scale = 1 cm. (**A**,**B**) *P. nubilosus*, males, Turkmenistan, Kopetdagh, Dushak Mt, 6 June 1986, V.Dubatolov leg. (**C**,**D**) *P. nubilosus*, females, Turkmenistan, Kopetdagh, Dushak Mt, 6 June 1986, V.Dubatolov leg. (**E**) *P. turatii*, male, Italy, Sicilia, Madonie, 1200’, 15 July 1910, coll. Krüger, mus. Turati E, coll. Avinov. (**F**) *P. turatii*, female, Italy, Sicilia, Madonie, 1200’, 15 July 1910, coll. Krüger, mus. Turati E, coll. Avinov. (**G**) *P. mnemosyne mnemosyne*, male, sample J178, Russia, Kaluga region, 27 May 1979, leg. I. Sokolov. (**H**) *P. mnemosyne mnemosyne*, female, Russia, Penza region, Serdobsk, 20 May 2008, leg. Polumordvinov.

**Figure 6:**
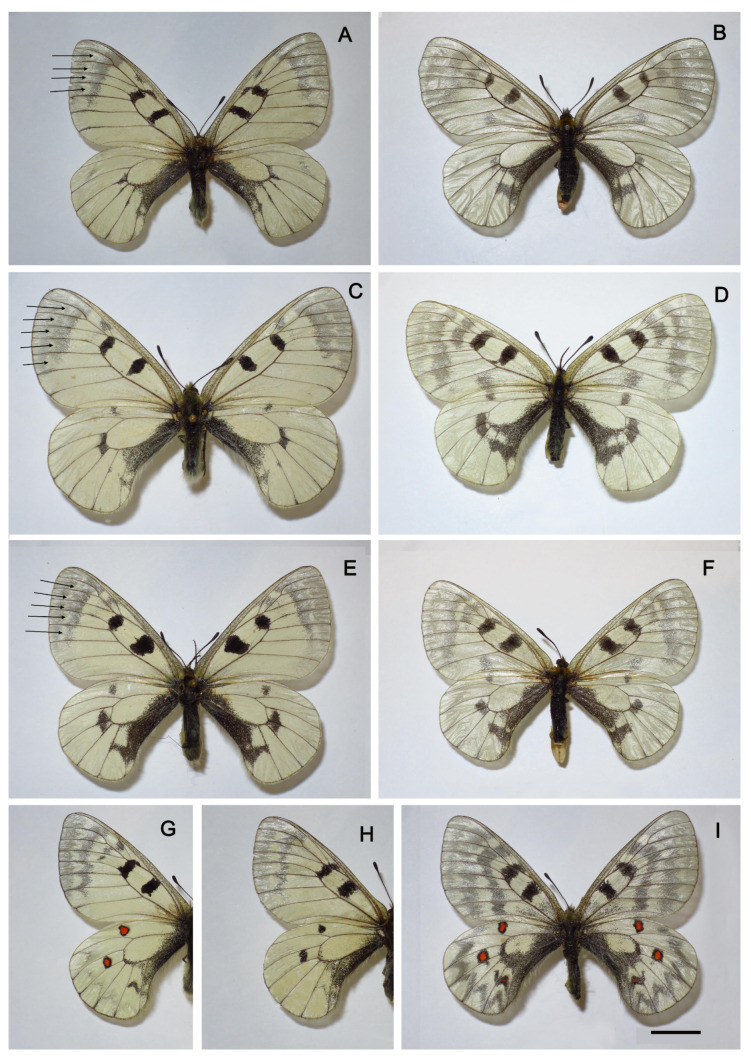
*Parnassius mnemosyne adolphi* (**A**,**B**), *P. mnemosyne gigantea* (**C**,**D**), *P. mnemosyne falsa* (**E**,**F**), and *P. ariadne* (**G**–**I**). The white band in the apical transparent part of the forewings is shown by arrows. All specimens are from the collection of the Zoological Institute, St. Petersburg. Scale = 1 cm. (**A**) *P. mnemosyne adolphi*, male, sample J115, Azerbaijan, Nakhichevan, Njus-Njus vill., Sary-Dara Mt, 2300 m, 7 July 1984, V.A.Lukhtanov leg. (**B**) *P. mnemosyne adolphi*, female, Transcaucasus, distr. Zangezur, loc. Litshk (prope Megri), 6 June 1910, E.Miller leg. (**C**) *P. mnemosyne gigantea*, male, Uzbekistan, Gissar Mts, 50 km east of Shakhrisyabz, 1700–2300 m, 19 May 1994, N.Kandul leg. (**D**) *P. mnemosyne gigantea*, female, (Kyrgyzstan), Alai Mts, Kok-Su, Kosh-Tjube, 3300 m, 28 July 1964, Bundel leg. (**E**) *P. mnemosyne falsa*, male, sample J112, Kyrgyzstan, Naryn-Too Range, 29–30 July 1995, V.Shchurov leg. (**F**) *P. mnemosyne falsa*, female, Kyrgyzstan, Kyrgyz Ala-Too Range, Uzyngyr, 2500 m, 13 July 1974, V.V. Dubatolov leg. (**G**) *P. ariadne*, male, Kazakhstan, Saur Mts, Zhanaturmys, 1100 m, 4 June 1987, V.A.Lukhtanov leg. (**H**) *P. ariadne*, male, Kazakhstan, S Altai, Kurtchum Mts, Kalinino, 22 June 1985, V.A.Lukhtanov leg. (**I**) *P. ariadne*, female, Kazakhstan, S Altai, Markakol Lake, Urunkhaika, 1700 m, 24 June 1983, V.A.Lukhtanov leg.

Wing pattern. The wing pattern of butterflies in the *P. mnemosyne* group is very variable (Figure 5 and Figure 6). However, it is possible to identify elements that are fixed or almost fixed for individual populations. First, it is necessary to note the complete absence of red spots in all species except *P. ariadne*. In *P. ariadne*, red spots are present, with the exception of rare aberrant specimens (Figure 6H). A feature of butterflies from Tian Shan (subspecies *falsa*), Gissaro-Alai (subspecies *gigantea*), and the Middle East (the taxa *nubilosus* and *adolphi*) is a white band in the apical transparent part of the forewings.

The males of the taxon *nubilosus* are characterized by the complete absence of black spots on the hind wings, reduced black spots on the forewings, and an additional small black streak on the discal cell of the forewing (shown by a blue arrow in Figure 5A,B). The last three characters, although they create a characteristic appearance for *P. nubilosus*, are not absolutely specific to this taxon. Sporadically, the males without black spots on the hind wings, with a reduced pattern on the forewings and with an additional small black streak on the discal cell of the forewing, are found in populations classified as *P. mnemosyne gigantea*, *P. mnemosyne adolphi*, *P. mnemosyne mnemosyne,* and *P. turatii* (but not *P. mnemosyne falsa*).

**Figure 7:**
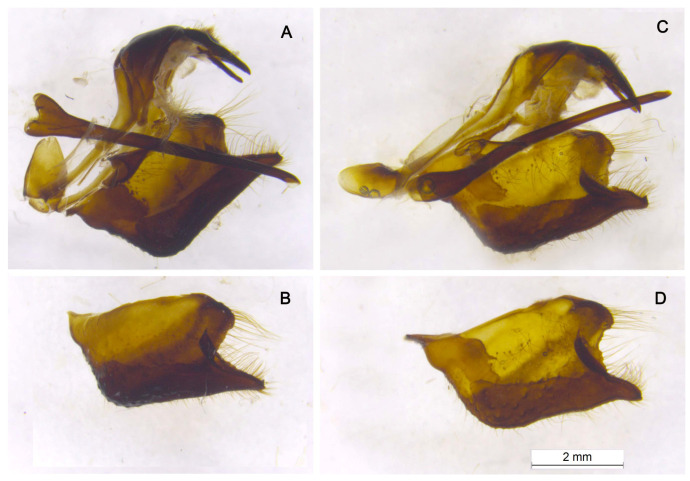
Male genitalia of *P. nubilosus* (**A**,**B**, sample J086) and *P. mnemosyne adolphi* (**A**,**C**, sample J115). (**A**,**C**), lateral view; left valve removed. (**B**,**D**), left valva. (**A**,**B**), Turkmenistan, Kopetdagh, Ai-Dere, 1 April 1979, Ju.Ya.Sokolova leg. (**C**,**D**), Azerbaijan, Nakhichevan, Njus-Njus vill., Sary-Dara Mt, 2300 m, 7 July 1984, V.A.Lukhtanov leg.

Male genitalia. In *P. nubilosus* (Figure 7A,B) as well as in *P. mnemosyne* (Figure 7C,D), uncus paired; branches of the gnathos are straight, rod-shaped; saccus is conical with a rounded apex; juxta is strongly sclerotized, V-shaped; valvae are massive; the caudal (lower) process of the valva is separated from the lobe-shaped costal (upper) process by a semicircular notch; aedeagus is thin, long, almost cylindrical. The specific features of the taxon *nubilosus* are compact, small-sized valvae with square outlines from the lateral view (Figure 7B) and a massive saccus (Figure 7A). In general, the male genitalia of *P. nubilosus* and *P. mnemosyne* are similar to the genitalia of other *Driopa* species studied by P. Gorbunov [49].

**Figure 8:**
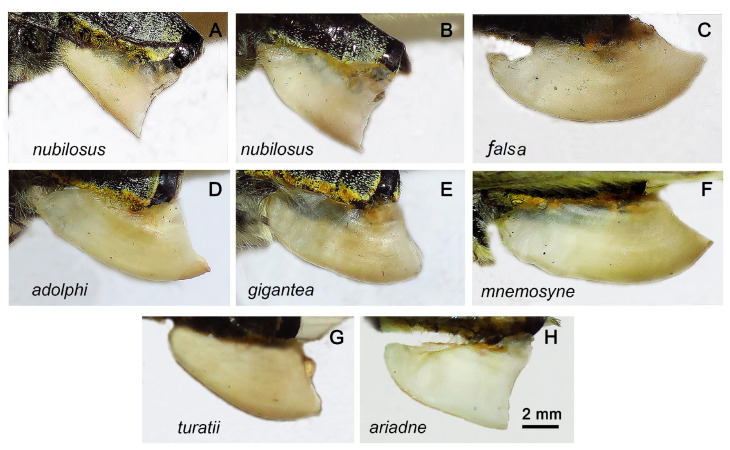
Sphragis of females of *P. nubilosus* (**A**,**B**), *P. mnemosyne falsa* (**C**), *P. mnemosyne adolphi* (**D**), *P. mnemosyne gigantea* (**E**), *P. mnemosyne mnemosyne* (**F**), *P. turatii* (**G**), and *P. ariadne* (**H**). All specimens are from the collection of the Zoological Institute, St. Petersburg. (**A**,**B**) Turkmenistan, Kopetdagh, Dushak Mt, 6 June 1986, V.Dubatolov leg. (**C**) Kyrgyzstan, Kyrgyz Ala-Too Range, Uzyngyr, 2500 m, 13 July 1974, V.V. Dubatolov leg. (**D**) Transcaucasus, distr. Zangezur, loc. Litshk (prope Megri), 6 June 1910, E.Miller leg. (**E**) (Kyrgyzstan), Alai Mts, Kok-Su, Kosh-Tjube, 3300 m, 28 July 1964, Bundel leg. (**F**) Russia, Penza region, Serdobsk, 20 May 2008, leg. Polumordvinov. (**G**) Italy, Sicilia, Madonie, 1200’, 15 July 1910, coll. Krüger, mus. Turati E, coll. Avinov. (**H**) Kazakhstan, S Altai, Kurtchum Mts, Kalinino, 7 June 1986, V.A.Lukhtanov leg.

Sphragis. (Figure 8). Sphragis appears in females after copulation. It is located at the end of the abdomen, on the ventral side. In *P. nubilosus* (Figure 8A,B), the sphragis is smaller than in *P. mnemosyne* and has a triangular (or almost triangular) outline when viewed from the side (Figure 8).

### 3.3. Ancestral State Reconstruction

Reconstruction of ancestral states using the MrBayes 3.2 program showed that, with a probability of 92%, the common ancestor of the *P. mnemosyne* clade did not have red ocelli on the wings (Figure 1). The common ancestor of the eastern subclade did not have red ocelli with a 99% probability. The common ancestor of the western subclade, which includes species closely related to *P. mnemosyne* and *P. ariadne*, did not have red ocelli with a 96% probability. Thus, with a 96% probability, the red ocelli of *P. ariadne* arose secondarily, probably as a reversion to the condition observed in species of the subgenus occupying a more basal position on the phylogenetic tree.

### 3.4. Nomenclature and Lectotype Designation of P. nubilosus

The type series of *Parnassius mnemosyne* var. *nubilosus* was collected by famous Russian (of German origin) entomologist Hugo Christoph in 1870 and 1871 from the area of Hadschyabad and Tasch in northeastern Iran [50], a region for which the name Hyrcania was used in the zoological literature of the time [51]. Judging by the original description, the type series included several specimens. This is evidenced by the fact that the taxon was not uncommon in the type-locality (“nicht selten”), the plural is used several times in the description of butterflies, and descriptions of both sexes are given in [50].

It is known that one part of Christoph’s collections from Iran (Persia) first ended up in the collection of Grand Duke Nikolai Mikhailovich Romanov and then in the Zoological Institute in St. Petersburg, and the other part of these collections came through H.J. Elwes’s collection to the Natural History Museum in London [52]. One syntype (male) of *P. mnemosyne* var. *nubilosus* was discovered by Verity [53] in London, where it remains today [54]. This syntype is depicted in the studies of Verity [53] and Tshikolovets and coauthors [55]. This male was mistakenly called the holotype in the study of Tshikolovets with coauthors [55]. According to the Codex of Zoological Nomenclature (Article 74.5) [56], this action does not constitute a valid lectotype designation.

Two other syntypes of *P. mnemosyne* var. *nubilosus*, originating from the collection of the Grand Duke Nikolai Mikhailovich Romanov, were found by us in the collection of the Zoological Institute.

The name *nubilosus* was repeatedly used in the taxonomic literature not only for butterflies from NE Iran and S Turkmenistan but also for butterflies from other regions, including Europe [51,53,57], which is obviously erroneous based on the data we received. Therefore, to maintain the stability of zoological nomenclature, we designate the lectotype of *Parnassius mnemosyne* var. *nubilosus.*

**Figure 9:**
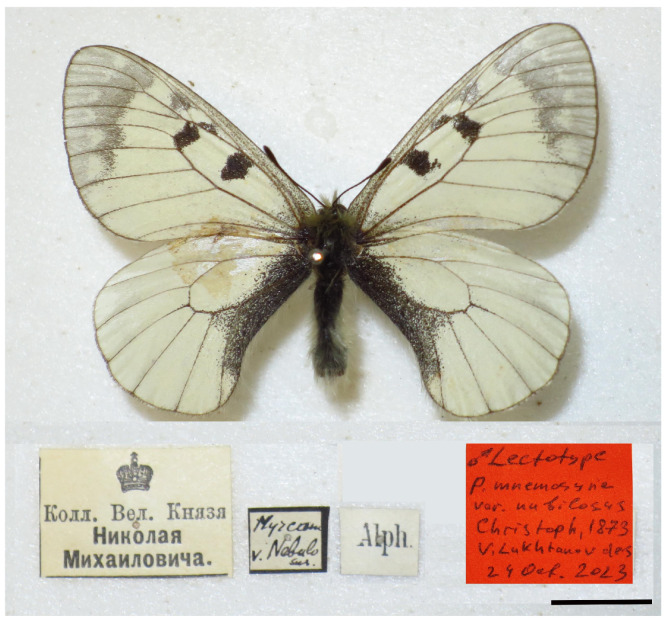
Lectotype of *Parnassius mnemosyne* var. *nubilosus* Christoph, 1873 (Zoological Institute, Russian Academy of Sciences, St. Petersburg). Scale = 1 cm. See the text for explanation of the labels.

As a lectotype, we select the male specimen shown in Figure 9 and bearing the following labels: “Koлл. Beл. Kнязя | Hикoлaя | Mиxaилoвичa.” (in Russian Translation: “coll. Grand Duke Nikolai Mikhailovich)” (printed); “Hyrcania | v. *Nebulosus*” [sic]” (in upper side, handwritten); “Alph” (eraky) (in underside, printed); “♂ Lectotype | *P. mnemosyne* | var. *nubilosus* | Christoph, 1873 | Lukhtanov des. | 24 Oct 2023” (handwritten). The lectotype is preserved in the collection of the Zoological Institute, Russian Academy of Sciences, St. Petersburg.

The paralectotype (female) in the collection of the Zoological Institute, Russian Academy of Sciences, St. Petersburg has the following labels: “Hyrcania | v. *Nebulosus*” [sic]” (in upper side, handwritten), “Alph” (eraky) (in underside, printed); “Koлл. Beл. Kнязя | Hикoлaя | Mиxaилoвичa.” (in Russian Translation: “coll. Grand Duke Nikolai Mikhailovich)” (printed); “Paralectotype ♀| *P. mnemosyne* | var. *nubilosus* | Christoph, 1873 | Lukhtanov des. | 24 Oct 2023” (handwritten). The male from the collection of the Natural History Museum in London, figured by Verity [53] and Tshikolovets et al. [55], also becomes the paralectotype.

Interestingly, the lectotype has the identification “*Nebulosus*” (the word is misspelled). Judging by the label, it was made by Sergei Alpheraky, who was the curator of the Grand Duke’s collection. The same misspelling of this name (“*Nebulosus*”) is also found in the monograph by Grum-Grshimailo [58]. Thus, this spelling is more likely to be an unjustified correction of the original spelling than a misprint.

### 3.5. Taxonomy and Nomenclature of P. ariadne

Eversmann (ref. [59], pp. 539–540, Table IX, Figure 1a–c) described and figured a new species named *Doritis clarius*, mentioning that it derived from “promontoriis Altaicis australibus” (mountain spurs of south Altai). However, the name *Doritis clarius* Eversmann, 1843, is invalid as it is a junior secondary homonym of *Papilio clarius* Hübner, 1805 (currently, both taxa belong to the genus *Parnassius*) and was replaced by the name *Doritis ariadne* Lederer (ref. [60], p. 354) by Hemming (ref. [61], p. 198). The name *Doritis ariadne* was first published by Lederer [60] in synonymy with *Doritis clarius* Eversmann, 1843, and is available according to Article 11.6.1 [56], as it was used before 1961 first by Hemming (ref. [61], p. 198).

**Figure 10:**
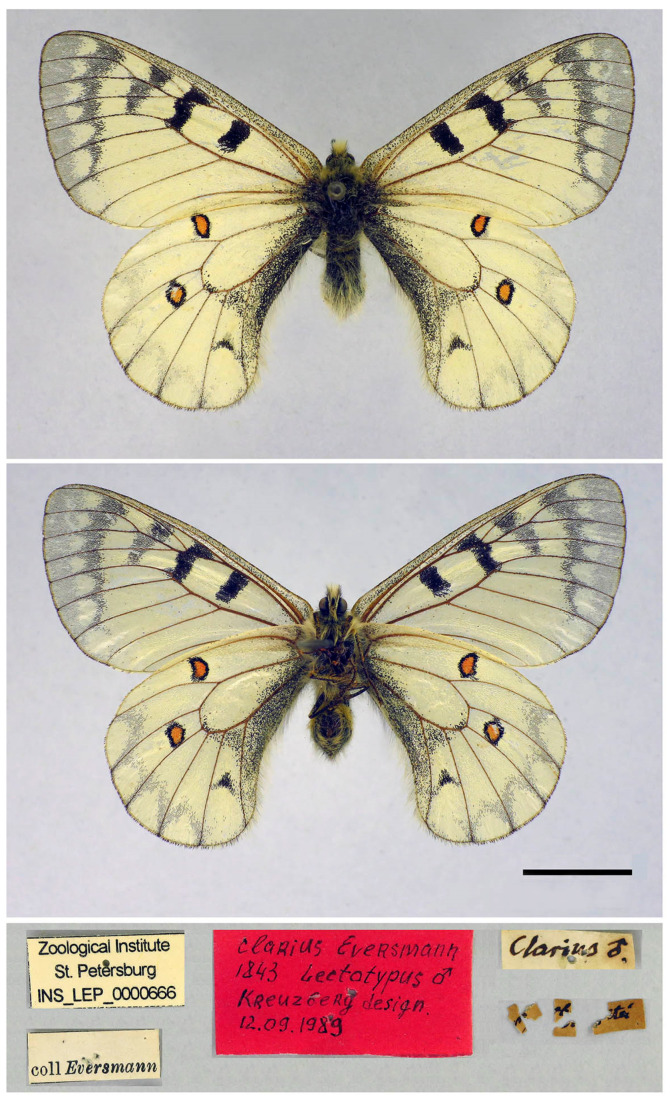
Lectotype of *Doritis clarius* Eversmann, 1843 (= *Parnassius ariadne* (Lederer, 1853)) (Zoological Institute, Russian Academy of Sciences, St. Petersburg, catalogue number: INS_LEP_0000666). Scale = 1 cm.

The lectotype of *Doritis clarius* Eversmann, 1843, was designated by Lukhtanov and coauthors [62]. It is preserved in the collection of the Zoological Institute, Russian Academy of Sciences, St. Petersburg. This is a male labelled “coll Eversmann” (printed), “*clarius* ♂” (handwritten), “*clarius* Eversmann | 1843 Lectotypus ♂ | Kreuzberg design. | 12.09.1989” (red paper, handwritten), “Tarbagatei” (broken label with a handwritten inscription), “Zoological Institute | St. Petersburg | INS_LEP_0000666” (printed) (Figure 10). The lectotype of *Doritis clarius* Eversmann, 1843, also becomes the lectotype of *Doritis ariadne* Lederer, 1853, according to Code Article 72.7 [56].

The designated lectotype is virtually identical with the male figured by Eversmann (ref. [59], Table IX, Figure 1a,b); most likely, it is this specimen that is depicted by Eversmann on the plate (Table IX, Figure 1a,b). According to the label, the lectotype originated from the “Tarbagatai” Mountains in eastern Kazakhstan. These mountains are located to the south of the Altai Mountains. In old literature, the Tarbagatai Mountains were often considered a part of the Altai (e.g., [63]), but currently, they are treated as a part of the Saur-Tarbagatai mountain system, which is located between Altai and Tian Shan.

*P. ariadne* is a local species known from the Kazakhstani, Russian, and Chinese parts of Altai and from the Saur-Tarbagatai mountain system in Kazakhstan and China. Populations of this species comprise three geographical groups: (1) the Saur-Tarbagatai group, (2) the group inhabiting the Altai mountains in the Irtysh river basin (separated from the first group by the Zaisan depression), and (3) the group inhabiting the Altai mountains in the basin of the Ob River (separated from the Irtysh group by the high main drainage divide of the Altai). We did not find any differentiation between butterflies from these three groups in the *COI* gene, which is in stark contrast to the situation found within *P. mnemosyne*.

As for morphology, there are also no significant differences between butterflies from these three population groups. An exception is the population of *P. ariadne*, described as *P. ariadne erlik* Yakovlev, 2009 [64] from the uppermost part of the Chuya River basin in the eastern part of the Russian Altai, where butterflies have small red eye-shaped spots, and individuals without red eye-shaped spots, as in Figure 6H, are relatively common. However, these characteristics are neither fixed nor unique. In addition, as a distinctive feature of the subspecies *P. ariadne erlik*, its association with the plant *Corydalis stricta* Steph. ex DC. (Papaveraceae: Fumarioideae) (the probable food plant of the caterpillars) was noted [64], while the remaining populations of the species are associated with *Corydalis nobilis* (L.) Pers. We obtained a DNA barcode from one individual of *P. ariadne erlik* with characteristically reduced red eye-shaped spots. The individual was collected by V. Lukhtanov 15 km north of Kosh-Agach on a mountain slope overgrown with *Corydalis stricta*, not far from the subspecies-type locality. Its DNA barcode differed from the barcodes of individuals from other populations by a single nucleotide substitution, which cannot be considered a serious distinction. We therefore consider this population, associated with *Corydalis stricta*, to be a highland form of *P. ariadne* rather than a separate subspecies.

For this reason, we consider all of the following names proposed as subspecific names as synonyms of *P. ariadne ariadne*:

*clarus* Bryk et Eisner, 1932 (TL: Saur-Gebirge);

*dentatus* Austaut, 1889(TL: “…les montagnes de Saisan…”;

*erlik* Yakovlev, 2009(TL: «Altai Rep., Chikhacheva Mts., Tabduair (Talduair) Mt., 2500 m»); 

*jiadengyuensis* Huang et Murayama, 1992(TL: Jiadengyu, Altai Mts, Xinjiang, China). 

### 3.6. Nomenclature of P. mnemosyne falsa

For Tien Shan populations of *P. mnemosyne*, the name *P. mnemosyne orientalis* Verity, 1911, is often used [29,65]. However, the name *orientalis* Verity, 1911, is infrasubspecific and an unavailable name since it was described as *Parnassius mnemosyne* var. *gigantea* race *orientalis* Verity, 1911 (ref. [53], p. 321), thus is quadrinomial and cannot be used for nomenclatural purposes. The oldest available name for this taxon is *Parnassius mnemosyne* var. *falsa* Pagenstecher, 1911 (ref. [66], p. 305). Pagenstecher attributed *falsa* to Bryk in his paper published in December 1911 [66], whereas Bryk’s name [67] was published on 1 June 1912, so Pagenstecher made it available with the type locality ‘Aulia, Ala, Zentralasien’. “Aulia, Ala” is a misspelling for Aulie-Ata (now Taraz) in Kazakhstan, a city located near the westernmost part of the Kyrgyz range in the Tien Shan.

## 4. Discussion

At the level of species and species groups, the topology of our mitochondrial tree (Figure 1) is fully compatible with the topology obtained for the subgenus *Driopa* using multigene mitochondrial [15] and nuclear phylogenomic data [17]. Thus, there are no conflicting signals in mitochondrial and nuclear DNAs and no reason to assume the influence of *Wolbachia* or introgressions [68,69] that would lead to mitochondrial discordance in the subgenus *Driopa*. At the same time, the relatively low resolution of phylogenetic analysis based on DNA barcodes should be noted. In our study, this manifests itself in the fact that the species of the Western subclade and the European populations of *P. mnemosyne* appear as polytomies on the phylogenetic tree. Clearly, the addition of nuclear genes is highly desirable in future studies to resolve these polytomies [70].

At the level of terminal lineages, the resulting topology is compatible with the pattern that would be expected if diversification was allopatric, that is, strictly confined to specific geographic regions. The *P*. *turatii* lineage is limited to the mountainous regions of southwestern Europe. This type of habitat is not unusual for many species [71]. The lineage of *P. mnemosyne mnemosyne* is limited to central and eastern Europe, including the adjacent territories of the Urals, Trans-Urals, North Caucasus, and Northern Turkey. The *P. mnemosyne adolphi* lineage is found in the highlands of Western Asia. The *P. mnemosyne falsa* lineage is limited to the Tian Shan. The lineage of *P. mnemosyne gigantea* is limited to the mountain systems of the Gissaro-Alai and western Pamirs. The *P. nubilosus* lineage is found in the mountain systems of northeastern Iran and Turkmenistan (Kopetdagh). The *P. ariadne* lineage is found in Altai and the Saur-Tarbagatai mountain system. There are numerous species and subspecies endemic to each of the above regions, and thus, these regions represent areas of independent speciation and subspeciation [71,72,73]. We recognize that nuclear, and especially genome-wide, data would be very important to support this conclusion. However, we assume the absence of mito-nuclear discordance and the pattern “one lineage–one geographic region” indicates that the mitochondrial phylogeny correctly reflects the processes of phylogenesis and geographic differentiation in the subgenus *Driopa*.

The level of mitochondrial differentiation between the studied lineages ranges from 0.95 to 2.42% for the subspecies of *P. mnemosyne* and from 2.29 to 7.8% for the species of the subgenus *Driopa*. Taking into account these data and the known rates of mitochondrial evolution in insects [74,75,76], we come to the conclusion that the age of these lineages is in the range of 0.5–5 million years. This range fits entirely within the Pliocene and Pleistocene periods. These age estimations for the subgenus *Driopa* correspond to the dates in the studies of previous authors [13,17]. In the study of Michel et al. [13], *P. mnemosyne falsa* (=*orientalis*) and *P. mnemosyne gigantea* are presented as poorly differentiated taxa, which is a consequence of an error in the subspecies identification. In the case of *P. mnemosyne gigantea*, this study presents a specimen from the Chatkal Range (Tian Shan), which actually belongs to *P. mnemosyne falsa*. Therefore, it can be assumed that the early divergence of the abovementioned taxa occurred in Pliocene and Pleistocene refugia in the Pyrenees and/or Apennines (*turatii*), Altai (*ariadne*), Kopetdagh (*nubilosus*), South Anatolia and Transcaucasus (*adolphi*), the mountains of southeastern Europe and Caucasus (*mnemosyne* s.s.), Tian Shan (*falsa*), and Gissaro-Alai (*gigantea*). Evolution in these refugia led to deep differentiation and, in four cases, to speciation.

Previously, an analysis of molecular markers revealed the taxonomic heterogeneity of populations attributed to *P. mnemosyne* [13]. On this basis, *P. turatii* was isolated from this complex as a separate species [30,31]. Our data show that the structure of this group is even more complex and includes another deeply differentiated lineage, namely, *P. nubilosus*. The study of morphology shows that this lineage has a set of almost fixed differences in the pattern of the wings, the different shapes of the valva in males, and a fixed difference in the shape of the sphragis in females. The latter feature is traditionally considered species-specific [1,2], although it should be borne in mind that such a structure is characteristic of the so-called complete sphragis. Sometimes in nature (and, accordingly, in museums), females with frail or incomplete sphragides are found. Such incomplete sphragides are probably produced by males that had mated several times previously and thus had exhausted the resources necessary to produce a sphragis of normal bulk [3].

Theoretically, the complete allopatry of the taxa *P. mnemosyne* and *P. nubilosus* and the parapatry of *P. mnemosyne* and *P. turatii* allow them to be interpreted as subspecies of a single species. However, from the point of view of the criteria we proposed earlier [28], and taking into account the fact that the *COI* threshold higher than 2.0% genetic distance produces molecular groupings largely consistent with traditional, morphologically defined species [77,78,79], they should be interpreted as species.

Our proposed classification of *P. mnemosyne* sensu stricto as a system of four subspecies contradicts the traditional systems adopted in many studies [1,29], in which the number of subspecies can approach 200 [29]. We cannot exclude that a more detailed study, based on more comprehensive material and genome-wide markers, will reveal a more complex structure in the four subspecies we suggested and the presence of additional sublineages that can be interpreted as subspecies. In principle, even from the material we present in this study, it is clear that *P. mnemosyne adolphi* includes three differentiated sublineages: (1) a sublineage from SE Turkey and the Levant, (2) a sublineage from the mountains of Southern Zagros, and (3) a sublineage from southern Transcaucasia and northern Iran. There is also no doubt that *P. mnemosyne mnemosyne* from Europe has a finer genetic sub-structure [21,32,34]. For example, Bayesian clustering analyses based on allozymes supported the presence of three main genetic lineages in the Carpathian Basin [34]. However, we are convinced that the number of these valid subspecies is not as large as is believed [29], and even if the additional subspecies are found, they will be part of the four subspecies we have identified. Therefore, it is possible that the four subspecies we suggested can be interpreted as groups of subspecies or even separate allopatric species. The latter is especially likely in relation to *P. mnemosyne adolphi*, which has a particularly high level of genetic differentiation and has previously been considered as a candidate for a separate species [13,30].

The most intriguing question in the evolution of the *P. mnemosyne* species complex is the origin and loss of a specialized reduced wing pattern without red ocelli on the hindwings. Although the red-spotted wing pattern undoubtedly predominates in the genus *Parnassius* as a whole [1,2], our data show that the white-black pattern without red ocelli is ancestral for the *P. mnemosyne* species group and that the red ocelli evolved in *P. ariadne* de novo as a reversion to the wild type. The re-appearance of the red spots is observed in the secondary contact zone in Altai, where the western branch of the complex, represented by the species *P. ariadne*, overlaps with the eastern branch, represented by the species *P. stubbendorfii*. These two species sporadically hybridize in Altai [80], and we hypothesize that the reversion to the red-ocelli of *P. ariadne* is the result of increased prezygotic isolation in order to avoid the maladaptive hybridization [81]. Like any other evolutionary hypothesis, this assumption is difficult to test experimentally, and testing using comparative phylogenetic analysis [82] methods requires a larger number of pairs of species. However, two points should be emphasized here. First, living in sympatry between the two sister lineages is correlated with clear, fixed differences between them in the male wing color, a feature on which the behavioral mechanism of prezygotic isolation in butterflies is based [81]. Second, these interspecific differences arose in situ—directly in the zone of secondary contact between the species. Both points correspond exactly to the predictions of reinforcement theory [82].

We can also hypothesize that interspecific hybridization between the western and eastern subclades could be a trigger for the re-appearance of the red ocelli in *P. ariadne*. As our analysis shows, the ancestors of both western and eastern subclades did not have red ocelli; however, as is known, distant hybridization can result in characters that were absent in the parental forms [83].

## Figures and Tables

**Table 1:** Minimum *COI* p-distances (%) between the taxa of the subgenus *Driopa*. Taxon names are given in full (left column) or as abbreviations (first row).

	*ori*	*gig*	*mne*	*ado*	*nub*	*tur*	*ari*	*stu*	*hoe*	*gla*	*clo*	*eve*	*nor*
*falsa*	0												
*gigantea*	1.22												
*mnemosyne*	0.92	0.92											
*adolphi*	2.45	2.45	2.14										
*nubilosus*	2.91	2.29	2.60	3.52									
*turatii*	3.24	2.93	2.78	3.70	2.78								
*ariadne*	3.37	3.67	3.36	3.06	3.52	3.55							
*stubbendorfii*	4.74	4.43	4.74	5.35	4.28	4.17	5.05						
*hoenei*	5.09	5.09	4.78	5.25	3.70	4.32	5.25	4.01					
*glacialis*	6.29	5.98	6.29	6.76	5.66	6.51	7.08	4.38	6.03				
*clodius*	3.52	3.52	3.82	3.82	3.98	3.86	3.82	3.81	4.78	5.94			
*eversmanni*	3.98	3.67	3.67	3.98	3.52	3.86	3.98	3.65	4.46	5.48	1.98		
*nordmanni*	5.58	5.89	5.58	6.36	4.81	5.43	5.43	5.12	5.74	7.18	4.34	4.65	0

## Data Availability

All the analyzed DNA sequences are available via the GenBank links provided.

## Supplementary Materials

The following supporting information can be downloaded at: https://www.mdpi.com/article/10.3390/insects14120942/s1, Supplementary Material S1: List of the studied voucher samples; Supplementary Material S2: The alignment of the analyzed COI sequences; Supplementary Material S3 Reconstruction of ancestral states.

## Appendix A. List of the Studied Samples and Obtained *COI* Sequences

**BOLD ID**	**GenBank ID**	**Haplotype**	**Identification**	**Country**	**Region**
LOWAM001-08	OR822545	gig1	*mnemosyne*	Kyrgyzstan	Alai Mts
LOWAM002-08	OR822537	gig1	*mnemosyne*	Kyrgyzstan	Alai Mts
LOWAM003-08	OR822507	gig1	*mnemosyne*	Kyrgyzstan	Alai Mts
LOWAM004-08	OR822401	gig2	*mnemosyne*	Uzbekistan	Gissar Mts
LOWAM005-08	OR822544	gig2	*mnemosyne*	Uzbekistan	Gissar Mts
LOWAM006-08	OR822581	gig2	*mnemosyne*	Uzbekistan	Gissar Mts
LOWAM007-08	OR822585	ori1	*mnemosyne*	Kyrgyzstan	Moldatoo Mts
LOWAM008-08	OR822475	ori1	*mnemosyne*	Kyrgyzstan	Moldatoo Mts
LOWAM009-08	OR822518	ori2	*mnemosyne*	Kyrgyzstan	Moldatoo Mts
LOWAM010-08	OR822548	gig2	*mnemosyne*	Tajikistan	Turkestanski Mts
LOWAM011-08	OR822588	gig2	*mnemosyne*	Tajikistan	Turkestanski Mts
LOWAM012-08	OR822423	gig2	*mnemosyne*	Tajikistan	Turkestanski Mts
LOWAM013-08	OR822478	mne5	*mnemosyne*	Turkey	Ovitdagi Gecidi
LOWAM014-08	OR822546	mne6	*mnemosyne*	Turkey	Ovitdagi Gecidi
LOWAM015-08	OR822577	mne3	*mnemosyne*	Russia	Don River
LOWAM016-08	OR822394	mne3	*mnemosyne*	Russia	Don River
LOWAM017-08	OR822413	gig1	*mnemosyne*	Kyrgyzstan	Alai Mts
LOWAM018-08	OR822525	gig6	*mnemosyne*	Kyrgyzstan	Alai Mts
LOWAM019-08	OR822524	gig4	*mnemosyne*	Kyrgyzstan	Alai Mts
LOWAM020-08	OR822403	mne3	*mnemosyne*	Russia	Saratovskaya Oblast
LOWAM021-08	OR822586	mne2	*mnemosyne*	Russia	Saratovskaya Oblast
LOWAM022-08	OR822563	mne3	*mnemosyne*	Russia	Saratovskaya Oblast
LOWAM023-08	OR822554	mne4	*mnemosyne*	Russia	Saratovskaya Oblast
LOWAM024-08	OR822454	mne3	*mnemosyne*	Russia	Saratovskaya Oblast
LOWAM025-08	OR822543	mne3	*mnemosyne*	Russia	Saratovskaya Oblast
LOWAM026-08	OR822532	gig2	*mnemosyne*	Uzbekistan	W Gissar Mts
LOWAM027-08	OR822515	ori3	*mnemosyne*	Kazakhstan	Karatau Mts
LOWAM028-08	OR822520	ori3	*mnemosyne*	Kazakhstan	Karatau Mts
LOWAM029-08	OR822502	gig2	*mnemosyne*	Tajikistan	Taribak
LOWAM030-08	OR822471	gig2	*mnemosyne*	Tajikistan	Taribak
LOWAM031-08	OR822490	gig2	*mnemosyne*	Tajikistan	Taribak
LOWAM032-08	OR822523	gig2	*mnemosyne*	Uzbekistan	Nuratau Mts
LOWAM033-08	OR822421	gig2	*mnemosyne*	Uzbekistan	Nuratau Mts
LOWAM034-08	OR822419	gig2	*mnemosyne*	Uzbekistan	Nuratau Mts
LOWAM036-08	OR822448	mne2	*mnemosyne*	Russia	Pskovskaya Oblast
LOWAM037-08	OR822459	mne4	*mnemosyne*	Russia	Pskovskaya Oblast
LOWAM038-08	OR822452	ori4	*mnemosyne*	Kyrgyzstan	Kirgizsky Khrebet
LOWAM039-08	OR822428	ori5	*mnemosyne*	Kyrgyzstan	Kirgizsky Khrebet
LOWAM040-08	OR822506	ori4	*mnemosyne*	Kyrgyzstan	Kirgizsky Khrebet
LOWAM041-08	OR822406	ori1	*mnemosyne*	Kyrgyzstan	Alabel Pass
LOWAM042-08	OR822465	mne3	*mnemosyne*	Russia	Belgorodskaya oblast
LOWAM043-08	OR822426	mne3	*mnemosyne*	Russia	Belgorodskaya oblast
LOWAM044-08	OR822503	mne1	*mnemosyne*	Russia	Belgorodskaya oblast
LOWAM045-08	OR822481	gig7	*mnemosyne*	Tajikistan	Turkestanski Mts
LOWAM046-08	OR822493	gig7	*mnemosyne*	Tajikistan	Turkestanski Mts
LOWAM047-08	OR822538	gig7	*mnemosyne*	Tajikistan	Turkestanski Mts
LOWAM048-08	OR822444	gig2	*mnemosyne*	Tajikistan	Turkestanski Mts
LOWAM049-08	OR822411	gig2	*mnemosyne*	Tajikistan	Turkestanski Mts
LOWAM050-08	OR822408	gig2	*mnemosyne*	Tajikistan	Turkestanski Mts
LOWAM051-08	OR822590	gig8	*mnemosyne*	Tajikistan	Revad
LOWAM052-08	OR822477	gig2	*mnemosyne*	Tajikistan	
LOWAM055-08	OR822420	gig8	*mnemosyne*	Tajikistan	Gissar Mts
LOWAM056-08	OR822404	gig2	*mnemosyne*	Tajikistan	Gissar Mts
LOWAM057-08	OR822480	gig2	*mnemosyne*	Tajikistan	Gissar Mts
LOWAM058-08	OR822508	ori1	*mnemosyne*	Kazakhstan	Karzhantau Mts
LOWAM059-08	OR822460	ori1	*mnemosyne*	Kazakhstan	Karzhantau Mts
LOWAM060-08	OR822418	ori1	*mnemosyne*	Kazakhstan	Karzhantau Mts
LOWAM061-08	OR822425	ori1	*mnemosyne*	Kazakhstan	Saryaigyr
LOWAM062-08	OR822587	gig2	*mnemosyne*	Tajikistan	Gissar Mts (West)
LOWAM063-08	OR822469	gig8	*mnemosyne*	Tajikistan	Gissar Mts (West)
LOWAM064-08	OR822447	ori9	*mnemosyne*	Kazakhstan	Kirgizsky Khrebet
LOWAM065-08	OR822517	ori9	*mnemosyne*	Kazakhstan	Kirgizsky Khrebet
LOWAM066-08	OR822552	ori9	*mnemosyne*	Kazakhstan	Kirgizsky Khrebet
LOWAM067-08	OR822415	ori6	*mnemosyne*	Kazakhstan	Kirgizsky Khrebet
LOWAM069-08	OR822551	mne7	*mnemosyne*	Russia	W Caucasus
LOWAM070-08	OR822533	mne5	*mnemosyne*	Russia	W Caucasus
LOWAM072-08	OR822571	ori1	*mnemosyne*	Kyrgyzstan	Chatkalsky Khrebet
LOWAM073-08	OR822453	ori7	*mnemosyne*	Kyrgyzstan	Takhtalyk
LOWAM074-08	OR822491	ori5	*mnemosyne*	Kyrgyzstan	Naryn-Too Mts
LOWAM075-08	OR822570	ori1	*mnemosyne*	Kyrgyzstan	Naryn-Too Mts
LOWAM076-08	OR822498	ori5	*mnemosyne*	Kyrgyzstan	Ak-Muz
LOWAM077-08	OR822557	ori2	*mnemosyne*	Kyrgyzstan	Moldatoo Mts
LOWAM078-08	OR822516	ori5	*mnemosyne*	Kyrgyzstan	Moldatoo Mts
LOWAM079-08	OR822439	ori9	*mnemosyne*	Kyrgyzstan	Moldatoo Mts
LOWAM080-08	OR822500	ori5	*mnemosyne*	Kyrgyzstan	Songkel Lake
LOWAM081-08	OR822463	ori5	*mnemosyne*	Kyrgyzstan	Songkel Lake
LOWAM082-08	OR822436	ori5	*mnemosyne*	Kyrgyzstan	Songkel Lake
LOWAM086-08	OR822567	nub1	*nubilosus*	Turkmenistan	Kopetdagh
LOWAM087-08	OR822476	gig8	*mnemosyne*	Uzbekistan	Gissar Mts (West)
LOWAM088-08	OR822432	gig2	*mnemosyne*	Uzbekistan	Gissar Mts (West)
LOWAM089-08	OR822505	gig2	*mnemosyne*	Uzbekistan	Gissar Mts (West)
LOWAM090-08	OR822528	ori1	*mnemosyne*	Kyrgyzstan	Chychkan
LOWAM091-08	OR822501	ori1	*mnemosyne*	Kyrgyzstan	Chychkan
LOWAM092-08	OR822510	ori1	*mnemosyne*	Kyrgyzstan	Chychkan
LOWAM093-08	OR822565	ori5	*mnemosyne*	Kyrgyzstan	between Alabel and Tjuz-Ashu passes
LOWAM094-08	OR822391	ori5	*mnemosyne*	Kyrgyzstan	Kirgizsky Khrebet
LOWAM095-08	OR822527	ori5	*mnemosyne*	Kyrgyzstan	Kirgizsky Khrebet
LOWAM096-08	OR822499	gig9	*mnemosyne*	Tajikistan	Gissar Mts (SW)
LOWAM097-08	OR822574	gig9	*mnemosyne*	Tajikistan	Gissar Mts (SW)
LOWAM098-08	OR822399	gig9	*mnemosyne*	Tajikistan	Gissar Mts (SW)
LOWAM099-08	OR822461	ori8	*mnemosyne*	Uzbekistan	Kuraminsky Khrebet
LOWAM100-08	OR822560	ori5	*mnemosyne*	Kyrgyzstan	Ferganski Khrebet
LOWAM101-08	OR822450	ori5	*mnemosyne*	Kyrgyzstan	Ferganski Khrebet
LOWAM102-08	OR822579	ori5	*mnemosyne*	Kyrgyzstan	Ferganski Khrebet
LOWAM103-08	OR822550	ori1	*mnemosyne*	Kazakhstan	Turaigyr
LOWAM104-08	OR822529	ori1	*mnemosyne*	Kazakhstan	Turaigyr
LOWAM105-08	OR822561	ori1	*mnemosyne*	Kazakhstan	Turaigyr
LOWAM106-08	OR822485	ori8	*mnemosyne*	Uzbekistan	S of Tashkent
LOWAM107-08	OR822564	gig2	*mnemosyne*	Uzbekistan	Seravshaski Khrebet
LOWAM111-08	OR822431	ori5	*mnemosyne*	Kyrgyzstan	Naryn-Too Mts
LOWAM112-08	OR822430	ori5	*mnemosyne*	Kyrgyzstan	Naryn-Too Mts
LOWAM115-08	OR822592	ado1	*mnemosyne*	Azerbaijan	Nakhichevan
LOWAM116-08	OR822566	ado2	*mnemosyne*	Iran	Elburs Mts
LOWAM117-08	OR822397	ado2	*mnemosyne*	Iran	Elburs Mts
LOWAM118-08	OR822396	mne1	*mnemosyne*	Russia	Belgorod Region
LOWAM119-08	OR822526	mne1	*mnemosyne*	Russia	Belgorod Region
LOWAM120-08	OR822486	mne3	*mnemosyne*	Russia	Belgorod Region
LOWAM121-08	OR822416	mne3	*mnemosyne*	Russia	Saratovskaya Oblast
LOWAM122-08	OR822542	mne3	*mnemosyne*	Russia	Saratovskaya Oblast
LOWAM123-08	OR822553	mne3	*mnemosyne*	Russia	Saratovskaya Oblast
LOWAM124-08	OR822479	ori5	*mnemosyne*	Kyrgyzstan	Ak-Muz
LOWAM125-08	OR822445	ori5	*mnemosyne*	Kyrgyzstan	Ak-Muz
LOWAM126-08	OR822539	gig1	*mnemosyne*	Kyrgyzstan	Alai Mts
LOWAM127-08	OR822511	gig1	*mnemosyne*	Kyrgyzstan	Alai Mts
LOWAM128-08	OR822578	gig1	*mnemosyne*	Kyrgyzstan	Alai Mts
LOWAM129-08	OR822457	gig8	*mnemosyne*	Tajikistan	Turkestanski Mts
LOWAM130-08	OR822575	gig2	*mnemosyne*	Tajikistan	Turkestanski Mts
LOWAM131-08	OR822562	gig2	*mnemosyne*	Tajikistan	Turkestanski Mts
LOWAM132-08	OR822496	gig2	*mnemosyne*	Tajikistan	Turkestanski Mts
LOWAM133-08	OR822488	gig2	*mnemosyne*	Uzbekistan	Gissar Mts West
LOWAM134-08	OR822559	gig2	*mnemosyne*	Uzbekistan	Gissar Mts West
LOWAM135-08	OR822462	gig9	*mnemosyne*	Uzbekistan	Gissar Mts West
LOWAM136-08	OR822451	gig3	*mnemosyne*	Uzbekistan	Gissar Mts
LOWAM137-08	OR822449	gig2	*mnemosyne*	Uzbekistan	Gissar Mts
LOWAM138-08	OR822556	gig9	*mnemosyne*	Uzbekistan	Gissar Mts
LOWAM139-08	OR822434	gig9	*mnemosyne*	Tajikistan	Gissar Mts SW
LOWAM140-08	OR822473	gig2	*mnemosyne*	Tajikistan	Gissar Mts
LOWAM141-08	OR822521	gig11	*mnemosyne*	Tajikistan	Gissar Mts
LOWAM142-08	OR822417	ori2	*mnemosyne*	Kyrgyzstan	Modatoo Mts
LOWAM143-08	OR822402	ori1	*mnemosyne*	Kyrgyzstan	Modatoo Mts
LOWAM144-08	OR822582	ori1	*mnemosyne*	Kyrgyzstan	Modatoo Mts
LOWAM145-08	OR822458	ori2	*mnemosyne*	Kyrgyzstan	Moldatoo Mts
LOWAM146-08	OR822536	ori2	*mnemosyne*	Kyrgyzstan	Moldatoo Mts
LOWAM147-08	OR822519	ori2	*mnemosyne*	Kyrgyzstan	Moldatoo Mts
LOWAM148-08	OR822456	ori5	*mnemosyne*	Kyrgyzstan	Songkel Lake
LOWAM149-08	OR822464	ori9	*mnemosyne*	Kyrgyzstan	Songkel Lake
LOWAM150-08	OR822422	ori5	*mnemosyne*	Kyrgyzstan	Songkel Lake
LOWAM151-08	OR822433	ori4	*mnemosyne*	Kyrgyzstan	Kirgizsky Khrebet
LOWAM152-08	OR822409	ori1	*mnemosyne*	Kyrgyzstan	Kirgizsky Khrebet
LOWAM153-08	OR822443	gig1	*mnemosyne*	Kyrgyzstan	Alai Mts
LOWAM154-08	OR822535	gig1	*mnemosyne*	Kyrgyzstan	Alai Mts
LOWAM155-08	OR822541	gig1	*mnemosyne*	Kyrgyzstan	Alai Mts
LOWAM156-08	OR822522	ori1	*mnemosyne*	Kyrgyzstan	Chychkan
LOWAM157-08	OR822410	ori1	*mnemosyne*	Kyrgyzstan	Chychkan
LOWAM158-08	OR822470	ori1	*mnemosyne*	Kyrgyzstan	Chychkan
LOWAM159-08	OR822509	ori5	*mnemosyne*	Kazakhstan	Kirgizsky Khrebet
LOWAM160-08	OR822455	ori5	*mnemosyne*	Kazakhstan	Kirgizsky Khrebet
LOWAM161-08	OR822580	ori5	*mnemosyne*	Kazakhstan	Kirgizsky Khrebet
LOWAM162-08	OR822555	ori2	*mnemosyne*	Kazakhstan	Karzhantau Mts
LOWAM163-08	OR822483	ori1	*mnemosyne*	Kazakhstan	Karzhantau Mts
LOWAM164-08	OR822393	ori1	*mnemosyne*	Kazakhstan	Karzhantau Mts
LOWAM165-08	OR822395	gig2	*mnemosyne*	Tajikistan	Taribak
LOWAM166-08	OR822583	gig2	*mnemosyne*	Tajikistan	Taribak
LOWAM167-08	OR822474	gig10	*mnemosyne*	Tajikistan	Taribak
LOWAM168-08	OR822487	gig2	*mnemosyne*	Uzbekistan	Nuratau Mts
LOWAM169-08	OR822576	gig2	*mnemosyne*	Uzbekistan	Nuratau Mts
LOWAM170-08	OR822497	gig10	*mnemosyne*	Uzbekistan	Nuratau Mts
LOWAM171-08	OR822424	gig2	*mnemosyne*	Tajikistan	Turkestanski Mts
LOWAM172-08	OR822429	gig2	*mnemosyne*	Tajikistan	Turkestanski Mts
LOWAM173-08	OR822530	gig10	*mnemosyne*	Tajikistan	Turkestanski Mts
LOWAM196-09	OR822494	nub1	*nubilosus*	Turkmenistan	West Kopetdagh
LOWAM197-09	OR822435	nub1	*nubilosus*	Turkmenistan	West Kopetdagh
LOWAM198-09	OR822540	ado3	*mnemosyne*	Turkey	Taurus (gory Tavr)
LOWAM199-09	OR822513	ado3	*mnemosyne*	Turkey	Taurus (gory Tavr)
LOWAM200-09	OR822584	mne5	*mnemosyne*	Georgia	Mestia
LOWAM201-09	OR822531	mne5	*mnemosyne*	Georgia	Mestia
LOWAM202-09	OR822467	mne4	*mnemosyne*	Russia	St. Petersburg region
LOWAM203-09	OR822414	mne4	*mnemosyne*	Russia	St. Petersburg region
LOWAM205-09	OR822512	ori1	*mnemosyne*	Kyrgyzstan	Kungey-Alatoo Mts
LOWAM206-09	OR822441	mne4	*mnemosyne*	Russia	Vologodskaya oblast
LOWAM207-09	OR822534	gig10	*mnemosyne*	Tajikistan	Obihingou
LOWAM208-09	OR822504	gig8	*mnemosyne*	Tajikistan	Peter The Great Mts
LOWAM209-09	OR822412	gig2	*mnemosyne*	Tajikistan	Gissar Mts
LOWAM210-09	OR822440	gig2	*mnemosyne*	Tajikistan	Gissar Mts
LOWAM212-09	OR822427	gig2	*mnemosyne*	Tajikistan	Gissar Mts
LOWAM213-09	OR822495	gig5	*mnemosyne*	Tajikistan	Alai Mts
BPALB154-16	OR822438	ado4	*mnemosyne*	Israel	Hermon
BPALB230-17	OR822392	gig2	*mnemosyne*	Tajikistan	
BPALB245-17	OR822591	gig2	*mnemosyne*	Tajikistan	
BPALB258-17	OR822442	gig1	*mnemosyne*	Tajikistan	
BPALB369-17	OR822468	gig1	*mnemosyne*	Tajikistan	
BPALB389-17	OR822484	gig1	*mnemosyne*	Tajikistan	
BPAL2225-13	OR822589	ado4	*mnemosyne*	Israel	N. Israel
BPAL2226-13	OR822405	ado4	*mnemosyne*	Israel	N. Israel
BPAL2794-15	OR822572	mne8	*mnemosyne*	Russia	Pskov
BPAL2795-15	OR822568	mne8	*mnemosyne*	Russia	Pskov
BPAL3195-16	OR822437	ado4	*mnemosyne*	Israel	Hermon
BPAL3196-16	OR822489	ado4	*mnemosyne*	Israel	Hermon
BPAL3358-16	OR822547	ado4	*mnemosyne*	Israel	Hermon Mt
BPAL2276-13	OR822466	nor1	*nordmanni*	Georgia	Adzharia
BPAL2277-13	OR822573	nor1	*nordmanni*	Georgia	Adzharia
BPAL2278-13	OR822398	nor1	*nordmanni*	Georgia	Adzharia
LOWAM269-11	OR822482	ari2	*ariadne*	Kazakhstan	West Altai
LOWAM273-11	OR822514	ari2	*ariadne*	Kazakhstan	West Altai
LOWAM287-11	OR822407	ari1	*ariadne*	Kazakhstan	Altai
LOWAM288-11	OR822446	ari1	*ariadne*	Kazakhstan	Altai
LOWAM289-11	OR822472	ari1	*ariadne*	Kazakhstan	Altai
LOWAM290-11	OR822400	ari1	*ariadne*	Kazakhstan	Altai
LOWAM292-11	OR822558	ari4	*ariadne*	Kazakhstan	Altai
LOWAM295-11	OR822492	ari3	*ariadne*	Kazakhstan	Altai
LOWAM296-11	OR822569	ari3	*ariadne*	Kazakhstan	Altai
LOWAM301-11	OR822549	ari2	*ariadne clarus*	Kazakhstan	Saur Mts
MA225	OR884225	ari6	*ariadne erlik*	Russia	N of Kosh-Agach
