# Supplementary material for: Taxonomic Structure and Wing Pattern Evolution in the Parnassius mnemosyne Species Complex (Lepidoptera, Papilionidae)
Source: Insects. 2023 Dec 12;14(12):942. doi: 10.3390/insects14120942 (PMC10744292; doi:10.3390/insects14120942)
Supplement: Supplementary file 1 [file insects-14-00942-s001.zip › Supplementary_Material_S3.pdf]

Supplementary Material S3. Reconstruction of ancestral states

To reconstruct the probabilities of ancestral states, a Bayesian approach was used as implemented in the program MrBayes3.2 (Ronquist et al., 2012) [43]. The states studied were coded as 0 (absence of red spots) and 1 (presence of red spots). These states were implemented into a matrix of molecular features. The probability of ancestral states for each node was calculated separately. The command blocks used to analyze ancestral states are provided below.

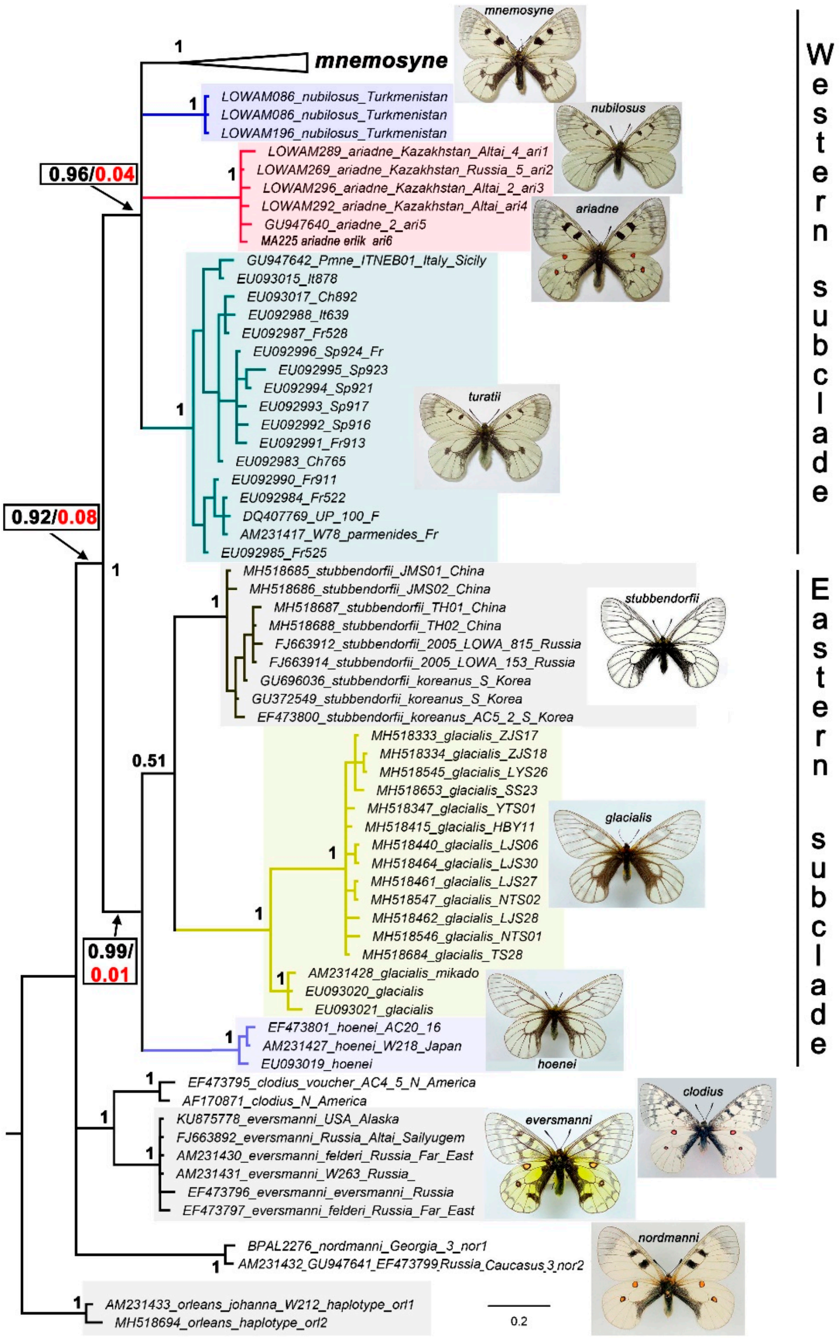

Figure S1. The Bayesian tree of the *Parnassius* (*Driopa*) species based on analysis of the mitochondrial *COI* barcodes. Numbers at nodes indicate Bayesian posterior probabilities (BPP) (higher than 0.5). Black/red values in the rectangles show the probabilities of the ancestral states "absence of red spots"/"presence of red spots" in the clades (1) (*mnemosyne* +*ariadne*) [0.96/0.04], (2) [(*mnemosyne* +*ariadne*)+(*stubbendorfii*+*glacialis*+*hoenei*)] [0.92/0.08] and (3) (*stubbendorfii*+*glacialis*+*hoenei*) [0.99/0.01]

(1) (*mnemosyne +ariadne*) clade

Probability of Ancestral state "no red spots" = 0.96±0.006

Parameter	Mean	Variance	Lower	Upper	Median	min ESS*	avg ESS	PSRF+
TL{all}	0.607933	0.003481	0.495735	0.718428	0.606854	190.49	191.39	0.999
r(A<->C){2}	0.049545	0.000215	0.025028	0.082874	0.048896	27.64	35.20	1.024
r(A<->G){2}	0.224390	0.001438	0.150871	0.297565	0.220734	22.81	35.43	1.001
r(A<->T){2}	0.039736	0.000063	0.025280	0.054317	0.038290	32.14	88.30	1.021
r(C<->G){2}	0.021513	0.000225	0.000421	0.055843	0.017037	31.28	38.51	1.000
r(C<->T){2}	0.644407	0.001830	0.561129	0.716927	0.646872	40.90	42.78	0.999
r(G<->T){2}	0.020410	0.000074	0.006173	0.036979	0.018717	43.86	64.22	1.000
pi(A){2}	0.302963	0.000283	0.269135	0.334567	0.304003	46.39	94.62	1.000
pi(C){2}	0.143621	0.000147	0.120124	0.167774	0.142642	59.51	66.54	1.020
pi(G){2}	0.142983	0.000151	0.117416	0.164763	0.142850	104.59	142.86	1.002
pi(T){2}	0.410433	0.000311	0.375599	0.443693	0.409026	99.23	113.17	1.007
alpha{2}	0.560108	0.065787	0.218632	1.068296	0.493593	30.88	46.96	0.999
pinvar{2}	0.493192	0.008850	0.324925	0.677312	0.503376	30.00	37.69	1.000
m{1}	4.982152	10.976314	0.202689	11.016320	4.349071	309.64	344.20	0.999
m{2}	0.993911	0.000026	0.984631	1.001159	0.994880	309.64	344.20	0.999
p(0){1@mnemosyne_clade}	0.034947	0.006044	0.000001	0.208347	0.005012	54.98	74.07	1.000
p(1){1@mnemosyne_clade}	0.965053	0.006044	0.790442	0.999999	0.994989	54.98	74.07	1.000

* Convergence diagnostic (ESS = Estimated Sample Size); min and avg values correspond to minimal and average ESS among runs. ESS value below 100 may indicate that the parameter is undersampled.

+ Convergence diagnostic (PSRF = Potential Scale Reduction Factor) should approach 1.0 as runs converge.

(2) [(*mnemosyne +ariadne*)+(*stubbendorfii+glacialis+hoenei*)] clade

Probability of Ancestral state "no red spots" = 0.92±0.019

Parameter	Mean	Variance	Lower	Upper	Median	min ESS*	avg ESS	PSRF+
TL{all}	0.598457	0.003300	0.479592	0.710142	0.596399	166.16	200.55	1.004
r(A<->C){2}	0.050037	0.000232	0.018536	0.076674	0.049107	34.07	43.13	0.999
r(A<->G){2}	0.223385	0.001893	0.149845	0.312143	0.220690	16.24	16.90	1.014
r(A<->T){2}	0.039723	0.000063	0.026157	0.055535	0.038529	62.44	63.13	1.006
r(C<->G){2}	0.024964	0.000411	0.000883	0.063662	0.019050	20.23	27.71	1.000

r(C<->T){2}	0.642032	0.002280	0.541108	0.721861	0.647017	9.97	14.31	1.010
r(G<->T){2}	0.019857	0.000066	0.006026	0.035787	0.018642	26.37	28.19	1.009
pi(A){2}	0.305557	0.000254	0.276933	0.340037	0.304288	60.95	71.88	1.001
pi(C){2}	0.144041	0.000131	0.123010	0.167293	0.144423	42.44	63.41	1.009
pi(G){2}	0.142076	0.000174	0.120838	0.170437	0.140927	32.97	45.58	1.012
pi(T){2}	0.408325	0.000241	0.380908	0.438656	0.409277	54.42	67.69	1.000
alpha{2}	0.557583	0.068148	0.180256	1.043169	0.494943	15.36	30.35	1.003
pinvar{2}	0.494900	0.009709	0.289974	0.672539	0.509138	9.92	21.80	1.000
m{1}	5.012332	12.059038	0.081419	11.808920	4.282275	336.75	365.77	1.001
m{2}	0.993865	0.000028	0.983473	1.001398	0.994982	336.75	365.77	1.001
p(0){1@mnemosyne_clade2}	0.083020	0.019270	0.000179	0.329243	0.036804	27.93	57.45	1.016
p(1){1@mnemosyne_clade2}	0.916980	0.019270	0.670498	0.999782	0.963255	27.93	57.45	1.016

----

* Convergence diagnostic (ESS = Estimated Sample Size); min and avg values

correspond to minimal and average ESS among runs.

ESS value below 100 may indicate that the parameter is undersampled.

\+ Convergence diagnostic (PSRF = Potential Scale Reduction Factor) should approach 1.0 as runs converge.

(3) (*stubbendorfii+glacialis+hoenei*)] clade

Probability of Ancestral state “no red spots” = 0.99±0.001

Parameter	Mean	Variance	Lower	Upper	Median	min ESS*	avg ESS	PSRF+
TL{all}	0.598549	0.003270	0.491822	0.717121	0.597192	163.85	224.87	1.008
r(A<->C){2}	0.053960	0.000229	0.023775	0.082434	0.052604	42.20	54.83	1.012
r(A<->G){2}	0.220837	0.001467	0.156058	0.295003	0.218292	17.32	21.17	1.003
r(A<->T){2}	0.040328	0.000064	0.023879	0.054431	0.039781	29.30	46.62	1.010
r(C<->G){2}	0.021933	0.000258	0.000465	0.052717	0.017961	34.18	51.02	1.001
r(C<->T){2}	0.642171	0.001755	0.552173	0.712290	0.641969	15.14	20.93	1.001
r(G<->T){2}	0.020771	0.000066	0.008143	0.034832	0.019767	63.47	66.40	1.000
pi(A){2}	0.304876	0.000285	0.269047	0.333987	0.304741	65.52	71.73	1.012
pi(C){2}	0.145445	0.000170	0.121690	0.169303	0.145278	63.99	66.19	1.008
pi(G){2}	0.142943	0.000176	0.117862	0.169670	0.142629	44.25	56.49	0.999
pi(T){2}	0.406736	0.000316	0.373710	0.442883	0.406925	60.22	74.09	1.001
alpha{2}	0.684629	0.094181	0.259595	1.354860	0.618408	22.32	32.77	1.081
pinvar{2}	0.532285	0.007768	0.373668	0.703313	0.545572	21.92	26.78	1.080
m{1}	4.929505	10.673132	0.572707	11.403790	4.290773	367.20	371.67	1.001
m{2}	0.993992	0.000025	0.984092	1.000653	0.994972	367.20	371.67	1.001
p(0){1@stubbendorfii_clade}	0.006969	0.000148	0.000001	0.028064	0.002567	110.77	143.72	1.000

p(1){1@stubbendorfii_clade} 0.993031 0.000148 0.971818 0.999999 0.997446 110.77 143.72 1.000

----

* Convergence diagnostic (ESS = Estimated Sample Size); min and avg values

correspond to minimal and average ESS among runs.

ESS value below 100 may indicate that the parameter is undersampled.

+ Convergence diagnostic (PSRF = Potential Scale Reduction Factor) should approach 1.0 as runs converge.

Alignment and command block for reconstruction of the ancestral states in the clade (1) (*mnemosyne* +*ariadne*)

```
#NEXUS

BEGIN DATA;
	DIMENSIONS  NTAX=125 NCHAR=655;
	format datatype=mixed(standard:1,DNA:2-655) interleave=yes gap=- missing=?;
	MATRIX
	Parnassius_orleans_AC10_5                    0
	LOWAM001_Kyrgyzstan_Alai_10                  1
	LOWAM004_Uzbekistan_Gissar_38                1
	LOWAM007_Kyrgyzstan_TianShan24               1
	LOWAM009_Kyrgyzstan_TianShan_6               1
	LOWAM013_Turkey_Georgia_3                    1
	LOWAM014_Turkey_Ovitdagi_1                   1
	LOWAM015_Don_Borisovka_Saratov               1
	LOWAM018_Kyrgyzstan_Karamyk                  1
	LOWAM019_Kyrgyzstan_Karamyk                  1
	LOWAM021_Russia_Saratov                      1
	LOWAM023_Saratov_Luga_Vologda_4              1
	LOWAM027_Kazakhstan_Karatau_2                1
	LOWAM037_Russia_Pskovskaya                   1
	LOWAM038_Kyrgyzstan_Tjuz_Ashu_3              1
	LOWAM039_Kyrgyzstan_23                       1
	LOWAM044_Russia_Borisovra_3                  1
	LOWAM045_Tajikistan_Shakhristan_3            1
	LOWAM067_Kazakhstan_Kaindy_1                 1
	LOWAM069_Russia_Teberda_2                    1
	LOWAM073_Kyrgyzstan_Takhtalyk1               1
	LOWAM074_Kyrgyzstan_Naryntoo                 1
	LOWAM086_Turkmenistan_3                      1
	LOWAM087_Uzbekistan_Sarchashma               1
	LOWAM095_Kyrgyzstan_Sosnovka                 1
	LOWAM096_Gissar_5                            1
	LOWAM098_Tajikistan_Khondiza1                1
	LOWAM099_Uzbekistan_Kamchik_2                1
	LOWAM100_Kyrgyzstan_Konduk                   1
	LOWAM115_Azerbaijan_Nyus_Nyus                1
	LOWAM116_Iran_Dizin_2                        1
	LOWAM122_Russia_Saratow_1                    1
	LOWAM136_Uzbekistan_Tamshush_1               1
	LOWAM149_Kyrgyzstan_Songkel_1                1
	LOWAM198_Turkey_Aladaglar_2                  1
	LOWAM213_Tajikistan_Tandukul_1               1
	Parnassius_clodius                           0
	Parnassius_eversmanni_2005_LOWA_108          0
	Parnassius_eversmanni_2005_LOWA_107          0
	Parnassius_stubbendorfii_2005_LOWA_815       1
	Parnassius_stubbendorfii_2005_LOWA_154       1
	Parnassius_stubbendorfii_2005_LOWA_153       1
	Parnassius_nordmanni                         0
	Parnassius_eversmanni                        0
	Parnassius_eversmanni_felderi                0
	Parnassius_ariadne_ariadne                   0
	Parnassius_glacialis_mikado                  1
	Parnassius_stubbendorfii_hoenei              1
	Parnassius_stubbendorfii_hoenei_AC20_16      1
	Parnassius_stubbendorfii_koreanus            1
	Parnassius_nordmanni_AC20_5                  0
	Parnassius_eversmanni_felderi_AC23_68        0
	Parnassius_eversmanni_eversmanni_AC1_14      0
```

Parnassius_clodius_AC4_5 0
Parnassius_ariadne_AC4_14 0
HQ004911_RV_07_C107_Romania 1
HQ004908_RV_07_D060_Romania 1
HQ004907_RV_08_M274_Romania 1
HQ004902_RV_08_M361_Romania 1
GU947642_Pmne_ITNEB01_Italy_Sicily 1
EU093018_Ca995 1
EU093017_Ch892 1
EU093016_Tk889 1
EU093015_It878 1
EU093014_Hu875 1
EU093013_Sk872_Cz_Slova 1
EU093011_Cz869 1
EU093010_Ru_Bosnia_Fi_Bu_Hu_Bel_Po_Rom_Ukr_ 1
EU093009_Kz848 1
EU093007_Fi810 1
EU093004_Tu999 1
EU093000_Bu961 1
EU092998_Bu951 1
EU092997_Ru940 1
EU092996_Sp924_Fr 1
EU092995_Sp923 1
EU092994_Sp921 1
EU092993_Sp917 1
EU092992_Sp916 1
EU092991_Fr913 1
EU092990_Fr911 1
EU092988_It639 1
EU092987_Fr528 1
EU092985_Fr525 1
EU092984_Fr522 1
EU092983_Ch765 1
EU092982_Pl653_Slova_Ukr 1
EU092981_At626 1
EU092980_At622 1
EU092978_At618_At_Slo 1
EU092977_At606_At_Cz_D_Fi_Hu_Po_Slova 1
EU092976_At602 1
EU092975_D757 1
EU092974_D756 1
EU092973_D744 1
EU092971_Hu697_Bu_Hu 1
EU092969_Bu775 1
EU092968_Bu772 1
EU093003_D982 1
DQ407769_UP_100_F 1
AM231426_W336_parvisi_Gre 1
AM231425_W333_angorae_Tur 1
AM231424_W331_angorae_Tur_Ankara 1
AM231423_W329_gigantea_Uzb_Chatkal 1
AM231422_W330_orientalis_Zailijskiy 1
AM231421_W292_ochracea_Tadj_Zeravsh_ 1
AM231420_W280_farsica_Iran_Fars 1
AM231419_W311_pseudonubilosus_Iran_Urmia 1
AM231418_W335_sheljuzhkoi_Tur_Adana 1
AM231417_W78_parmenides_Fr 1
EU836682_h_1_32_Rus 1
EU836681_h_1_31_Rus 1
EU836680_A_Bo_Bu_Cr_Cz_Hu_Sl_Li_Fi_Po_Ru 1
EU836675_h_1_19_It 1
EU836674_h_1_18_At_It 1
EU836672_h_1_10_It 1
EU836671_h_1_09_It 1
EU836670_h_1_07_Gre 1
EU836669_h_1_06_Gre 1
EU836668_h_1_05_Gre 1
EU836667_h_1_04_Gre 1
EU836666_h_1_03_Mac 1
EU836665_h_1_02_Gre_Mac 1
EU836664_h_2_06_At_It 1
EU836663_h_2_05_It 1

Parnassius_orleans_AC10_5 ------------------------
TGAGCAGGTATAATAGGAACCTCTTTAAGATTATTAATTCGTACTGAATTAGGTAATCCTGGATCTTTAATTGGAGATGATCAAATTTATAACACTA
TTGTAACAGCTCATGCTTTTATTATAATTTTTTTTATAGTTATACCAATTATAATTGGAGGATTTGGAAATTGATTAATTCCATTAATATTAGGAGC
CCCAGATATAGCTTTCCCCCGAATAAATAATATAAGATTTTGACTATTACCCCCCTCATTAACCTTATTAATTTCCAGAAGAATCGTAGAAAATGGA
GCAGGAACTGGATGAACAGTTTATCCCCCTTTATCCTCTAATATTGCCCATAGTGGAAGATCAGTTGATTTAGCTATCTTTTCTTTACATTTAGCTG
GAATTTCATCTATTTTAGGAGCTATTAATTTTATTACAACTATTATTAATATACGAATTAATCATATATCATTTGATCAAATACCCCTTTTCGTATG

AGCAGTAGGAATTACTGCTTTACTTTTATTATTATCTTTACCTGTTTTAGCAGGTGCTATTACTATACTTCTTACAGATCGAAATCTTAATACTTCA
TTTTTTGATCCAGCAGGAGGTGGAGATCCTATTTTATACCAACATTTA

LOWAM001_Kyrgyzstan_Alai_10
ACACTATATTTTATTTTTGGTATTTGAGCAGGTATAGTAGGAACCTCTTTAAGATTATTAATTCGTACTGAATTAGGTAATCCTGGATCTTTAATTG
GAGATGATCAAATTTATAATACTATTGTAACAGCTCATGCTTTTATTATAATTTTTTTCATAGTTATACCAATTATAATTGGAGGATTTGGAAATTG
ATTAATTCCTTTAATATTAGGAGCCCCAGATATAGCTTTTCCTCGAATAAATAATATAAGATTTTGATTACTACCCCCTTCATTAACTTTATTAATT
TCTAGAAGAATTGTAGAAAATGGAGCAGGAACAGGATGAACAGTTTATCCCCCTTTATCATCTAATATCGCTCATAGAGGTAGTTCAGTTGATTTAG
CTATTTTTTCTTTACATTTAGCAGGAATTTCATCAATTTTAGGAGCTATTAATTTCATTACAACTATTATTAATATACGAATTAACCATATATCATT
TGATCAAATACCCCTCTTTGTATGAGCAGTAGGAATTACTGCTTTACTTTTATTATTATCTTTACCTGTATTAGCTGGTGCTATTACTATATTATTA
ACAGATCGAAATCTTAATACTTCATTTTTTGATCCTGCAGGAGGTGGAGATCCTATTTTATATCAACATTTA

LOWAM004_Uzbekistan_Gissar_38
ACACTATATTTTATTTTTGGTATTTGAGCAGGTATAGTAGGAACCTCTTTAAGATTATTAATTCGTACTGAATTAGGTAATCCTGGATCTTTAATTG
GAGATGATCAAATTTATAATACTATTGTAACAGCTCATGCTTTTATTATAATTTTTTTCATAGTTATACCAATTATAATTGGAGGATTTGGAAATTG
ATTAATTCCTTTAATATTAGGAGCCCCAGATATAGCTTTTCCTCGAATAAATAATATAAGATTTTGATTACTACCCCCTTCATTAACTTTATTAATT
TCCAGAAGAATTGTAGAAAATGGAGCAGGAACAGGATGAACAGTTTATCCCCCTTTATCATCTAATATCGCTCATAGAGGTAGTTCAGTTGATTTAG
CTATTTTTTCTTTACATTTAGCAGGAATTTCATCAATTTTAGGAGCTATTAATTTCATTACAACTATTATTAATATACGAATTAACCATATATCATT
TGATCAAATACCCCTCTTTGTATGAGCAGTAGGAATTACTGCTTTACTTTTATTATTATCTTTACCTGTATTAGCTGGTGCTATTACTATATTATTA
ACAGATCGAAATCTTAATACTTCATTTTTTGATCCTGCAGGAGGTGGAGATCCTATTTTATATCAACATTTA

LOWAM007_Kyrgyzstan_TianShan24
ACACTATATTTTATTTTTGGTATTTGAGCAGGTATAGTAGGAACCTCTTTAAGATTATTAATTCGTACTGAATTAGGTAATCCTGGATCTTTAATTG
GAGATGATCAAATTTATAATACTATTGTAACAGCTCATGCTTTTATTATAATTTTTTTATAGTTATACCAATTATAATTGGAGGATTTGGAAATTG
ATTAATTCCTTTAATATTAGGGGCCCCAGATATAGCTTTCCTCGAATAAATAATATAAGATTTTGATTATTACCCCCTTCATTAACTTTATTAATT
TCTAGAAGAATTGTAGAAAATGGAGCAGGAACAGGATGAACAGTTTATCCCCCTTTATCATCTAATATCGCTCATAGAGGTAGTTCAGTTGATTTAG
CTATTTTTTCTTTACATTTAGCAGGAATTTCATCAATCTTAGGAGCTATTAATTTTATTACAACTATTATTAATATACGAATTAATCATATATCATT
TGATCAAATACCTCTCTTTGTATGAGCAGTAGGAATTACTGCTTTACTTTTATTACTATCTTTACCTGTATTAGCTGGTGCTATTACTATATTATTA
ACAGATCGAAATCTTAATACTTCATTTTTTGATCCTGCAGGAGGTGGAGATCCTATTTTATATCAACATTTA

LOWAM009_Kyrgyzstan_TianShan_6
ACACTATATTTTATTTTTGGTATTTGAGCAGGTATAGTAGGAACCTCTTTAAGATTATTAATTCGTACTGAATTAGGTAATCCTGGATCTTTGATTG
GAGATGATCAAATTTATAATACTATTGTAACAGCTCATGCTTTTATTATAATTTTTTTATAGTTATACCAATTATAATTGGAGGATTTGGAAATTG
ATTAATTCCTTTAATATTAGGGGCCCCAGATATAGCTTTTCCTCGAATAAATAATATAAGATTTTGATTATTACCCCCTTCATTAACTTTATTAATT
TCTAGAAGAATTGTAGAAAATGGAGCAGGAACAGGATGAACAGTTTATCCCCCTTTATCATCTAATATCGCTCATAGAGGTAGTTCAGTTGATTTAG
CTATTTTTTCTTTACATTTAGCAGGAATTTCATCAATCTTAGGAGCTATTAATTTTATTACAACTATTATTAATATACGAATTAATCATATATCATT
TGATCAAATACCTCTCTTTGTATGAGCAGTAGGAATTACTGCTTTACTTTTATTACTATCTTTACCTGTATTAGCTGGTGCTATTACTATATTATTA
ACAGATCGAAATCTTAATACTTCATTTTTTGATCCTGCAGGAGGTGGAGATCCTATTTTATATCAACATTTA

LOWAM013_Turkey_Georgia_3
ACACTATATTTTATTTTTGGTATTTGAGCAGGTATAGTAGGAACCTCTTTAAGATTATTAATTCGTACTGAATTAGGTAATCCTGGATCTTTAATTG
GAGATGATCAAATTTATAATACTATTGTAACAGCTCATGCTTTTATTATAATTTTTTTATAGTTATACCAATTATAATTGGAGGATTTGGAAATTG
ATTAATTCCTTTAATATTAGGAGCCCCAGATATAGCTTTTCCTCGAATAAATAATATAAGATTTTGATTACTGCCCCCCTCATTAACTTTATTAATT
TCTAGAAGAATTGTAGAAAATGGAGCAGGAACAGGATGAACAGTTTATCCCCCTTTATCATCTAATATCGCTCATAGAGGTAGTTCAGTTGATTTAG
CTATTTTTTCTTTACATTTAGCAGGAATTTCATCAATCTTAGGAGCTATTAATTTTATTACAACTATTATTAATATACGAATTAACCATATATCATT
TGATCAAATACCTCTCTTTGTATGAGCAGTAGGAATTACTGCTTTACTTTTATTATTATCTTTACCTGTATTAGCTGGTGCTATTACTATATTATTA
ACAGATCGAAATCTTAATACTTCATTTTTTGATCCTGCAGGAGGTGGAGATCCTATTTTATATCAACATTTA

LOWAM014_Turkey_Ovitdagi_1
ACACTATATTTTATTTTTGGTATTTGAGCAGGTATAGTAGGAACCTCTTTAAGATTATTAATTCGTACTGAATTAGGTAATCCTGGATCTTTAATTG
GAGATGATCAAATTTATAATACTATTGTAACAGCTCATGCTTTTATTATAATTTTTTTATAGTTATACCAATTATAATTGGAGGATTTGGAAATTG
ATTAATTCCTTTAATATTAGGAGCCCCAGATATAGCTTTTCCTCGAATAAATAATATAAGATTTTGATTACTGCCCCCCTCATTAACTTTATTAATT
TCCAGAAGAATTGTAGAAAATGGAGCAGGAACAGGATGAACAGTTTATCCCCCTTTATCATCTAATATCGCTCATAGAGGTAGTTCAGTTGATTTAG
CTATTTTTTCTTTACATTTAGCAGGAATTTCATCAATCTTAGGAGCTATTAATTTTATTACAACTATTATTAATATACGAATTAACCATATATCATT
TGATCAAATACCTCTCTTTGTATGAGCAGTAGGAATTACTGCTTTACTTTTATTATTATCTTTACCTGTATTAGCTGGTGCTATTACTATATTATTA
ACAGATCGAAATCTTAATACTTCATTTTTTGATCCTGCAGGAGGTGGAGATCCTATTTTATATCAACATTTA

LOWAM015_Don_Borisovka_Saratov
ACACTATATTTTATTTTTGGTATTTGAGCAGGTATAGTAGGAACCTCTTTAAGATTATTAATTCGTACTGAATTAGGTAATCCTGGATCTTTAATTG
GAGATGATCAAATTTATAATACTATTGTAACAGCTCATGCTTTTATTATAATTTTTTTATAGTTATACCAATTATAATTGGAGGATTTGGAAATTG
ATTAATTCCTTTAATATTAGGAGCCCCAGATATAGCTTTTCCTCGAATAAATAATATAAGATTTTGATTACTACCCCCCTCATTAACTTTATTAATT
TCTAGAAGAATTGTAGAAAATGGAGCAGGAACAGGATGAACAGTTTATCCCCCTTTATCATCTAATATCGCTCATAGAGGTAGTTCAGTTGATTTAG
CTATTTTTTCTTTACATTTAGCAGGAATTTCATCAATCTTAGGAGCTATTAATTTTATTACAACTATTATTAATATACGAATTAACCATATATCATT
TGATCAAATACCTCTCTTTGTATGAGCAGTAGGAATTACTGCTTTACTTTTATTATTATCTTTACCTGTATTAGCTGGTGCTATTACTATATTATTA
ACAGATCGAAATCTTAATACTTCATTTTTTGATCCTGCAGGAGGTGGGGATCCTATTTTATATCAACATTTA

LOWAM018_Kyrgyzstan_Karamyk
ACACTATATTTTATTTTTGGTATTTGAGCAGGTATAGTAGGAACCTCTTTAAGATTATTAATTCGTACTGAATTAGGTAATCCTGGATCTTTAATTG
GAGATGATCAAATTTATAATACTATTGTAACAGCTCATGCTTTTATTATAATTTTTTTATAGTTATACCAATTATAATTGGAGGATTTGGAAATTG
ATTAATTCCTTTAATATTAGGAGCCCCAGATATAGCTTTTCCTCGAATAAATAATATAAGATTTTGATTACTACCCCCTTCATTAACTTTATTAATT
TCTAGAAGAATTGTAGAAAATGGAGCAGGAACAGGATGAACAGTTTATCCCCCTTTATCATCTAATATCGCTCATAGAGGTAGTTCAGTTGATTTAG
CTATTTTTTCTTTACATTTAGCAGGAATTTCATCAATTTTAGGAGCTATTAATTTCATTACAACTATTATTAATATACGAATTAACCATATATCATT
TGATCAAATACCCCTCTTTGTATGAGCAGTAGGAATTACTGCTTTACTTTTATTATTATCTTTACCTGTATTAGCTGGTGCTATTACTATATTATTA
ACAGATCGAAATCTTAATACTTCATTTTTTGATCCAGCAGGAGGTGGAGATCCTATTTTATATCAACATTTA

LOWAM019_Kyrgyzstan_Karamyk
ACACTATATTTTATTTTTGGTATTTGAGCAGGTATAGTAGGAACCTCTTTAAGATTATTAATTCGTACTGAATTAGGTAATCCTGGATCTTTAATTG
GAGATGATCAAATTTATAATACTATTGTAACAGCTCATGCTTTTATTATAATTTTTTTCATAGTTATACCAATTATAATTGGAGGATTTGGAAATTG
ATTAATTCCTTTAATATTAGGAGCCCCAGATATAGCTTTTCCTCGAATAAATAATATAAGATTTTGATTACTACCCCCTTCATTAACTTTATTAATT
TCTAGAAGAATTGTAGAAAATGGAGCAGGAACAGGATGAACA?TTTATCCCCCTTTATCATCTAATATCGCTCATAGAGGTAGTTCAGTTGATTTAG

CTATTTTTTCTTTACATTTAGCAGGAATTTCATCAATTTTAGGGGCTATTAATTTCATTACAACTATTATTAATATACGAATTAACCATATATCATT
TGATCAAATACCCCTCTTTGTATGAGCAGTAGGAATTACTGCTTTACTTTTATTATTATCTTTACCTGTATTAGCTGGTGCTATTACTATATTATTA
ACAGATCGAAATCTTAATACTTCATTTTTTGATCCTGCAGGAGGTGGAGATCCTATTTTATATCAACATTTA

LOWAM021_Russia_Saratov
ACACTATATTTTATTTTTGGTATTTGAGCAGGTATAGTAGGAACCTCTTTAAGATTATTAATTCGTACTGAATTAGGTAATCCTGGATCTTTAATTG
GAGATGATCAAATTTATAATACTATTGTAACAGCTCATGCTTTTATTATAATTTTTTTTATAGTTATACCAATTATAATTGGAGGATTTGGAAATTG
ATTAATTCCTTTAATATTAGGAGCCCCAGATATAGCTTTTCCTCGAATAAATAATATAAGATTTTGATTACTACCCCCCTCATTAACTTTATTAATT
TCTAGAAGAATTGTAGAAAATGGAGCAGGAACAGGATGAACAGTTTATCCCCCTTTATCATCTAATATCGCTCATAGAGGTAGTTCAGTTGATTTAG
CTATTTTTTCTTTACATTTAGCAGGAATTTCATCAATCTTAGGAGCTATTAATTTTATTACAACTATTATTAATATACGAATTAACCATATATCATT
TGATCAAATACCTCTCTTTGTATGAGCAGTAGGAATTACTGCTTTACTTTTATTATTATCTTTACCTGTATTAGCTGGTGCTATTACTATATTATTA
ACAGATCGAAATCTTAATAATTCATTTTTTGATCCTGCAGGAGGTGGGGATCCTATTTTATATCAACATTTA

LOWAM023_Saratov_Luga_Vologda_4
ACACTATATTTTATTTTTGGTATTTGAGCAGGTATAGTAGGAACCTCTTTAAGATTATTAATTCGTACTGAATTAGGTAATCCTGGATCTTTAATTG
GAGATGATCAAATTTATAATACTATTGTAACAGCTCATGCTTTTATTATAATTTTTTTTATAGTTATACCAATTATAATTGGAGGATTTGGAAATTG
ATTAATTCCTTTAATATTAGGAGCCCCAGATATAGCTTTTCCTCGAATAAATAATATAAGATTTTGATTACTACCCCCCTCATTAACTTTATTAATT
TCTAGAAGAATTGTAGAAAATGGAGCAGGAACAGGATGAACAGTTTATCCCCCTTTATCATCTAATATCGCTCATAGAGGTAGTTCAGTTGATTTAG
CTATTTTTTCTTTACATTTAGCAGGAATTTCATCAATCTTAGGAGCTATTAATTTTATTACAACTATTATTAATATACGAATTAACCATATATCATT
TGATCAAATACCTCTCTTTGTATGAGCAGTAGGAATTACTGCTTTACTTTTATTATTATCTTTACCTGTATTAGCTGGTGCTATTACTATATTATTA
ACAGATCGAAATCTTAATACTTCATTTTTTGATCCTGCAGGAGGTGGAGATCCTATTTTATATCAACATTTA

LOWAM027_Kazakhstan_Karatau_2
ACACTATATTTTATTTTTGGTATTTGAGCAGGTATAGTAGGAACCTCTTTAAGATTATTAATTCGTACTGAATTAGGTAATCCTGGATCTTTAATTG
GAGATGATCAAATTTATAATACTATTGTAACAGCTCATGCTTTTATTATAATTTTTTTTATAGTTATACCAATTATAATTGGAGGATTTGGAAATTG
ATTAATTCCTTTAATATTAGGGGCCCCAGATATAGCTTTTCCTCGAATAAATAATATAAGATTTTGATTATTACCCCCTTCATTAACTTTATTAATT
TCTAGAAGAATTGTAGAAAATGGAGCAGGAACAGGATGAACAGTTTATCCCCCTTTATCATCTAATATCGCTCATAGAGGTAGTTCAGTTGATTTAG
CTATTTTTTCTTTACATTTAGCAGGAATTTCATCAATCTTAGGAGCTATTAATTTTATTACAACTATTATTAATATACGAATTAATCATATATCATT
TGATCAAATACCTCTCTTTGTATGAGCAGTAGGAATTACTGCTTTACTTTTATTACTATCCTTACCTGTATTAGCTGGTGCTATTACTATATTATTA
ACAGATCGAAATCTTAATACTTCATTTTTTGATCCTGCAGGAGGTGGAGATCCTATTTTATATCAACATTTA

LOWAM037_Russia_Pskovskaya ---------
TTTATTTTTGGTATTTGAGCAGGTATAGTAGGAACCTCTTTAAGATTATTAATTCGTACTGAATTAGGTAATCCTGGATCTTTAATTGGAGATGATC
AAATTTATAATACTATTGTAACAGCTCATGCTTTTATTATAATTTTTTTTATAGTTATACCAATTATAATTGGAGGATTTGGAAATTGATTAATTCC
TTTAATATTAGGAGCCCCAGATATAGCTTTTCCTCGAATAAATAATATAAGATTTTGATTACTACCCCCCTCATTAACTTTATTAATTTCTAGAAGA
ATTGTAGAAAATGGAGCAGGAACAGGATGAACAGTTTATCCCCCTTTATCATCTAATATCGCTCATAGAGGTAGTTCAGTTGATTTAGCTATTTTTT
CTTTACATTTAGCAGGAATTTCATCAATCTTAGGAGCTATTAATTTTATTACAACTATTATTAATATACGAATTAACCATATATCATTTGATCAAAT
ACCTCTCTTTGTATGAGCAGTAGGAATTACTGCTTTACTTTTATTATTATCTTTACCTGTATTAGCTGGTGCTATTACTATATTATTAACAGATCGA
AATCTTAATACTTCATTTTTTGATCCTGCAGGAGGTGGAGATCCTATTTTATATCAACATTTA

LOWAM038_Kyrgyzstan_Tjuz_Ashu_3
ACACTATATTTTATTTTTGGTATTTGAGCAGGTATAGTAGGAACCTCTTTAAGATTATTAATTCGTACTGAATTAGGTAATCCTGGATCTTTAATTG
GAGATGATCAAATTTATAATACTATTGTAACAGCTCATGCTTTTATTATAATTTTTTTTATAGTTATACCAATTATAATTGGAGGATTTGGAAATTG
ATTAATTCCTTTAATATTAGGGGCCCCAGATATAGCTTTTCCTCGAATAAATAATATAAGATTTTGATTATTACCCCCTTCATTAACTTTATTAATT
TCTAGAAGAATTGTAGAAAATGGAGCAGGAACAGGATGAACAATTTATCCCCCTTTATCATCTAATATCGCTCATAGAGGTAGTTCAGTTGATTTAG
CTATTTTTTCTTTACATTTAGCGGGAATTTCATCAATCTTAGGAGCTATTAATTTTATTACAACTATTATTAATATACGAATTAATCATATATCATT
TGATCAAATACCTCTCTTTGTATGAGCAGTAGGAATTACTGCTTTACTTTTATTACTATCTTTACCTGTATTAGCTGGTGCTATTACTATATTATTA
ACAGATCGAAATCTTAATACTTCATTTTTTGATCCTGCAGGAGGTGGAGATCCTATTTTATATCAACATTTA

LOWAM039_Kyrgyzstan_23
ACACTATATTTTATTTTTGGTATTTGAGCAGGTATAGTAGGAACCTCTTTAAGATTATTAATTCGTACTGAATTAGGTAATCCTGGATCTTTAATTG
GAGATGATCAAATTTATAATACTATTGTAACAGCTCATGCTTTTATTATAATTTTTTTTATAGTTATACCAATTATAATTGGAGGATTTGGAAATTG
ATTAATTCCTTTAATATTAGGGGCCCCAGATATAGCTTTTCCTCGAATAAATAATATAAGATTTTGATTATTACCCCCTTCATTAACTTTATTAATT
TCTAGAAGAATTGTAGAAAATGGAGCAGGAACAGGATGAACAGTTTATCCCCCTTTATCATCTAATATCGCTCATAGAGGTAGTTCAGTTGATTTAG
CTATTTTTTCTTTACATTTAGCGGGAATTTCATCAATCTTAGGAGCTATTAATTTTATTACAACTATTATTAATATACGAATTAATCATATATCATT
TGATCAAATACCTCTCTTTGTATGAGCAGTAGGAATTACTGCTTTACTTTTATTACTATCTTTACCTGTATTAGCTGGTGCTATTACTATATTATTA
ACAGATCGAAATCTTAATACTTCATTTTTTGATCCTGCAGGAGGTGGAGATCCTATTTTATATCAACATTTA

LOWAM044_Russia_Borisovra_3
ACACTATATTTTATTTTTGGTATTTGAGCAGGTATAGTAGGAACCTCTTTAAGATTATTAATTCGTACTGAATTAGGTAATCCTGGATCTTTAATTG
GAGATGATCAAATTTATAATACTATTGTAACAGCTCATGCTTTTATTATAATTTTTTTTATAGTTATACCAATTATAATTGGAGGATTTGGAAATTG
ATTAATTCCCTTAATATTAGGAGCCCCAGATATAGCTTTTCCTCGAATAAATAATATAAGATTTTGATTACTACCCCCCTCATTAACTTTATTAATT
TCTAGAAGAATTGTAGAAAATGGAGCAGGAACAGGATGAACAGTTTATCCCCCTTTATCATCTAATATCGCTCATAGAGGTAGTTCAGTTGATTTAG
CTATTTTTTCTTTACATTTAGCAGGAATTTCATCAATCTTAGGAGCTATTAATTTTATTACAACTATTATTAATATACGAATTAACCATATATCATT
TGATCAAATACCTCTCTTTGTATGAGCAGTAGGAATTACTGCTTTACTTTTATTATTATCTTTACCTGTATTAGCTGGTGCTATTACTATATTATTA
ACAGATCGAAATCTTAATACTTCATTTTTTGATCCTGCAGGAGGTGGAGATCCTATTTTATATCAACATTTA

LOWAM045_Tajikistan_Shakhristan_3
ACACTATATTTTATTTTTGGTATTTGAGCAGGTATAGTAGGAACCTCTTTAAGATTATTAATTCGTACTGAATTAGGTAATCCTGGATCTTTAATTG
GAGATGATCAAATTTATAATACTATTGTAACAGCTCATGCTTTTATTATAATTTTTTTCATAGTTATACCAATTATAATTGGAGGATTTGGAAATTG
ATTAATTCCTTTAATATTAGGAGCCCCAGATATAGCTTTTCCTCGAATAAATAATATAAGATTTTGATTACTACCCCCTTCATTAACTTTATTAATT
TCCAGAAGAATTGTAGAAAATGGAGCAGGAACAGGATGAACAGTTTATCCCCCTTTATCATCTAATATTGCTCATAGAGGTAGTTCAGTTGATTTAG
CTATTTTTTCTTTACATTTAGCAGGAATTTCATCAATTTTAGGAGCTATTAATTTCATTACAACTATTATTAATATACGAATTAACCATATATCATT
TGATCAAATACCCCTCTTTGTATGAGCAGTAGGAATTACTGCTTTACTTTTATTATTATCTTTACCTGTATTAGCTGGTGCTATTACTATATTATTA
ACAGATCGAAATCTTAATACTTCATTTTTTGATCCTGCAGGAGGTGGAGATCCTATTTTATATCAACATTTA

LOWAM067_Kazakhstan_Kaindy_1
ACACTATATTTTATTTTTGGTATTTGAGCAGGTATAGTAGGAACCTCTTTAAGATTATTAATTCGTACTGAATTAGGTAATCCTGGATCTTTAATTG
GAGATGATCAAATTTATAATACTATTGTAACAGCTCATGCTTTTATTATAATTTTTTTTATAGTTATACCAATTATAATTGGAGGATTTGGAAATTG
ATTAATTCCTTTAATATTAGGGGCCCCAGATATAGCTTTTCCTCGAATAAATAATATAAGATTTTGATTATTACCCCCTTCATTAACTTTATTAATT

TCTAGAATAATTGTAGAAAATGGAGCAGGAACAGGATGAACAGTTTATCCCCCTTTATCATCTAATATCGCTCATAGAGGTAGTTCAGTTGATTTAG
CTATTTTTTCTTTACATTTAGCGGGAATTTCATCAATCTTAGGAGCTATTAATTTTATTACAACTATTATTAATATACGAATTAATCATATATCATT
TGATCAAATACCTCTCTTTGTATGAGCAGTAGGAATTACTGCTTTACTTTTATTACTATCTTTACCTGTATTAGCTGGTGCTATTACTATATTATTA
ACAGATCGAAATCTTAATACTTCATTTTTTGATCCTGCAGGAGGTGGAGATCCTATTTTATATCAACATTTA

LOWAM069_Russia_Teberda_2 ---------
TTTATTTTTGGTATTTGAGCAGGTATAGTAGGAACCTCTTTAAGATTATTAATTCGTACTGAATTAGGTAATCCTGGATCTTTAATTGGAGATGATC
AAATTTATAATACTATTGTAACAGCTCATGCTTTTATTATAATTTTTTTTATAGTTATACCAATTATAATTGGAGGATTTGGAAATTGATTAATTCC
TTTAATATTAGGAGCCCCAGATATAGCTTTTCCTCGAATAAATAATATAAGATTTTGATTACTGCCCCCCTCATTAACTTTATTAATTTCTAGAAGA
ATTGTAGAAAATGGAGCAGGAACAGGATGAACAGTTTATCCCCCTTTATCATCTAATATCGCTCATAGAGGTAGTTCAGTTGATTTAGCTATTTTTT
CTTTACATTTAGCAGGAATTTCATCAATCTTAGGAGCTATTAATTTTATTACAACTATTATTAATATACGAATTAATCATATATCATTTGATCAAAT
ACCTCTCTTTGTATGAGCAGTAGGAATTAC?GCTTTACTTTTATTATTATCTTTACCTGTATTAGCTGG?GCTATTACTATATTATTAACAGATCGA
AATCT?AATACTTCATTTTTTGATCCTGCAGGAGGAGGAGATCCTATTTTATATCAACATTTA

LOWAM073_Kyrgyzstan_Takhtalyk1
ACACTATATTTTATTTTTGGTATTTGAGCAGGTATAGTAGGAACCTCTTTAAGATTATTAATTCGTACTGAATTAGGTAATCCTGGATCTTTAATTG
GAGATGATCAAATTTATAATACTATTGTAACAGCTCATGCTTTTATTATAATTTTTTTTATAGTTATACCAATTATAATTGGAGGATTTGGAAATTG
ATTAATTCCTTTAATATTAGGGGCCCCAGATATAGCTTTTCCTCGAATAAATAATATAAGATTTTGATTATTACCCCCTTCATTAACTTTATTAATT
TCTAGAAGAATTGTAGAAAATGGAGCAGGAACAGGATGAACAGTTTATCCCCCTTTATCATCTAATATCGCTCATAGAGGTAGTTCAGTTGATTTAG
CTATTTTTTCTTTACATTTAGCGGGGATTTCATCAATCTTAGGAGCTATTAATTTTATTACAACTATTATTAATATACGAATTAATCATATATCATT
TGATCAAATACCTCTCTTTGTATGAGCAGTAGGAATTACTGCTTTACTTTTATTATTATCTTTACCTGTATTAGCTGGTGCTATTACTATATTATTA
ACAGATCGAAATCTTAATACTTCATTTTTTGATCCTGCAGGAGGTGGAGATCCTATTTTATATCAACATTTA

LOWAM074_Kyrgyzstan_Naryntoo
ACACTATATTTTATTTTTGGTATTTGAGCAGGTATAGTAGGAACCTCTTTAAGATTATTAATTCGTACTGAATTAGGTAATCCTGGATCTTTAATTG
GAGATGATCAAATTTATAATACTATTGTAACAGCTCATGCTTTTATTATAATTTTTTTTATAGTTATACCAATTATAATTGGAGGATTTGGAAATTG
ATTAATTCCTTTAATATTAGGGGCCCCAGATATAGCTTTTCCTCGAATAAATAATATAAGATTTTGATTATTACCCCCTTCATTAACTTTATTAATT
TCTAGAAGAATTGTAGAAAATGGAGCAGGAACAGGATGAACAGTTTATCCCCCTTTATCATCTAATATCGCTCATAGAGGTAGTTCAGTTGATTTAG
CTATTTTTTCTTTACATTTAGCGGGAATTTCATCAATCTTAGGAGCTATTAATTTTATTACAACTATTATTAATATACGAATTAATCATATATCATT
TGATCAAATACCTCTCTTTGTATGAGCAGTAGGAATTACTGCTTTACTTTTATTACTATCTTTACCTGTATTAGCTGGTGCTATTACTATATTATTA
ACAGATCGAAATCTTAATACTTCATTTTTTGATCCTGCAGGAGGTGGAGATCCTATTTTATATCAACATTTA

LOWAM086_Turkmenistan_3
ACATTATATTTTATTTTTGGTATTTGAGCAGGTATAGTAGGAACTTCCTTAAGATTATTAATTCGTACTGAATTAGGTAATCCTGGATCTTTAATTG
GAGATGATCAAATTTATAATACTATTGTAACAGCTCATGCTTTTATTATAATTTTTTTTATAGTTATACCAATTATAATTGGAGGATTTGGAAATTG
ATTAATTCCTTTAATATTAGGAGCCCCAGATATAGCTTTCCCCCGAATAAATAATATAAGATTTTGATTATTACCCCCCTCATTAACTTTATTAATT
TCTAGAAGAATTGTAGAAAATGGAGCAGGAACTGGATGAACAGTTTATCCCCCTTTATCATCTAATATTGCCCATAGAGGTAGTTCAGTTGATTTAG
CTATCTTTTCTTTACATTTAGCAGGAATTTCATCAATTTTAGGAGCTATTAATTTTATTACAACTATTATTAATATACGAATTAATCATATATCATT
TGATCAAATACCTCTTTTTGTATGAGCAGTAGGAATTACTGCTTTACTTTTATTACTATCTTTACCTGTATTAGCTGGTGCTATTACTATATTATTA
ACAGATCGAAATCTTAATACCTCATTTTTTGATCCAGCAGGAGGTGGAGATCCTATTTTATATCAACATTTA

LOWAM087_Uzbekistan_Sarchashma
ACACTATATTTTATTTTTGGTATTTGAGCAGGTATAGTAGGAACCTCTTTAAGATTATTAATTCGTACTGAATTAGGTAATCCTGGATCTTTAATTG
GAGATGATCAAATTTATAATACTATTGTAACAGCTCATGCTTTTATTATAATTTTTTTCATAGTTATACCAATTATAATTGGAGGATTTGGAAATTG
ATTAATTCCTTTAATATTAGGAGCCCCAGATATAGCTTTTCCTCGAATAAATAATATAAGATTTTGATTACTACCCCCTTCATTAACTTTATTAATT
TCCAGAAGAATTGTAGAAAATGGAGCAGGAACAGGATGAACAGTTTATCCCCCTTTATCATCTAATATCGCTCATAGAGGTAGTTCAGTTGATTTAG
CTATTTTTTCTTTACATTTAGCAGGAATTTCATCAATTTTAGGAGCTATTAATTTCATTACAACTATTATTAATATACGAATTAACCATATATCATT
TGATCAAATACCCCTCTTTGTATGAGCAGTAGGAATTACTGCTTTACTTTTATTATTATCTTTACCTGTATTAGCTGGTGCTATTACTATATTATTA
ACAGATCGAAATCTTAATACTTCATTTTTTGATCCTGCAGGAGGTGGAGATCCTATTTA??ATCAACATTTA

LOWAM095_Kyrgyzstan_Sosnovka
ACACTATATTTTATTTTTGGTATTTGAGCAGGTATAGTAGGAACCTCTTTAAGATTATTAATTCGTACTGAATTAGGTAATCCTGGATCTTTAATTG
GAGATGATCAAATTTATAATACTATTGTAACAGCTCATGCTTTTATTATAATTTTTTTTATAGTTATACCAATTATAATTGGAGGATTTGGAAATTG
ATTAATTCCTTTAATATTAGGGGCTCCAGATATAGCTTTTCCTCGAATAAATAATATAAGATTTTGATTATTACCCCCTTCATTAACTTTATTAATT
TCTAGAAGAATTGTAGAAAATGGAGCAGGAACAGGATGAACAGTTTATCCCCCTTTATCATCTAATATCGCTCATAGAGGTAGTTCAGTTGATTTAG
CTATTTTTTCTTTACATTTAGCGGGAATTTCATCAATCTTAGGAGCTATTAATTTTATTACAACTATTATTAATATACGAATTAATCATATATCATT
TGATCAAATACCTCTCTTTGTATGAGCAGTAGGAATTACTGCTTTACTTTTATTACTATCTTTACCTGTATTAGCTGGTGCTATTACTATATTATTA
ACAGATCGAAATCTTAATACTTCATTTTTTGATCCTGCAGGAGGTGGAGATCCTATTTTATATCAACATTTA

LOWAM096_Gissar_5
ACACTATATTTTATTTTTGGTATTTGAGCAGGTATAGTAGGAACCTCTTTAAGATTATTAATTCGTACTGAATTAGGTAATCCTGGATCTTTAATTG
GAGATGATCAAATTTATAATACTATTGTAACAGCTCATGCTTTTATTATAATTTTTTTCATAGTTATACCAATTATAATTGGAGGATTTGGAAATTG
ATTAATTCCTTTAATATTAGGAGCCCCAGATATAGCTTTTCCTCGAATAAATAATATAAGATTTTGATTACTACCCCCTTCATTAACTTTATTAATT
TCCAGAAGAATTGTAGAAAATGGAGCAGGAACAGGATGAACAGTTTATCCCCCTTTATCGTCTAATATCGCTCATAGAGGTAGTTCAGTTGATTTAG
CTATTTTTTCTTTACATTTAGCAGGAATTTCATCAATTTTAGGAGCTATTAATTTCATTACAACTATTATTAATATACGAATTAACCATATATCATT
TGATCAAATACCCCTCTTTGTATGAGCAGTAGGAATTACTGCTTTACTTTTATTATTATCTTTACCTGTATTAGCTGGTGCTATTACTATATTATTA
ACAGATCGAAATCTTAATACTTCATTTTTTGATCCTGCAGGAGGTGGAGATCCTATTTTATATCAACATTTA

LOWAM098_Tajikistan_Khondiza1
ACACTATATTTTATTTTTGGTATTTGAGCAGGTATAATAGGAACCTCTTTAAGATTATTAATTCGTACTGAATTAGGTAATCCTGGATCTTTAATTG
GAGATGATCAAATTTATAATACTATTGTAACAGCTCATGCTTTTATTATAATTTTTTTCATAGTTATACCAATTATAATTGGAGGATTTGGAAATTG
ATTAATTCCTTTAATATTAGGAGCCCCAGATATAGCTTTTCCTCGAATAAATAATATAAGATTTTGATTACTACCCCCTTCATTAACTTTATTAATT
TCCAGAAGAATTGTAGAAAATGGAGCAGGAACAGGATGAACAGTTTATCCCCCTTTATCGTCTAATATCGCTCATAGAGGTAGTTCAGTTGATTTAG
CTATTTTTTCTTTACATTTAGCAGGAATTTCATCAATTTTAGGAGCTATTAATTTCATTACAACTATTATTAATATACGAATTAACCATATATCATT
TGATCAAATACCCCTCTTTGTATGAGCAGTAGGAATTACTGCTTTACTTTTATTATTATCTTTACCTGTATTAGCTGGTGCTATTACTATATTATTA
ACAGATCGAAATCTTAATACTTCATTTTTTGATCCTGCAGGAGGTGGAGATCCTATTTTATATCAACATTTA

LOWAM099_Uzbekistan_Kamchik_2
ACACTATATTTTATTTTTGGTATTTGAGCAGGTATAGTAGGAACCTCTTTAAGATTATTAATTCGTACTGAATTAGGTAATCCTGGATCTTTAATTG
GAGATGATCAAATTTATAATACTATTGTAACAGCTCATGCTTTTATTATAATTTTTTTTATAGTTATACCAATTATAATTGGAGGATTTGGAAATTG

ATTAATTCCTTTAATATTAGGGGCCCCAGATATAGCTTTTCCTCGAATAAATAATATAAGATTTTGATTATTACCCCCTTCATTAACTTTATTAATT
TCTAGAAGAATTGTAGAAAATGGAGCAGGAACAGGATGAACAGTTTATCCCCCTTTATCATCTAATATCGCTCATAGAGGTAGTTCAGTTGATTTAG
CTATTTTTTCTTTACATTTAGCAGGAATTTCATCAATTTTAGGAGCTATTAATTTTATTACAACTATTATTAATATACGAATTAATCATATATCATT
TGATCAAATACCTCTCTTTGTATGAGCAGTAGGAATTACTGCTTTACTTTTATTACTATCTTTACCTGTATTAGCTGGTGCTATTACTATATTATTA
ACAGATCGAAATCTTAATACTTCATTTTTTGATCCTGCAGGAGGTGGAGATCCTATTTTATATCAACATTTA

LOWAM100_Kyrgyzstan_Konduk
ACACTATATTTTATTTTTGGTATTTGAGCAGGTATAGTAGGAACCTCTTTAAGATTATTAATTCGTACTGAATTAGGTAATCCTGGATCTTTAATTG
GAGATGATCAAATTTATAATACTATTGTAACAGCTCATGCTTTTATTATAATTTTTTTTATAGTTATACCAATTATAATTGGAGGATTTGGAAATTG
ATTAATTCCTTTAATATTAGGGGCCCCAGATATAGCTTTTCCTCGAATAAATAATATAAGATTTTGATTATTACCCCCTTCATTAACTTTATTAATT
TCTAGAAGAATTGTAGAAAATGGAGCAGGAACAGGATGAACAGTTTATCCCCCTTTATCATCTAATATCGCTCATAGAGGTAGTTCAGTTGATTTAG
CTATTTTTTCTTTACATTTAGCGGGAATTTCATCAATCTTAGGAGCTATTAATTTTATTACAACTATTATTAATATACGAATTAATCATATATCATT
TGATCAAATACCTCTCTTTGTATGAGCAGTAGGAATTACTGCTTTACTTTTATTACTATCTTTACCTGTATTAGCTGGTGCTATTACTATATTATTA
ACAGATCGAAATCTTAATACTTCATTTTTTGATCCTGCAGGAGGTGGAGATCCTATTTTATATCAACATTTA

LOWAM115_Azerbaijan_Nyus_Nyus
ACACTATATTTTATTTTCGGTATTTGAGCAGGTATAGTAGGAACCTCTTTAAGATTATTAATTCGTACTGAATTAGGTAATCCCGGATCTTTAATTG
GAGATGATCAAATTTATAACACTATTGTAACAGCTCATGCTTTTATTATAATTTTTTTTATAGTTATACCAATTATAATTGGAGGATTTGGAAATTG
ATTAATTCCTTTAATATTAGGAGCTCCAGATATAGCTTTTCCTCGAATAAATAATATAAGATTTTGATTACTCCCCCCCTCATTAACTTTATTAATT
TCTAGAAGAATTGTAGAAAATGGAGCAGGAACAGGATGAACAATTTATCCCCCTTTATCATCTAATATTGCTCATAGAGGTAGTTCAGTTGATTTAG
CTATTTTTTCTTTACATTTAGCAGGAATTTCATCAATCTTAGGAGCTATTAATTTTATTACAACTATTATTAACATACGAATTAATCATATATCATT
TGATCAAATACCCCTTTTTGTATGAGCAGTAGGAATTACTGCATTACTTTTATTATTATCTTTACCTGTATTAGCTGGTGCTATTACTATATTATTA
ACAGATCGAAATCTTAATACTTCATTTTTTGATCCTGCAGGAGGTGGAGATCCTATTTTATATCAACACTTA

LOWAM116_Iran_Dizin_2
ACACTATATTTTATTTTCGGTATTTGAGCAGGTATAGTAGGAACCTCTTTAAGATTATTAATTCGTACTGAATTAGGTAATCCTGGATCTTTAATTG
GAGATGATCAAATTTATAACACTATTGTAACAGCTCATGCTTTTATTATAATTTTTTTTATAGTTATACCAATTATAATTGGAGGATTTGGAAATTG
ATTAATTCCTTTAATATTAGGAGCTCCAGATATAGCTTTTCCTCGAATAAATAATATAAGATTTTGATTACTCCCCCCCTCATTAACTTTATTAATT
TCTAGAAGAATTGTAGAAAATGGAGCAGGAACAGGATGAACAATTTATCCCCCTTTATCATCTAATATTGCTCATAGAGGTAGTTCAGTTGATTTAG
CTATTTTTTCTTTACATTTAGCAGGAATTTCATCAATCTTAGGAGCTATTAATTTTATTACAACTATTATTAACATACGAATTAATCATATATCATT
TGATCAAATACCCCTTTTTGTATGAGCAGTAGGAATTACTGCATTACTTTTATTATTATCTTTACCTGTATTAGCTGGTGCTATTACTATATTATTA
ACAGATCGAAATCTTAATACTTCATTTTTTGATCCTGCAGGAGGTGGAGATCCTATTTTATATCAACACTTA

LOWAM122_Russia_Saratow_1
ACACTATATTTTATTTTTGGTATTTGAGCAGGTATAGTAGGAACCTCTTTAAGATTATTAATTCGTACTGAATTAGGTAATCCTGGATCTTTAATTG
GAGATGATCAAATTTATAACACTATTGTAACAGCTCATGCTTTTATTATAATTTTTTTTATAGTTATACCAATTATAATTGGAGGATTTGGAAATTG
ATTAATTCCTTTAATATTAGGAGCCCCAGATATAGCTTTTCCTCGAATAAATAATATAAGATTTTGATTACTACCCCCCTCATTAACTTTATTAATT
TCTAGAAGAATTGTAGAAAATGGAGCAGGAACAGGATGAACAGTTTATCCCCCTTTATCATCTAATATCGCTCATAGAGGTAGTTCAGTTGATTTAG
CTATTTTTTCTTTACATTTAGCAGGAATTTCATCAATCTTAGGAGCTATTAATTTTATTACAACTATTATTAATATACGAATTAACCATATATCATT
TGATCAAATACCTCTCTTTGTATGAGCAGTAGGAATTACTGCTTTACTTTTATTATTATCTTTACCTGTATTAGCTGGTGCTATTACTATATTATTA
ACAGATCGAAATCTTAATACTTCATTTTTTGATCCTGCAGGAGGTGGGGATCCTATTTTATATCAACATTTA

LOWAM136_Uzbekistan_Tamshush_1
ACACTATATTTTATTTTTGGTATTTGAGCAGGTATAGTAGGAACCTCTTTAAGATTATTAATTCGTACTGAATTAGGTAATCCTGGATCTTTAATTG
GAGATGATCAAATTTATAATACTATTGTAACAGCTCATGCTTTTATTATAATTTTTTTCATAGTTATACCAATTATAATTGGAGGATTTGGAAATTG
ATTAATTCCTTTAATATTAGGAGCCCCAGATATAGCTTTTCCTCGAATAAATAATATAAGATTTTGATTACTACCCCCTTCATTAACTTTATTAATT
TCCAGAAGAATTGTAGAAAATGGAGCAGGAACAGGATGAACAGTTTATCCCCCTTTATCATCTAATATCGCTCATAGAGGTAGTTCAGTTGATTTAG
CTATTTTTTCTTTACATTTAGCAGGAATTTCATCAATTTTAGGAGCTATTAATTTCATTACAACTATTATTAATATACGAATTAACCATATATCATT
TGATCAAATACCCCTCTTTGTATGAGCAATAGGAATTACTGCTTTACTTTTATTATTATCTTTACCTGTATTAGCTGGTGCTATTACTATATTATTA
ACAGATCGAAATCTTAATACTTCATTTTTTGATCCTGCAGGAGGTGGAGATCCTATTTTATATCAACATTTA

LOWAM149_Kyrgyzstan_Songkel_1
ACACTATATTTTATTTTTGGTATTTGAGCAGGTATAGTAGGAACCTCTTTAAGATTATTAATTCGTACTGAATTAGGTAATCCTGGATCTTTAATTG
GAGATGATCAAATTTATAATACTATTGTAACAGCTCATGCTTTTATTATAATTTTTTTTATAGTTATACCAATTATAATTGGAGGATTTGGAAATTG
ATTAATTCCTTTAATATTAGGGGCCCCAGATATAGCTTTTCCTCGAATAAATAATATAAGATTTTGATTATTACCCCCCTCATTAACTTTATTAATT
TCTAGAAGAATTGTAGAAAATGGAGCAGGAACAGGATGAACAGTTTATCCCCCTTTATCATCTAATATTGCTCATAGAGGTAGTTCAGTTGATTTAG
CTATTTTTTCTTTACATTTAGCGGGAATTTCATCAATCTTAGGAGCTATTAATTTTATTACAACTATTATTAATATACGAATTAATCATATATCATT
TGATCAAATACCTCTCTTTGTATGAGCAGTAGGAATTACTGCTTTACTTTTATTACTATCTTTACCTGTATTAGCTGGTGCTATTACTATATTATTA
ACAGATCGAAATCTTAATACTTCATTTTTTGATCCTGCAGGAGGTGGAGATCCTATTTTATATCAACATTTA

LOWAM198_Turkey_Aladaglar_2
ACACTATATTTTATTTTTGGTATTTGAGCAGGTATAGTAGGAACCTCTTTAAGATTATTAATTCGTACTGAATTAGGTAATCCTGGATCTTTAATTA
GAGATGATCAAATTTATAATACTATTGTAACAGCTCATGCTTTTATTATAATTTTTTTTATAGTTATACCAATTATAATTGGAGGATTTGGAAATTG
ATTAATTCCTTTAATATTAGGAGCTCCAGATATAGCTTTTCCTCGAATAAATAATATAAGATTTTGATTACTTCCCCCTTCATTAACTTTATTAATT
TCTAGAAGAATTGTAGAAAATGGAGCAGGAACAGGATGAACAGTTTATCCCCCTTTATCATCTAATATTGCTCATAGAGGTAGTTCAGTTGATTTAG
CTATTTTTTCTTTACATTTAGCAGGAATTTCATCAATCTTAGGAGCTATTAATTTTATTACAACTATTATTAATATA--------------------
----

LOWAM213_Tajikistan_Tandukul_1
ACACTATATTTTATTTTTGGTATTTGAGCAGGTATAGTAGGAACCTCTTTAAGATTATTAATTCGTACTGAATTAGGTAATCCTGGATCTTTAATTG
GAGATGATCAAATTTATAATACTATTGTAACAGCTCATGCTTTTATTATAATTTTTTTCATAGTTATACCAATTATAATTGGAGGATTTGGAAATTG
ATTAATTCCTTTAATATTAGGAGCCCCAGATATAGCTTTTTCTCGAATAAATAATATAAGATTTTGATTACTACCCCCTTCATTAACTTTATTAATT
TCTAGAAGAATTGTAGAAAATGGAGCAGGAACAGGATGAACAGTTTATCCCCCTTTATTATCTAATATCGCTCATAGAGGTAGTTCAGTTGATTTAG
CTATTTTTTCTTTACATTTAGCAGGAATTTCATCAATTTTAGGAGCTATTAATTTCATTACAACTATTATTAATATACGAATTAACCATATATCATT
TGATCAAATACCCCTCTTTGTATGAGCAGTAGGAATTACTGCGTTACTTTTATTATTATCTTTACCTGTATTAGCTGGTGCTATTACTATATTATTA
ACAGATCGAAATCTTAATACTTCATTTTTTGATCCTGCAGGAGGTGGAGATCCTATTTTATATCAACATTTA

Parnassius_clodius
ACATTATATTTTATTTTTGGTATTTGAGCAGGTATAGTAGGAACCTCTTTAAGATTATTAATTCGTACTGAATTAGGTAATCCTGGATCTTTAATTG

GAGATGATCAAATTTATAATACTATTGTAACAGCTCATGCTTTTATCATAATTTTTTTCATAGTTATACCAATTATAATTGGAGGATTTGGAAATTG
ATTAATTCCATTAATATTAGGAGCTCCAGATATAGCTTTTCCTCGAATAAATAATATAAGATTTTGATTATTACCCCCTTCATTAACTTTATTAATT
TCTAGAAGAATTGTACAAAATGGAGCAGGAACTGGATGAACAGTTTATCCCCCTTTATCATCTAATATTGCTCATAGAGGAAGATCAGTTGATTTAG
CTATTTTTTCTTTACATTTAGCTGGAATTTCATCTATCTTAGGAGCTATTAATTTTATTACAACTATTATTAATATACGAATTAATCATATATCATT
TGATCAAATACCCCTTTTTGTATGAGCAGTAGGAATTACCGCTTTACTTCTATTATTATCTTTACCTGTTTTAGCAGGTGCTATTACCATATTATTA
ACAGATCGAAATCTTAATACTTCATTTTTTGATCCAGCAGGAGGTGGAGATCCTATTTTATATCAACACTTA

Parnassius_eversmanni_2005_LOWA_108 ---------------------
ATTTGAGCAGGTATAGTAGGAACCTCTTTAAGATTATTAATTCGTTCTGAATTAGGTAATCCTGGATCTTTAATTGGAGATGATCAAATTTATAATA
CTATTGTAACAGCTCATGCTTTTATTATAATTTTTTTATAGTTATACCAATTATAATTGGAGGATTTGGAAATTGATTAATTCCATTAATATTAGG
AGCTCCAGATATAGCTTTTCCTCGAATAAATAATATAAGATTTTGATTATTACCCCCCTCATTAACCTTACTAATTTCTAGAAGAATTGTAGAAAAT
GGAGCAGGAACTGGATGAACGGTTTATCCCCCTTTATCATCTAATATTGCTCATAGAGGAAGATCAGTTGATTTAGCTATCTTTTCTTTACATTTAG
CTGGAATTTCATCTATCTTAGGAGCTATTAATTTTATTACAACTATTATTAATATACGAATTAATCATATATCATTTGATCAAATACCCCTTTTTGT
ATGAGCAGTAGGAATTACTGCTTTACTTTTATTATTATCTTTACCTGTTTTAGCAGGTGCTATTACCATATTATTAACAGATCGAAATCTTAATACT
TCATTTTTTGACCCAGCAGGAGGTGGAGATCCTATTTTATATCAACATTTA

Parnassius_eversmanni_2005_LOWA_107 ---------------------
ATTTGAGCAGGTATAGTAGGAACCTCTTTAAGATTATTAATTCGTTCTGAATTAGGTAATCCTGGATCTTTAATTGGAGATGATCAAATTTATAATA
CTATTGTAACAGCTCATGCTTTTATTATAATTTTTTTATAGTTATACCAATTATAATTGGAGGATTTGGAAATTGATTAATTCCATTAATATTAGG
AGCTCCAGATATAGCTTTTCCTCGAATAAATAATATAAGATTTTGATTATTACCCCCCTCATTAACCTTACTAATTTCTAGAAGAATTGTAGAAAAT
GGAGCAGGAACTGGATGAACGGTTTATCCCCCTTTATCATCTAATATTGCTCATAGAGGAAGATCAGTTGATTTAGCTATCTTTTCTTTACATTTAG
CTGGAATTTCATCTATCTTAGGAGCTATTAATTTTATTACAACTATTATTAATATACGAATTAATCATATATCATTTGATCAAATACCCCTTTTTGT
ATGAGCAGTAGGAATTACTGCTTTACTTTTATTATTATCTTTACCTGTTTTAGCAGGTGCTATTACCATATTATTAACAGATCGAAATCTTAATACT
TCATTTTTTGACCCAGCAGGAGGTGGAGATCCTATTTTATATCAACATTTA

Parnassius_stubbendorfii_2005_LOWA_815
ACATTATATTTTATTTTTGGTATTTGAGCAGGTATAGTAGGAACTTCTTTAAGATTATTAATTCGTACTGAATTAGGTAATCCTGGATCTTTAATTG
GAGATGATCAAATTTATAATACTATTGTAACAGCTCATGCTTTTATTATAATTTTTTTATAGTTATACCAATTATAATTGGAGGATTTGGAAATTG
ATTAATTCCTTTAATATTAGGAGCCCCAGATATAGCTTTTCCTCGAATAAATAATATAAGATTTTGATTATTACCCCCTTCATTAACTTTACTAATT
TCCAGAAGAATTGTAGAAAATGGGGCAGGAACTGGATGAACAGTCTACCCTCCTTTATCATCTAATATTGCTCACGGAGGAAGATCTGTTGATTTAG
CTATTTTTTCCTTACATTTAGCGGGAATTTCATCTATTTTAGGAGCCATTAATTTTATTACAACTATTATTAATATACGAATTAATCATATATCATT
TGATCAAATACCTCTTTTTGTATGAGCAGTAGGAATTACTGCTTTACTTTTATTATTATCTCTACCTGTTTTAGCAGGTGCTATTACTATATTATTA
ACAGATCGAAATCTTAATACTTCATTTTTTGACCCAGCAGGAGGTGGAGATCCTATTTTATATCAACACTTA

Parnassius_stubbendorfii_2005_LOWA_154
ACATTATATTTTATTTTTGGTATTTGAGCAGGTATAGTAGGAACTTCTTTAAGATTATTAATTCGTACTGAATTAGGTAATCCTGGATCTTTAATTG
GAGATGATCAAATTTATAATACTATTGTAACAGCTCATGCTTTTATTATAATTTTTTTATAGTTATACCAATTATAATTGGAGGATTTGGAAATTG
ATTAATTCCTTTAATATTAGGAGCCCCAGATATAGCTTTTCCTCGAATAAATAATATAAGATTTTGATTATTACCCCCTTCATTAACTTTACTAATT
TCCAGAAGAATTGTAGAAAATGGAGCAGGAACTGGATGAACAGTCTACCCTCCTTTATCATCTAATATTGCTCACGGAGGAAGATCTGTTGATTTAG
CTATTTTTTCCTTACATTTAGCGGGAATTTCATCTATTTTAGGAGCCATTAATTTTATTACAACTATTATTAATATACGAATTAATCATATATCATT
TGATCAAATACCTCTTTTTGTATGAGCAGTAGGAATTACTGCTTTACTTTTATTATTATCTCTACCTGTTTTAGCAGGTGCTATTACTATATTATTA
ACAGATCGAAATCTTAATACTTCATTTTTTGACCCAGCAGGAGGTGGAGATCCTATTTTATATCAACACTTA

Parnassius_stubbendorfii_2005_LOWA_153 -
CATTATATTTTATTTTTGGTATTTGAGCAGGTATAGTAGGAACTTCTTTAAGATTATTAATTCGTACTGAATTAGGTAATCCTGGATCTTTAATTGG
AGATGATCAAATTTATAATACTATTGTAACAGCTCATGCTTTTATTATAATTTTTTTATAGTTATACCAATTATAATTGGAGGATTTGGAAATTGA
TTAATTCCTTTAATATTAGGAGCCCCAGATATAGCTTTTCCTCGAATAAATAATATAAGATTTTGATTATTACCCCCTTCATTAACTTTACTAATTT
CCAGAAGAATTGTAGAAAATGGAGCAGGAACTGGATGAACAGTCTACCCTCCTTTATCATCTAATATTGCTCACGGAGGAAGATCTGTTGATTTAGC
TATTTTTTCCTTACATTTAGCGGGAATTTCATCTATTTTAGGAGCCATTAATTTTATTACAACTATTATTAATATACGAATTAATCATATATCATTT
GATCAAATACCTCTTTTTGTATGAGCAGTAGGAATTACTGCTTTACTTTTATTATTATCTCTACCTGTTTTAGCAGGTGCTATTACTATATTATTAA
CAGATCGAAATCTTAATACTTCATTTTTTGACCCAGCAGGAGGTGGAGATCCTATTTTATATCAACACTTA

Parnassius_nordmanni
ACATTATATTTTATTTTTGGTATTTGAGCAGGTATAGTAGGAACTTCTTTAAGATTATTAATTCGTACTGAATTAGGTAATCCTGGATCTCTAATTG
GAGATGATCAAATTTACAATACTATCGTAACAGCTCATGCTTTTATTATAATTTTTTTCATAGTTATACCAATTATAATTGGAGGATTTGGAAATTG
ATTAATTCCATTAATATTAGGAGCTCCAGATATAGCTTTCCCCCGAATAAATAATATAAGATTTTGATTATTACCCCCCTCATTAACTCTATTAATT
TCTAGAAGAATTGTAGAAAATGGGGCAGGAACTGGATGAACAGTTTACCCCCCTTTATCATCTAATATTGCTCATAGAGGAAGATCAGTTGACTTAG
CTATTTTTTCTTTACATTTGGCTGGGATTTCTTCTATTTTAGGAGCTATTAATTTTATCACAACTATTGTTAATATACGAATTAACTATATATCATT
TGATCAAATACCTCTTTTCGTATGAGCAGTAGGAATTACTGCTTTACTTTTATTATTATCTTTACCTGTTTTAGCAGGTGCTATTACTATATTATTA
ACAGATCGAAATCTTAATACTTCATTTTTTGATCCAGCAGGAGG?GGAGACCCTATTCTATATCAA------

Parnassius_eversmanni
ACATTATATTTTATTTTTGGTATTTGAGCAGGTATAGTAGGAACCTCTTTAAGATTATTAATTCGTTCTGAATTAGGTAATCCTGGATCTTTAATTG
GAGATGATCAAATTTATAATACTATTGTAACAGCTCATGCTTTTATTATAATTTTTTTATAGTTATACCAATTATAATTGGAGGATTTGGAAATTG
ATTAATTCCATTAATATTAGGAGCTCCAGATATAGCTTTTCCTCGAATAAATAATATAAGATTTTGATTATTACCCCCCTCATTAACCTTACTAATT
TCTAGAAGAATTGTAGAAAATGGAGCAGGAACTGGATGAACGGTTTATCCCCCTTTATCATCTAATATTGCTCATAGAGGAAGATCAGTTGATTTAG
CTATCTTTTCTTTACATTTAGCTGGAATTTCATCTATCTTAGGAGCTATTAATTTTATTACAACTATTATTAATATACGAATTAATCATATATCATT
TGATCAAATACCCCTTTTTGTATGAGCAGTAGGAATTACTGCTTTACTTTTATTATTATCTTTACCTGTTTTAGCAGGTGCTATTACCATATTATTA
ACAGATCGAAATCTTAATACTTCATTTTTTGACCCAGCAGGAGGTGGAGATCCTATTTTATATCAA------

Parnassius_eversmanni_felderi
ACATTATATTTTATTTTTGGTATTTGAGCAGGTATAGTAGGAACCTCTTTAAGATTATTAATTCGTTCTGAATTAGGTAATCCTGGATCTTTAATTG
GAGATGATCAAATTTATAATACTATTGTAACAGCTCATGCTTTTATTATAATTTTTTTATAGTTATACCAATTATAATTGGAGGATTTGGAAATTG
ATTAATTCCATTAATATTAGGAGCTCCAGATATAGCTTTTCCTCGAATAAATAATATAAGATTTTGATTATTACCCCCCTCATTAACCTTACTAATT
TCTAGAAGAATTGTAGAAAATGGAGCAGGAACTGGATGAACGGTTTATCCCCCTTTATCATCTAATATTGCTCATAGAGGAAGATCAGTTGATTTAG
CTATCTTTTCTTTACA?TTAGCTGGAATTTCATCTATCTTAGGAGCTATTAATTTTATTACAACTATTATTAATATACGAATTAATCATATATCATT
TGATCAAATACCCCTTTTTGTATGAGCAGTAGGAATTACTGCTTTACTTTTATTATTATCTTTACCTGTTTTAGCAGGTGCTATTACCATATTATTA
ACAGATCGAAATCTTAATACTTCATTTTTTGACCCAGCAGGAGGTGGAGATCCTATTTTATATCAA------

Parnassius_ariadne_ariadne
ACATTATATTTATTTTTGGTATTTGAGCAGGTATAGTAGGAACCTCTTTAAGATTATTAATTCGTACTGAATTAGGTAATCCTGGATCTTTAATTG
GAGATGATCAAATTTATAATACTATCGTAACAGCTCATGCTTTTATTATAATTTTTTTTATAGTTATACCAATTATAATTGGAGGATTTGGAAATTG
ATTAATTCCTTTAATATTAGGAGCTCCAGATATAGCCTTTCCTCGAATAAATAATATAAGATTTTGATTACTACCCCCCTCATTAACTTTATTAATC
TCTAGAAGAATTGTAGAAAATGGAGCAGGAACTGGATGAACAGTTTATCCCCCTTTATCATCTAATATTGCTCATAGAGGAAGTTCAGTTGATTTAG
CCATTTTCTCTTTACATTTAGCAGGAATTTCATCAATTTTAGGAGCTATTAATTTTATCACAACTATTATTAATATACGAATTAATCATATATCATT
TGATCAAATACCCCTTTTTGTTTGAGCAGTAGGAATTACTGCTTTACTATTATTATTATCTTTACCTGTATTAGCTGGTGCTATTACTATATTATTA
ACAGATCGAAATCTTAATACTTCATTTTTTGATCCAGCAGGA????GAGATCCTATTTTATATCAA------

Parnassius_glacialis_mikado
ACATTATATTTATTTTTGGTATTTGAGCAGGTATAGTAGGAACCTCCTTAAGATTATTAATTCGTACTGAATTAGGTAATCCTGGATCTTTAATTG
GAGATGATCAAATTTATAATACTATTGTAACAGCTCATGCTTTTATTATAATTTTTTTTATAGTTATACCAATTATAATTGGAGGATTTGGAAATTG
ATTAATCCCTTTAATATTAGGAGCTCCAGATATAGCTTTCCCCCGAATAAATAATATAAGATTTTGATTATTACCCCCCTCATTAACTTTACTAATT
TCCAGAAGAATTGTAGAAAATGGAGCAGGAACAGGATGAACAGTTTATCCCCCATTATCCTCTAATATTGCCCACAGAGGAAGATCTGTTGATTTAG
CTATTTTTTCTTTACATTTAGCAGGAATTTCATCTATTCTAGGAGCTATTAATTTTATTACAACTATTATTAATATACGAATTAATCATATATCATT
TGATCAAATACCTCTCTTTGTTTGAGCAGTAGGAATTACTGCTTTACTTTTATTATTATCCCTACCTGTTTTAGCAGGTGCTATTACTATATTATTA
ACAGATCGAAATCTTAATACTTCTTTTTTTGACCCAGCAGGAGGTGGAGATCCAATTTTATATCAA------

Parnassius_stubbendorfii_hoenei
ACATTATATTTATTTTTGGTATTTGAGCAGGAATAGTAGGAACCTCCCTAAGATTATTAATTCGTACTGAATTAGGTAATCCCGGATCTTTAATTG
GAGATGATCAAATTTACAATACTATTGTAACAGCTCATGCTTTCATTATAATTTTTTTTATAGTTATACCAATTATAATTGGAGGATTTGGAAATTG
ACTGATTCCTTTAATATTAGGAGCCCCAGATATAGCTTTCCCCCGAATAAATAATATAAGATTTTGATTACTACCCCCCTCATTAACTTTATTAATT
TCTAGAAGAATTGTAGAAAATGGAGCAGGAACTGGATGAACAGTCTATCCCCCTTTATCATCTAATATTGCCCATAGAGGAAGATCCGTTGATTTAG
CTATTTTTTCCTTACATTTAGCAGGAATTTCATCTATTTTAGGAGCCATTAATTTTATTACAACTATTATTAATATACGAATTAATCATATATCATT
TGATCAAATACCTCTTTTTGTATGAGCAGTAGGAATTACTGCTTTACTTTTATTATTATCTTTACCTGTTTTAGCAGGTGCTATTACTATATTATTA
ACAGATCGAAATCTTAATACTTCCTTTTTTGACCCAGCAGGAGGTGGAGATCCTATTTTATACCAA------

Parnassius_stubbendorfii_hoenei_AC20_16 ------------------------
TGAGCAGGAATAGTAGGAACCTCCCTAAGATTATTAATTCGTACTGAATTAGGTAATCCCGGATCTTTAATTGGAGATGATCAAATTTACAATACTA
TTGTAACAGCTCATGCTTTCATTATAATTTTTTTTATAGTTATACCAATTATAATTGGAGGATTTGGAAATTGACTGATTCCTTTAATATTAGGAGC
CCCAGATATAGCTTTCCCCCGAATAAATAATATAAGATTTTGATTACTACCCCCCTCATTAACTTTATTAATTTCTAGAAGAATTGTAGAAAATGGA
GCAGGAACTGGATGAACAGTCTATCCCCCTTTATCATCTAATATTGCCCATAGAGGAAGATCCGTTGATTTAGCTATTTTTTCCTTACATTTAGCAG
GAATTTCATCTATTTTAGGAGCCATTAATTTTATTACAACTATTATTAATATACGAATTAATCATATATCATTTGATCAAATACCTCTTTTTGTATG
AGCAGTAGGAATTACTGCTTTACTTTTATTATTATCTTTACCTGTTTTAGCAGGTGCTATTACTATATTATTAACAGATCGAAATCTTAATACTTCC
TTTTTTGACCCCGCAGGAGGTGGAGATCCTATTTTATACCAACATTTA

Parnassius_stubbendorfii_koreanus ------------------------
TGAGCAGGTATAGTAGGAACTTCTTTAAGATTATTAATTCGTACTGAATTAGGTAATCCTGGATCTTTAATTGGAGATGATCAAATTTATAATACTA
TTGTAACAGCTCATGCTTTTATTATAATTTTTTTTATAGTTATACCAATTATAATTGGAGGATTTGGAAATTGATTAATTCCTTTAATATTAGGAGC
CCCAGATATAGCTTTTCCTCGAATAAATAATATAAGATTTTGACTATTACCCCCCTCATTAACTTTACTAATTTCCAGAAGAATTGTAGAAAATGGA
GCAGGAACTGGATGAACAGTCTACCCTCCTTTATCATCTAATATTGCTCACGGAGGAAGATCTGTTGATTTAGCTATTTTTTCCTTACATTTAGCAG
GAATTTCATCTATTTTAGGAGCCATTAATTTTATTACAACTATTATTAATATACGAATTAATCATATATCATTTGATCAAATACCTCTTTTTGTATG
AGCAGTAGGAATTACTGCTTTACTTTTATTATTATCTCTACCTGTTTTAGCAGGTGCTATTACTATATTATTAACAGATCGAAATCTTAATACTTCA
TTTTTTGACCCAGCAGGAGGTGGAGATCCTATTTTATATCAACACTTA

Parnassius_nordmanni_AC20_5 ------------------------
TGAGCAGGTATAGTAGGAACTTCTTTAAGATTATTAATTCGTACTGAATTAGGTAATCCTGGATCTCTAATTGGAGATGATCAAATTTACAATACTA
TCGTAACAGCTCATGCTTTTATTATAATTTTTTTCATAGTTATACCAATTATAATTGGAGGATTTGGAAATTGATTAATTCCATTAATATTAGGAGC
TCCAGATATAGCTTTCCCCCGAATAAATAATATAAGATTTTGATTATTACCCCCCTCATTAACTCTATTAATTTCTAGAAGAATTGTAGAAAATGGG
GCAGGAACTGGATGAACAGTTTACCCCCCTTTATCATCTAATATTGCTCATAGAGGAAGATCAGTTGACTTAGCTATTTTTTCTTTACATTTGGCTG
GGATTTCTTCTATTTTAGGAGCTATTAATTTTATCACAACTATTGTTAATATACGAATTAACTATATATCATTTGATCAAATACCTCTTTTCGTATG
AGCAGTAGGAATTACTGCTTTACTTTTATTATTATCTTTACCTGTTTTAGCAGGTGCTATTACTATATTATTAACAGATCGAAATCTTAATACTTCA
TTTTTTGATCCAGCAGGAGGTGGAGACCCTATTCTATATCAACATTTA

Parnassius_eversmanni_felderi_AC23_68 ------------------------
TGAGCAGGTATAGTAGGAACCTCTTTAAGATTATTAATTCGTTCTGAATTAGGTAATCCTGGATCTTTAATTGGAGATGATCAAATTTATAATACTA
TTGTAACAGCTCATGCTTTTATTATAATTTTTTTTATAGTTATACCAATTATAATTGGAGGATTTGGAAATTGATTAATTCCATTAATATTAGGAGC
TCCAGATATAGCTTTTCCTCGAATAAATAATATAAGATTTTGATTATTACCCCCCTCATTAACCTTACTAATTTCTAGAAGAATTGTAGAAAATGGA
GCAGGAACTGGATGAACGGTTTATCCCCCTTTATCATCTAATATTGCTCATAGAGGAAGATCAGTTGATTTAGCTATCTTTTCTTTACATTTAGCTG
GAATTTCATCTATCTTAGGAGCTATTAATTTTATTACAACTATTATTAATATACGAATTAATCATATATCATTTGATCAAATACCCCTTTTTGTATG
AGCAGTAGGAATTACTGCTTTACTTTTATTATTATCTTTACCTGTTTTAGCAGGTGCTATTACCATATTATTAACAGATCGAAATCTTAATACTTCA
TTTTTTGACCCAGCAGGAGGTGGAGATCCTATTTTATTTCAACATTTA

Parnassius_eversmanni_eversmanni_AC1_14 ------------------------
TGATCAGGTATAGTAGGAACCTCTTTAAGATTATTAATTCGTTCTGAATTAGGTAATCCTGGATCTTTAATTGGAGATGATCAAATTTATAATACTA
TTGTAACAGCTCATGCTTTTATTATAATTTTTTTTATAGTTATACCAATTATAATTGGAGGATTTGGAAATTGATTAATTCCATTAATATTAGGAGC
TCCAGATATAGCTTTTCCTCGAATAAATAATATAAGATTTTGATTATTACCCCCCTCATTAACCTTACTAATTTCTAGAAGAATTGTAGAAAATGGA
GCAGGAACTGGATGAACGGTTTATCCCCCTTTATCATCTAATATTGCTCATAGAGGAAGATCAGTTGATTTAGCTATCTTTTCTTTACATTTAGCTG
GAATTTCATCTATCTTAGGAGCTATTAATTTTATTACAACTATTATTAATATACGAATTAATCATATATCATTTGATCAAATACCCCTTTTTGTATG
AGCAGTAGGAATTACTGCTTTACTTTTATTATTATCTTTACCTGTTTTAGCAGGTGCTATTACCATATTATTAACAGATCGAAATCTCAATACTTCA
TTTTTTGACCCAGCAGGAGGTGGAGATCCTATTTTATATCAACATTTA

Parnassius_clodius_AC4_5 ------------------------
TGAGCAGGAATATTAGGAACCTCTTTAAGATTATTAATTCGTACTGAATTAGGTAATCCTGGATCTTTAATTGGAGATGATCAAATTTATAATACTA
TTGTAACAGCTCATGCTTTTATCATAATTTTTTTCATAGTTATACCAATTATAATTGGAGGATTTGGAAATTGATTAATTCCATTAATATTAGGAGC
TCCAGATATAGCTTTTCCTCGAATAAATAATATAAGATTTTGATTATTACCCCCTTCATTAACTTTATTAATTTCTAGAAGAATTGTAGAAAATGGA
GCAGGAACTGGATGAACAGTTTATCCCCCTTTATCATCTAATATTGCTCATAGAGGAAGATCAGTTGATTTAGCTATTTTTTCTTTACATTTAGCTG
GAATTTCATCTATCTTAGGAGCTATTAATTTTATTACAACTATTATTAATATACGAATTAATCATATATCATTTGATCAAATACCCCTTTTTGTATG
AGCAGTAGGAATTACCGCTTTACTTCTATTATTATCTTTACCTGTTTTAGCAGGTGCTATTACCATATTATTAACAGATCGAAATCTTAATACTTCA
TTTTTTGATCCAGCAGGAGGTGGAGATCCTATTTTATATCAACACTTA

Parnassius_ariadne_AC4_14 ------------------------
TGAGCAGGTATAGTAGGAACCTCTTTAAGATTATTAATTCGTACTGAATTAGGTAATCCTGGATCTTTAATTGGAGATGATCAAATTTATAATACTA
TCGTAACAGCTCATGCTTTTATTATAATTTTTTTTATAGTTATACCAATTATAATTGGAGGATTTGGAAATTGATTAATTCCTTTAATATTAGGAGC
TCCAGATATAGCCTTTCCTCGAATAAATAATATAAGATTTTGATTACTACCCCCCTCATTAACTTTATTAATCTCTAGAAGAATTGTAGAAAATGGA
GCAGGAACTGGATGAACAGTTTATCCCCCTTTATCATCTAATATTGCTCATAGAGGAAGTTCAGTTGATTTAGCCATTTTCTCTTTACATTTAGCAG
GAATTTCATCAATTTTAGGAGCTATTAATTTTATCACAACTATTATTAATATACGAATTAATCATATATCATTTGATCAAATACCCCTTTTTGTTTG
AGCAGTAGGAATTACTGCTTTACTATTATTATTATCTTTACCTGTATTAGCTGGTGCTATTACTATATCATTAACAGATCGAAATCTTAATACTTCA
TTTTTTGATCCAGCAGGAGGTGGAGATCCTATTTTATATCAACACTTA

HQ004911_RV_07_C107_Romania
ACACTATATTTTATTTTTGGTATTTGAGCAGGTATAGTAGGAACCTCTTTAAGATTATTAATTCGTACTGAATTAGGTAATCCTGGATCTTTAATTG
GAGATGATCAAATTTATAATACTATTGTAACAGCTCATGCTTTTATTATAATTTTTTTTATAGTTATACCAATTATAATTGGAGGATTTGGAAATTG
ATTAATTCCTTTAATATTAGGAGCCCCAGATATAGCTTTTCCTCGAATAAATAATATAAGATTTTGATTACTACCCCCCTCATTAACTTTATTAATT
TCTAGAAGAATTGTAGAAAATGGAGCAGGAACAGGATGAACAGTTTATCCCCCTTTATCATCTAATATCGCTCATAGAGGTAGTTCAGTTGATTTAG
CTATTTTTTCTTTACATTTAGCAGGAATTTCATCAATCTTAGGAGCTATTAATTTTATTACAACTATTATTAATATACGAATTAACCATATATCATT
TGATCAAATACCTCTCTTTGTATGAGCAGTAGGAATTACTGCTTTACTTTTATTATTATCTTTACCTGTATTAGCTGGTGCTATTACTATATTATTA
ACAGATCGAAATCTTAATACTTCATTTTTTGATCCTGCAGGAGGTGGAGATCCTATTTTATATCAACATTTA

HQ004908_RV_07_D060_Romania
ACACTATATTTTATTTTTGGTATTTGAGCAGGTATAGTAGGAACCTCTTTAAGATTATTAATTCGTACTGAATTAGGTAATCCTGGATCTTTAATTG
GAGATGATCAAATTTATAATACTATTGTAACAGCTCATGCTTTTATTATAATTTTTTTTATAGTTATACCAATTATAATTGGAGGATTTGGAAATTG
ATTAATTCCTTTAATATTAGGAGCCCCAGATATAGCTTTTCCTCGAATAAATAATATAAGATTTTGATTACTACCCCCCTCATTAACTTTATTAATT
TCTAGAAGAATTGTAGAAAATGGAGCAGGAACAGGATGAACAGTTTATCCCCCTTTATCATCTAATATCGCTCATAGAGGTAGTTCAGTTGATTTAG
CTATTTTTTCTTTACATTTAGCAGGAATTTCATCAATCTTAGGAGCTATTAATTTTATTACAACTATTATTAATATACGAATTAACCATATATCATT
TGATCAAATACCTCTCTTTGTATGAGCAGTAGGAATTACTGCTTTACTTTTATTATTATCTCTACCTGTATTAGCTGGTGCTATTACTATATTATTA
ACAGATCGAAATCTTAATACTTCATTTTTTGATCCTGCAGGAGGTGGAGATCCTATTTTATATCAACATTTA

HQ004907_RV_08_M274_Romania
ACACTATATTTTATTTTTGGTATTTGAGCAGGTATAGTAGGAACCTCTTTAAGATTATTAATTCGTACTGAATTAGGTAATCCTGGATCTTTAATTG
GAGATGATCAAATTTATAATACTATTGTAACAGCTCATGCTTTTATTATAATTTTTTTTATAGTTATACCAATTATAATTGGAGGATTTGGAAATTG
ATTAATTCCTTTAATATTAGGAGCCCCAGATATAGCTTTTCCTCGAATAAATAATATAAGATTTTGATTACTACCCCCCTCATTAACTTTATTAATT
TCTAGAAGAATTGTAGAAAATGGAGCAGGAACAGGATGAACAGTTTATCCCCCTTTATCATCTAATATCGCTCATAGAGGTAGTTCAGTTGATTTAG
CTATTTTTTCTTTACATTTAGCAGGAATTTCATCAATCTTAGGAGCTATTAATTTTATTACAACTATTATTAATATACGAATTAACCATATATCATT
TGATCAAATACCTCTATTTGTATGAGCAGTAGGAATTACTGCTTTACTTTTATTATTATCTTTACCTGTATTAGCTGGTGCTATTACTATATTATTA
ACAGATCGAAATCTTAATACTTCATTTTTTGATCCTGCAGGAGGTGGAGATCCTATTTTATATCAACATTTA

HQ004902_RV_08_M361_Romania
ACACTATATTTTATTTTTGGTATTTGAGCAGGTATAGTAGGAACCTCTTTAAGATTATTAATTCGTACTGAATTAGGTAATCCTGGATCTTTAATTG
GAGATGATCAAATTTATAATACTATTGTAACAGCTCATGCTTTTATTATAATTTTTTTTATAGTTATACCAATTATAATTGGAGGATTTGGAAATTG
ATTAATTCCCTTAATATTAGGAGCCCCAGATATAGCTTTTCCTCGAATAAATAATATAAGATTTTGATTACTACCCCCCTCATTAACTTTATTAATT
TCTAGAAGAATTGTAGAAAATGGAGCAGGAACAGGATGAACAGTTTATCCCCCTTTATCATCTAATATCGCTCATAGAGGTAGTTCAGTTGATTTAG
CTATTTTTTCTTTACATTTAGCAGGAATTTCATCAATCTTAGGAGCTATTAATTTTATTACAACTATTATTAATATACGAATTAACCATATATCATT
TGATCAAATACCTCTCTTTGTATGAGCAGTAGGAATTACTGCTTTACTTTTATTATTATCTTTACCTGTATTAGCTGGTGCTATTACTATATTATTA
ACAGATCGAAATCTTAATACTTCATTTTTTGATCCTGCAGGAGGTGGAGATCCTATTTTATATCAACATTTA

GU947642_Pmne_ITNEB01_Italy_Sicily --

CCCCTTCATTAACCTTATTAATTTCTAGAAGAATTGTAGAAAATGGAGCAGGAACTGGATGAACAGTTTACCCCCCTTTATCATCTAATATTGCTCA
TAGAGGAAGTTCAGTTGATTTAGCTATTTTTTCCTTACATTTAGCGGGAATTTCATCAATCTTAGGAGCTATTAATTTTATTACAACTATTATTAAT
ATACGAATTAATCATATATCATTCGATCAAATACCTCTTTTTGTATGAGCAGTAGGAATTACTGCTTTACTTTTATTATTATCTTTACCTGTATTAG
CTGGTGCTATTACTATATTATTAACAGATCGAAATCTTAATACTTCATTTTTTGATCCCGCAGGAGGTGGTGATCCTATTTTATACCAACATTTA

EU093018_Ca995 --

TTTAATATTAGGAGCCCCAGATATAGCTTTTCCTCGAATAAATAATATAAGATTTTGATTACTGCCCCCCTCATTAACTTTATTAATTTCTAGAAGA
ATTGTAGAAAATGGAGCAGGAACAGGATGAACAGTTTATCCCCCTTTATCATCTAATATCGCTCATAGAGGTAGTTCAGTTGATTTAGCTATTTTTT
CTTTACATTTAGCAGGAATTTCATCAATCTTAGGAGCTATTAATTTTATTACAACTATTATTAATATACGAATTAACCATATATCATTTGATCAAAT
ACCTCTCTTTGTATGAGCAGTAGGAATTACTGCTTTACTTTTATTATTATCTTTACCTGTATTAGCTGGTGCTATTACTATATTATTAACAGATCGA
AATCTTAATACTTCATTTTTTGATCCTGCAGGAGGTGGAGATCCTATTTTATATCAACATTTA

EU093017_Ch892 --

TTTAATATTAGGAGCCCCAGATATAGCTTTCCCCCGAATAAATAATATAAGATTTTGATTACTACCCCCTTCATTAACCTTATTAATTTCTAGAAGA
ATTGTAGAAAATGGAGCAGGAACTGGATGAACAGTTTACCCCCCTTTATCATCTAATATTGCTCATAGAGGAAGTTCAGTTGATTTAGCTATTTTTT
CCCTACATTTAGCGGGAATTTCATCAATCTTAGGAGCTATTAATTTTATTACAACTATTATCAATATACGAATTAATCATATATCATTCGATCAAAT
ACCTCTTTTTGTATGAGCAGTAGGAATTACTGCTTTACTTTTATTATTATCCTTACCTGTATTAGCTGGTGCTATTACTATATTATTAACAGATCGA
AATCTTAATACTTCATTTTTTGATCCAGCAGGAGGTGGTGATCCTATTTTATATCAACATTTA

EU093016_Tk889 --

TTTAATATTAGGAGCCCCAGATATAGCTTTTCCTCGAATAAATAATATAAGATTTTGATTACTACCCCCCTCATTAACTTTATTAATTTCTAGAAGA
ATTGTAGAAAATGGAGCAGGAACAGGATGAACAGTTTATCCCCCTTTATCATCTAATATTGCTCATAGAATTAGTTCAGTTGATTTAGCTATTTTTT
CTTTACATTTAGCAGGAATTTCATCAATCTTAGGAGCTATTAATTTTATTACAACTATTATTAATATACGAATTAATCATTTATCATTTGATCAAAT

ACCCCTCTTTGTATGAGCAGTAGGAATTACTGCTTTACTTTTATTATTATCTTTACCTGTATTAGCTGGTGCTATTACTATATTATTAACAGATCGA
AATCTTAATACTTCATTTTTTGATCCTGCAGGAGGTGGAGATCCTATTTTATATCAACATTA

EU093015_It878 --

TTTAATATTAGGAGCCCCAGATATAGCTTTCCCCCGAATAAATAATATAAGATTTTGATTACTACCCCCTTCATTAACCTTATTAATTTCTAGAAGA
ATTGTAGAAAATGGAGCAGGAACTGGATGAACAGTTTACCCCCCTTTATCATCTAATATTGCTCATAGAGGAAGTTCAGTTGATTTAGCTATTTTTT
CCTTACATTTAGCGGGAATTTCATCAATCTTAGGAGCTATTAATTTTATTACAACTATTATTAATATACGAATTAATCATATATCATTCGATCAAAT
ACCTCTTTTTGTATGAGCAGTAGGAATTACTGCTTTACTTTTATTATTATCTTTACCTGTATTAGCTGGTGCTATTACTATATTATTAACAGATCGA
AATCTTAATACTTCATTTTTTGATCCAGCAGGAGGTGGTGATCCTATTTTATACCAACATTA

EU093014_Hu875 --

TTTAATATTAGGAGCCCCAGATATAGCTTTTCCTCGAATAAATAATATAAGATTTTGATTACTACCCCCCTCATTAACTTTATTAATTTCTAGAAGA
ATTGTAGAAAATGGAGCAGGAACAGGATGAACAGTTTATCCCCCTTTATCATCTAATATCGCTCATAGAGGTAGTTCAGTTGATTTAGCTATTTTTT
CTTTACATTTAGCGGGAATTTCATCAATCTTAGGAGCTATTAATTTTATTACAACTATTATTAATATACGAATTAACCATATATCATTTGATCAAAT
ACCTCTCTTTGTATGAGCAGTAGGAATTACTGCTTTACTTTTATTATTATCTTTACCTGTATTAGCTGGTGCTATTACTATATTATTAACAGATCGA
AATCTTAATACTTCATTTTTTGATCCTGCAGGAGGTGGAGATCCTATTTTATATCAACATTA

EU093013_Sk872_Cz_Slova --

TTTAATATTAGGAGCCCCAGATATAGCTTTTCCTCGAATAAATAATATAAGATTTTGATTACTACCCCCCTCATTAACTTTATTAATTTCTAGAAGA
ATTGTAGAAAATGGAGCAGGAACAGGATGAACAGTTTATCCCCCTTTATCATCTAATATCGCTCATAGAGGTAGTTCAGTTGATTTAGCTATTTTTT
CTTTACATTTGGCAGGAATTTCATCAATCTTAGGAGCTATTAATTTTATTACAACTATTATTAATATACGAATTAATCATATATCATTTGATCAAAT
ACCTCTCTTTGTATGAGCAGTAGGAATTACTGCTTTACTTTTATTATTATCTTTACCTGTATTAGCTGGTGCTATTACTATATTATTAACAGATCGA
AATCTTAATACTTCATTTTTTGATCCTGCAGGAGGTGGAGATCCTATTTTATATCAACATTA

EU093011_Cz869 --

TTTAATATTAGGAGCCCCAGATATAGCTTTTCCTCGAATAAATAATATAAGATTTTGATTACTACCCCCCTCATTAACTTTATTAATTTCTAGAAGA
ATTGTAGAAAATGGAGCAGGAACAGGATGAACAGTTTATCCCCCTTTATCATCTAATATTGCTCATAGAGGTAGTTCAGTTGATTTAGCTATTTTTT
CTTTACATTTAGCAGGAATTTCATCAATCTTAGGAGCTATTAATTTTATTACAACTATTATTAATATACGAATTAACCATATATCATTTGATCAAAT
ACCTCTCTTTGTATGAGCAGTAGGAATTACTGCTTTACTTTTATTATTATCTTTACCTGTATTAGCTGGTGTTATTACTATATTATTAACAGATCGA
AATCTTAATACTTCATTTTTTGATCCTGCAGGAGGTGGAGATCCTATTTTATATCAACATTA

EU093010_Ru_Bosnia_Fi_Bu_Hu_Bel_Po_Rom_Ukr_ --

TTTAATATTAGGAGCCCCAGATATAGCTTTTCCTCGAATAAATAATATAAGATTTTGATTACTACCCCCCTCATTAACTTTATTAATTTCTAGAAGA
ATTGTAGAAAATGGAGCAGGAACAGGATGAACAGTTTATCCCCCTTTATCATCTAATATCGCTCATAGAGGTAGTTCAGTTGATTTAGCTATTTTTT
CTTTACATTTAGCAGGAATTTCATCAATCTTAGGAGCTATTAATTTTATTACAACTATTATTAATATACGAATTAACCATATATCATTTGATCAAAT
ACCTCTCTTTGTATGAGCAGTAGGAATTACTGCTTTACTTTTATTATTATCTTTACCTGTATTAGCTGGTGCTATTACTATATTATTAACAGATCGA
AATCTTAATACTTCATTTTTTGATCCTGCAGGAGGTGGAGATCCTATTTTATATCAACATTA

EU093009_Kz848 --

TTTAATATTAGGGGCCCCAGATATAGCTTTTCCTCGAATAAATAATATAAGATTTTGATTATTACCCCCTTCATTAACTTTATTAATTTCTAGAAGA
ATTGTAGAAAATGGAGCAGGAACAGGATGAACAGTTTATCCCCCTTTATCATCTAATATCGCTCATAGAGGTAGTTCAGTTGATTTAGCTATTTTTT
CTTTACATTTAGCAGGAATTTCATCAATCTTAGGAGCTATTAATTTTATTACAACTATTATTAATATACGAATTAATCATATATCATTTGATCAAAT
ACCTCTCTTTGTATGAGCAGTAGGAATTACTGCTTTACTTTTATTACTATCTTTACCTGTATTAGCTGGTGCTATTACTATATTATTAACAGATCGA
AATCTTAATACTTCATTTTTTGATCCTGCAGGAGGTGGAGATCCTATTTTATATCAACATTA

EU093007_Fi810 --

TTTAATATTAGGAGCCCCAGATATAGCCTTTCCTCGAATAAATAATATAAGATTTTGATTACTACCCCCCTCATTAACTTTATTAATTTCTAGAAGA
ATTGTAGAAAATGGAGCAGGAACAGGATGAACAGTTTATCCCCCTTTATCATCTAATATCGCTCATAGAGGTAGTTCAGTTGATTTAGCTATTTTTT
CTTTACATTTAGCAGGAATTTCATCAATCTTAGGAGCTATTAATTTTATTACAACTATTATTAATATACGAATTAACCATATATCATTTGATCAAAT
ACCTCTCTTTGTATGAGCAGTAGGAATTACTGCTTTACTTTTATTATTATCTTTACCTGTATTAGCTGGTGCTATTACTATATTATTAACAGATCGA
AATCTTAATACTTCATTTTTTGATCCTGCAGGAGGTGGAGATCCTATTTTATATCAACATTA

EU093004_Tu999 --

TTTAATATTAGGAGCTCCAGATATAGCTTTTCCTCGAATAAATAACATAAGATTTTGATTACTCCCCCCTTCATTAACTTTATTAATTTCTAGAAGA
ATTGTAGAAAATGGAGCAGGAACAGGATGAACAGTTTATCCCCCTTTATCATCTAATATTGCTCATAGAGGTAGTTCAGTTGATTTAGCTATTTTTT
CTTTACATTTAGCAGGAATTTCATCAATCTTAGGAGCTATTAATTTTATTACAACTATTATTAATATACGAATTAATAATATATCATTTGATCAAAT
ACCTCTTTTTGTATGAGCAGTAGGAATTACTGCATTACTTTTATTATTATCTTTACCTGTATTAGCTGGTGCTATTACTATATTATTAACAGATCGA
AATCTTAATACTTCATTTTTTGATCCCGCAGGAGGTGGAGATCCTATTTTATATCAACACTTA

EU093000_Bu961 --

TTTAATATTAGGAGCCCCAGATATAGCTTTTCCTCGAATAAATAATATAAGATTTTGATTACTACCCCCCTCATTAACTTTATTAATTTCTAGAAGA
ATTGTAGAAAATGGAGCAGGAACAGGATGAACAGTTTATCCCCCTTTATCATCTAATATCGCTCATAGAGGTAGTTCAGTTGATTTAGCTATTTTTT

CTTTACATTTAGCAGGAATTTCATCAATCTTAGGAGCTATTAATTTTATTACAACTATTATTAATATACGAATTAATCATATATCATTTGATCAAAT
ACCTCTCTTTGTATGAGCAGTAGGAATTACTGCTTTACTTTTATTATTATCTTTACCTGTATTAGCTGGTGCTATTACTATATTATTAACAGATCGA
AATCTTAATACTTCATTTTTTGATCCTGCAGGAGGTGGAGATCCTATTTTATATCAACATTTA

EU092998_Bu951 ---

TTTAATATTAGGAGCCCCAGATATAGCTTTTCCTCGAATAAATAATATAAGATTTTGATTACTACCCCCCTCATTAACTTTATTAATTTCTAGAAGA
ATTGTAGAAAATGGAGCAGGAACAGGATGAACAGTTTATCCCCCTTTATCATCTAATATCGCTCATAGTGGTAGTTCAGTTGATTTAGCTATTTTTT
CTTTACATTTAGCAGGAATTTCATCAATCTTAGGAGCTATTAATTTTATTACAACTATTATTAATATACGAATTAACCATATATCATTTGATCAAAT
ACCTCTCTTTGTATGAGCAGTAGGAATTACTGCTTTACTTTTATTATTATCTTTACCTGTATTAGCTGGTGCTATTACTATATTATTAACAGATCGA
AATCTTAATACTTCATTTTTTGATCCTGCAGGAGGTGGAGATCCTATTTTATATCAACATTTA

EU092997_Ru940 ---

TTTAATATTAGGAGCCCCAGATATAGCTTTTCCTCGAATAAATAATATAAGATTTTGATTACTACCCCCCTCATTAACTTTATTAATTTCTAGAAGA
ATTGTAGAAAATGGAGCAGGAACAGGATGAACAGTTTATCCCCCTTTATCATCTAATATCGCTCATAGAGGTAGTTCAGTTGATTTAGCTATTTTTT
CTTTACATTTAGCAGGAATTTCATCAATCTTAGGAGCTATTAATTTTATTACAACTATTATTAATATACGAATTAACCATATATCATTTGATCAAAT
ACCTCTCTTTGTATGAGCAGTAGGAATTACTGCTTTACTTTTATTATTATCTCTACCTGTATTAGCTGGTGCTATTACTATATTATTAACAGATCGA
AATCTTAATACTTCATTTTTTGATCCTGCAGGAGGTGGGGATCCTATTTTATATCAACATTTA

EU092996_Sp924_Fr ---

TTTAATATTAGGAGCCCCAGATATAGCTTTCCCCCGAATAAATAATATAAGATTTTGATTACTACCCCCTTCATTAACCTTATTAATTTCTAGAAGA
ATTGTAGAAAATGGAGCAGGAACTGGATGAACAGTTTACCCCCCTTTATCATCCAATATTGCTCATAGAGGAAGTTCAGTTGATTTAGCTATTTTTT
CCCTACATTTAGCAGGAATTTCATCAATCTTAGGAGCTATTAATTTTATTACAACTATTATTAATATACGAATTAATCATATATCATTCGATCAAAT
ACCTCTTTTTGTGTGAGCAGTAGGAATTACTGCTTTACTTTTATTATTATCCTTACCTGTATTAGCTGGTGCTATTACTATATTATTAACAGATCGA
AATCTTAATACTTCATTTTTTGATCCAGCAGGAGGTGGTGATCCTATTTTATATCAACATTTA

EU092995_Sp923 ---

TTTAATATTAGGAGCCCCAGATATAGCTTTCCCCCGAATAAATAATATAAGATTTTGATTACTACCCCCTTCATTAACCTTATTAATTTCTAGAAGA
ATTGTAGAAAATGGAGCAGGAACTGGATGAACAGTTTATCCCCCTTTATCATCCAATATTGCTCATAGAGGAAGTTCAGTTGATTTAGCTATTTTTT
CCCTACATTTAGCGGGAATTTCATCAATCTTAGGAGCTATTAATTTTATTACAACTATTATTAATATACGAATTAATCATATATCATTCGATCAAAT
ACCTCTTTTTGTGTGAGCAGTAGGAATTACTGCTTTACTTTTATTATTATCCTTACCTGTATTAGCTGGTGCTATTACTATATTATTAACGGATCGA
AATCTTAATACTTCATTTTTTGATCCAGCAGGAGGTGGTGATCCTATTTTATATCAACATTTA

EU092994_Sp921 ---

TTTAATATTAGGAGCCCCAGATATAGCTTTCCCCCGAATAAATAATATAAGATTTTGATTACTACCCCCTTCATTAACCTTATTAATTTCTAGAAGA
ATTGTAGAAAATGGAGCAGGAACTGGATGAACAGTTTACCCCCCTTTATCATCCAATATTGCTCATAGAGGAAGTTCAGTTGATTTAGCTATTTTTT
CCCTACATTTAGCAGGAATTTCATCAATCTTAGGAGCTATTAATTTTATTACAACTATTATTAATATACGAATTAATCATATATCATTCGATCAAAT
ACCTCTTTTTGTGTGAGCAGTAGGAATTACTGCTTTACTTTTATTATTATCCTTACCTGTATTAGCTGGTGCTATTACTATATTATTAACGGATCGA
AATCTTAATACTTCATTTTTTGATCCAGCAGGAGGTGGTGATCCTATTTTATATCAACATTTA

EU092993_Sp917 ---

TTTAATATTAGGAGCCCCAGATATAGCTTTCCCCCGAATAAATAATATAAGATTTTGATTACTACCCCCTTCATTAACCTTATTAATTTCTAGAAGA
ATTGTAGAAAATGGAGCAGGAACTGGATGAACAGTTTATCCCCCTTTATCATCCAATATTGCTCATAGAGGAAGTTCAGTTGATTTAGCTATTTTTT
CCCTACATTTAGCAGGAATTTCATCAATCTTAGGAGCTATTAATTTTATTACAACTATTATTAATATACGAATTAATCATATATCATTCGATCAAAT
ACCTCTTTTTGTGTGAGCAGTAGGAATTACTGCTTTACTTTTATTATTATCCTTACCTGTATTAGCTGGTGCTATTACTATATTATTAACAGATCGA
AATCTTAATACTTCATTTTTTGATCCAGCAGGAGGTGGTGATCCTATTTTATATCAACATTTA

EU092992_Sp916 ---

TTTAATATTAGGAGCCCCAGATATAGCTTTCCCCCGAATAAATAATATAAGATTTTGATTACTACCCCCTTCATTAACCTTATTAATTTCTAGAAGA
ATTGTAGAAAATGGAGCAGGAACTGGATGAACAGTTTACCCCCCTTTATCATCCAATATTGCTCATAGAGGAAGTTCAGTTGATTTAGCTATTTTTT
CCCTACATTTAGCAGGAATTTCATCAATCTTAGGAGCTATTAATTTTATTACAACTATTATTAATATACGAATTAATCATATATCATTCGATCAAAT
ACCTCTTTTTGTGTGAGCAGTAGGAATTACTGCTTTACTTTTATTATTATCCTTACCTGTATTAGCTGGTGCTATTACTATATTATTAACAGATCGA
AATATTAATACTTCATTTTTTGATCCAGCAGGAGGTGGTGATCCTATTTTATATCAACATTTA

EU092991_Fr913 ---

TTTAATATTAGGAGCCCCAGATATAGCTTTCCCCCGAATAAATAATATAAGATTTTGATTACTACCCCCTTCATTAACCTTATTAATTTCTAGAAGA
ATTGTAGAAAATGGAGCAGGAACTGGATGAACAATTTACCCCCCTTTATCATCCAATATTGCTCATAGAGGAAGTTCAGTTGATTTAGCTATTTTTT
CCCTACATTTAGCAGGAATTTCATCAATCTTAGGAGCTATTAATTTTATTACAACTATTATTAATATACGAATTAATCATATATCATTCGATCAAAT
ACCTCTTTTTGTGTGAGCAGTAGGAATTACTGCTTTACTTTTATTATTATCCTTACCTGTATTAGCTGGTGCTATTACTATATTATTAACAGATCGA
AATCTTAATACTTCATTTTTTGATCCAGCAGGAGGTGGTGATCCTATTTTATATCAACATTTA

EU092990_Fr911 ---

TTTAATATTAGGAGCCCCAGATATAGCTTTCCCCCGAATAAATAATATAAGATTTTGATTACTACCCCCTTCATTAACCTTATTAATTTCTAGAAGA

ATTGTAGAAAATGGAGCAGGAACTGGATGAACAGTTTACCCCCCTTTATCATCTAATATTGCTCATAGAGGAAGTTCAGTTGATTTAGCTATTTTTT
CCTTACATTTAGCAGGAATTTCATCAATCTTAGGAGCTATTAATTTTATTACAACTATTATTAATATACGAATTAATCATATATCATTCGATCAAAT
ACCTCTTTTTGTATGAGCAGTAGGAATTACTGCTTTACTTTTATTATTATCATTACCTGTATTAGCTGGTGCTATTACTATATTATTAACAGATCGA
AATCTTAATACTTCATTTTTTGATCCAGCAGGGGGTGGTGATCCTATTTTATATCAACATTTA

EU092988_It639 --

TTTAATATTAGGAGCCCCAGATATAGCTTTCCCCCGAATAAATAATATAAGATTTTGATTACTACCCCCTTCATTAACCTTATTAATTTCTAGAAGA
ATTGTAGAAAATGGAGCAGGAACTGGATGAACAGTTTACCCCCCTTTATCATCTAATATTGCTCATAGAGGAAGTTCAGTTGATTTAGCTATTTTTT
CCCTACATTTAGCGGGAATTTCATCAATCTTAGGAGCTATTAATTTTATTACAACTATTATTAATATACGAATTAATCATATATCATTTGATCAAAT
ACCTCTTTTTGTATGAGCAGTAGGAATTACTGCTTTACTTTTATTATTATCCTTACCTGTATTAGCTGGTGCTATTACTATATTATTAACAGATCGA
AATCTTAATACTTCATTTTTTGATCCAGCAGGAGGTGGTGATCCTATTTTATATCAACATTTA

EU092987_Fr528 --

TTTAATATTAGGAGCCCCAGATATAGCTTTCCCCCGAATAAATAATATAAGATTTTGATTACTACCCCCTTCATTAACCTTATTAATTTCTAGAAGA
ATTGTAGAAAATGGAGCAGGAACTGGATGAACAGTTTACCCCCCTTTATCATCTAATATTGCTCATAGAGGAAGTTCAGTTGATTTAGCTATTTTTT
CCCTACATTTAGCGGGAATTTCATCAATCTTAGGAGCTATTAATTTTATTACAACTATTATTAATATACGAATTAATCATATATCATTCGATCAAAT
ACCTCTTTTTGTATGAGCAGTAGGAATTACTGCTTTACTTTTATTATTATCCTTACCTGTATTAGCTGGTGCTATTACTATATTATTAACAGATCGA
AATCTTAATACTTCATTTTTTGATCCAGCAGGAGGTGGTGATCCTATTTTATATCAACATTTA

EU092985_Fr525 --

TTTAATATTAGGAGCCCCAGATATAGCTTTCCCCCGAATAAATAATATAAGATTTTGATTACTACCCCCTTCATTAACCTTATTAATTTCTAGAAGA
ATTGTAGAAAATGGAGCAGGAACTGGATGAACAGTTTACCCCCCTTTATCATCTAATATTGCTCATAGAGGAAGTTCAGTTGATTTAGCTATTTTTT
CCTTACATTTAGCAGGAATTTCATCAATCTTAGGAGCTATTAATTTTATTACAACTATTATTAATATACGAATTAATCATATATCATTCGATCAAAT
ACCTCTTTTTGTATGAGCAGTAGGAATTACTGCTTTACTTTTATTATTATCATTACCTGTATTAGCTGGTGCTATTACTATATTATTAACAGATCGA
AATCTTAATACTTCATTTTTTGATCCAGCAGGAGGTGGTGATCCTATTTTATATCAACATTTA

EU092984_Fr522 --

TTTAATATTAGGAGCCCCAGATATAGCTTTCCCCCGAATAAATAATATAAGATTTTGATTACTACCCCCTTCATTAACCTTATTAATTTCTAGAAGA
ATTGTAGAAAATGGAGCAGGAACTGGATGAACAGTTTACCCCCCTTTATCATCTAATATTGCTCATAGAGGAAGTTCAGTTGATTTAGCTATTTTTT
CCTTACATTTAGCAGGAATTTCATCAATCTTAGGAGCTATTAATTTTATTACAACTATTATTAATATACGAATTAATCATATATCATTCGATCAAAT
ACCTCTTTTTGTATGAGCAGTAGGAATTACTGCTTTACTCTTATTATTATCATTACCTGTATTAGCTGGTGCTATTACTATATTATTAACAGATCGA
AATCTTAATACTTCATTTTTTGATCCAGCAGGGGGTGGTGATCCTATTTTATATCAACATTTA

EU092983_Ch765 --

TTTAATATTAGGAGCCCCAGATATAGCTTTCCCCCGAATAAATAATATAAGATTTTGATTACTACCCCCTTCATTAACCTTATTAATTTCTAGAAGA
ATTGTAGAAAATGGAGCAGGAACTGGATGAACAGTTTACCCCCCTTTATCATCTAATATTGCTCATAGAGGAAGTTCAGTTGATTTAGCTATTTTTT
CCCTACATTTAGCAGGAATTTCATCAATCTTAGGAGCTATTAATTTTATTACAACTATTATTAATATACGAATTAATCATATATCATTCGATCAAAT
ACCTCTTTTTGTATGAGCAGTAGGAATTACTGCTTTACTTTTATTATTATCCTTACCTGTATTAGCTGGTGCTATTACTATATTATTAACAGATCGA
AATCTTAATACTTCATTTTTTGATCCAGCAGGAGGTGGTGATCCTATTTTATATCAACATTTA

EU092982_Pl653_Slova_Ukr --

TTTAATATTAGGAGCCCCAGATATAGCTTTTCCTCGAATAAATAATATAAGATTTTGATTACTACCCCCCTCATTAACTTTATTAATTTCTAGAAGA
ATTGTAGAAAATGGAGCAGGAACAGGATGAACAATTTATCCCCCTTTATCATCTAATATCGCTCATAGAGGTAGTTCAGTTGATTTAGCTATTTTTT
CTTTACATTTAGCAGGAATTTCATCAATCTTAGGAGCTATTAATTTTATTACAACTATTATTAATATACGAATTAACCATATATCATTTGATCAAAT
ACCTCTCTTTGTATGAGCAGTAGGAATTACTGCTTTACTTTTATTATTATCTTTACCTGTATTAGCTGGTGCTATTACTATATTATTAACAGATCGA
AATCTTAATACTTCATTTTTTGATCCTGCAGGAGGTGGAGATCCTATTTTATATCAACATTTA

EU092981_At626 --

TTTAATATTAGGAGCCCCAGATATAGCTTTTCCTCGAATAAATAATATAAGATTTTGATTACTACCCCCCTCATTAACTTTATTAATTTCTAGAAGA
ATTGTAGAAAATGGAGCAGGAACAGGATGAACAGTTTATCCCCCTTTATCATCTAATATCGCTCATAGAGGTAGTTCAGTTGATTTAGCTATTTTTT
CTTTACATTTAGCAGGAATTTCATCAATCTTAGGAGCTATTAATTTTATTACAACTATTATTAATATACGAATTAACCATATATCATTTGATCAAAT
ACCTCTCTTTGTATGAGCAGTAGGAATTACTGCTTTACTTTTATTATTATCTTTACCTGTGTTAGCTGGTGCTATTACTATATTATTAACAGATCGA
AATCTTAATACTTCATTTTTTGATCCTGCAGGAGGTGGAGATCCTATTTTATATCAACATTTA

EU092980_At622 --

TTTAATATTAGGAGCCCCAGATATAGCTTTTCCTCGAATAAATAATATAAGATTTTGATTACTACCCCCCTCATTAACTTTATTAATTTCTAGAAGA
ATTGTAGAAAATGGAGCAGGAACAGGATGAACAGTTTATCCCCCTTTATCATCTAATATTGCTCATAGAGGTAGTTCAGTTGATTTAGCTATTTTTT
CTTTACATTTAGCAGGAATTTCATCAATCTTAGGAGCTATTAATTTTATTACAACTATTATTAATATACGAATTAACCATATATCATTTGATCAAAT
ACCTCTCTTTGTATGAGCAGTAGGAATTACTGCTTTACTTTTATTACTATCTTTACCTGTATTAGCTGGTGCTATTACTATATTATTAACAGATCGA
AATCTTAATACTTCATTTTTTGATCCTGCAGGAGGTGGAGATCCTATTTTATATCAACATTTA

EU092978_At618_At_Slo --

TTTAATATTAGGAGCCCCAGATATAGCTTTTCCTCGAATAAATAATATAAGATTTTGATTACTACCCCCCTCATTAACTTTATTAATTTCTAGAAGA
ATTGTAGAAAATGGGGCAGGAACAGGATGAACAGTTTATCCCCCTTTATCATCTAATATCGCTCATAGAGGTAGTTCAGTTGATTTAGCTATTTTTT
CTTTACATTTAGCAGGAATTTCATCAATCTTAGGAGCTATTAATTTTATTACAACTATTATTAATATACGAATTAACCATATATCATTTGATCAAAT
ACCTCTCTTTGTATGAGCAGTAGGAATTACTGCTTTACTTTTATTATTATCTTTACCTGTATTAGCTGGTGCTATTACTATATTATTAACAGATCGA
AATCTTAATACTTCATTTTTTGATCCTGCAGGAGGTGGAGATCCTATTTTATATCAACATTTA

EU092977_At606_At_Cz_D_Fi_Hu_Po_Slova --

TTTAATATTAGGAGCCCCAGATATAGCTTTTCCTCGAATAAATAATATAAGATTTTGATTACTACCCCCCTCATTAACTTTATTAATTTCTAGAAGA
ATTGTAGAAAATGGAGCAGGAACAGGATGAACAGTTTATCCCCCTTTATCATCTAATATTGCTCATAGAGGTAGTTCAGTTGATTTAGCTATTTTTT
CTTTACATTTAGCAGGAATTTCATCAATCTTAGGAGCTATTAATTTTATTACAACTATTATTAATATACGAATTAACCATATATCATTTGATCAAAT
ACCTCTCTTTGTATGAGCAGTAGGAATTACTGCTTTACTTTTATTATTATCTTTACCTGTATTAGCTGGTGCTATTACTATATTATTAACAGATCGA
AATCTTAATACTTCATTTTTTGATCCTGCAGGAGGTGGAGATCCTATTTTATATCAACATTTA

EU092976_At602 --

TTTAATATTAGGAGCCCCAGATATAGCTTTTCCTCGAATAAATAATATAAGATTTTGATTACTACCCCCCTCATTAACTTTATTAATTTCTAGAAGA
ATTGTAGAAAATGGAGCAGGAACAGGATGAACAGTTTATCCCCCTTTATCATCTAATATTGCTCATAGAGGTAGTTCAGTTGATTTAGCTATTTTTT
CTTTACATTTAGTAGGAATTTCATCAATCTTAGGAGCTATTAATTTTATTACAACTATTATTAATATACGAATTAACCATATATCATTTGATCAAAT
ACCTCTCTTTGTATGAGCAGTAGGAATTACTGCTTTACTTTTATTATTATCTTTACCTGTATTAGCTGGTGCTATTACTATATTATTAACAGATCGA
AATCTTAATACTTCATTTTTTGATCCTGCAGGAGGTGGAGATCCTATTTTATATCAACATTTA

EU092975_D757 --

TTTAATATTAGGAGCCCCAGATATAGCTTTTCCTCGAATAAATAATATAAGATTTTGATTACTACCCCCCTCATTAACTTTATTAATTTCTAGAAGA
ATTGTAGAAAATGGAGCAGGAACAGGATGAACAGTTTATCTTCCTTTATCATCTAATATTGCTCATAGAGGTAGTTCAGTTGATTTAGCTATTTTTT
CTTTACATTTAGCAGGAATTTCATCAATCTTAGGAGCTATTAATTTTATTACAACTATTATTAATATACGAATTAACCATATATCATTTGATCAAAT
ACCTCTCTTTGTATGAGCAGTAGGAATTACTGCTTTACTTTTATTATTATCTTTACCTGTATTAGCTGGTGCTATTACTATATTATTAACAGATCGA
AATCTTAATACTTCATTTTTTGATCCTGCAGGAGGTGGAGATCCTATTTTATATCAACATTTA

EU092974_D756 --

TTTAATATTAGGAGCCCCAGATATAGCTTTTCCTCGAATAAATAATATAAGATTTTGATTACTACCCCCCTCATTAACTTTATTAATTTCTAGAAGA
ATTGTAGAAAATGGAGCAGGAACAGGATGAACAATTTATCCCCCTTTATCATCTAATATTGCTCATAGAGGTAGTTCAGTTGATTTAGCTATTTTTT
CTTTACATTTAGCAGGAATTTCATCAATCTTAGGAGCTATTAATTTTATTACAACTATTATTAATATACGAATTAACCATATATCATTTGATCAAAT
ACCTCTCTTTGTATGAGCAGTAGGAATTACTGCTTTACTTTTATTATTATCTTTACCTGTATTAGCTGGTGCTATTACTATATTATTAACAGATCGA
AATCTTAATACTTCATTTTTTGATCCTGCAGGAGGTGGAGATCCTATTTTATATCAACATTTA

EU092973_D744 --

TTTAATATTAGGAGCCCCAGATATAGCTTTTCCTCGAATAAATAATATAAGATTTTGATTACTACCCCCCTCATTAACTTTATTAATTTCTAGAAGA
ATTGTAGAAAATGGAGCAGGAACAGGATGAACGGTTTATCCCCCTTTATCATCTAATATTGCTCATAGAGGTAGTTCAGTTGATTTAGCTATTTTTT
CTTTACATTTAGCAGGAATTTCATCAATCTTAGGAGCTATTAATTTTATTACAACTATTATTAATATACGAATTAACCATATATCATTTGATCAAAT
ACCTCTCTTTGTATGAGCAGTAGGAATTACTGCTTTACTTTTATTATTATCTTTACCTGTATTAGCTGGTGCTATTACTATATTATTAACAGATCGA
AATCTTAATACTTCATTTTTTGATCCTGCAGGAGGTGGAGATCCTATTTTATATCAACATTTA

EU092971_Hu697_Bu_Hu --

TTTAATATTAGGAGCCCCAGATATAGCTTTTCCTCGAATAAATAATATAAGATTTTGATTACTACCCCCCTCATTAACTTTATTAATTTCTAGAAGA
ATTGTAGAAAATGGAGCAGGAACAGGATGAACAGTTTATCCCCCTTTATCATCTAATATCGCTCATAGAGGTAGTTCAGTTGATTTAGCTATTTTTT
CTTTACATTTAGCAGGAATTTCATCAATCTTAGGAGCTATTAATTTTATTACAACTATTATTAATATACGAATTAACCATATATCATTTGATCAAAT
ACCTCTCTTTGTATGAGCAGTAGGAATTACTGCTTTACTTTTATTATTATCTTTACCTGTATTAGCTGGTGCTATTACTATATTATTAACAGATCGA
AATCTTAATACTTCATTTTTTGATCCTGCAGGAGGTGGAGATCCTATTTTATATCAACATTTA

EU092969_Bu775 --

TTTAATATTAGGAGCCCCAGATATAGCTTTTCCTCGAATAAATAATATAAGATTTTGATTACTACCCCCCTCATTAACTTTATTAATTTCTAGAAGA
ATTGTAGAAAATGGAGCAGGAACAGGATGAACAATTTATCCCCCTTTATCATCTAATATCGCTCACAGAGGTAGTTCAGTTGATTTAGCTATTTTTT
CTTTACATTTAGCAGGAATTTCATCAATCTTAGGAGCTATTAATTTTATTACAACTATTATTAATATACGAATTAACCATATATCATTTGATCAAAT
ACCTCTCTTTGTATGAGCAGTAGGAATTACTGCTTTACTTTTATTATTATCTTTACCTGTATTAGCTGGTGCTATTACTATATTATTAACAGATCGA
AATCTTAATACTTCATTTTTTGATCCTGCAGGAGGTGGAGATCCTATTTTATATCAACATTTA

EU092968_Bu772 --

TTTAATATTAGGAGCCCCAGATATAGCTTTTCCTCGAATAAATAATATAAGATTTTGATTACTACCCCCCTCATTAACTTTATTAATTTCTAGAAGA
ATTGTAGAAAATGGAGCAGGAACAGGATGAACAGTTTATCCCCCTTTATCATCTAATATCGCTCATAGAGGTAGTTCAGTTGATTTAGCTATTTTTT
CTTTACATTTAGCAGGAATTTCATCAATCTTAGGAGCTATTAATTTTATTACAACTATTATTAATATACGAATTAACCATATATCATTTGATCAAAT
ACCTCTCTTTGTATGAGCAGTAGGAATTACTGCTTTACTTTTATTATTATCTCTACCTGTATTAGCTGGTGCTATTACTATATTATTAACAGATCGA
AATCTTAATACTTCATTTTTTGATCCTGCAGGAGGTGGAGATCCTATTTTATATCAACATTTA

EU093003_D982 --

TTTAATATTAGGAGCCCCAGATATAGCTTTTCCTCGAATAAATAATATAAGATTTTGATTACTACCCCCCTCATTAACTTTATTAATTTCTAGAAGA
ATTGTAGAAAATGGAGCAGGAACAGGATGAACAGTTTATCCCCCTTTATCATCTAATATTGCTCATAGAGGTAGTTCAGTTGATTTAGCTATTTTTT
CTTTACATTTAGCAGGAATTTCATCAATCTTAGGAGCTATTAATTTTATTACAACTATTATTAATATACGAATTAACCATATATCATTTGATCAAAT
ACCTCTCTTTGTATGGGCAGTAGGAATTACTGCTTTACTTTTATTATTATCTTTACCTGTATTAGCTGGTGCTATTACTATATTATTAACAGATCGA
AATCTTAATACTTCATTTTTTGATCCTGCAGGAGGTGGAGATCCTATTTTATATCAACATTTA

DQ407769_UP_100_F
ACATTATATTTTATTTTTGGTATTTGAGCAGGTATAGTAGGAACCTCTTTAAGATTATTAATTCGTACTGAATTAGGTAATCCTGGATCTTTAATTG
GAGATGATCAAATTTATAACACTATTGTAACAGCTCATGCTTTTATTATAATTTTTTTTATAGTTATACCAATTATAATTGGAGGATTTGGAAATTG
ATTAATTCCTTTAATATTAGGAGCCCCAGATATAGCTTTCCCCCGAATAAATAATATAAGATTTTGATTACTACCCCCTTCATTAACCTTATTAATT
TCTAGAAGAATTGTAGAAAATGGAGCAGGAACTGGATGAACAGTTTACCCCCCTTTATCATCTAATATTGCTCATAGAGGAAGTTCAGTTGATTTAG
CTATTTTTTCCTTACATTTAGCAGGAATTTCATCAATCTTAGGAGCTATTAATTTTATTACAACTATTATTAATATACGAATTAATCATATATCATT
CGATCAAATACCTCTTTTTGTATGAGCAGTAGGAATTACTGCTTTACTCTTATTATTATCATTACCTGTATTAGCTGGTGCTATTACTATATTATTA
ACAGATCGAAATCTTAATACTTCATTTTTTGATCCAGCAGGGGGTGGTGA----------------------

AM231426_W336_parvisi_Gre
ACACTATATTTTATTTTTGGTATTTGAGCAGGTATAGTAGGAACCTCTTTAAGATTATTAATTCGTACTGAATTAGGTAATCCTGGATCTTTAATTG
GAGATGATCAAATTTATAATACTATTGTAACAGCTCATGCTTTTATTATAATTTTTTTTATAGTTATACCAATTATAATTGGAGGATTTGGAAATTG
ATTAATTCCTTTAATATTAGGAGCCCCAGATATAGCTTTTCCTCGAATAAATAATATAAGATTTTGATTACTACCCCCCTCATTAACTTTATTAATT
TCTAGAAGAATTG????????????????????GGATGAACAGTTTATCCCCCTTTATCATCTAATATCGCTCATAGAGGTAGTTCAGTTGATTTAG
CTATTTTTTCTTTACATTTAGCAGGAATTTCATCAATCTTAGGGGCTATTAATTTTATTACAACTATTATTAATATACGAATTAACCATATATCATT
TGATCAAATACCTCTCTTTGTATGAGCAGTAGGAATTACTGCTTTACTTTTATTATTATCTTTACCTGTATTAGCTGGTGCTATTACTATATTATTA
ACAGATCGAAATCTTAATACTTCATTTTTTGATCYTGCAGGAGGTGGAGATCCTATTTTATATCAA------

AM231425_W333_angorae_Tur
ACACTATATTTTATTTTTGGTATTTGAGCAGGTATAGTAGGAACCTCTTTAAGATTATTAATTCGTACTGAATTAGGTAATCCTGGATCTTTAATTG
GGGATGATCAAATTTATAATACTATTGTAACAGCTCATGCTTTTATTATAATTTTTTTTATAGTTATACCAATTATAATTGGAGGATTTGGAAATTG
ATTAATTCCTTTAATATTAGGAGCCCCAGATATAGCTTTTCCTCGAATAAATAATATAAGATTTTGATTATTACCCCCCTCATTAACTTTATTAATT
TCTAGAAGAATTG????????????????????GGATGAACAGTTTATCCCCCTTTATCATCTAATATTTCTCATAGAGGTAGTTCAGTTGATTTAG
CTATTTTTTCTTTACATTTAGCAGGAATTTCATCAATCTTAGGAGCTATTAATTTTATTACAACTATTATTAATATACGAATTAATCATATATCATT
TGATCAAATACCCCTTTTTGTATGGGCAGTAGGAATTACTGCTTTACTTTTATTATTATCTTTACCTGTATTAGCTGGTGCTWTTACTATATTATTA
ACAGATCGAAATCTTAATACTTCATTTTTCGATCCCGCAGGAGGTGGGGATCCTATTTTATATCAA------

AM231424_W331_angorae_Tur_Ankara
ACACTATATTTTATTTTTGGTATTTGAGCAGGTATAGTAGGAACCTCTTTAAGATTATTAATTCGTACTGAATTAGGTAATCCTGGATCTTTAATTG
GAGATGATCAAATTTATAATACTATTGTAACAGCTCATGCTTTTATTATAATTTTTTTTATAGTTATACCAATTATAATTGGAGGATTTGGAAATTG
ATTAATTCCTTTAATATTAGGAGCCCCAGATATAGCTTTTCCTCGAATAAATAATATAAGATTTTGATTATTACCCCCCTCATTAACTTTATTAATT
TCTAGAAGAATTGTA??????????????????GGATGAACAGTTTATCCCCCTTTATCATCTAATATCGCTCATAGAGGTAGCTCAGTTGATTTAG
CTATTTTTTCTTTACATTTAGCAGGAATTTCATCAATCTTAGGAGCTATTAATTTTATTACAACTATTATTAATATACGAATTAACCATATATCATT
TGATCAAATACCTCTCTTTGTATGAGCAGTAGGAATTACCGCTTTACTTTTATTATTATCTTTACCTGTATTAGCTGGTGCTATTACTATATTATTA
ACAGATCGAAATCTTAATACTTCATTTTTTGATCCTGCAGGAGGTGGAGATCCTATTTTATATCAA------

AM231423_W329_gigantea_Uzb_Chatkal
ACACTATATTTTATTTTTGGTATTTGAGCAGGTATAGTAGGAACCTCTTTAAGATTATTAATTCGTACTGAATTAGGTAATCCTGGATCTTTAATTG
GAGATGATCAAATTTATAATACTATTGTAACAGCTCATGCTTTTATTATAATTTTTTTTATAGTTATACCAATTATAATTGGAGGATTTGGAAATTG
ATTAATTCCTTTAATATTAGGAGCCCCAGATATAGCTTTTCCTCGAATAAATAATATAAGATTTTGATTATTACCCCCTTCATTAACTTTATTAATT
TCTAGAAGAATTG????????????????????GGATGAACAGTTTATCCCCCTTTATCATCTAATATCGCTCATAGAGGTAGTTCAGTTGATTTAG
CTATTTTTTCTTTACATTTAGCAGGAATTTCATCAATCTTAGGAGCTATTAATTTTATTACAACTATTATTAATATACGAATTAATCATATATCATT
TGATCAAATACCTCTCTTTGTATGAGCAGTAGGAATTACTGCTTTACTTTTATTACTATCTTTACCTGTATTAGCTGGTGCTATTACTATATTATTA
ACAGATCGAAATCTTAATACTTCATTTTTTGATCCTGCAGGAGGTGGAGATCCTATTTTATATCAA------

AM231422_W330_orientalis_Zailijskiy
ACACTATATTTTATTTTTGGTATTTGAGCAGGTATAGTAGGAACCTCTTTAAGATTATTAATTCGTACTGAATTAGGTAATCCTGGATCTTTAATTG
GAGATGATCAAATTTATAATACTATTGTAACAGCTCATGCTTTTATTATAATTTTTTTTATAGTTATACCAATTATAATTGGAGGATTTGGAAATTG
ATTAATTCCTTTAATATTAGGGGCCCCAGATATAGCTTTTCCTCGAATAAATAATATAAGATTTTGATTATTACCCCCTTCATTAACTTTATTAATT
TCTAGAAGAATTGTAGAAAATGGAGCAGGAACAGGATGAACAGTTTATCCCCCTTTATCATCTAATATCGCTCATAGAGGTAGTTCAGTTGATTTAG
CTATTTTTTCTTTACATTTAGCGGGAATTTCATCAATCTTAGGAGCTATTAATTTTATTACAACTATTATTAATATACGAATTAATCATATATCATT
TGATCAAATACCTCTCTTTGTATGAGCAGTAGGAATTACTGCTTTACTTTTATTACTATCTTTACCTGTATTAGCTGGTGCTATTACTATATTATTA
ACAGATCGAAATCTTAATACTTCATTTTTTGATCCTGCAGGAGGTGGAGATCCTATTTTATATCAA------

AM231421_W292_ochracea_Tadj_Zeravsh_
ACACTATATTTTATTTTTGGTATTTGAGCAGGTATAGTAGGAACCTCTTTAAGATTATTAATTCGTACTGAATTAGGTAATCCTGGATCTTTAATTG
GAGATGATCAAATTTATAATACTATTGTAACAGCTCATGCTTTTATTATAATTTTTTTCATAGTTATACCAATTATAATTGGAGGATTTGGAAATTG
ATTAATTCCTTTAATATTAGGAGCCCCAGATATAGCTTTTCCTCGAATAAATAATATAAGATTTTGATTACTACCCCCTTCATTAACTTTATTAATT
TCCAGAAGAATTGTAGAAAATGGAGCAGGAACAGGATGAACAGTTTATCCCCCTTTATCATCTAATATCGCTCATAGAGGTAGTTCAGTTGATTTAG
CTATTTTTTCTTTACATTTAGCAGGAATTTCATCAATTTTAGGAGCTATTAATTTCATTACAACTATTATTAATATACGAATTAACCATATATCATT
TGATCAAATACCCCTCTTTGTATGAGCAGTAGGAATTACTGCTTTACTTTTATTATTATCTTTACCTGTATTAGCTGGTGCTATTACTATATTATTA
ACAGATCGAAATCTTAATACTTCATTTTTTGATCCTGCAGG??GTGGAGATCCTATTTTATATCAA------

AM231420_W280_farsica_Iran_Fars
ACACTATATTTTATTTTCGGTATTTGAGCAGGTATAGTAGGAACCTCTTTAAGATTATTAATTCGTACTGAATTAGGTAATCCTGGATCTTTAATTG
GAGATGATCAAATTTATAATACTATTGTAACAGCTCATGCTTTTATTATAATCTTTTTTATAGTTATACCAATTATAATTGGAGGATTTGGAAATTG
ATTAATTCCTTTAATATTAGGAGCTCCAGATATAGCTTTCCCCCGAATAAATAATATAAGATTTTGATTACTCCCCCCCTCATTAACTTTATTAATT
TCTAGAAGAATTGTAGAAAATGGAGCAGGAACAGGATGAACAGTTTACCCCCCTTTATCATCTAATATTGCCCACAGAGGTAGTTCAGTTGATTTAG
CTATTTTTTCTTTACATTTAGCAGGAATTTCATCAATTTTAGGAGCTATTAATTTTATTACAACTATTATTAATATACGAATTAATTATATATCATT
TGATCAAATACCTCTTTTTGTATGAGCAGTAGGAATTACAGCATTACTTTTATTATTATCTTTACCTGTATTAGCTGGTGCTATTACTATATTATTA
ACAGATCGAAATCTTAACACTTCATTTTTTGATCCTGCA????GTGGGGATCCTATTTTATATCAA------

AM231419_W311_pseudonubilosus_Iran_Urmia
ACACTATATTTTATTTTCGGTATTTGAGCGGGTATAGTAGGAACCTCTTTAAGATTATTAATTCGTACTGAATTAGGTAATCCTGGATCTTTAATTG
GAGATGATCAAATTTATAACACTATTGTAACAGCTCATGCTTTTATTATAATTTTTTTTATAGTTATACCAATTATAATTGGAGGATTTGGAAATTG
ATTAATTCCTTTAATATTAGGAGCTCCAGATATAGCTTTTCCTCGAATAAATAATATAAGATTTTGATTACTCCCCCCCTCATTAACTTTATTAATT
TCTAGAAGAATTGTAGAAAATGGAGCAGGAACAGGATGAACAATTTATCCCCCTTTATCATCTAATATTGCTCATAGAGGTAGTTCAGTTGATTTAG
CTATTTTTTCTTTACATTTAGCAGGAATTTCATCAATCTTAGGAGCTATTAATTTTATTACAACTATTATTAACATACGAATTAATCATATATCATT
TGATCAAATACCTCTTTTTGTATGAGCAGTAGGAATTACTGCATTACTTTTATTATTATCTTTACCTGTATTAGCTGGTGCTATTACTATATTATTA
ACAGATCGAAATCTTAATACTTCATTTTTTGATCCTGCAGGA?GTGGAGATCCTATTTTATATCAA------

AM231418_W335_sheljuzhkoi_Tur_Adana
ACACTATATTTTATTTTTGGTATTTGAGCAGGTATAGTAGGAACCTCTTTAAGATTATTAATTCGTACTGAATTAGGTAATCCTGGATCTTTAATTA
GAGATGATCAAATTTATAATACTATTGTAACAGCTCATGCTTTTATTATAATTTTTTTTATAGTTATACCAATTATAATTGGAGGATTTGGAAATTG
ATTAATTCCTTTAATATTAGGAGCCCCAGATATAGCTTTTCCTCGAATAAATAATATAAGATTTTGATTACTTCCCCCTTCATTAACTTTATTAATT
TCTAGAAGAATTG????????????????????GGATGAACAGTTTATCCCCCTTTATCATCTAATATTGCTCATAGAGGTAGTTCAGTTGATTTAG
CTATTTTTTCTTTACATTTAGCAGGAATTTCATCAATCTTAGGAGCTATTAATTTTATTACAACTATTATTAATATACGAATTAATAATATATCATT
TGATCAAATACCTCTTTTTGTATGAGCAGTAGGAATTACTGCATTACTTTTATTATTATCCCTACCTGTATTAGCTGGTGCTATTACTATATTATTA
ACAGATCGAAATCTTAATACTTCATTTTTTGATCCCGCAGGAGGTGGAGATCCTATTTTATATCAA------

AM231417_W78_parmenides_Fr
ACATTATATTTTATTTTTGGTATTTGAGCAGGTATAGTAGGAACCTCTTTAAGATTATTAATTCGTACTGAATTAGGTAATCCTGGATCTTTAATTG
GAGATGATCAAATTTATAATACTATTGTAACAGCTCATGCTTTTATTATAATTTTTTTTATAGTTATACCAATTATAATTGGAGGATTTGGAAATTG
ATTAATTCCTTTAATATTAGGAGCCCCAGATATAGCTTTCCCCGAATAAATAATATAAGATTTTGATTACTACCCCCTTCATTAACCTTATTAATT
TCTAGAAGAATTGTAGAAAATGGAGCAGGAACTGGATGAACAGTTTACCCCCCTTTATCATCTAATATTGCTCATAGAGGAAGTTCAGTTGATTTAG
CTATTTTTTCCTTACATTTAGCAGGAATTTCATCAATCTTAGGAGCTATTAATTTTATTACAACTATTATTAATATACGAATTAATCATATATCATT
CGATCAAATACCTCTTTTTGTATGAGCAGTAGGAATTACTGCTTTACTCTTATTATTATCATTACCTGTATTAGCTGGTGCTATTACTATATTATTA
ACAGATCGAAATCTTAATACTTCATTTTTTGATCCAGCAGGGGGTGGTGATCCTATTTTATATCAA------

EU836682_h_1_32_Rus ---

GCTTTTCCTCGAATAAATAATATAAGATTTTGATTACTACCCCCCTCATTAACTTTATTAATTTCTAGAAGAATTGTAGAAAATGGAGCAGGAACAG
GATGAACAGTTTATCCCCCTTTATCATCTAATATCGCTCATAGAGGTAGTTCAGTTGATTTAGCTATTTTTTCTTTACATTTAGCAGGAATTTCATC
AATCTTAGGAGCTATTAATTTTATTACAACTATTATTAATATACGAATTAACCATATATCATTTGATCAAATACCTCTCTTTGTATGAGCAGTAGGA
ATTACTGCTTTACTTTTATTATTATCTTTACCTGTATTAGCTGGTGCTATTACTATATTATTAACAGATCGAAATCTTAATACTTCATTTTTTGATC
CTGCAGGAGGTGGGGATCCTATTTTATATCAACATTTA

EU836681_h_1_31_Rus ---

GCTTTTCCTCGAATAAATAATATAAGATTTTGATTACTACCCCCCTCATTAACTTTATTAATTTCTAGAAGAATTGTAGAAAATGGAGCAGGAACAG
GATGAACAATTTATCCCCCTTTATCATCTAATATCGCTCATAGAGGTAGTTCAGTTGATTTAGCTATTTTTTCTTTACATTTAGCAGGAATTTCATC
AATCTTAGGAGCTATTAATTTTATTACAACTATTATTAATATACGAATTAACCATATATCATTTGATCAAATACCTCTCTTTGTATGAGCAGTAGGA
ATTACTGCTTTACTTTTATTATTATCTTTACCTGTATTAGCTGGTGCTATTACTATATTATTAACAGATCGAAATCTTAATACTTCATTTTTTGATC
CTGCAGGAGGTGGGGATCCTATTTTATATCAACATTTA

EU836680_A_Bo_Bu_Cr_Cz_Hu_Sl_Li_Fi_Po_Ru ---

GCTTTTCCTCGAATAAATAATATAAGATTTTGATTACTACCCCCCTCATTAACTTTATTAATTTCTAGAAGAATTGTAGAAAATGGAGCAGGAACAG
GATGAACAGTTTATCCCCCTTTATCATCTAATATCGCTCATAGAGGTAGTTCAGTTGATTTAGCTATTTTTTCTTTACATTTAGCAGGAATTTCATC
AATCTTAGGAGCTATTAATTTTATTACAACTATTATTAATATACGAATTAACCATATATCATTTGATCAAATACCTCTCTTTGTATGAGCAGTAGGA
ATTACTGCTTTACTTTTATTATTATCTTTACCTGTATTAGCTGGTGCTATTACTATATTATTAACAGATCGAAATCTTAATACTTCATTTTTTGATC
CTGCAGGAGGTGGAGATCCTATTTTATATCAACATTTA

EU836675_h_1_19_It ---

GCTTTCCCTCGAATAAATAATATAAGATTTTGATTACTACCCCCCTCATTAACTTTATTAATTTCTAGAAGAATTGTAGAAAATGGAGCAGGAACAG
GATGAACAGTTTATCCCCCTTTATCATCTAATATTGCTCATAGAGGTAGTTCAGTTGATTTAGCTATTTTTTCTTTACATTTAGCAGGAATTTCATC
AATCTTAGGAGCTATTAATTTTATTACAACTATTATTAATATACGAATTAACCATATATCATTTGATCAAATACCTCTCTTTGTATGAGCAGTAGGA
ATTACTGCTTTACTTTTATTATTATCTTTACCTGTATTAGCTGGTGCTATTACTATATTATTAACAGATCGAAATCTTAATACTTCATTTTTTGATC
CTGCAGGAGGTGGAGATCCTATTTTATATCAACATTTA

EU836674_h_1_18_At_It ---

GCTTTTCCTCGAATAAATAATATAAGATTTTGATTACTACCCCCCTCATTAACTTTATTAATTTCTAGAAGAATTGTAGAAAATGGAGCAGGAACAG
GATGAACAGTTTATCCCCCTTTATCATCTAATATTGCTCATAGAGGTAGTTCAGTTGATTTAGCTATTTTTTCTTTACATTTAGCAGGAATTTCATC
AATCTTAGGAGCTATTAATTTTATTACAACTATTATTAATATACGAATTAACCATATATCATTTGATCAAATACCTCTCTTTGTATGAGCAGTAGGA
ATTACTGCTTTACTTTTATTATTATCTTTACCTGTATTAGCTGGTGCTATTACTATATTATTAACAGATCGAAATCTTAATACTTCATTTTTTGATC
CTGCAGGAGGTGGAGATCCTATTTTATATCAACATTTA

EU836672_h_1_10_It ---

GCTTTCCCTCGAATAAATAATATAAGATTTTGATTACTACCCCCCTCATTAACTTTATTAATTTCTAGAAGAATTGTAGAAAATGGGGCAGGAACAG
GATGAACAGTTTATCCCCCTTTATCATCTAATATCGCTCATAGAGGTAGTTCAGTTGATTTAGCTATTTTTTCTTTACATTTAGCAGGAATTTCATC
AATCTTAGGAGCTATTAATTTTATTACAACTATTATTAATATACGAATTAACCATATATCATTTGATCAAATACCTCTCTTTGTATGAGCAGTAGGA
ATTACTGCTTTACTTTTATTATTATCTTTACCTGTATTAGCTGGTGCTATTACTATATTATTAACAGATCGAAATCTTAATACTTCATTTTTTGATC
CTGCAGGAGGTGGAGATCCTATTTTATATCAACATTTA

EU836671_h_1_09_It --

GCTTTTCCTCGAATAAATAATATAAGATTTTGATTACTACCCCCCTCATTAACTTTATTAATTTCTAGAAGAATTGTAGAAAATGGGGCAGGAACAG
GATGAACAGTTTATCCCCCTTTATCATCTAATATCGCTCATAGAGGTAGTTCAGTTGATTTAGCTATTTTTTCTTTACATTTAGCAGGAATTTCATC
AATCTTAGGAGCTATTAATTTTATTACAACTATTATTAATATACGAATTAACCATATATCATTTGATCAAATACCTCTCTTTGTATGAGCAGTAGGA
ATTACTGCTTTACTTTTATTATTATCTTTACCTGTATTAGCTGGTGCTATTACTATATTATTAACAGATCGAAATCTTAATACTTCATTTTTTGATC
CTGCAGGAGGTGGAGATCCTATTTTATATCAACATTTA

EU836670_h_1_07_Gre --

GCTTTTCCTCGAATAAATAATATAAGATTTTGATTACTACCCCCCTCATTAACTTTATTAATTTCTAGAAGAATTGTAGAAAATGGAGCAGGAACAG
GATGAACAATTTATCCCCCTTTATCATCTAATATCGCTCATAGAGGTAGTTCAGTTGATTTAGCTATTTTTTCTTTACATTTAGCAGGAATTTCATC
AATCTTAGGGGCTATTAATTTTATTACAACTATTATTAATATACGAATTAACCATATATCATTTGATCAAATACCTCTCTTTGTATGAGCAGTAGGA
ATTACTGCTTTACTTTTATTATTATCTTTACCTGTATTAGCTGGTGCTATTACTATATTATTAACAGATCGAAATCTTAATACTTCATTTTTTGATC
CTGCAGGAGGTGGAGATCCTATTTTATATCAACATTTA

EU836669_h_1_06_Gre --

GCTTTCCCTCGAATAAATAATATAAGATTTTGATTACTACCCCCCTCATTAACTTTATTAATTTCTAGAAGAATTGTAGAAAATGGAGCAGGAACAG
GATGAACAGTTTATCCCCCTTTATCATCTAATATAGCTCATAGAGGTAGTTCAGTTGATTTAGCTATTTTTTCTTTACATTTAGCAGGAATTTCATC
AATCTTAGGAGCTATTAATTTTATTACAACTATTATTAATATACGAATTAACCATATATCATTTGATCAAATACCTCTCTTTGTATGAGCAGTAGGA
ATTACTGCTTTACTTTTATTATTATCTTTACCTGTATTAGCTGGTGCTATTACTATATTATTAACAGATCGAAATCTTAATACTTCATTTTTTGATC
CTGCAGGAGGTGGAGATCCTATTTTATATCAACATTTA

EU836668_h_1_05_Gre --

GCTTTCCCTCGAATAAATAATATAAGATTTTGATTACTACCCCCCTCATTAACTTTATTAATTTCTAGAAGAATTGTAGAAAATGGAGCAGGAACAG
GATGAACAGTTTATCCCCCTTTATCATCTAATATCGCTCATAGAGGTAGTTCAGTTGATTTAGCTATTTTTTCTTTACATTTAGCAGGAATTTCATC
AATCTTAGGGGCTATTAATTTTATTACAACTATTATTAATATACGAATTAACCATATATCATTTGATCAAATACCTCTCTTTGTATGAGCAGTAGGA
ATTACTGCTTTACTTTTATTATTATCTTTACCTGTATTAGCTGGTGCTATTACTATATTATTAACAGATCGAAATCTTAATACTTCATTTTTTGATC
CTGCAGGAGGTGGAGATCCTATTTTATATCAACATTTA

EU836667_h_1_04_Gre --

GCTTTCCCTCGAATAAATAATATAAGATTTTGATTACTACCCCCCTCATTAACTTTATTAATTTCTAGAAGAATTGTAGAAAATGGAGCAGGAACAG
GATGAACAGTTTATCCCCCTTTATCATCTAATATCGCTCATAGAGGTAGTTCAGTTGATTTAGCTATTTTTTCTTTACATTTAGCAGGAATTTCATC
AATCTTAGGGGCTATTAATTTTATTACAACTATTATTAATATACGAATTAACCATATATCATTTGATCAAATACCTCTCTTTGTATGAGCAGTAGGA
ATTACTGCTTTACTTTTATTATTATCTTTACCCGTATTAGCTGGTGCTATTACTATATTATTAACAGATCGAAATCTTAATACTTCATTTTTTGATC
CTGCAGGAGGTGGAGATCCTATTTTATATCAACATTTA

EU836666_h_1_03_Mac --

GCTTTTCCTCGAATAAATAATATAAGATTTTGATTACTACCCCCCTCATTAACTTTATTAATTTCTAGAAGAATTGTAGAAAATGGAGCAGGAACAG
GATGAACAGTTTATCCCCCTTTATCATCTAATATCGCTCATAGAGGTAGTTCAGTTGATTTAGCTATTTTTTCTTTACATTTAGCGGGAATTTCATC
AATCTTAGGGGCTATTAATTTTATTACAACTATTATTAATATACGAATTAACCATATATCATTTGATCAAATACCTCTCTTTGTATGAGCAGTAGGA
ATTACTGCTTTACTTTTATTATTATCTTTACCTGTATTAGCTGGTGCTATTACTATATTATTAACAGATCGAAATCTTAATACTTCATTTTTTGATC
CTGCAGGAGGTGGAGATCCTATTTTATATCAACATTTA

EU836665_h_1_02_Gre_Mac --

GCTTTTCCTCGAATAAATAATATAAGATTTTGATTACTACCCCCCTCATTAACTTTATTAATTTCTAGAAGAATTGTAGAAAATGGAGCAGGAACAG
GATGAACAGTTTATCCCCCTTTATCATCTAATATCGCTCATAGAGGTAGTTCAGTTGATTTAGCTATTTTTTCTTTACATTTAGCAGGAATTTCATC
AATCTTAGGGGCTATTAATTTTATTACAACTATTATTAATATACGAATTAACCATATATCATTTGATCAAATACCTCTCTTTGTATGAGCAGTAGGA
ATTACTGCTTTACTTTTATTATTATCTTTACCTGTATTAGCTGGTGCTATTACTATATTATTAACAGATCGAAATCTTAATACTTCATTTTTTGATC
CTGCAGGAGGTGGAGATCCTATTTTATATCAACATTTA

EU836664_h_2_06_At_It --

GCTTTTCCTCGAATAAATAATATAAGATTTTGATTGCTACCCCCCTCATTAACTTTATTAATTTCTAGAAGAATTGTAGAAAATGGAGCAGGAACAG
GATGAACAGTTTATCCCCCTTTATCATCTAATATCGCTCATAGAGGTAGTTCAGTTGATTTAGCTATTTTTTCTTTACATTTAGCAGGAATTTCATC
AATCTTAGGAGCTATTAATTTTATTACAACTATTATTAATATACGAATTAACCATATATCATTTGATCAAATACCTCTCTTTGTATGAGCAGTAGGA
ATTACTGCTTTACTTTTATTATTATCTTTACCTGTATTAGCTGGTGCTATTACTATATTATTAACAGATCGAAATCTTAATACTTCATTTTTTGATC
CTGCAGGAGGTGGAGATCCTATTTTATATCAACATTTA

EU836663_h_2_05_It --

GCTTTCCCTCGAATAAATAATATAAGATTTTGATTGCTACCCCCCTCATTAACTTTATTAATTTCTAGAAGAATTGTAGAAAATGGAGCAGGAACAG
GATGAACAGTTTATCCCCCTTTATCATCTAATATCGCTCATAGAGGTAGTTCAGTTGATTTAGCTATTTTTTCTTTACATTTAGCAGGAATTTCATC
AATCTTAGGAGCTATTAATTTTATTACAACTATTATTAATATACGAATTAACCATATATCATTTGATCAAATACCTCTCTTTGTATGAGCAGTAGGA

```
ATTACTGCTTTACTTTTATTATTATCTTTACCTGTATTAGCTGGTGCTATTACTATATTATTAACAGATCGAAATCTTAATACTTCATTTTTTGATC
CTGCAGGAGGTGGAGATCCTATTTTATATCAACATTTA;


END;
begin mrbayes;
	set autoclose=yes nowarn=yes;
	partition ancstates = 2: 1, 2-655;
	set partition=ancstates;
	lset applyto=(2) rates=invgamma nst=6;
	unlink statefreq=(all) revmat=(all) shape=(all) pinvar=(all);
	prset ratepr=variable;
	constraint mnemosyne_clade=LOWAM001_Kyrgyzstan_Alai_10 LOWAM004_Uzbekistan_Gissar_38
LOWAM007_Kyrgyzstan_TianShan24 LOWAM009_Kyrgyzstan_TianShan_6 LOWAM013_Turkey_Georgia_3
LOWAM014_Turkey_Ovitdagi_1 LOWAM015_Don_Borisovka_Saratov LOWAM018_Kyrgyzstan_Karamyk
LOWAM019_Kyrgyzstan_Karamyk LOWAM021_Russia_Saratov LOWAM023_Saratov_Luga_Vologda_4
LOWAM027_Kazakhstan_Karatau_2 LOWAM037_Russia_Pskovskaya LOWAM038_Kyrgyzstan_Tjuz_Ashu_3
LOWAM039_Kyrgyzstan_23 LOWAM044_Russia_Borisovra_3 LOWAM045_Tajikistan_Shakhristan_3
LOWAM067_Kazakhstan_Kaindy_1 LOWAM069_Russia_Teberda_2 LOWAM073_Kyrgyzstan_Takhtalyk1
LOWAM074_Kyrgyzstan_Naryntoo LOWAM086_Turkmenistan_3 LOWAM087_Uzbekistan_Sarchashma
LOWAM095_Kyrgyzstan_Sosnovka LOWAM096_Gissar_5 LOWAM098_Tajikistan_Khondiza1
LOWAM099_Uzbekistan_Kamchik_2 LOWAM100_Kyrgyzstan_Konduk LOWAM115_Azerbaijan_Nyus_Nyus
LOWAM116_Iran_Dizin_2 LOWAM122_Russia_Saratow_1 LOWAM136_Uzbekistan_Tamshush_1
LOWAM149_Kyrgyzstan_Songkel_1 LOWAM198_Turkey_Aladaglar_2 LOWAM213_Tajikistan_Tandukul_1
Parnassius_ariadne_ariadne Parnassius_ariadne_AC4_14 HQ004911_RV_07_C107_Romania
HQ004908_RV_07_D060_Romania HQ004907_RV_08_M274_Romania HQ004902_RV_08_M361_Romania
GU947642_Pmne_ITNEB01_Italy_Sicily EU093018_Ca995 EU093017_Ch892 EU093016_Tk889 EU093015_It878
EU093014_Hu875 EU093013_Sk872_Cz_Slova EU093011_Cz869 EU093010_Ru_Bosnia_Fi_Bu_Hu_Bel_Po_Rom_Ukr_
EU093009_Kz848 EU093007_Fi810 EU093004_Tu999 EU093000_Bu961 EU092998_Bu951 EU092997_Ru940
EU092996_Sp924_Fr EU092995_Sp923 EU092994_Sp921 EU092993_Sp917 EU092992_Sp916 EU092991_Fr913
EU092990_Fr911 EU092988_It639 EU092987_Fr528 EU092985_Fr525 EU092984_Fr522 EU092983_Ch765
EU092982_Pl653_Slova_Ukr EU092981_At626 EU092980_At622 EU092978_At618_At_Slo
EU092977_At606_At_Cz_D_Fi_Hu_Po_Slova EU092976_At602 EU092975_D757 EU092974_D756 EU092973_D744
EU092971_Hu697_Bu_Hu EU092969_Bu775 EU092968_Bu772 EU093003_D982 DQ407769_UP_100_F
AM231426_W336_parvisi_Gre AM231425_W333_angorae_Tur AM231424_W331_angorae_Tur_Ankara
AM231423_W329_gigantea_Uzb_Chatkal AM231422_W330_orientalis_Zailijskiy
AM231421_W292_ochracea_Tadj_Zeravsh_ AM231420_W280_farsica_Iran_Fars
AM231419_W311_pseudonubilosus_Iran_Urmia AM231418_W335_sheljuzhkoi_Tur_Adana
AM231417_W78_parmenides_Fr EU836682_h_1_32_Rus EU836681_h_1_31_Rus
EU836680_A_Bo_Bu_Cr_Cz_Hu_Sl_Li_Fi_Po_Ru EU836675_h_1_19_It EU836674_h_1_18_At_It
EU836672_h_1_10_It EU836671_h_1_09_It EU836670_h_1_07_Gre EU836669_h_1_06_Gre EU836668_h_1_05_Gre
EU836667_h_1_04_Gre EU836666_h_1_03_Mac EU836665_h_1_02_Gre_Mac EU836664_h_2_06_At_It
EU836663_h_2_05_It;
	prset topologypr=constraints(mnemosyne_clade);
	report applyto=(1) ancstates=yes;
	mcmcp ngen= 100000 relburnin=yes burninfrac=0.25 printfreq=100  samplefreq=100 nchains=4
savebrlens=yes;
	mcmc;
	sump;
end;
```

Alignment and command block for reconstruction of the ancestral states in the clade (2) [(*mnemosyne* +*ariadne*)+(*stubbendorfii*+*glacialis*+*hoenei*)]

```
#NEXUS

BEGIN DATA;
	DIMENSIONS  NTAX=125 NCHAR=655;
	format datatype=mixed(standard:1,DNA:2-655) interleave=yes gap=- missing=?;
	MATRIX
	Parnassius_orleans_AC10_5                 0
	LOWAM001_Kyrgyzstan_Alai_10               1
	LOWAM004_Uzbekistan_Gissar_38             1
	LOWAM007_Kyrgyzstan_TianShan24            1
	LOWAM009_Kyrgyzstan_TianShan_6            1
	LOWAM013_Turkey_Georgia_3                 1
	LOWAM014_Turkey_Ovitdagi_1                1
	LOWAM015_Don_Borisovka_Saratov            1
	LOWAM018_Kyrgyzstan_Karamyk               1
	LOWAM019_Kyrgyzstan_Karamyk               1
	LOWAM021_Russia_Saratov                   1
	LOWAM023_Saratov_Luga_Vologda_4           1
	LOWAM027_Kazakhstan_Karatau_2             1
```

LOWAM037_Russia_Pskovskaya	1
LOWAM038_Kyrgyzstan_Tjuz_Ashu_3	1
LOWAM039_Kyrgyzstan_23	1
LOWAM044_Russia_Borisovra_3	1
LOWAM045_Tajikistan_Shakhristan_3	1
LOWAM067_Kazakhstan_Kaindy_1	1
LOWAM069_Russia_Teberda_2	1
LOWAM073_Kyrgyzstan_Takhtalyk1	1
LOWAM074_Kyrgyzstan_Naryntoo	1
LOWAM086_Turkmenistan_3	1
LOWAM087_Uzbekistan_Sarchashma	1
LOWAM095_Kyrgyzstan_Sosnovka	1
LOWAM096_Gissar_5	1
LOWAM098_Tajikistan_Khondiza1	1
LOWAM099_Uzbekistan_Kamchik_2	1
LOWAM100_Kyrgyzstan_Konduk	1
LOWAM115_Azerbaijan_Nyus_Nyus	1
LOWAM116_Iran_Dizin_2	1
LOWAM122_Russia_Saratow_1	1
LOWAM136_Uzbekistan_Tamshush_1	1
LOWAM149_Kyrgyzstan_Songkel_1	1
LOWAM198_Turkey_Aladaglar_2	1
LOWAM213_Tajikistan_Tandukul_1	1
Parnassius_clodius	0
Parnassius_eversmanni_2005_LOWA_108	0
Parnassius_eversmanni_2005_LOWA_107	0
Parnassius_stubbendorfii_2005_LOWA_815	1
Parnassius_stubbendorfii_2005_LOWA_154	1
Parnassius_stubbendorfii_2005_LOWA_153	1
Parnassius_nordmanni	0
Parnassius_eversmanni	0
Parnassius_eversmanni_felderi	0
Parnassius_ariadne_ariadne	0
Parnassius_glacialis_mikado	1
Parnassius_stubbendorfii_hoenei	1
Parnassius_stubbendorfii_hoenei_AC20_16	1
Parnassius_stubbendorfii_koreanus	1
Parnassius_nordmanni_AC20_5	0
Parnassius_eversmanni_felderi_AC23_68	0
Parnassius_eversmanni_eversmanni_AC1_14	0
Parnassius_clodius_AC4_5	0
Parnassius_ariadne_AC4_14	0
HQ004911_RV_07_C107_Romania	1
HQ004908_RV_07_D060_Romania	1
HQ004907_RV_08_M274_Romania	1
HQ004902_RV_08_M361_Romania	1
GU947642_Pmne_ITNEB01_Italy_Sicily	1
EU093018_Ca995	1
EU093017_Ch892	1
EU093016_Tk889	1
EU093015_It878	1
EU093014_Hu875	1
EU093013_Sk872_Cz_Slova	1
EU093011_Cz869	1
EU093010_Ru_Bosnia_Fi_Bu_Hu_Bel_Po_Rom_Ukr_	1
EU093009_Kz848	1
EU093007_Fi810	1
EU093004_Tu999	1
EU093000_Bu961	1
EU092998_Bu951	1
EU092997_Ru940	1
EU092996_Sp924_Fr	1
EU092995_Sp923	1
EU092994_Sp921	1
EU092993_Sp917	1
EU092992_Sp916	1
EU092991_Fr913	1
EU092990_Fr911	1
EU092988_It639	1
EU092987_Fr528	1
EU092985_Fr525	1
EU092984_Fr522	1
EU092983_Ch765	1
EU092982_Pl653_Slova_Ukr	1
EU092981_At626	1
EU092980_At622	1
EU092978_At618_At_Slo	1
EU092977_At606_At_Cz_D_Fi_Hu_Po_Slova	1
EU092976_At602	1
EU092975_D757	1

EU092974_D756 1
EU092973_D744 1
EU092971_Hu697_Bu_Hu 1
EU092969_Bu775 1
EU092968_Bu772 1
EU093003_D982 1
DQ407769_UP_100_F 1
AM231426_W336_parvisi_Gre 1
AM231425_W333_angorae_Tur 1
AM231424_W331_angorae_Tur_Ankara 1
AM231423_W329_gigantea_Uzb_Chatkal 1
AM231422_W330_orientalis_Zailijskiy 1
AM231421_W292_ochracea_Tadj_Zeravsh_ 1
AM231420_W280_farsica_Iran_Fars 1
AM231419_W311_pseudonubilosus_Iran_Urmia 1
AM231418_W335_sheljuzhkoi_Tur_Adana 1
AM231417_W78_parmenides_Fr 1
EU836682_h_1_32_Rus 1
EU836681_h_1_31_Rus 1
EU836680_A_Bo_Bu_Cr_Cz_Hu_Sl_Li_Fi_Po_Ru 1
EU836675_h_1_19_It 1
EU836674_h_1_18_At_It 1
EU836672_h_1_10_It 1
EU836671_h_1_09_It 1
EU836670_h_1_07_Gre 1
EU836669_h_1_06_Gre 1
EU836668_h_1_05_Gre 1
EU836667_h_1_04_Gre 1
EU836666_h_1_03_Mac 1
EU836665_h_1_02_Gre_Mac 1
EU836664_h_2_06_At_It 1
EU836663_h_2_05_It 1

Parnassius_orleans_AC10_5 ------------------------
TGAGCAGGTATAATAGGAACCTCTTTAAGATTATTAATTCGTACTGAATTAGGTAATCCTGGATCTTTAATTGGAGATGATCAAATTTATAACACTA
TTGTAACAGCTCATGCTTTTATTATAATTTTTTTTATAGTTATACCAATTATAATTGGAGGATTTGGAAATTGATTAATTCCATTAATATTAGGAGC
CCCAGATATAGCTTTCCCCCGAATAAATAATATAAGATTTTGACTATTACCCCCCTCATTAACCTTATTAATTTCCAGAAGAATCGTAGAAAATGGA
GCAGGAACTGGATGAACAGTTTATCCCCCTTTATCCTCTAATATTGCCCATAGTGGAAGATCAGTTGATTTAGCTATCTTTTCTTTACATTTAGCTG
GAATTTCATCTATTTTAGGAGCTATTAATTTTATTACAACTATTATTAATATACGAATTAATCATATATCATTTGATCAAATACCCCTTTTCGTATG
AGCAGTAGGAATTACTGCTTTACTTTTATTATTATCTTTACCTGTTTTAGCAGGTGCTATTACTATACTTCTTACAGATCGAAATCTTAATACTTCA
TTTTTTGATCCAGCAGGAGGTGGAGATCCTATTTTATACCAACATTTA

LOWAM001_Kyrgyzstan_Alai_10
ACACTATATTTTATTTTTGGTATTTGAGCAGGTATAGTAGGAACCTCTTTAAGATTATTAATTCGTACTGAATTAGGTAATCCTGGATCTTTAATTG
GAGATGATCAAATTTATAATACTATTGTAACAGCTCATGCTTTTATTATAATTTTTTTCATAGTTATACCAATTATAATTGGAGGATTTGGAAATTG
ATTAATTCCTTTAATATTAGGAGCCCCAGATATAGCTTTTCCTCGAATAAATAATATAAGATTTTGATTACTACCCCCTTCATTAACTTTATTAATT
TCTAGAAGAATTGTAGAAAATGGAGCAGGAACAGGATGAACAGTTTATCCCCCTTTATCATCTAATATCGCTCATAGAGGTAGTTCAGTTGATTTAG
CTATTTTTTCTTTACATTTAGCAGGAATTTCATCAATTTTAGGAGCTATTAATTTCATTACAACTATTATTAATATACGAATTAACCATATATCATT
TGATCAAATACCCCTCTTTGTATGAGCAGTAGGAATTACTGCTTTACTTTTATTATTATCTTTACCTGTATTAGCTGGTGCTATTACTATATTATTA
ACAGATCGAAATCTTAATACTTCATTTTTTGATCCTGCAGGAGGTGGAGATCCTATTTTATATCAACATTTA

LOWAM004_Uzbekistan_Gissar_38
ACACTATATTTTATTTTTGGTATTTGAGCAGGTATAGTAGGAACCTCTTTAAGATTATTAATTCGTACTGAATTAGGTAATCCTGGATCTTTAATTG
GAGATGATCAAATTTATAATACTATTGTAACAGCTCATGCTTTTATTATAATTTTTTTCATAGTTATACCAATTATAATTGGAGGATTTGGAAATTG
ATTAATTCCTTTAATATTAGGAGCCCCAGATATAGCTTTTCCTCGAATAAATAATATAAGATTTTGATTACTACCCCCTTCATTAACTTTATTAATT
TCCAGAAGAATTGTAGAAAATGGAGCAGGAACAGGATGAACAGTTTATCCCCCTTTATCATCTAATATCGCTCATAGAGGTAGTTCAGTTGATTTAG
CTATTTTTTCTTTACATTTAGCAGGAATTTCATCAATTTTAGGAGCTATTAATTTCATTACAACTATTATTAATATACGAATTAACCATATATCATT
TGATCAAATACCCCTCTTTGTATGAGCAGTAGGAATTACTGCTTTACTTTTATTATTATCTTTACCTGTATTAGCTGGTGCTATTACTATATTATTA
ACAGATCGAAATCTTAATACTTCATTTTTTGATCCTGCAGGAGGTGGAGATCCTATTTTATATCAACATTTA

LOWAM007_Kyrgyzstan_TianShan24
ACACTATATTTTATTTTTGGTATTTGAGCAGGTATAGTAGGAACCTCTTTAAGATTATTAATTCGTACTGAATTAGGTAATCCTGGATCTTTAATTG
GAGATGATCAAATTTATAATACTATTGTAACAGCTCATGCTTTTATTATAATTTTTTTTATAGTTATACCAATTATAATTGGAGGATTTGGAAATTG
ATTAATTCCTTTAATATTAGGGGCCCCAGATATAGCTTTTCCTCGAATAAATAATATAAGATTTTGATTATTACCCCCTTCATTAACTTTATTAATT
TCTAGAAGAATTGTAGAAAATGGAGCAGGAACAGGATGAACAGTTTATCCCCCTTTATCATCTAATATCGCTCATAGAGGTAGTTCAGTTGATTTAG
CTATTTTTTCTTTACATTTAGCAGGAATTTCATCAATCTTAGGAGCTATTAATTTTATTACAACTATTATTAATATACGAATTAATCATATATCATT
TGATCAAATACCTCTCTTTGTATGAGCAGTAGGAATTACTGCTTTACTTTTATTACTATCTTTACCTGTATTAGCTGGTGCTATTACTATATTATTA
ACAGATCGAAATCTTAATACTTCATTTTTTGATCCTGCAGGAGGTGGAGATCCTATTTTATATCAACATTTA

LOWAM009_Kyrgyzstan_TianShan_6
ACACTATATTTTATTTTTGGTATTTGAGCAGGTATAGTAGGAACCTCTTTAAGATTATTAATTCGTACTGAATTAGGTAATCCTGGATCTTTGATTG
GAGATGATCAAATTTATAATACTATTGTAACAGCTCATGCTTTTATTATAATTTTTTTTATAGTTATACCAATTATAATTGGAGGATTTGGAAATTG
ATTAATTCCTTTAATATTAGGGGCCCCAGATATAGCTTTTCCTCGAATAAATAATATAAGATTTTGATTATTACCCCCTTCATTAACTTTATTAATT
TCTAGAAGAATTGTAGAAAATGGAGCAGGAACAGGATGAACAGTTTATCCCCCTTTATCATCTAATATCGCTCATAGAGGTAGTTCAGTTGATTTAG
CTATTTTTTCTTTACATTTAGCAGGAATTTCATCAATCTTAGGAGCTATTAATTTTATTACAACTATTATTAATATACGAATTAATCATATATCATT
TGATCAAATACCTCTCTTTGTATGAGCAGTAGGAATTACTGCTTTACTTTTATTACTATCTTTACCTGTATTAGCTGGTGCTATTACTATATTATTA
ACAGATCGAAATCTTAATACTTCATTTTTTGATCCTGCAGGAGGTGGAGATCCTATTTTATATCAACATTTA

LOWAM013_Turkey_Georgia_3
ACACTATATTTTATTTTTGGTATTTGAGCAGGTATAGTAGGAACCTCTTTAAGATTATTAATTCGTACTGAATTAGGTAATCCTGGATCTTTAATTG

GAGATGATCAAATTTATAATACTATTGTAACAGCTCATGCTTTTATTATAATTTTTTTATAGTTATACCAATTATAATTGGAGGATTTGGAAATTG
ATTAATTCCTTTAATATTAGGAGCCCCAGATATAGCTTTTCCTCGAATAAATAATATAAGATTTTGATTACTGCCCCCCTCATTAACTTTATTAATT
TCTAGAAGAATTGTAGAAAATGGAGCAGGAACAGGATGAACAGTTTATCCCCCTTTATCATCTAATATCGCTCATAGAGGTAGTTCAGTTGATTTAG
CTATTTTTTCTTTACATTTAGCAGGAATTTCATCAATCTTAGGAGCTATTAATTTTATTACAACTATTATTAATATACGAATTAACCATATATCATT
TGATCAAATACCTCTCTTTGTATGAGCAGTAGGAATTACTGCTTTACTTTTATTATTATCTTTACCTGTATTAGCTGGTGCTATTACTATATTATTA
ACAGATCGAAATCTTAATACTTCATTTTTTGATCCTGCAGGAGGTGGAGATCCTATTTTATATCAACATTTA

LOWAM014_Turkey_Ovitdagi_1
ACACTATATTTTATTTTTGGTATTTGAGCAGGTATAGTAGGAACCTCTTTAAGATTATTAATTCGTACTGAATTAGGTAATCCTGGATCTTTAATTG
GAGATGATCAAATTTATAATACTATTGTAACAGCTCATGCTTTTATTATAATTTTTTTATAGTTATACCAATTATAATTGGAGGATTTGGAAATTG
ATTAATTCCTTTAATATTAGGAGCCCCAGATATAGCTTTTCCTCGAATAAATAATATAAGATTTTGATTACTGCCCCCCTCATTAACTTTATTAATT
TCCAGAAGAATTGTAGAAAATGGAGCAGGAACAGGATGAACAGTTTATCCCCCTTTATCATCTAATATCGCTCATAGAGGTAGTTCAGTTGATTTAG
CTATTTTTTCTTTACATTTAGCAGGAATTTCATCAATCTTAGGAGCTATTAATTTTATTACAACTATTATTAATATACGAATTAACCATATATCATT
TGATCAAATACCTCTCTTTGTATGAGCAGTAGGAATTACTGCTTTACTTTTATTATTATCTTTACCTGTATTAGCTGGTGCTATTACTATATTATTA
ACAGATCGAAATCTTAATACTTCATTTTTTGATCCTGCAGGAGGTGGAGATCCTATTTTATATCAACATTTA

LOWAM015_Don_Borisovka_Saratov
ACACTATATTTTATTTTTGGTATTTGAGCAGGTATAGTAGGAACCTCTTTAAGATTATTAATTCGTACTGAATTAGGTAATCCTGGATCTTTAATTG
GAGATGATCAAATTTATAATACTATTGTAACAGCTCATGCTTTTATTATAATTTTTTTATAGTTATACCAATTATAATTGGAGGATTTGGAAATTG
ATTAATTCCTTTAATATTAGGAGCCCCAGATATAGCTTTTCCTCGAATAAATAATATAAGATTTTGATTACTACCCCCCTCATTAACTTTATTAATT
TCTAGAAGAATTGTAGAAAATGGAGCAGGAACAGGATGAACAGTTTATCCCCCTTTATCATCTAATATCGCTCATAGAGGTAGTTCAGTTGATTTAG
CTATTTTTTCTTTACATTTAGCAGGAATTTCATCAATCTTAGGAGCTATTAATTTTATTACAACTATTATTAATATACGAATTAACCATATATCATT
TGATCAAATACCTCTCTTTGTATGAGCAGTAGGAATTACTGCTTTACTTTTATTATTATCTTTACCTGTATTAGCTGGTGCTATTACTATATTATTA
ACAGATCGAAATCTTAATACTTCATTTTTTGATCCTGCAGGAGGTGGGGATCCTATTTTATATCAACATTTA

LOWAM018_Kyrgyzstan_Karamyk
ACACTATATTTTATTTTTGGTATTTGAGCAGGTATAGTAGGAACCTCTTTAAGATTATTAATTCGTACTGAATTAGGTAATCCTGGATCTTTAATTG
GAGATGATCAAATTTATAATACTATTGTAACAGCTCATGCTTTTATTATAATTTTTTTATAGTTATACCAATTATAATTGGAGGATTTGGAAATTG
ATTAATTCCTTTAATATTAGGAGCCCCAGATATAGCTTTTCCTCGAATAAATAATATAAGATTTTGATTACTACCCCCTTCATTAACTTTATTAATT
TCTAGAAGAATTGTAGAAAATGGAGCAGGAACAGGATGAACAGTTTATCCCCCTTTATCATCTAATATCGCTCATAGAGGTAGTTCAGTTGATTTAG
CTATTTTTTCTTTACATTTAGCAGGAATTTCATCAATTTTAGGAGCTATTAATTTCATTACAACTATTATTAATATACGAATTAACCATATATCATT
TGATCAAATACCCCTCTTTGTATGAGCAGTAGGAATTACTGCTTTACTTTTATTATTATCTTTACCTGTATTAGCTGGTGCTATTACTATATTATTA
ACAGATCGAAATCTTAATACTTCATTTTTTGATCCAGCAGGAGGTGGAGATCCTATTTTATATCAACATTTA

LOWAM019_Kyrgyzstan_Karamyk
ACACTATATTTTATTTTTGGTATTTGAGCAGGTATAGTAGGAACCTCTTTAAGATTATTAATTCGTACTGAATTAGGTAATCCTGGATCTTTAATTG
GAGATGATCAAATTTATAATACTATTGTAACAGCTCATGCTTTTATTATAATTTTTTTCATAGTTATACCAATTATAATTGGAGGATTTGGAAATTG
ATTAATTCCTTTAATATTAGGAGCCCCAGATATAGCTTTTCCTCGAATAAATAATATAAGATTTTGATTACTACCCCCTTCATTAACTTTATTAATT
TCTAGAAGAATTGTAGAAAATGGAGCAGGAACAGGATGAACA?TTTATCCCCCTTTATCATCTAATATCGCTCATAGAGGTAGTTCAGTTGATTTAG
CTATTTTTTCTTTACATTTAGCAGGAATTTCATCAATTTTAGGGGCTATTAATTTCATTACAACTATTATTAATATACGAATTAACCATATATCATT
TGATCAAATACCCCTCTTTGTATGAGCAGTAGGAATTACTGCTTTACTTTTATTATTATCTTTACCTGTATTAGCTGGTGCTATTACTATATTATTA
ACAGATCGAAATCTTAATACTTCATTTTTTGATCCTGCAGGAGGTGGAGATCCTATTTTATATCAACATTTA

LOWAM021_Russia_Saratov
ACACTATATTTTATTTTTGGTATTTGAGCAGGTATAGTAGGAACCTCTTTAAGATTATTAATTCGTACTGAATTAGGTAATCCTGGATCTTTAATTG
GAGATGATCAAATTTATAATACTATTGTAACAGCTCATGCTTTTATTATAATTTTTTTATAGTTATACCAATTATAATTGGAGGATTTGGAAATTG
ATTAATTCCTTTAATATTAGGAGCCCCAGATATAGCTTTTCCTCGAATAAATAATATAAGATTTTGATTACTACCCCCCTCATTAACTTTATTAATT
TCTAGAAGAATTGTAGAAAATGGAGCAGGAACAGGATGAACAGTTTATCCCCCTTTATCATCTAATATCGCTCATAGAGGTAGTTCAGTTGATTTAG
CTATTTTTTCTTTACATTTAGCAGGAATTTCATCAATCTTAGGAGCTATTAATTTTATTACAACTATTATTAATATACGAATTAACCATATATCATT
TGATCAAATACCTCTCTTTGTATGAGCAGTAGGAATTACTGCTTTACTTTTATTATTATCTTTACCTGTATTAGCTGGTGCTATTACTATATTATTA
ACAGATCGAAATCTTAATAATTCATTTTTTGATCCTGCAGGAGGTGGGGATCCTATTTTATATCAACATTTA

LOWAM023_Saratov_Luga_Vologda_4
ACACTATATTTTATTTTTGGTATTTGAGCAGGTATAGTAGGAACCTCTTTAAGATTATTAATTCGTACTGAATTAGGTAATCCTGGATCTTTAATTG
GAGATGATCAAATTTATAATACTATTGTAACAGCTCATGCTTTTATTATAATTTTTTTATAGTTATACCAATTATAATTGGAGGATTTGGAAATTG
ATTAATTCCTTTAATATTAGGAGCCCCAGATATAGCTTTTCCTCGAATAAATAATATAAGATTTTGATTACTACCCCCCTCATTAACTTTATTAATT
TCTAGAAGAATTGTAGAAAATGGAGCAGGAACAGGATGAACAGTTTATCCCCCTTTATCATCTAATATCGCTCATAGAGGTAGTTCAGTTGATTTAG
CTATTTTTTCTTTACATTTAGCAGGAATTTCATCAATCTTAGGAGCTATTAATTTTATTACAACTATTATTAATATACGAATTAACCATATATCATT
TGATCAAATACCTCTCTTTGTATGAGCAGTAGGAATTACTGCTTTACTTTTATTATTATCTTTACCTGTATTAGCTGGTGCTATTACTATATTATTA
ACAGATCGAAATCTTAATACTTCATTTTTTGATCCTGCAGGAGGTGGAGATCCTATTTTATATCAACATTTA

LOWAM027_Kazakhstan_Karatau_2
ACACTATATTTTATTTTTGGTATTTGAGCAGGTATAGTAGGAACCTCTTTAAGATTATTAATTCGTACTGAATTAGGTAATCCTGGATCTTTAATTG
GAGATGATCAAATTTATAATACTATTGTAACAGCTCATGCTTTTATTATAATTTTTTTATAGTTATACCAATTATAATTGGAGGATTTGGAAATTG
ATTAATTCCTTTAATATTAGGGGCCCCAGATATAGCTTTTCCTCGAATAAATAATATAAGATTTTGATTATTACCCCCTTCATTAACTTTATTAATT
TCTAGAAGAATTGTAGAAAATGGAGCAGGAACAGGATGAACAGTTTATCCCCCTTTATCATCTAATATCGCTCATAGAGGTAGTTCAGTTGATTTAG
CTATTTTTTCTTTACATTTAGCAGGAATTTCATCAATCTTAGGAGCTATTAATTTTATTACAACTATTATTAATATACGAATTAATCATATATCATT
TGATCAAATACCTCTCTTTGTATGAGCAGTAGGAATTACTGCTTTACTTTTATTACTATCCTTACCTGTATTAGCTGGTGCTATTACTATATTATTA
ACAGATCGAAATCTTAATACTTCATTTTTTGATCCTGCAGGAGGTGGAGATCCTATTTTATATCAACATTTA

LOWAM037_Russia_Pskovskaya ---------
TTTATTTTTGGTATTTGAGCAGGTATAGTAGGAACCTCTTTAAGATTATTAATTCGTACTGAATTAGGTAATCCTGGATCTTTAATTGGAGATGATC
AAATTTATAATACTATTGTAACAGCTCATGCTTTTATTATAATTTTTTTATAGTTATACCAATTATAATTGGAGGATTTGGAAATTGATTAATTCC
TTTAATATTAGGAGCCCCAGATATAGCTTTTCCTCGAATAAATAATATAAGATTTTGATTACTACCCCCCTCATTAACTTTATTAATTTCTAGAAGA
ATTGTAGAAAATGGAGCAGGAACAGGATGAACAGTTTATCCCCCTTTATCATCTAATATCGCTCATAGAGGTAGTTCAGTTGATTTAGCTATTTTTT
CTTTACATTTAGCAGGAATTTCATCAATCTTAGGAGCTATTAATTTTATTACAACTATTATTAATATACGAATTAACCATATATCATTTGATCAAAT
ACCTCTCTTTGTATGAGCAGTAGGAATTACTGCTTTACTTTTATTATTATCTTTACCTGTATTAGCTGGTGCTATTACTATATTATTAACAGATCGA
AATCTTAATACTTCATTTTTTGATCCTGCAGGAGGTGGAGATCCTATTTTATATCAACATTTA

LOWAM038_Kyrgyzstan_Tjuz_Ashu_3
ACACTATATTTTATTTTTGGTATTTGAGCAGGTATAGTAGGAACCTCTTTAAGATTATTAATTCGTACTGAATTAGGTAATCCTGGATCTTTAATTG
GAGATGATCAAATTTATAATACTATTGTAACAGCTCATGCTTTTATTATAATTTTTTTTATAGTTATACCAATTATAATTGGAGGATTTGGAAATTG
ATTAATTCCTTTAATATTAGGGGCCCCAGATATAGCTTTTCCTCGAATAAATAATATAAGATTTTGATTATTACCCCCTTCATTAACTTTATTAATT
TCTAGAAGAATTGTAGAAAATGGAGCAGGAACAGGATGAACAATTTATCCCCCTTTATCATCTAATATCGCTCATAGAGGTAGTTCAGTTGATTTAG
CTATTTTTTCTTTACATTTAGCGGGAATTTCATCAATCTTAGGAGCTATTAATTTTATTACAACTATTATTAATATACGAATTAATCATATATCATT
TGATCAAATACCTCTCTTTGTATGAGCAGTAGGAATTACTGCTTTACTTTTATTACTATCTTTACCTGTATTAGCTGGTGCTATTACTATATTATTA
ACAGATCGAAATCTTAATACTTCATTTTTTGATCCTGCAGGAGGTGGAGATCCTATTTTATATCAACATTTA

LOWAM039_Kyrgyzstan_23
ACACTATATTTTATTTTTGGTATTTGAGCAGGTATAGTAGGAACCTCTTTAAGATTATTAATTCGTACTGAATTAGGTAATCCTGGATCTTTAATTG
GAGATGATCAAATTTATAATACTATTGTAACAGCTCATGCTTTTATTATAATTTTTTTTATAGTTATACCAATTATAATTGGAGGATTTGGAAATTG
ATTAATTCCTTTAATATTAGGGGCCCCAGATATAGCTTTTCCTCGAATAAATAATATAAGATTTTGATTATTACCCCCTTCATTAACTTTATTAATT
TCTAGAAGAATTGTAGAAAATGGAGCAGGAACAGGATGAACAGTTTATCCCCCTTTATCATCTAATATCGCTCATAGAGGTAGTTCAGTTGATTTAG
CTATTTTTTCTTTACATTTAGCGGGAATTTCATCAATCTTAGGAGCTATTAATTTTATTACAACTATTATTAATATACGAATTAATCATATATCATT
TGATCAAATACCTCTCTTTGTATGAGCAGTAGGAATTACTGCTTTACTTTTATTACTATCTTTACCTGTATTAGCTGGTGCTATTACTATATTATTA
ACAGATCGAAATCTTAATACTTCATTTTTTGATCCTGCAGGAGGTGGAGATCCTATTTTATATCAACATTTA

LOWAM044_Russia_Borisovra_3
ACACTATATTTTATTTTTGGTATTTGAGCAGGTATAGTAGGAACCTCTTTAAGATTATTAATTCGTACTGAATTAGGTAATCCTGGATCTTTAATTG
GAGATGATCAAATTTATAATACTATTGTAACAGCTCATGCTTTTATTATAATTTTTTTTATAGTTATACCAATTATAATTGGAGGATTTGGAAATTG
ATTAATTCCCTTAATATTAGGAGCCCCAGATATAGCTTTTCCTCGAATAAATAATATAAGATTTTGATTACTACCCCCCTCATTAACTTTATTAATT
TCTAGAAGAATTGTAGAAAATGGAGCAGGAACAGGATGAACAGTTTATCCCCCTTTATCATCTAATATCGCTCATAGAGGTAGTTCAGTTGATTTAG
CTATTTTTTCTTTACATTTAGCAGGAATTTCATCAATCTTAGGAGCTATTAATTTTATTACAACTATTATTAATATACGAATTAACCATATATCATT
TGATCAAATACCTCTCTTTGTATGAGCAGTAGGAATTACTGCTTTACTTTTATTATTATCTTTACCTGTATTAGCTGGTGCTATTACTATATTATTA
ACAGATCGAAATCTTAATACTTCATTTTTTGATCCTGCAGGAGGTGGAGATCCTATTTTATATCAACATTTA

LOWAM045_Tajikistan_Shakhristan_3
ACACTATATTTTATTTTTGGTATTTGAGCAGGTATAGTAGGAACCTCTTTAAGATTATTAATTCGTACTGAATTAGGTAATCCTGGATCTTTAATTG
GAGATGATCAAATTTATAATACTATTGTAACAGCTCATGCTTTTATTATAATTTTTTTCATAGTTATACCAATTATAATTGGAGGATTTGGAAATTG
ATTAATTCCTTTAATATTAGGAGCCCCAGATATAGCTTTTCCTCGAATAAATAATATAAGATTTTGATTACTACCCCCTTCATTAACTTTATTAATT
TCCAGAAGAATTGTAGAAAATGGAGCAGGAACAGGATGAACAGTTTATCCCCCTTTATCATCTAATATTGCTCATAGAGGTAGTTCAGTTGATTTAG
CTATTTTTTCTTTACATTTAGCAGGAATTTCATCAATTTTAGGAGCTATTAATTTCATTACAACTATTATTAATATACGAATTAACCATATATCATT
TGATCAAATACCCCTCTTTGTATGAGCAGTAGGAATTACTGCTTTACTTTTATTATTATCTTTACCTGTATTAGCTGGTGCTATTACTATATTATTA
ACAGATCGAAATCTTAATACTTCATTTTTTGATCCTGCAGGAGGTGGAGATCCTATTTTATATCAACATTTA

LOWAM067_Kazakhstan_Kaindy_1
ACACTATATTTTATTTTTGGTATTTGAGCAGGTATAGTAGGAACCTCTTTAAGATTATTAATTCGTACTGAATTAGGTAATCCTGGATCTTTAATTG
GAGATGATCAAATTTATAATACTATTGTAACAGCTCATGCTTTTATTATAATTTTTTTTATAGTTATACCAATTATAATTGGAGGATTTGGAAATTG
ATTAATTCCTTTAATATTAGGGGCCCCAGATATAGCTTTTCCTCGAATAAATAATATAAGATTTTGATTATTACCCCCTTCATTAACTTTATTAATT
TCTAGAATAATTGTAGAAAATGGAGCAGGAACAGGATGAACAGTTTATCCCCCTTTATCATCTAATATCGCTCATAGAGGTAGTTCAGTTGATTTAG
CTATTTTTTCTTTACATTTAGCGGGAATTTCATCAATCTTAGGAGCTATTAATTTTATTACAACTATTATTAATATACGAATTAATCATATATCATT
TGATCAAATACCTCTCTTTGTATGAGCAGTAGGAATTACTGCTTTACTTTTATTACTATCTTTACCTGTATTAGCTGGTGCTATTACTATATTATTA
ACAGATCGAAATCTTAATACTTCATTTTTTGATCCTGCAGGAGGTGGAGATCCTATTTTATATCAACATTTA

LOWAM069_Russia_Teberda_2 ---------
TTTATTTTTGGTATTTGAGCAGGTATAGTAGGAACCTCTTTAAGATTATTAATTCGTACTGAATTAGGTAATCCTGGATCTTTAATTGGAGATGATC
AAATTTATAATACTATTGTAACAGCTCATGCTTTTATTATAATTTTTTTTATAGTTATACCAATTATAATTGGAGGATTTGGAAATTGATTAATTCC
TTTAATATTAGGAGCCCCAGATATAGCTTTTCCTCGAATAAATAATATAAGATTTTGATTACTGCCCCCCTCATTAACTTTATTAATTTCTAGAAGA
ATTGTAGAAAATGGAGCAGGAACAGGATGAACAGTTTATCCCCCTTTATCATCTAATATCGCTCATAGAGGTAGTTCAGTTGATTTAGCTATTTTTT
CTTTACATTTAGCAGGAATTTCATCAATCTTAGGAGCTATTAATTTTATTACAACTATTATTAATATACGAATTAATCATATATCATTTGATCAAAT
ACCTCTCTTTGTATGAGCAGTAGGAATTAC?GCTTTACTTTTATTATTATCTTTACCTGTATTAGCTGG?GCTATTACTATATTATTAACAGATCGA
AATCT?AATACTTCATTTTTTGATCCTGCAGGAGGAGGAGATCCTATTTTATATCAACATTTA

LOWAM073_Kyrgyzstan_Takhtalyk1
ACACTATATTTTATTTTTGGTATTTGAGCAGGTATAGTAGGAACCTCTTTAAGATTATTAATTCGTACTGAATTAGGTAATCCTGGATCTTTAATTG
GAGATGATCAAATTTATAATACTATTGTAACAGCTCATGCTTTTATTATAATTTTTTTTATAGTTATACCAATTATAATTGGAGGATTTGGAAATTG
ATTAATTCCTTTAATATTAGGGGCCCCAGATATAGCTTTTCCTCGAATAAATAATATAAGATTTTGATTATTACCCCCTTCATTAACTTTATTAATT
TCTAGAAGAATTGTAGAAAATGGAGCAGGAACAGGATGAACAGTTTATCCCCCTTTATCATCTAATATCGCTCATAGAGGTAGTTCAGTTGATTTAG
CTATTTTTTCTTTACATTTAGCGGGGATTTCATCAATCTTAGGAGCTATTAATTTTATTACAACTATTATTAATATACGAATTAATCATATATCATT
TGATCAAATACCTCTCTTTGTATGAGCAGTAGGAATTACTGCTTTACTTTTATTATTATCTTTACCTGTATTAGCTGGTGCTATTACTATATTATTA
ACAGATCGAAATCTTAATACTTCATTTTTTGATCCTGCAGGAGGTGGAGATCCTATTTTATATCAACATTTA

LOWAM074_Kyrgyzstan_Naryntoo
ACACTATATTTTATTTTTGGTATTTGAGCAGGTATAGTAGGAACCTCTTTAAGATTATTAATTCGTACTGAATTAGGTAATCCTGGATCTTTAATTG
GAGATGATCAAATTTATAATACTATTGTAACAGCTCATGCTTTTATTATAATTTTTTTTATAGTTATACCAATTATAATTGGAGGATTTGGAAATTG
ATTAATTCCTTTAATATTAGGGGCCCCAGATATAGCTTTTCCTCGAATAAATAATATAAGATTTTGATTATTACCCCCTTCATTAACTTTATTAATT
TCTAGAAGAATTGTAGAAAATGGAGCAGGAACAGGATGAACAGTTTATCCCCCTTTATCATCTAATATCGCTCATAGAGGTAGTTCAGTTGATTTAG
CTATTTTTTCTTTACATTTAGCGGGAATTTCATCAATCTTAGGAGCTATTAATTTTATTACAACTATTATTAATATACGAATTAATCATATATCATT
TGATCAAATACCTCTCTTTGTATGAGCAGTAGGAATTACTGCTTTACTTTTATTACTATCTTTACCTGTATTAGCTGGTGCTATTACTATATTATTA
ACAGATCGAAATCTTAATACTTCATTTTTTGATCCTGCAGGAGGTGGAGATCCTATTTTATATCAACATTTA

LOWAM086_Turkmenistan_3
ACATTATATTTTATTTTTGGTATTTGAGCAGGTATAGTAGGAACTTCCTTAAGATTATTAATTCGTACTGAATTAGGTAATCCTGGATCTTTAATTG
GAGATGATCAAATTTATAATACTATTGTAACAGCTCATGCTTTTATTATAATTTTTTTTATAGTTATACCAATTATAATTGGAGGATTTGGAAATTG
ATTAATTCCTTTAATATTAGGAGCCCCAGATATAGCTTTCCCCCGAATAAATAATATAAGATTTTGATTATTACCCCCCTCATTAACTTTATTAATT
TCTAGAAGAATTGTAGAAAATGGAGCAGGAACTGGATGAACAGTTTATCCCCCTTTATCATCTAATATTGCCCATAGAGGTAGTTCAGTTGATTTAG
CTATCTTTTCTTTACATTTAGCAGGAATTTCATCAATTTTAGGAGCTATTAATTTTATTACAACTATTATTAATATACGAATTAATCATATATCATT
TGATCAAATACCTCTTTTTGTATGAGCAGTAGGAATTACTGCTTTACTTTTATTACTATCTTTACCTGTATTAGCTGGTGCTATTACTATATTATTA
ACAGATCGAAATCTTAATACCTCATTTTTTGATCCAGCAGGAGGTGGAGATCCTATTTTATATCAACATTTA

LOWAM087_Uzbekistan_Sarchashma
ACACTATATTTTATTTTTGGTATTTGAGCAGGTATAGTAGGAACCTCTTTAAGATTATTAATTCGTACTGAATTAGGTAATCCTGGATCTTTAATTG
GAGATGATCAAATTTATAATACTATTGTAACAGCTCATGCTTTTATTATAATTTTTTTCATAGTTATACCAATTATAATTGGAGGATTTGGAAATTG
ATTAATTCCTTTAATATTAGGAGCCCCAGATATAGCTTTTCCTCGAATAAATAATATAAGATTTTGATTACTACCCCCTTCATTAACTTTATTAATT
TCCAGAAGAATTGTAGAAAATGGAGCAGGAACAGGATGAACAGTTTATCCCCCTTTATCATCTAATATCGCTCATAGAGGTAGTTCAGTTGATTTAG
CTATTTTTTCTTTACATTTAGCAGGAATTTCATCAATTTTAGGAGCTATTAATTTCATTACAACTATTATTAATATACGAATTAACCATATATCATT
TGATCAAATACCCCTCTTTGTATGAGCAGTAGGAATTACTGCTTTACTTTTATTATTATCTTTACCTGTATTAGCTGGTGCTATTACTATATTATTA
ACAGATCGAAATCTTAATACTTCATTTTTTGATCCTGCAGGAGGTGGAGATCCTATTTA??ATCAACATTTA

LOWAM095_Kyrgyzstan_Sosnovka
ACACTATATTTTATTTTTGGTATTTGAGCAGGTATAGTAGGAACCTCTTTAAGATTATTAATTCGTACTGAATTAGGTAATCCTGGATCTTTAATTG
GAGATGATCAAATTTATAATACTATTGTAACAGCTCATGCTTTTATTATAATTTTTTTTATAGTTATACCAATTATAATTGGAGGATTTGGAAATTG
ATTAATTCCTTTAATATTAGGGGCTCCAGATATAGCTTTTCCTCGAATAAATAATATAAGATTTTGATTATTACCCCCTTCATTAACTTTATTAATT
TCTAGAAGAATTGTAGAAAATGGAGCAGGAACAGGATGAACAGTTTATCCCCCTTTATCATCTAATATCGCTCATAGAGGTAGTTCAGTTGATTTAG
CTATTTTTTCTTTACATTTAGCGGGAATTTCATCAATCTTAGGAGCTATTAATTTTATTACAACTATTATTAATATACGAATTAATCATATATCATT
TGATCAAATACCTCTCTTTGTATGAGCAGTAGGAATTACTGCTTTACTTTTATTACTATCTTTACCTGTATTAGCTGGTGCTATTACTATATTATTA
ACAGATCGAAATCTTAATACTTCATTTTTTGATCCTGCAGGAGGTGGAGATCCTATTTTATATCAACATTTA

LOWAM096_Gissar_5
ACACTATATTTTATTTTTGGTATTTGAGCAGGTATAGTAGGAACCTCTTTAAGATTATTAATTCGTACTGAATTAGGTAATCCTGGATCTTTAATTG
GAGATGATCAAATTTATAATACTATTGTAACAGCTCATGCTTTTATTATAATTTTTTTCATAGTTATACCAATTATAATTGGAGGATTTGGAAATTG
ATTAATTCCTTTAATATTAGGAGCCCCAGATATAGCTTTTCCTCGAATAAATAATATAAGATTTTGATTACTACCCCCTTCATTAACTTTATTAATT
TCCAGAAGAATTGTAGAAAATGGAGCAGGAACAGGATGAACAGTTTATCCCCCTTTATCGTCTAATATCGCTCATAGAGGTAGTTCAGTTGATTTAG
CTATTTTTTCTTTACATTTAGCAGGAATTTCATCAATTTTAGGAGCTATTAATTTCATTACAACTATTATTAATATACGAATTAACCATATATCATT
TGATCAAATACCCCTCTTTGTATGAGCAGTAGGAATTACTGCTTTACTTTTATTATTATCTTTACCTGTATTAGCTGGTGCTATTACTATATTATTA
ACAGATCGAAATCTTAATACTTCATTTTTTGATCCTGCAGGAGGTGGAGATCCTATTTTATATCAACATTTA

LOWAM098_Tajikistan_Khondiza1
ACACTATATTTTATTTTTGGTATTTGAGCAGGTATAATAGGAACCTCTTTAAGATTATTAATTCGTACTGAATTAGGTAATCCTGGATCTTTAATTG
GAGATGATCAAATTTATAATACTATTGTAACAGCTCATGCTTTTATTATAATTTTTTTCATAGTTATACCAATTATAATTGGAGGATTTGGAAATTG
ATTAATTCCTTTAATATTAGGAGCCCCAGATATAGCTTTTCCTCGAATAAATAATATAAGATTTTGATTACTACCCCCTTCATTAACTTTATTAATT
TCCAGAAGAATTGTAGAAAATGGAGCAGGAACAGGATGAACAGTTTATCCCCCTTTATCGTCTAATATCGCTCATAGAGGTAGTTCAGTTGATTTAG
CTATTTTTTCTTTACATTTAGCAGGAATTTCATCAATTTTAGGAGCTATTAATTTCATTACAACTATTATTAATATACGAATTAACCATATATCATT
TGATCAAATACCCCTCTTTGTATGAGCAGTAGGAATTACTGCTTTACTTTTATTATTATCTTTACCTGTATTAGCTGGTGCTATTACTATATTATTA
ACAGATCGAAATCTTAATACTTCATTTTTTGATCCTGCAGGAGGTGGAGATCCTATTTTATATCAACATTTA

LOWAM099_Uzbekistan_Kamchik_2
ACACTATATTTTATTTTTGGTATTTGAGCAGGTATAGTAGGAACCTCTTTAAGATTATTAATTCGTACTGAATTAGGTAATCCTGGATCTTTAATTG
GAGATGATCAAATTTATAATACTATTGTAACAGCTCATGCTTTTATTATAATTTTTTTTATAGTTATACCAATTATAATTGGAGGATTTGGAAATTG
ATTAATTCCTTTAATATTAGGGGCCCCAGATATAGCTTTTCCTCGAATAAATAATATAAGATTTTGATTATTACCCCCTTCATTAACTTTATTAATT
TCTAGAAGAATTGTAGAAAATGGAGCAGGAACAGGATGAACAGTTTATCCCCCTTTATCATCTAATATCGCTCATAGAGGTAGTTCAGTTGATTTAG
CTATTTTTTCTTTACATTTAGCAGGAATTTCATCAATTTTAGGAGCTATTAATTTTATTACAACTATTATTAATATACGAATTAATCATATATCATT
TGATCAAATACCTCTCTTTGTATGAGCAGTAGGAATTACTGCTTTACTTTTATTACTATCTTTACCTGTATTAGCTGGTGCTATTACTATATTATTA
ACAGATCGAAATCTTAATACTTCATTTTTTGATCCTGCAGGAGGTGGAGATCCTATTTTATATCAACATTTA

LOWAM100_Kyrgyzstan_Konduk
ACACTATATTTTATTTTTGGTATTTGAGCAGGTATAGTAGGAACCTCTTTAAGATTATTAATTCGTACTGAATTAGGTAATCCTGGATCTTTAATTG
GAGATGATCAAATTTATAATACTATTGTAACAGCTCATGCTTTTATTATAATTTTTTTTATAGTTATACCAATTATAATTGGAGGATTTGGAAATTG
ATTAATTCCTTTAATATTAGGGGCCCCAGATATAGCTTTTCCTCGAATAAATAATATAAGATTTTGATTATTACCCCCTTCATTAACTTTATTAATT
TCTAGAAGAATTGTAGAAAATGGAGCAGGAACAGGATGAACAGTTTATCCCCCTTTATCATCTAATATCGCTCATAGAGGTAGTTCAGTTGATTTAG
CTATTTTTTCTTTACATTTAGCGGGAATTTCATCAATCTTAGGAGCTATTAATTTTATTACAACTATTATTAATATACGAATTAATCATATATCATT
TGATCAAATACCTCTCTTTGTATGAGCAGTAGGAATTACTGCTTTACTTTTATTACTATCTTTACCTGTATTAGCTGGTGCTATTACTATATTATTA
ACAGATCGAAATCTTAATACTTCATTTTTTGATCCTGCAGGAGGTGGAGATCCTATTTTATATCAACATTTA

LOWAM115_Azerbaijan_Nyus_Nyus
ACACTATATTTTATTTTCGGTATTTGAGCAGGTATAGTAGGAACCTCTTTAAGATTATTAATTCGTACTGAATTAGGTAATCCCGGATCTTTAATTG
GAGATGATCAAATTTATAACACTATTGTAACAGCTCATGCTTTTATTATAATTTTTTTTATAGTTATACCAATTATAATTGGAGGATTTGGAAATTG
ATTAATTCCTTTAATATTAGGAGCTCCAGATATAGCTTTTCCTCGAATAAATAATATAAGATTTTGATTACTCCCCCCCTCATTAACTTTATTAATT
TCTAGAAGAATTGTAGAAAATGGAGCAGGAACAGGATGAACAATTTATCCCCCTTTATCATCTAATATTGCTCATAGAGGTAGTTCAGTTGATTTAG
CTATTTTTTCTTTACATTTAGCAGGAATTTCATCAATCTTAGGAGCTATTAATTTTATTACAACTATTATTAACATACGAATTAATCATATATCATT
TGATCAAATACCCCTTTTTGTATGAGCAGTAGGAATTACTGCATTACTTTTATTATTATCTTTACCTGTATTAGCTGGTGCTATTACTATATTATTA
ACAGATCGAAATCTTAATACTTCATTTTTTGATCCTGCAGGAGGTGGAGATCCTATTTTATATCAACACTTA

LOWAM116_Iran_Dizin_2
ACACTATATTTTATTTTCGGTATTTGAGCAGGTATAGTAGGAACCTCTTTAAGATTATTAATTCGTACTGAATTAGGTAATCCTGGATCTTTAATTG
GAGATGATCAAATTTATAACACTATTGTAACAGCTCATGCTTTTATTATAATTTTTTTTATAGTTATACCAATTATAATTGGAGGATTTGGAAATTG
ATTAATTCCTTTAATATTAGGAGCTCCAGATATAGCTTTTCCTCGAATAAATAATATAAGATTTTGATTACTCCCCCCCTCATTAACTTTATTAATT
TCTAGAAGAATTGTAGAAAATGGAGCAGGAACAGGATGAACAATTTATCCCCCTTTATCATCTAATATTGCTCATAGAGGTAGTTCAGTTGATTTAG
CTATTTTTTCTTTACATTTAGCAGGAATTTCATCAATCTTAGGAGCTATTAATTTTATTACAACTATTATTAACATACGAATTAATCATATATCATT
TGATCAAATACCCCTTTTTGTATGAGCAGTAGGAATTACTGCATTACTTTTATTATTATCTTTACCTGTATTAGCTGGTGCTATTACTATATTATTA
ACAGATCGAAATCTTAATACTTCATTTTTTGATCCTGCAGGAGGTGGAGATCCTATTTTATATCAACACTTA

LOWAM122_Russia_Saratow_1
ACACTATATTTTATTTTTGGTATTTGAGCAGGTATAGTAGGAACCTCTTTAAGATTATTAATTCGTACTGAATTAGGTAATCCTGGATCTTTAATTG
GAGATGATCAAATTTATAACACTATTGTAACAGCTCATGCTTTTATTATAATTTTTTTTATAGTTATACCAATTATAATTGGAGGATTTGGAAATTG
ATTAATTCCTTTAATATTAGGAGCCCCAGATATAGCTTTTCCTCGAATAAATAATATAAGATTTTGATTACTACCCCCCTCATTAACTTTATTAATT
TCTAGAAGAATTGTAGAAAATGGAGCAGGAACAGGATGAACAGTTTATCCCCCTTTATCATCTAATATCGCTCATAGAGGTAGTTCAGTTGATTTAG
CTATTTTTTCTTTACATTTAGCAGGAATTTCATCAATCTTAGGAGCTATTAATTTTATTACAACTATTATTAATATACGAATTAACCATATATCATT

TGATCAAATACCTCTCTTTGTATGAGCAGTAGGAATTACTGCTTTACTTTTATTATTATCTTTACCTGTATTAGCTGGTGCTATTACTATATTATTA
ACAGATCGAAATCTTAATACTTCATTTTTTGATCCTGCAGGAGGTGGGGATCCTATTTTATATCAACATTTA

LOWAM136_Uzbekistan_Tamshush_1
ACACTATATTTTATTTTTGGTATTTGAGCAGGTATAGTAGGAACCTCTTTAAGATTATTAATTCGTACTGAATTAGGTAATCCTGGATCTTTAATTG
GAGATGATCAAATTTATAATACTATTGTAACAGCTCATGCTTTTATTATAATTTTTTTCATAGTTATACCAATTATAATTGGAGGATTTGGAAATTG
ATTAATTCCTTTAATATTAGGAGCCCCAGATATAGCTTTTCCTCGAATAAATAATATAAGATTTTGATTACTACCCCCTTCATTAACTTTATTAATT
TCCAGAAGAATTGTAGAAAATGGAGCAGGAACAGGATGAACAGTTTATCCCCCTTTATCATCTAATATCGCTCATAGAGGTAGTTCAGTTGATTTAG
CTATTTTTTCTTTACATTTAGCAGGAATTTCATCAATTTTAGGAGCTATTAATTTCATTACAACTATTATTAATATACGAATTAACCATATATCATT
TGATCAAATACCCCTCTTTGTATGAGCAATAGGAATTACTGCTTTACTTTTATTATTATCTTTACCTGTATTAGCTGGTGCTATTACTATATTATTA
ACAGATCGAAATCTTAATACTTCATTTTTTGATCCTGCAGGAGGTGGAGATCCTATTTTATATCAACATTTA

LOWAM149_Kyrgyzstan_Songkel_1
ACACTATATTTTATTTTTGGTATTTGAGCAGGTATAGTAGGAACCTCTTTAAGATTATTAATTCGTACTGAATTAGGTAATCCTGGATCTTTAATTG
GAGATGATCAAATTTATAATACTATTGTAACAGCTCATGCTTTTATTATAATTTTTTTATAGTTATACCAATTATAATTGGAGGATTTGGAAATTG
ATTAATTCCTTTAATATTAGGGGCCCCAGATATAGCTTTTCCTCGAATAAATAATATAAGATTTTGATTATTACCCCCTTCATTAACTTTATTAATT
TCTAGAAGAATTGTAGAAAATGGAGCAGGAACAGGATGAACAGTTTATCCCCCTTTATCATCTAATATTGCTCATAGAGGTAGTTCAGTTGATTTAG
CTATTTTTTCTTTACATTTAGCGGGAATTTCATCAATCTTAGGAGCTATTAATTTTATTACAACTATTATTAATATACGAATTAATCATATATCATT
TGATCAAATACCTCTCTTTGTATGAGCAGTAGGAATTACTGCTTTACTTTTATTACTATCTTTACCTGTATTAGCTGGTGCTATTACTATATTATTA
ACAGATCGAAATCTTAATACTTCATTTTTTGATCCTGCAGGAGGTGGAGATCCTATTTTATATCAACATTTA

LOWAM198_Turkey_Aladaglar_2
ACACTATATTTTATTTTTGGTATTTGAGCAGGTATAGTAGGAACCTCTTTAAGATTATTAATTCGTACTGAATTAGGTAATCCTGGATCTTTAATTA
GAGATGATCAAATTTATAATACTATTGTAACAGCTCATGCTTTTATTATAATTTTTTTATAGTTATACCAATTATAATTGGAGGATTTGGAAATTG
ATTAATTCCTTTAATATTAGGAGCTCCAGATATAGCTTTTCCTCGAATAAATAATATAAGATTTTGATTACTTCCCCCTTCATTAACTTTATTAATT
TCTAGAAGAATTGTAGAAAATGGAGCAGGAACAGGATGAACAGTTTATCCCCCTTTATCATCTAATATTGCTCATAGAGGTAGTTCAGTTGATTTAG
CTATTTTTTCTTTACATTTAGCAGGAATTTCATCAATCTTAGGAGCTATTAATTTTATTACAACTATTATTAATATA--------------------
----

LOWAM213_Tajikistan_Tandukul_1
ACACTATATTTTATTTTTGGTATTTGAGCAGGTATAGTAGGAACCTCTTTAAGATTATTAATTCGTACTGAATTAGGTAATCCTGGATCTTTAATTG
GAGATGATCAAATTTATAATACTATTGTAACAGCTCATGCTTTTATTATAATTTTTTTCATAGTTATACCAATTATAATTGGAGGATTTGGAAATTG
ATTAATTCCTTTAATATTAGGAGCCCCAGATATAGCTTTTTCTCGAATAAATAATATAAGATTTTGATTACTACCCCCTTCATTAACTTTATTAATT
TCTAGAAGAATTGTAGAAAATGGAGCAGGAACAGGATGAACAGTTTATCCCCCTTTATTATCTAATATCGCTCATAGAGGTAGTTCAGTTGATTTAG
CTATTTTTTCTTTACATTTAGCAGGAATTTCATCAATTTTAGGAGCTATTAATTTCATTACAACTATTATTAATATACGAATTAACCATATATCATT
TGATCAAATACCCCTCTTTGTATGAGCAGTAGGAATTACTGCGTTACTTTTATTATTATCTTTACCTGTATTAGCTGGTGCTATTACTATATTATTA
ACAGATCGAAATCTTAATACTTCATTTTTTGATCCTGCAGGAGGTGGAGATCCTATTTTATATCAACATTTA

Parnassius_clodius
ACATTATATTTTATTTTTGGTATTTGAGCAGGTATAGTAGGAACCTCTTTAAGATTATTAATTCGTACTGAATTAGGTAATCCTGGATCTTTAATTG
GAGATGATCAAATTTATAATACTATTGTAACAGCTCATGCTTTTATCATAATTTTTTTCATAGTTATACCAATTATAATTGGAGGATTTGGAAATTG
ATTAATTCCATTAATATTAGGAGCTCCAGATATAGCTTTTCCTCGAATAAATAATATAAGATTTTGATTATTACCCCCTTCATTAACTTTATTAATT
TCTAGAAGAATTGTACAAAATGGAGCAGGAACTGGATGAACAGTTTATCCCCCTTTATCATCTAATATTGCTCATAGAGGAAGATCAGTTGATTTAG
CTATTTTTTCTTTACATTTAGCTGGAATTTCATCTATCTTAGGAGCTATTAATTTTATTACAACTATTATTAATATACGAATTAATCATATATCATT
TGATCAAATACCCCTTTTTGTATGAGCAGTAGGAATTACCGCTTTACTTCTATTATTATCTTTACCTGTTTTAGCAGGTGCTATTACCATATTATTA
ACAGATCGAAATCTTAATACTTCATTTTTTGATCCAGCAGGAGGTGGAGATCCTATTTTATATCAACACTTA

Parnassius_eversmanni_2005_LOWA_108 ---------------------
ATTTGAGCAGGTATAGTAGGAACCTCTTTAAGATTATTAATTCGTTCTGAATTAGGTAATCCTGGATCTTTAATTGGAGATGATCAAATTTATAATA
CTATTGTAACAGCTCATGCTTTTATTATAATTTTTTTTATAGTTATACCAATTATAATTGGAGGATTTGGAAATTGATTAATTCCATTAATATTAGG
AGCTCCAGATATAGCTTTTCCTCGAATAAATAATATAAGATTTTGATTATTACCCCCCTCATTAACCTTACTAATTTCTAGAAGAATTGTAGAAAAT
GGAGCAGGAACTGGATGAACGGTTTATCCCCCTTTATCATCTAATATTGCTCATAGAGGAAGATCAGTTGATTTAGCTATCTTTTCTTTACATTTAG
CTGGAATTTCATCTATCTTAGGAGCTATTAATTTTATTACAACTATTATTAATATACGAATTAATCATATATCATTTGATCAAATACCCCTTTTTGT
ATGAGCAGTAGGAATTACTGCTTTACTTTTATTATTATCTTTACCTGTTTTAGCAGGTGCTATTACCATATTATTAACAGATCGAAATCTTAATACT
TCATTTTTTGACCCAGCAGGAGGTGGAGATCCTATTTTATATCAACATTTA

Parnassius_eversmanni_2005_LOWA_107 ---------------------
ATTTGAGCAGGTATAGTAGGAACCTCTTTAAGATTATTAATTCGTTCTGAATTAGGTAATCCTGGATCTTTAATTGGAGATGATCAAATTTATAATA
CTATTGTAACAGCTCATGCTTTTATTATAATTTTTTTTATAGTTATACCAATTATAATTGGAGGATTTGGAAATTGATTAATTCCATTAATATTAGG
AGCTCCAGATATAGCTTTTCCTCGAATAAATAATATAAGATTTTGATTATTACCCCCCTCATTAACCTTACTAATTTCTAGAAGAATTGTAGAAAAT
GGAGCAGGAACTGGATGAACGGTTTATCCCCCTTTATCATCTAATATTGCTCATAGAGGAAGATCAGTTGATTTAGCTATCTTTTCTTTACATTTAG
CTGGAATTTCATCTATCTTAGGAGCTATTAATTTTATTACAACTATTATTAATATACGAATTAATCATATATCATTTGATCAAATACCCCTTTTTGT
ATGAGCAGTAGGAATTACTGCTTTACTTTTATTATTATCTTTACCTGTTTTAGCAGGTGCTATTACCATATTATTAACAGATCGAAATCTTAATACT
TCATTTTTTGACCCAGCAGGAGGTGGAGATCCTATTTTATATCAACATTTA

Parnassius_stubbendorfii_2005_LOWA_815
ACATTATATTTTATTTTTGGTATTTGAGCAGGTATAGTAGGAACTTCTTTAAGATTATTAATTCGTACTGAATTAGGTAATCCTGGATCTTTAATTG
GAGATGATCAAATTTATAATACTATTGTAACAGCTCATGCTTTTATTATAATTTTTTTTATAGTTATACCAATTATAATTGGAGGATTTGGAAATTG
ATTAATTCCTTTAATATTAGGAGCCCCAGATATAGCTTTTCCTCGAATAAATAATATAAGATTTTGATTATTACCCCCTTCATTAACTTTACTAATT
TCCAGAAGAATTGTAGAAAATGGGGCAGGAACTGGATGAACAGTCTACCCTCCTTTATCATCTAATATTGCTCACGGAGGAAGATCTGTTGATTTAG
CTATTTTTTCCTTACATTTAGCGGGAATTTCATCTATTTTAGGAGCCATTAATTTTATTACAACTATTATTAATATACGAATTAATCATATATCATT
TGATCAAATACCTCTTTTTGTATGAGCAGTAGGAATTACTGCTTTACTTTTATTATTATCTCTACCTGTTTTAGCAGGTGCTATTACTATATTATTA
ACAGATCGAAATCTTAATACTTCATTTTTTGACCCAGCAGGAGGTGGAGATCCTATTTTATATCAACACTTA

Parnassius_stubbendorfii_2005_LOWA_154
ACATTATATTTTATTTTTGGTATTTGAGCAGGTATAGTAGGAACTTCTTTAAGATTATTAATTCGTACTGAATTAGGTAATCCTGGATCTTTAATTG
GAGATGATCAAATTTATAATACTATTGTAACAGCTCATGCTTTTATTATAATTTTTTTTATAGTTATACCAATTATAATTGGAGGATTTGGAAATTG
ATTAATTCCTTTAATATTAGGAGCCCCAGATATAGCTTTTCCTCGAATAAATAATATAAGATTTTGATTATTACCCCCTTCATTAACTTTACTAATT
TCCAGAAGAATTGTAGAAAATGGAGCAGGAACTGGATGAACAGTCTACCCTCCTTTATCATCTAATATTGCTCACGGAGGAAGATCTGTTGATTTAG

CTATTTTTTCCTTACATTTAGCGGGAATTTCATCTATTTTAGGAGCCATTAATTTTATTACAACTATTATTAATATACGAATTAATCATATATCATT
TGATCAAATACCTCTTTTTGTATGAGCAGTAGGAATTACTGCTTTACTTTTATTATTATCTCTACCTGTTTTAGCAGGTGCTATTACTATATTATTA
ACAGATCGAAATCTTAATACTTCATTTTTTGACCCAGCAGGAGGTGGAGATCCTATTTTATATCAACACTTA

Parnassius_stubbendorfii_2005_LOWA_153 -

CATTATATTTTATTTTTGGTATTTGAGCAGGTATAGTAGGAACTTCTTTAAGATTATTAATTCGTACTGAATTAGGTAATCCTGGATCTTTAATTGG
AGATGATCAAATTTATAATACTATTGTAACAGCTCATGCTTTTATTATAATTTTTTTTATAGTTATACCAATTATAATTGGAGGATTTGGAAATTGA
TTAATTCCTTTAATATTAGGAGCCCCAGATATAGCTTTTCCTCGAATAAATAATATAAGATTTTGATTATTACCCCCTTCATTAACTTTACTAATTT
CCAGAAGAATTGTAGAAAATGGAGCAGGAACTGGATGAACAGTCTACCCTCCTTTATCATCTAATATTGCTCACGGAGGAAGATCTGTTGATTTAGC
TATTTTTTCCTTACATTTAGCGGGAATTTCATCTATTTTAGGAGCCATTAATTTTATTACAACTATTATTAATATACGAATTAATCATATATCATTT
GATCAAATACCTCTTTTTGTATGAGCAGTAGGAATTACTGCTTTACTTTTATTATTATCTCTACCTGTTTTAGCAGGTGCTATTACTATATTATTAA
CAGATCGAAATCTTAATACTTCATTTTTTGACCCAGCAGGAGGTGGAGATCCTATTTTATATCAACACTTA

Parnassius_nordmanni

ACATTATATTTTATTTTTGGTATTTGAGCAGGTATAGTAGGAACTTCTTTAAGATTATTAATTCGTACTGAATTAGGTAATCCTGGATCTCTAATTG
GAGATGATCAAATTTACAATACTATCGTAACAGCTCATGCTTTTATTATAATTTTTTTCATAGTTATACCAATTATAATTGGAGGATTTGGAAATTG
ATTAATTCCATTAATATTAGGAGCTCCAGATATAGCTTTCCCCCGAATAAATAATATAAGATTTTGATTATTACCCCCCTCATTAACTCTATTAATT
TCTAGAAGAATTGTAGAAAATGGGGCAGGAACTGGATGAACAGTTTACCCCCCTTTATCATCTAATATTGCTCATAGAGGAAGATCAGTTGACTTAG
CTATTTTTTCTTTACATTTGGCTGGGATTTCTTCTATTTTAGGAGCTATTAATTTTATCACAACTATTGTTAATATACGAATTAACTATATATCATT
TGATCAAATACCTCTTTTCGTATGAGCAGTAGGAATTACTGCTTTACTTTTATTATTATCTTTACCTGTTTTAGCAGGTGCTATTACTATATTATTA
ACAGATCGAAATCTTAATACTTCATTTTTTGATCCAGCAGGAGG?GGAGACCCTATTCTATATCAA------

Parnassius_eversmanni

ACATTATATTTTATTTTTGGTATTTGAGCAGGTATAGTAGGAACCTCTTTAAGATTATTAATTCGTTCTGAATTAGGTAATCCTGGATCTTTAATTG
GAGATGATCAAATTTATAATACTATTGTAACAGCTCATGCTTTTATTATAATTTTTTTTATAGTTATACCAATTATAATTGGAGGATTTGGAAATTG
ATTAATTCCATTAATATTAGGAGCTCCAGATATAGCTTTTCCTCGAATAAATAATATAAGATTTTGATTATTACCCCCCTCATTAACCTTACTAATT
TCTAGAAGAATTGTAGAAAATGGAGCAGGAACTGGATGAACGGTTTATCCCCCTTTATCATCTAATATTGCTCATAGAGGAAGATCAGTTGATTTAG
CTATCTTTTCTTTACATTTAGCTGGAATTTCATCTATCTTAGGAGCTATTAATTTTATTACAACTATTATTAATATACGAATTAATCATATATCATT
TGATCAAATACCCCTTTTTGTATGAGCAGTAGGAATTACTGCTTTACTTTTATTATTATCTTTACCTGTTTTAGCAGGTGCTATTACCATATTATTA
ACAGATCGAAATCTTAATACTTCATTTTTTGACCCAGCAGGAGGTGGAGATCCTATTTTATATCAA------

Parnassius_eversmanni_felderi

ACATTATATTTTATTTTTGGTATTTGAGCAGGTATAGTAGGAACCTCTTTAAGATTATTAATTCGTTCTGAATTAGGTAATCCTGGATCTTTAATTG
GAGATGATCAAATTTATAATACTATTGTAACAGCTCATGCTTTTATTATAATTTTTTTTATAGTTATACCAATTATAATTGGAGGATTTGGAAATTG
ATTAATTCCATTAATATTAGGAGCTCCAGATATAGCTTTTCCTCGAATAAATAATATAAGATTTTGATTATTACCCCCCTCATTAACCTTACTAATT
TCTAGAAGAATTGTAGAAAATGGAGCAGGAACTGGATGAACGGTTTATCCCCCTTTATCATCTAATATTGCTCATAGAGGAAGATCAGTTGATTTAG
CTATCTTTTCTTTACA?TTAGCTGGAATTTCATCTATCTTAGGAGCTATTAATTTTATTACAACTATTATTAATATACGAATTAATCATATATCATT
TGATCAAATACCCCTTTTTGTATGAGCAGTAGGAATTACTGCTTTACTTTTATTATTATCTTTACCTGTTTTAGCAGGTGCTATTACCATATTATTA
ACAGATCGAAATCTTAATACTTCATTTTTTGACCCAGCAGGAGGTGGAGATCCTATTTTATATCAA------

Parnassius_ariadne_ariadne

ACATTATATTTTATTTTTGGTATTTGAGCAGGTATAGTAGGAACCTCTTTAAGATTATTAATTCGTACTGAATTAGGTAATCCTGGATCTTTAATTG
GAGATGATCAAATTTATAATACTATCGTAACAGCTCATGCTTTTATTATAATTTTTTTTATAGTTATACCAATTATAATTGGAGGATTTGGAAATTG
ATTAATTCCTTTAATATTAGGAGCTCCAGATATAGCCTTTCCTCGAATAAATAATATAAGATTTTGATTACTACCCCCCTCATTAACTTTATTAATC
TCTAGAAGAATTGTAGAAAATGGAGCAGGAACTGGATGAACAGTTTATCCCCCTTTATCATCTAATATTGCTCATAGAGGAAGTTCAGTTGATTTAG
CCATTTTCTCTTTACATTTAGCAGGAATTTCATCAATTTTAGGAGCTATTAATTTTATCACAACTATTATTAATATACGAATTAATCATATATCATT
TGATCAAATACCCCTTTTTGTTTGAGCAGTAGGAATTACTGCTTTACTATTATTATTATCTTTACCTGTATTAGCTGGTGCTATTACTATATTATTA
ACAGATCGAAATCTTAATACTTCATTTTTTGATCCAGCAGGA????GAGATCCTATTTTATATCAA------

Parnassius_glacialis_mikado

ACATTATATTTTATTTTTGGTATTTGAGCAGGTATAGTAGGAACCTCCTTAAGATTATTAATTCGTACTGAATTAGGTAATCCTGGATCTTTAATTG
GAGATGATCAAATTTATAATACTATTGTAACAGCTCATGCTTTTATTATAATTTTTTTTATAGTTATACCAATTATAATTGGAGGATTTGGAAATTG
ATTAATCCCTTTAATATTAGGAGCTCCAGATATAGCTTTCCCCCGAATAAATAATATAAGATTTTGATTATTACCCCCCTCATTAACTTTACTAATT
TCCAGAAGAATTGTAGAAAATGGAGCAGGAACAGGATGAACAGTTTATCCCCCATTATCCTCTAATATTGCCCACAGAGGAAGATCTGTTGATTTAG
CTATTTTTTCTTTACATTTAGCAGGAATTTCATCTATTCTAGGAGCTATTAATTTTATTACAACTATTATTAATATACGAATTAATCATATATCATT
TGATCAAATACCTCTCTTTGTTTGAGCAGTAGGAATTACTGCTTTACTTTTATTATTATCCCTACCTGTTTTAGCAGGTGCTATTACTATATTATTA
ACAGATCGAAATCTTAATACTTCTTTTTTTGACCCAGCAGGAGGTGGAGATCCAATTTTATATCAA------

Parnassius_stubbendorfii_hoenei

ACATTATATTTTATTTTTGGTATTTGAGCAGGAATAGTAGGAACCTCCCTAAGATTATTAATTCGTACTGAATTAGGTAATCCCGGATCTTTAATTG
GAGATGATCAAATTTACAATACTATTGTAACAGCTCATGCTTTCATTATAATTTTTTTTATAGTTATACCAATTATAATTGGAGGATTTGGAAATTG
ACTGATTCCTTTAATATTAGGAGCCCCAGATATAGCTTTCCCCCGAATAAATAATATAAGATTTTGATTACTACCCCCCTCATTAACTTTATTAATT
TCTAGAAGAATTGTAGAAAATGGAGCAGGAACTGGATGAACAGTCTATCCCCCTTTATCATCTAATATTGCCCATAGAGGAAGATCCGTTGATTTAG
CTATTTTTTCCTTACATTTAGCAGGAATTTCATCTATTTTAGGAGCCATTAATTTTATTACAACTATTATTAATATACGAATTAATCATATATCATT
TGATCAAATACCTCTTTTTGTATGAGCAGTAGGAATTACTGCTTTACTTTTATTATTATCTTTACCTGTTTTAGCAGGTGCTATTACTATATTATTA
ACAGATCGAAATCTTAATACTTCCTTTTTTGACCCAGCAGGAGGTGGAGATCCTATTTTATACCAA------

Parnassius_stubbendorfii_hoenei_AC20_16 ------------------------

TGAGCAGGAATAGTAGGAACCTCCCTAAGATTATTAATTCGTACTGAATTAGGTAATCCCGGATCTTTAATTGGAGATGATCAAATTTACAATACTA
TTGTAACAGCTCATGCTTTCATTATAATTTTTTTTATAGTTATACCAATTATAATTGGAGGATTTGGAAATTGACTGATTCCTTTAATATTAGGAGC
CCCAGATATAGCTTTCCCCCGAATAAATAATATAAGATTTTGATTACTACCCCCCTCATTAACTTTATTAATTTCTAGAAGAATTGTAGAAAATGGA
GCAGGAACTGGATGAACAGTCTATCCCCCTTTATCATCTAATATTGCCCATAGAGGAAGATCCGTTGATTTAGCTATTTTTTCCTTACATTTAGCAG
GAATTTCATCTATTTTAGGAGCCATTAATTTTATTACAACTATTATTAATATACGAATTAATCATATATCATTTGATCAAATACCTCTTTTTGTATG
AGCAGTAGGAATTACTGCTTTACTTTTATTATTATCTTTACCTGTTTTAGCAGGTGCTATTACTATATTATTAACAGATCGAAATCTTAATACTTCC
TTTTTTGACCCCGCAGGAGGTGGAGATCCTATTTTATACCAACATTTA

Parnassius_stubbendorfii_koreanus ------------------------

TGAGCAGGTATAGTAGGAACTTCTTTAAGATTATTAATTCGTACTGAATTAGGTAATCCTGGATCTTTAATTGGAGATGATCAAATTTATAATACTA
TTGTAACAGCTCATGCTTTTATTATAATTTTTTTTATAGTTATACCAATTATAATTGGAGGATTTGGAAATTGATTAATTCCTTTAATATTAGGAGC
CCCAGATATAGCTTTTCCTCGAATAAATAATATAAGATTTTGACTATTACCCCCCTCATTAACTTTACTAATTTCCAGAAGAATTGTAGAAAATGGA

GCAGGAACTGGATGAACAGTCTACCCTCCTTTATCATCTAATATTGCTCACGGAGGAAGATCTGTTGATTTAGCTATTTTTTCCTTACATTTAGCAG
GAATTTCATCTATTTTAGGAGCCATTAATTTTATTACAACTATTATTAATATACGAATTAATCATATATCATTTGATCAAATACCTCTTTTTGTATG
AGCAGTAGGAATTACTGCTTTACTTTTATTATTATCTCTACCTGTTTTAGCAGGTGCTATTACTATATTATTAACAGATCGAAATCTTAATACTTCA
TTTTTTGACCCAGCAGGAGGTGGAGATCCTATTTTATATCAACACTTA

Parnassius_nordmanni_AC20_5 ------------------------
TGAGCAGGTATAGTAGGAACTTCTTTAAGATTATTAATTCGTACTGAATTAGGTAATCCTGGATCTCTAATTGGAGATGATCAAATTTACAATACTA
TCGTAACAGCTCATGCTTTTATTATAATTTTTTTCATAGTTATACCAATTATAATTGGAGGATTTGGAAATTGATTAATTCCATTAATATTAGGAGC
TCCAGATATAGCTTTCCCCCGAATAAATAATATAAGATTTTGATTATTACCCCCCTCATTAACTCTATTAATTTCTAGAAGAATTGTAGAAAATGGG
GCAGGAACTGGATGAACAGTTTACCCCCCTTTATCATCTAATATTGCTCATAGAGGAAGATCAGTTGACTTAGCTATTTTTTCTTTACATTTGGCTG
GGATTTCTTCTATTTTAGGAGCTATTAATTTTATCACAACTATTGTTAATATACGAATTAACTATATATCATTTGATCAAATACCTCTTTTCGTATG
AGCAGTAGGAATTACTGCTTTACTTTTATTATTATCTTTACCTGTTTTAGCAGGTGCTATTACTATATTATTAACAGATCGAAATCTTAATACTTCA
TTTTTTGATCCAGCAGGAGGTGGAGACCCTATTCTATATCAACATTTA

Parnassius_eversmanni_felderi_AC23_68 ------------------------
TGAGCAGGTATAGTAGGAACCTCTTTAAGATTATTAATTCGTTCTGAATTAGGTAATCCTGGATCTTTAATTGGAGATGATCAAATTTATAATACTA
TTGTAACAGCTCATGCTTTTATTATAATTTTTTTTATAGTTATACCAATTATAATTGGAGGATTTGGAAATTGATTAATTCCATTAATATTAGGAGC
TCCAGATATAGCTTTTCCTCGAATAAATAATATAAGATTTTGATTATTACCCCCCTCATTAACCTTACTAATTTCTAGAAGAATTGTAGAAAATGGA
GCAGGAACTGGATGAACGGTTTATCCCCCTTTATCATCTAATATTGCTCATAGAGGAAGATCAGTTGATTTAGCTATCTTTTCTTTACATTTAGCTG
GAATTTCATCTATCTTAGGAGCTATTAATTTTATTACAACTATTATTAATATACGAATTAATCATATATCATTTGATCAAATACCCCTTTTTGTATG
AGCAGTAGGAATTACTGCTTTACTTTTATTATTATCTTTACCTGTTTTAGCAGGTGCTATTACCATATTATTAACAGATCGAAATCTTAATACTTCA
TTTTTTGACCCAGCAGGAGGTGGAGATCCTATTTTATTTCAACATTTA

Parnassius_eversmanni_eversmanni_AC1_14 ------------------------
TGATCAGGTATAGTAGGAACCTCTTTAAGATTATTAATTCGTTCTGAATTAGGTAATCCTGGATCTTTAATTGGAGATGATCAAATTTATAATACTA
TTGTAACAGCTCATGCTTTTATTATAATTTTTTTTATAGTTATACCAATTATAATTGGAGGATTTGGAAATTGATTAATTCCATTAATATTAGGAGC
TCCAGATATAGCTTTTCCTCGAATAAATAATATAAGATTTTGATTATTACCCCCCTCATTAACCTTACTAATTTCTAGAAGAATTGTAGAAAATGGA
GCAGGAACTGGATGAACGGTTTATCCCCCTTTATCATCTAATATTGCTCATAGAGGAAGATCAGTTGATTTAGCTATCTTTTCTTTACATTTAGCTG
GAATTTCATCTATCTTAGGAGCTATTAATTTTATTACAACTATTATTAATATACGAATTAATCATATATCATTTGATCAAATACCCCTTTTTGTATG
AGCAGTAGGAATTACTGCTTTACTTTTATTATTATCTTTACCTGTTTTAGCAGGTGCTATTACCATATTATTAACAGATCGAAATCTCAATACTTCA
TTTTTTGACCCAGCAGGAGGTGGAGATCCTATTTTATATCAACATTTA

Parnassius_clodius_AC4_5 ------------------------
TGAGCAGGAATATTAGGAACCTCTTTAAGATTATTAATTCGTACTGAATTAGGTAATCCTGGATCTTTAATTGGAGATGATCAAATTTATAATACTA
TTGTAACAGCTCATGCTTTTATCATAATTTTTTTCATAGTTATACCAATTATAATTGGAGGATTTGGAAATTGATTAATTCCATTAATATTAGGAGC
TCCAGATATAGCTTTTCCTCGAATAAATAATATAAGATTTTGATTATTACCCCCTTCATTAACTTTATTAATTTCTAGAAGAATTGTAGAAAATGGA
GCAGGAACTGGATGAACAGTTTATCCCCCTTTATCATCTAATATTGCTCATAGAGGAAGATCAGTTGATTTAGCTATTTTTTCTTTACATTTAGCTG
GAATTTCATCTATCTTAGGAGCTATTAATTTTATTACAACTATTATTAATATACGAATTAATCATATATCATTTGATCAAATACCCCTTTTTGTATG
AGCAGTAGGAATTACCGCTTTACTTCTATTATTATCTTTACCTGTTTTAGCAGGTGCTATTACCATATTATTAACAGATCGAAATCTTAATACTTCA
TTTTTTGATCCAGCAGGAGGTGGAGATCCTATTTTATATCAACACTTA

Parnassius_ariadne_AC4_14 ------------------------
TGAGCAGGTATAGTAGGAACCTCTTTAAGATTATTAATTCGTACTGAATTAGGTAATCCTGGATCTTTAATTGGAGATGATCAAATTTATAATACTA
TCGTAACAGCTCATGCTTTTATTATAATTTTTTTTATAGTTATACCAATTATAATTGGAGGATTTGGAAATTGATTAATTCCTTTAATATTAGGAGC
TCCAGATATAGCCTTTCCTCGAATAAATAATATAAGATTTTGATTACTACCCCCCTCATTAACTTTATTAATCTCTAGAAGAATTGTAGAAAATGGA
GCAGGAACTGGATGAACAGTTTATCCCCCTTTATCATCTAATATTGCTCATAGAGGAAGTTCAGTTGATTTAGCCATTTTCTCTTTACATTTAGCAG
GAATTTCATCAATTTTAGGAGCTATTAATTTTATCACAACTATTATTAATATACGAATTAATCATATATCATTTGATCAAATACCCCTTTTTGTTTG
AGCAGTAGGAATTACTGCTTTACTATTATTATTATCTTTACCTGTATTAGCTGGTGCTATTACTATATCATTAACAGATCGAAATCTTAATACTTCA
TTTTTTGATCCAGCAGGAGGTGGAGATCCTATTTTATATCAACACTTA

HQ004911_RV_07_C107_Romania
ACACTATATTTTATTTTTGGTATTTGAGCAGGTATAGTAGGAACCTCTTTAAGATTATTAATTCGTACTGAATTAGGTAATCCTGGATCTTTAATTG
GAGATGATCAAATTTATAATACTATTGTAACAGCTCATGCTTTTATTATAATTTTTTTTATAGTTATACCAATTATAATTGGAGGATTTGGAAATTG
ATTAATTCCTTTAATATTAGGAGCCCCAGATATAGCTTTTCCTCGAATAAATAATATAAGATTTTGATTACTACCCCCCTCATTAACTTTATTAATT
TCTAGAAGAATTGTAGAAAATGGAGCAGGAACAGGATGAACAGTTTATCCCCCTTTATCATCTAATATCGCTCATAGAGGTAGTTCAGTTGATTTAG
CTATTTTTTCTTTACATTTAGCAGGAATTTCATCAATCTTAGGAGCTATTAATTTTATTACAACTATTATTAATATACGAATTAACCATATATCATT
TGATCAAATACCTCTCTTTGTATGAGCAGTAGGAATTACTGCTTTACTTTTATTATTATCTTTACCTGTATTAGCTGGTGCTATTACTATATTATTA
ACAGATCGAAATCTTAATACTTCATTTTTTGATCCTGCAGGAGGTGGAGATCCTATTTTATATCAACATTTA

HQ004908_RV_07_D060_Romania
ACACTATATTTTATTTTTGGTATTTGAGCAGGTATAGTAGGAACCTCTTTAAGATTATTAATTCGTACTGAATTAGGTAATCCTGGATCTTTAATTG
GAGATGATCAAATTTATAATACTATTGTAACAGCTCATGCTTTTATTATAATTTTTTTTATAGTTATACCAATTATAATTGGAGGATTTGGAAATTG
ATTAATTCCTTTAATATTAGGAGCCCCAGATATAGCTTTTCCTCGAATAAATAATATAAGATTTTGATTACTACCCCCCTCATTAACTTTATTAATT
TCTAGAAGAATTGTAGAAAATGGAGCAGGAACAGGATGAACAGTTTATCCCCCTTTATCATCTAATATCGCTCATAGAGGTAGTTCAGTTGATTTAG
CTATTTTTTCTTTACATTTAGCAGGAATTTCATCAATCTTAGGAGCTATTAATTTTATTACAACTATTATTAATATACGAATTAACCATATATCATT
TGATCAAATACCTCTCTTTGTATGAGCAGTAGGAATTACTGCTTTACTTTTATTATTATCTCTACCTGTATTAGCTGGTGCTATTACTATATTATTA
ACAGATCGAAATCTTAATACTTCATTTTTTGATCCTGCAGGAGGTGGAGATCCTATTTTATATCAACATTTA

HQ004907_RV_08_M274_Romania
ACACTATATTTTATTTTTGGTATTTGAGCAGGTATAGTAGGAACCTCTTTAAGATTATTAATTCGTACTGAATTAGGTAATCCTGGATCTTTAATTG
GAGATGATCAAATTTATAATACTATTGTAACAGCTCATGCTTTTATTATAATTTTTTTTATAGTTATACCAATTATAATTGGAGGATTTGGAAATTG
ATTAATTCCTTTAATATTAGGAGCCCCAGATATAGCTTTTCCTCGAATAAATAATATAAGATTTTGATTACTACCCCCCTCATTAACTTTATTAATT
TCTAGAAGAATTGTAGAAAATGGAGCAGGAACAGGATGAACAGTTTATCCCCCTTTATCATCTAATATCGCTCATAGAGGTAGTTCAGTTGATTTAG
CTATTTTTTCTTTACATTTAGCAGGAATTTCATCAATCTTAGGAGCTATTAATTTTATTACAACTATTATTAATATACGAATTAACCATATATCATT
TGATCAAATACCTCTATTTGTATGAGCAGTAGGAATTACTGCTTTACTTTTATTATTATCTTTACCTGTATTAGCTGGTGCTATTACTATATTATTA
ACAGATCGAAATCTTAATACTTCATTTTTTGATCCTGCAGGAGGTGGAGATCCTATTTTATATCAACATTTA

HQ004902_RV_08_M361_Romania
ACACTATATTTTATTTTTGGTATTTGAGCAGGTATAGTAGGAACCTCTTTAAGATTATTAATTCGTACTGAATTAGGTAATCCTGGATCTTTAATTG
GAGATGATCAAATTTATAATACTATTGTAACAGCTCATGCTTTTATTATAATTTTTTTTATAGTTATACCAATTATAATTGGAGGATTTGGAAATTG

ATTAATTCCCTTAATATTAGGAGCCCCAGATATAGCTTTTCCTCGAATAAATAATATAAGATTTTGATTACTACCCCCCTCATTAACTTTATTAATT
TCTAGAAGAATTGTAGAAAATGGAGCAGGAACAGGATGAACAGTTTATCCCCCTTTATCATCTAATATCGCTCATAGAGGTAGTTCAGTTGATTTAG
CTATTTTTTCTTTACATTTAGCAGGAATTTCATCAATCTTAGGAGCTATTAATTTTATTACAACTATTATTAATATACGAATTAACCATATATCATT
TGATCAAATACCTCTCTTTGTATGAGCAGTAGGAATTACTGCTTTACTTTTATTATTATCTTTACCTGTATTAGCTGGTGCTATTACTATATTATTA
ACAGATCGAAATCTTAATACTTCATTTTTTGATCCTGCAGGAGGTGGAGATCCTATTTTATATCAACATTTA

GU947642_Pmne_ITNEB01_Italy_Sicily ---

CCCCTTCATTAACCTTATTAATTTCTAGAAGAATTGTAGAAAATGGAGCAGGAACTGGATGAACAGTTTACCCCCCTTTATCATCTAATATTGCTCA
TAGAGGAAGTTCAGTTGATTTAGCTATTTTTTCCTTACATTTAGCGGGAATTTCATCAATCTTAGGAGCTATTAATTTTATTACAACTATTATTAAT
ATACGAATTAATCATATATCATTCGATCAAATACCTCTTTTTGTATGAGCAGTAGGAATTACTGCTTTACTTTTATTATTATCTTTACCTGTATTAG
CTGGTGCTATTACTATATTATTAACAGATCGAAATCTTAATACTTCATTTTTTGATCCCGCAGGAGGTGGTGATCCTATTTTATACCAACATTTA

EU093018_Ca995 ---

TTTAATATTAGGAGCCCCAGATATAGCTTTTCCTCGAATAAATAATATAAGATTTTGATTACTGCCCCCCTCATTAACTTTATTAATTTCTAGAAGA
ATTGTAGAAAATGGAGCAGGAACAGGATGAACAGTTTATCCCCCTTTATCATCTAATATCGCTCATAGAGGTAGTTCAGTTGATTTAGCTATTTTTT
CTTTACATTTAGCAGGAATTTCATCAATCTTAGGAGCTATTAATTTTATTACAACTATTATTAATATACGAATTAACCATATATCATTTGATCAAAT
ACCTCTCTTTGTATGAGCAGTAGGAATTACTGCTTTACTTTTATTATTATCTTTACCTGTATTAGCTGGTGCTATTACTATATTATTAACAGATCGA
AATCTTAATACTTCATTTTTTGATCCTGCAGGAGGTGGAGATCCTATTTTATATCAACATTTA

EU093017_Ch892 ---

TTTAATATTAGGAGCCCCAGATATAGCTTTCCCCCGAATAAATAATATAAGATTTTGATTACTACCCCCTTCATTAACCTTATTAATTTCTAGAAGA
ATTGTAGAAAATGGAGCAGGAACTGGATGAACAGTTTACCCCCCTTTATCATCTAATATTGCTCATAGAGGAAGTTCAGTTGATTTAGCTATTTTTT
CCCTACATTTAGCGGGAATTTCATCAATCTTAGGAGCTATTAATTTTATTACAACTATTATCAATATACGAATTAATCATATATCATTCGATCAAAT
ACCTCTTTTTGTATGAGCAGTAGGAATTACTGCTTTACTTTTATTATTATCCTTACCTGTATTAGCTGGTGCTATTACTATATTATTAACAGATCGA
AATCTTAATACTTCATTTTTTGATCCAGCAGGAGGTGGTGATCCTATTTTATATCAACATTTA

EU093016_Tk889 ---

TTTAATATTAGGAGCCCCAGATATAGCTTTTCCTCGAATAAATAATATAAGATTTTGATTACTACCCCCCTCATTAACTTTATTAATTTCTAGAAGA
ATTGTAGAAAATGGAGCAGGAACAGGATGAACAGTTTATCCCCCTTTATCATCTAATATTGCTCATAGAATTAGTTCAGTTGATTTAGCTATTTTTT
CTTTACATTTAGCAGGAATTTCATCAATCTTAGGAGCTATTAATTTTATTACAACTATTATTAATATACGAATTAATCATTTATCATTTGATCAAAT
ACCCCTCTTTGTATGAGCAGTAGGAATTACTGCTTTACTTTTATTATTATCTTTACCTGTATTAGCTGGTGCTATTACTATATTATTAACAGATCGA
AATCTTAATACTTCATTTTTTGATCCTGCAGGAGGTGGAGATCCTATTTTATATCAACATTTA

EU093015_It878 ---

TTTAATATTAGGAGCCCCAGATATAGCTTTCCCCCGAATAAATAATATAAGATTTTGATTACTACCCCCTTCATTAACCTTATTAATTTCTAGAAGA
ATTGTAGAAAATGGAGCAGGAACTGGATGAACAGTTTACCCCCCTTTATCATCTAATATTGCTCATAGAGGAAGTTCAGTTGATTTAGCTATTTTTT
CCTTACATTTAGCGGGAATTTCATCAATCTTAGGAGCTATTAATTTTATTACAACTATTATTAATATACGAATTAATCATATATCATTCGATCAAAT
ACCTCTTTTTGTATGAGCAGTAGGAATTACTGCTTTACTTTTATTATTATCTTTACCTGTATTAGCTGGTGCTATTACTATATTATTAACAGATCGA
AATCTTAATACTTCATTTTTTGATCCAGCAGGAGGTGGTGATCCTATTTTATACCAACATTTA

EU093014_Hu875 ---

TTTAATATTAGGAGCCCCAGATATAGCTTTTCCTCGAATAAATAATATAAGATTTTGATTACTACCCCCCTCATTAACTTTATTAATTTCTAGAAGA
ATTGTAGAAAATGGAGCAGGAACAGGATGAACAGTTTATCCCCCTTTATCATCTAATATCGCTCATAGAGGTAGTTCAGTTGATTTAGCTATTTTTT
CTTTACATTTAGCGGGAATTTCATCAATCTTAGGAGCTATTAATTTTATTACAACTATTATTAATATACGAATTAACCATATATCATTTGATCAAAT
ACCTCTCTTTGTATGAGCAGTAGGAATTACTGCTTTACTTTTATTATTATCTTTACCTGTATTAGCTGGTGCTATTACTATATTATTAACAGATCGA
AATCTTAATACTTCATTTTTTGATCCTGCAGGAGGTGGAGATCCTATTTTATATCAACATTTA

EU093013_Sk872_Cz_Slova ---

TTTAATATTAGGAGCCCCAGATATAGCTTTTCCTCGAATAAATAATATAAGATTTTGATTACTACCCCCCTCATTAACTTTATTAATTTCTAGAAGA
ATTGTAGAAAATGGAGCAGGAACAGGATGAACAGTTTATCCCCCTTTATCATCTAATATCGCTCATAGAGGTAGTTCAGTTGATTTAGCTATTTTTT
CTTTACATTTGGCAGGAATTTCATCAATCTTAGGAGCTATTAATTTTATTACAACTATTATTAATATACGAATTAATCATATATCATTTGATCAAAT
ACCTCTCTTTGTATGAGCAGTAGGAATTACTGCTTTACTTTTATTATTATCTTTACCTGTATTAGCTGGTGCTATTACTATATTATTAACAGATCGA
AATCTTAATACTTCATTTTTTGATCCTGCAGGAGGTGGAGATCCTATTTTATATCAACATTTA

EU093011_Cz869 ---

TTTAATATTAGGAGCCCCAGATATAGCTTTTCCTCGAATAAATAATATAAGATTTTGATTACTACCCCCCTCATTAACTTTATTAATTTCTAGAAGA
ATTGTAGAAAATGGAGCAGGAACAGGATGAACAGTTTATCCCCCTTTATCATCTAATATTGCTCATAGAGGTAGTTCAGTTGATTTAGCTATTTTTT
CTTTACATTTAGCAGGAATTTCATCAATCTTAGGAGCTATTAATTTTATTACAACTATTATTAATATACGAATTAACCATATATCATTTGATCAAAT
ACCTCTCTTTGTATGAGCAGTAGGAATTACTGCTTTACTTTTATTATTATCTTTACCTGTATTAGCTGGTGTTATTACTATATTATTAACAGATCGA
AATCTTAATACTTCATTTTTTGATCCTGCAGGAGGTGGAGATCCTATTTTATATCAACATTTA

EU093010_Ru_Bosnia_Fi_Bu_Hu_Bel_Po_Rom_Ukr_ ---

TTTAATATTAGGAGCCCCAGATATAGCTTTTCCTCGAATAAATAATATAAGATTTTGATTACTACCCCCCTCATTAACTTTATTAATTTCTAGAAGA
ATTGTAGAAAATGGAGCAGGAACAGGATGAACAGTTTATCCCCCTTTATCATCTAATATCGCTCATAGAGGTAGTTCAGTTGATTTAGCTATTTTTT
CTTTACATTTAGCAGGAATTTCATCAATCTTAGGAGCTATTAATTTTATTACAACTATTATTAATATACGAATTAACCATATATCATTTGATCAAAT
ACCTCTCTTTGTATGAGCAGTAGGAATTACTGCTTTACTTTTATTATTATCTTTACCTGTATTAGCTGGTGCTATTACTATATTATTAACAGATCGA
AATCTTAATACTTCATTTTTTGATCCTGCAGGAGGTGGAGATCCTATTTTATATCAACATTTA

EU093009_Kz848 ---

TTTAATATTAGGGGCCCCAGATATAGCTTTTCCTCGAATAAATAATATAAGATTTTGATTATTACCCCCTTCATTAACTTTATTAATTTCTAGAAGA
ATTGTAGAAAATGGAGCAGGAACAGGATGAACAGTTTATCCCCCTTTATCATCTAATATCGCTCATAGAGGTAGTTCAGTTGATTTAGCTATTTTTT
CTTTACATTTAGCAGGAATTTCATCAATCTTAGGAGCTATTAATTTTATTACAACTATTATTAATATACGAATTAATCATATATCATTTGATCAAAT
ACCTCTCTTTGTATGAGCAGTAGGAATTACTGCTTTACTTTTATTACTATCTTTACCTGTATTAGCTGGTGCTATTACTATATTATTAACAGATCGA
AATCTTAATACTTCATTTTTTGATCCTGCAGGAGGTGGAGATCCTATTTTATATCAACATTTA

EU093007_Fi810 ---

TTTAATATTAGGAGCCCCAGATATAGCCTTTCCTCGAATAAATAATATAAGATTTTGATTACTACCCCCCTCATTAACTTTATTAATTTCTAGAAGA
ATTGTAGAAAATGGAGCAGGAACAGGATGAACAGTTTATCCCCCTTTATCATCTAATATCGCTCATAGAGGTAGTTCAGTTGATTTAGCTATTTTTT
CTTTACATTTAGCAGGAATTTCATCAATCTTAGGAGCTATTAATTTTATTACAACTATTATTAATATACGAATTAACCATATATCATTTGATCAAAT
ACCTCTCTTTGTATGAGCAGTAGGAATTACTGCTTTACTTTTATTATTATCTTTACCTGTATTAGCTGGTGCTATTACTATATTATTAACAGATCGA
AATCTTAATACTTCATTTTTTGATCCTGCAGGAGGTGGAGATCCTATTTTATATCAACATTTA

EU093004_Tu999 ---

TTTAATATTAGGAGCTCCAGATATAGCTTTTCCTCGAATAAATAACATAAGATTTTGATTACTCCCCCCTTCATTAACTTTATTAATTTCTAGAAGA
ATTGTAGAAAATGGAGCAGGAACAGGATGAACAGTTTATCCCCCTTTATCATCTAATATTGCTCATAGAGGTAGTTCAGTTGATTTAGCTATTTTTT
CTTTACATTTAGCAGGAATTTCATCAATCTTAGGAGCTATTAATTTTATTACAACTATTATTAATATACGAATTAATAATATATCATTTGATCAAAT
ACCTCTTTTTGTATGAGCAGTAGGAATTACTGCATTACTTTTATTATTATCTTTACCTGTATTAGCTGGTGCTATTACTATATTATTAACAGATCGA
AATCTTAATACTTCATTTTTTGATCCCGCAGGAGGTGGAGATCCTATTTTATATCAACACTTA

EU093000_Bu961 ---

TTTAATATTAGGAGCCCCAGATATAGCTTTTCCTCGAATAAATAATATAAGATTTTGATTACTACCCCCCTCATTAACTTTATTAATTTCTAGAAGA
ATTGTAGAAAATGGAGCAGGAACAGGATGAACAGTTTATCCCCCTTTATCATCTAATATCGCTCATAGAGGTAGTTCAGTTGATTTAGCTATTTTTT
CTTTACATTTAGCAGGAATTTCATCAATCTTAGGAGCTATTAATTTTATTACAACTATTATTAATATACGAATTAATCATATATCATTTGATCAAAT
ACCTCTCTTTGTATGAGCAGTAGGAATTACTGCTTTACTTTTATTATTATCTTTACCTGTATTAGCTGGTGCTATTACTATATTATTAACAGATCGA
AATCTTAATACTTCATTTTTTGATCCTGCAGGAGGTGGAGATCCTATTTTATATCAACATTTA

EU092998_Bu951 ---

TTTAATATTAGGAGCCCCAGATATAGCTTTTCCTCGAATAAATAATATAAGATTTTGATTACTACCCCCCTCATTAACTTTATTAATTTCTAGAAGA
ATTGTAGAAAATGGAGCAGGAACAGGATGAACAGTTTATCCCCCTTTATCATCTAATATCGCTCATAGTGGTAGTTCAGTTGATTTAGCTATTTTTT
CTTTACATTTAGCAGGAATTTCATCAATCTTAGGAGCTATTAATTTTATTACAACTATTATTAATATACGAATTAACCATATATCATTTGATCAAAT
ACCTCTCTTTGTATGAGCAGTAGGAATTACTGCTTTACTTTTATTATTATCTTTACCTGTATTAGCTGGTGCTATTACTATATTATTAACAGATCGA
AATCTTAATACTTCATTTTTTGATCCTGCAGGAGGTGGAGATCCTATTTTATATCAACATTTA

EU092997_Ru940 ---

TTTAATATTAGGAGCCCCAGATATAGCTTTTCCTCGAATAAATAATATAAGATTTTGATTACTACCCCCCTCATTAACTTTATTAATTTCTAGAAGA
ATTGTAGAAAATGGAGCAGGAACAGGATGAACAGTTTATCCCCCTTTATCATCTAATATCGCTCATAGAGGTAGTTCAGTTGATTTAGCTATTTTTT
CTTTACATTTAGCAGGAATTTCATCAATCTTAGGAGCTATTAATTTTATTACAACTATTATTAATATACGAATTAACCATATATCATTTGATCAAAT
ACCTCTCTTTGTATGAGCAGTAGGAATTACTGCTTTACTTTTATTATTATCTCTACCTGTATTAGCTGGTGCTATTACTATATTATTAACAGATCGA
AATCTTAATACTTCATTTTTTGATCCTGCAGGAGGTGGGGATCCTATTTTATATCAACATTTA

EU092996_Sp924_Fr ---

TTTAATATTAGGAGCCCCAGATATAGCTTTCCCCCGAATAAATAATATAAGATTTTGATTACTACCCCCTTCATTAACCTTATTAATTTCTAGAAGA
ATTGTAGAAAATGGAGCAGGAACTGGATGAACAGTTTACCCCCCTTTATCATCCAATATTGCTCATAGAGGAAGTTCAGTTGATTTAGCTATTTTTT
CCCTACATTTAGCAGGAATTTCATCAATCTTAGGAGCTATTAATTTTATTACAACTATTATTAATATACGAATTAATCATATATCATTCGATCAAAT
ACCTCTTTTTGTGTGAGCAGTAGGAATTACTGCTTTACTTTTATTATTATCCTTACCTGTATTAGCTGGTGCTATTACTATATTATTAACAGATCGA
AATCTTAATACTTCATTTTTTGATCCAGCAGGAGGTGGTGATCCTATTTTATATCAACATTTA

EU092995_Sp923 ---

TTTAATATTAGGAGCCCCAGATATAGCTTTCCCCCGAATAAATAATATAAGATTTTGATTACTACCCCCTTCATTAACCTTATTAATTTCTAGAAGA
ATTGTAGAAAATGGAGCAGGAACTGGATGAACAGTTTATCCCCCTTTATCATCCAATATTGCTCATAGAGGAAGTTCAGTTGATTTAGCTATTTTTT
CCCTACATTTAGCGGGAATTTCATCAATCTTAGGAGCTATTAATTTTATTACAACTATTATTAATATACGAATTAATCATATATCATTCGATCAAAT
ACCTCTTTTTGTGTGAGCAGTAGGAATTACTGCTTTACTTTTATTATTATCCTTACCTGTATTAGCTGGTGCTATTACTATATTATTAACGGATCGA
AATCTTAATACTTCATTTTTTGATCCAGCAGGAGGTGGTGATCCTATTTTATATCAACATTTA

EU092994_Sp921 --

TTTAATATTAGGAGCCCCAGATATAGCTTTCCCCCGAATAAATAATATAAGATTTTGATTACTACCCCCTTCATTAACCTTATTAATTTCTAGAAGA
ATTGTAGAAAATGGAGCAGGAACTGGATGAACAGTTTACCCCCCTTTATCATCCAATATTGCTCATAGAGGAAGTTCAGTTGATTTAGCTATTTTTT
CCCTACATTTAGCAGGAATTTCATCAATCTTAGGAGCTATTAATTTTATTACAACTATTATTAATATACGAATTAATCATATATCATTCGATCAAAT
ACCTCTTTTTGTGTGAGCAGTAGGAATTACTGCTTTACTTTTATTATTATCCTTACCTGTATTAGCTGGTGCTATTACTATATTATTAACGGATCGA
AATCTTAATACTTCATTTTTTGATCCAGCAGGAGGTGGTGATCCTATTTTATATCAACATTTA

EU092993_Sp917 --

TTTAATATTAGGAGCCCCAGATATAGCTTTCCCCCGAATAAATAATATAAGATTTTGATTACTACCCCCTTCATTAACCTTATTAATTTCTAGAAGA
ATTGTAGAAAATGGAGCAGGAACTGGATGAACAGTTTATCCCCCTTTATCATCCAATATTGCTCATAGAGGAAGTTCAGTTGATTTAGCTATTTTTT
CCCTACATTTAGCAGGAATTTCATCAATCTTAGGAGCTATTAATTTTATTACAACTATTATTAATATACGAATTAATCATATATCATTCGATCAAAT
ACCTCTTTTTGTGTGAGCAGTAGGAATTACTGCTTTACTTTTATTATTATCCTTACCTGTATTAGCTGGTGCTATTACTATATTATTAACAGATCGA
AATCTTAATACTTCATTTTTTGATCCAGCAGGAGGTGGTGATCCTATTTTATATCAACATTTA

EU092992_Sp916 --

TTTAATATTAGGAGCCCCAGATATAGCTTTCCCCCGAATAAATAATATAAGATTTTGATTACTACCCCCTTCATTAACCTTATTAATTTCTAGAAGA
ATTGTAGAAAATGGAGCAGGAACTGGATGAACAGTTTACCCCCCTTTATCATCCAATATTGCTCATAGAGGAAGTTCAGTTGATTTAGCTATTTTTT
CCCTACATTTAGCAGGAATTTCATCAATCTTAGGAGCTATTAATTTTATTACAACTATTATTAATATACGAATTAATCATATATCATTCGATCAAAT
ACCTCTTTTTGTGTGAGCAGTAGGAATTACTGCTTTACTTTTATTATTATCCTTACCTGTATTAGCTGGTGCTATTACTATATTATTAACAGATCGA
AATATTAATACTTCATTTTTTGATCCAGCAGGAGGTGGTGATCCTATTTTATATCAACATTTA

EU092991_Fr913 --

TTTAATATTAGGAGCCCCAGATATAGCTTTCCCCCGAATAAATAATATAAGATTTTGATTACTACCCCCTTCATTAACCTTATTAATTTCTAGAAGA
ATTGTAGAAAATGGAGCAGGAACTGGATGAACAATTTACCCCCCTTTATCATCCAATATTGCTCATAGAGGAAGTTCAGTTGATTTAGCTATTTTTT
CCCTACATTTAGCAGGAATTTCATCAATCTTAGGAGCTATTAATTTTATTACAACTATTATTAATATACGAATTAATCATATATCATTCGATCAAAT
ACCTCTTTTTGTGTGAGCAGTAGGAATTACTGCTTTACTTTTATTATTATCCTTACCTGTATTAGCTGGTGCTATTACTATATTATTAACAGATCGA
AATCTTAATACTTCATTTTTTGATCCAGCAGGAGGTGGTGATCCTATTTTATATCAACATTTA

EU092990_Fr911 --

TTTAATATTAGGAGCCCCAGATATAGCTTTCCCCCGAATAAATAATATAAGATTTTGATTACTACCCCCTTCATTAACCTTATTAATTTCTAGAAGA
ATTGTAGAAAATGGAGCAGGAACTGGATGAACAGTTTACCCCCCTTTATCATCTAATATTGCTCATAGAGGAAGTTCAGTTGATTTAGCTATTTTTT
CCTTACATTTAGCAGGAATTTCATCAATCTTAGGAGCTATTAATTTTATTACAACTATTATTAATATACGAATTAATCATATATCATTCGATCAAAT
ACCTCTTTTTGTATGAGCAGTAGGAATTACTGCTTTACTTTTATTATTATCATTACCTGTATTAGCTGGTGCTATTACTATATTATTAACAGATCGA
AATCTTAATACTTCATTTTTTGATCCAGCAGGGGGTGGTGATCCTATTTTATATCAACATTTA

EU092988_It639 --

TTTAATATTAGGAGCCCCAGATATAGCTTTCCCCCGAATAAATAATATAAGATTTTGATTACTACCCCCTTCATTAACCTTATTAATTTCTAGAAGA
ATTGTAGAAAATGGAGCAGGAACTGGATGAACAGTTTACCCCCCTTTATCATCTAATATTGCTCATAGAGGAAGTTCAGTTGATTTAGCTATTTTTT
CCCTACATTTAGCGGGAATTTCATCAATCTTAGGAGCTATTAATTTTATTACAACTATTATTAATATACGAATTAATCATATATCATTTGATCAAAT
ACCTCTTTTTGTATGAGCAGTAGGAATTACTGCTTTACTTTTATTATTATCCTTACCTGTATTAGCTGGTGCTATTACTATATTATTAACAGATCGA
AATCTTAATACTTCATTTTTTGATCCAGCAGGAGGTGGTGATCCTATTTTATATCAACATTTA

EU092987_Fr528 --

TTTAATATTAGGAGCCCCAGATATAGCTTTCCCCCGAATAAATAATATAAGATTTTGATTACTACCCCCTTCATTAACCTTATTAATTTCTAGAAGA
ATTGTAGAAAATGGAGCAGGAACTGGATGAACAGTTTACCCCCCTTTATCATCTAATATTGCTCATAGAGGAAGTTCAGTTGATTTAGCTATTTTTT
CCCTACATTTAGCGGGAATTTCATCAATCTTAGGAGCTATTAATTTTATTACAACTATTATTAATATACGAATTAATCATATATCATTCGATCAAAT
ACCTCTTTTTGTATGAGCAGTAGGAATTACTGCTTTACTTTTATTATTATCCTTACCTGTATTAGCTGGTGCTATTACTATATTATTAACAGATCGA
AATCTTAATACTTCATTTTTTGATCCAGCAGGAGGTGGTGATCCTATTTTATATCAACATTTA

EU092985_Fr525 --

TTTAATATTAGGAGCCCCAGATATAGCTTTCCCCCGAATAAATAATATAAGATTTTGATTACTACCCCCTTCATTAACCTTATTAATTTCTAGAAGA
ATTGTAGAAAATGGAGCAGGAACTGGATGAACAGTTTACCCCCCTTTATCATCTAATATTGCTCATAGAGGAAGTTCAGTTGATTTAGCTATTTTTT
CCTTACATTTAGCAGGAATTTCATCAATCTTAGGAGCTATTAATTTTATTACAACTATTATTAATATACGAATTAATCATATATCATTCGATCAAAT
ACCTCTTTTTGTATGAGCAGTAGGAATTACTGCTTTACTTTTATTATTATCATTACCTGTATTAGCTGGTGCTATTACTATATTATTAACAGATCGA
AATCTTAATACTTCATTTTTTGATCCAGCAGGAGGTGGTGATCCTATTTTATATCAACATTTA

EU092984_Fr522 --

TTTAATATTAGGAGCCCCAGATATAGCTTTCCCCCGAATAAATAATATAAGATTTTGATTACTACCCCCTTCATTAACCTTATTAATTTCTAGAAGA
ATTGTAGAAAATGGAGCAGGAACTGGATGAACAGTTTACCCCCCTTTATCATCTAATATTGCTCATAGAGGAAGTTCAGTTGATTTAGCTATTTTTT
CCTTACATTTAGCAGGAATTTCATCAATCTTAGGAGCTATTAATTTTATTACAACTATTATTAATATACGAATTAATCATATATCATTCGATCAAAT
ACCTCTTTTTGTATGAGCAGTAGGAATTACTGCTTTACTCTTATTATTATCATTACCTGTATTAGCTGGTGCTATTACTATATTATTAACAGATCGA
AATCTTAATACTTCATTTTTTGATCCAGCAGGGGGTGGTGATCCTATTTTATATCAACATTTA

EU092983_Ch765 --

TTTAATATTAGGAGCCCCAGATATAGCTTTCCCCCGAATAAATAATATAAGATTTTGATTACTACCCCCTTCATTAACCTTATTAATTTCTAGAAGA
ATTGTAGAAAATGGAGCAGGAACTGGATGAACAGTTTACCCCCCTTTATCATCTAATATTGCTCATAGAGGAAGTTCAGTTGATTTAGCTATTTTTT
CCCTACATTTAGCAGGAATTTCATCAATCTTAGGAGCTATTAATTTTATTACAACTATTATTAATATACGAATTAATCATATATCATTCGATCAAAT
ACCTCTTTTTGTATGAGCAGTAGGAATTACTGCTTTACTTTTATTATTATCCTTACCTGTATTAGCTGGTGCTATTACTATATTATTAACAGATCGA
AATCTTAATACTTCATTTTTTGATCCAGCAGGAGGTGGTGATCCTATTTTATATCAACATTTA

EU092982_Pl653_Slova_Ukr --

TTTAATATTAGGAGCCCCAGATATAGCTTTTCCTCGAATAAATAATATAAGATTTTGATTACTACCCCCCTCATTAACTTTATTAATTTCTAGAAGA
ATTGTAGAAAATGGAGCAGGAACAGGATGAACAATTTATCCCCCTTTATCATCTAATATCGCTCATAGAGGTAGTTCAGTTGATTTAGCTATTTTTT
CTTTACATTTAGCAGGAATTTCATCAATCTTAGGAGCTATTAATTTTATTACAACTATTATTAATATACGAATTAACCATATATCATTTGATCAAAT
ACCTCTCTTTGTATGAGCAGTAGGAATTACTGCTTTACTTTTATTATTATCTTTACCTGTATTAGCTGGTGCTATTACTATATTATTAACAGATCGA
AATCTTAATACTTCATTTTTTGATCCTGCAGGAGGTGGAGATCCTATTTTATATCAACATTTA

EU092981_At626 --

TTTAATATTAGGAGCCCCAGATATAGCTTTTCCTCGAATAAATAATATAAGATTTTGATTACTACCCCCCTCATTAACTTTATTAATTTCTAGAAGA
ATTGTAGAAAATGGAGCAGGAACAGGATGAACAGTTTATCCCCCTTTATCATCTAATATCGCTCATAGAGGTAGTTCAGTTGATTTAGCTATTTTTT
CTTTACATTTAGCAGGAATTTCATCAATCTTAGGAGCTATTAATTTTATTACAACTATTATTAATATACGAATTAACCATATATCATTTGATCAAAT
ACCTCTCTTTGTATGAGCAGTAGGAATTACTGCTTTACTTTTATTATTATCTTTACCTGTGTTAGCTGGTGCTATTACTATATTATTAACAGATCGA
AATCTTAATACTTCATTTTTTGATCCTGCAGGAGGTGGAGATCCTATTTTATATCAACATTTA

EU092980_At622 --

TTTAATATTAGGAGCCCCAGATATAGCTTTTCCTCGAATAAATAATATAAGATTTTGATTACTACCCCCCTCATTAACTTTATTAATTTCTAGAAGA
ATTGTAGAAAATGGAGCAGGAACAGGATGAACAGTTTATCCCCCTTTATCATCTAATATTGCTCATAGAGGTAGTTCAGTTGATTTAGCTATTTTTT
CTTTACATTTAGCAGGAATTTCATCAATCTTAGGAGCTATTAATTTTATTACAACTATTATTAATATACGAATTAACCATATATCATTTGATCAAAT
ACCTCTCTTTGTATGAGCAGTAGGAATTACTGCTTTACTTTTATTACTATCTTTACCTGTATTAGCTGGTGCTATTACTATATTATTAACAGATCGA
AATCTTAATACTTCATTTTTTGATCCTGCAGGAGGTGGAGATCCTATTTTATATCAACATTTA

EU092978_At618_At_Slo --

TTTAATATTAGGAGCCCCAGATATAGCTTTTCCTCGAATAAATAATATAAGATTTTGATTACTACCCCCCTCATTAACTTTATTAATTTCTAGAAGA
ATTGTAGAAAATGGGGCAGGAACAGGATGAACAGTTTATCCCCCTTTATCATCTAATATCGCTCATAGAGGTAGTTCAGTTGATTTAGCTATTTTTT
CTTTACATTTAGCAGGAATTTCATCAATCTTAGGAGCTATTAATTTTATTACAACTATTATTAATATACGAATTAACCATATATCATTTGATCAAAT
ACCTCTCTTTGTATGAGCAGTAGGAATTACTGCTTTACTTTTATTATTATCTTTACCTGTATTAGCTGGTGCTATTACTATATTATTAACAGATCGA
AATCTTAATACTTCATTTTTTGATCCTGCAGGAGGTGGAGATCCTATTTTATATCAACATTTA

EU092977_At606_At_Cz_D_Fi_Hu_Po_Slova --

TTTAATATTAGGAGCCCCAGATATAGCTTTTCCTCGAATAAATAATATAAGATTTTGATTACTACCCCCCTCATTAACTTTATTAATTTCTAGAAGA
ATTGTAGAAAATGGAGCAGGAACAGGATGAACAGTTTATCCCCCTTTATCATCTAATATTGCTCATAGAGGTAGTTCAGTTGATTTAGCTATTTTTT
CTTTACATTTAGCAGGAATTTCATCAATCTTAGGAGCTATTAATTTTATTACAACTATTATTAATATACGAATTAACCATATATCATTTGATCAAAT
ACCTCTCTTTGTATGAGCAGTAGGAATTACTGCTTTACTTTTATTATTATCTTTACCTGTATTAGCTGGTGCTATTACTATATTATTAACAGATCGA
AATCTTAATACTTCATTTTTTGATCCTGCAGGAGGTGGAGATCCTATTTTATATCAACATTTA

EU092976_At602 --

TTTAATATTAGGAGCCCCAGATATAGCTTTTCCTCGAATAAATAATATAAGATTTTGATTACTACCCCCCTCATTAACTTTATTAATTTCTAGAAGA
ATTGTAGAAAATGGAGCAGGAACAGGATGAACAGTTTATCCCCCTTTATCATCTAATATTGCTCATAGAGGTAGTTCAGTTGATTTAGCTATTTTTT
CTTTACATTTAGTAGGAATTTCATCAATCTTAGGAGCTATTAATTTTATTACAACTATTATTAATATACGAATTAACCATATATCATTTGATCAAAT
ACCTCTCTTTGTATGAGCAGTAGGAATTACTGCTTTACTTTTATTATTATCTTTACCTGTATTAGCTGGTGCTATTACTATATTATTAACAGATCGA
AATCTTAATACTTCATTTTTTGATCCTGCAGGAGGTGGAGATCCTATTTTATATCAACATTTA

EU092975_D757 --

TTTAATATTAGGAGCCCCAGATATAGCTTTTCCTCGAATAAATAATATAAGATTTTGATTACTACCCCCCTCATTAACTTTATTAATTTCTAGAAGA
ATTGTAGAAAATGGAGCAGGAACAGGATGAACAGTTTATCTTCCTTTATCATCTAATATTGCTCATAGAGGTAGTTCAGTTGATTTAGCTATTTTTT
CTTTACATTTAGCAGGAATTTCATCAATCTTAGGAGCTATTAATTTTATTACAACTATTATTAATATACGAATTAACCATATATCATTTGATCAAAT
ACCTCTCTTTGTATGAGCAGTAGGAATTACTGCTTTACTTTTATTATTATCTTTACCTGTATTAGCTGGTGCTATTACTATATTATTAACAGATCGA
AATCTTAATACTTCATTTTTTGATCCTGCAGGAGGTGGAGATCCTATTTTATATCAACATTTA

EU092974_D756 --

TTTAATATTAGGAGCCCCAGATATAGCTTTTCCTCGAATAAATAATATAAGATTTTGATTACTACCCCCCTCATTAACTTTATTAATTTCTAGAAGA
ATTGTAGAAAATGGAGCAGGAACAGGATGAACAATTTATCCCCCTTTATCATCTAATATTGCTCATAGAGGTAGTTCAGTTGATTTAGCTATTTTTT
CTTTACATTTAGCAGGAATTTCATCAATCTTAGGAGCTATTAATTTTATTACAACTATTATTAATATACGAATTAACCATATATCATTTGATCAAAT

ACCTCTCTTTGTATGAGCAGTAGGAATTACTGCTTTACTTTTATTATTATCTTTACCTGTATTAGCTGGTGCTATTACTATATTATTAACAGATCGA
AATCTTAATACTTCATTTTTTGATCCTGCAGGAGGTGGAGATCCTATTTTATATCAACATTTA

EU092973_D744 --

TTTAATATTAGGAGCCCCAGATATAGCTTTTCCTCGAATAAATAATATAAGATTTTGATTACTACCCCCCTCATTAACTTTATTAATTTCTAGAAGA
ATTGTAGAAAATGGAGCAGGAACAGGATGAACGGTTTATCCCCCTTTATCATCTAATATTGCTCATAGAGGTAGTTCAGTTGATTTAGCTATTTTTT
CTTTACATTTAGCAGGAATTTCATCAATCTTAGGAGCTATTAATTTTATTACAACTATTATTAATATACGAATTAACCATATATCATTTGATCAAAT
ACCTCTCTTTGTATGAGCAGTAGGAATTACTGCTTTACTTTTATTATTATCTTTACCTGTATTAGCTGGTGCTATTACTATATTATTAACAGATCGA
AATCTTAATACTTCATTTTTTGATCCTGCAGGAGGTGGAGATCCTATTTTATATCAACATTTA

EU092971_Hu697_Bu_Hu --

TTTAATATTAGGAGCCCCAGATATAGCTTTTCCTCGAATAAATAATATAAGATTTTGATTACTACCCCCCTCATTAACTTTATTAATTTCTAGAAGA
ATTGTAGAAAATGGAGCAGGAACAGGATGAACAGTTTATCCCCCTTTATCATCTAATATCGCTCATAGAGGTAGTTCAGTTGATTTAGCTATTTTTT
CTTTACATTTAGCAGGAATTTCATCAATCTTAGGAGCTATTAATTTTATTACAACTATTATTAATATACGAATTAACCATATATCATTTGATCAAAT
ACCTCTCTTTGTATGAGCAGTAGGAATTACTGCTTTACTTTTATTATTATCTTTACCTGTATTAGCTGGTGCTATTACTATATTATTAACAGATCGA
AATCTTAATACTTCATTTTTTGATCCTGCAGGAGGTGGAGATCCTATTTTATATCAACATTTA

EU092969_Bu775 --

TTTAATATTAGGAGCCCCAGATATAGCTTTTCCTCGAATAAATAATATAAGATTTTGATTACTACCCCCCTCATTAACTTTATTAATTTCTAGAAGA
ATTGTAGAAAATGGAGCAGGAACAGGATGAACAATTTATCCCCCTTTATCATCTAATATCGCTCACAGAGGTAGTTCAGTTGATTTAGCTATTTTTT
CTTTACATTTAGCAGGAATTTCATCAATCTTAGGAGCTATTAATTTTATTACAACTATTATTAATATACGAATTAACCATATATCATTTGATCAAAT
ACCTCTCTTTGTATGAGCAGTAGGAATTACTGCTTTACTTTTATTATTATCTTTACCTGTATTAGCTGGTGCTATTACTATATTATTAACAGATCGA
AATCTTAATACTTCATTTTTTGATCCTGCAGGAGGTGGAGATCCTATTTTATATCAACATTTA

EU092968_Bu772 --

TTTAATATTAGGAGCCCCAGATATAGCTTTTCCTCGAATAAATAATATAAGATTTTGATTACTACCCCCCTCATTAACTTTATTAATTTCTAGAAGA
ATTGTAGAAAATGGAGCAGGAACAGGATGAACAGTTTATCCCCCTTTATCATCTAATATCGCTCATAGAGGTAGTTCAGTTGATTTAGCTATTTTTT
CTTTACATTTAGCAGGAATTTCATCAATCTTAGGAGCTATTAATTTTATTACAACTATTATTAATATACGAATTAACCATATATCATTTGATCAAAT
ACCTCTCTTTGTATGAGCAGTAGGAATTACTGCTTTACTTTTATTATTATCTCTACCTGTATTAGCTGGTGCTATTACTATATTATTAACAGATCGA
AATCTTAATACTTCATTTTTTGATCCTGCAGGAGGTGGAGATCCTATTTTATATCAACATTTA

EU093003_D982 --

TTTAATATTAGGAGCCCCAGATATAGCTTTTCCTCGAATAAATAATATAAGATTTTGATTACTACCCCCCTCATTAACTTTATTAATTTCTAGAAGA
ATTGTAGAAAATGGAGCAGGAACAGGATGAACAGTTTATCCCCCTTTATCATCTAATATTGCTCATAGAGGTAGTTCAGTTGATTTAGCTATTTTTT
CTTTACATTTAGCAGGAATTTCATCAATCTTAGGAGCTATTAATTTTATTACAACTATTATTAATATACGAATTAACCATATATCATTTGATCAAAT
ACCTCTCTTTGTATGGGCAGTAGGAATTACTGCTTTACTTTTATTATTATCTTTACCTGTATTAGCTGGTGCTATTACTATATTATTAACAGATCGA
AATCTTAATACTTCATTTTTTGATCCTGCAGGAGGTGGAGATCCTATTTTATATCAACATTTA

DQ407769_UP_100_F
ACATTATATTTTATTTTTGGTATTTGAGCAGGTATAGTAGGAACCTCTTTAAGATTATTAATTCGTACTGAATTAGGTAATCCTGGATCTTTAATTG
GAGATGATCAAATTTATAACACTATTGTAACAGCTCATGCTTTTATTATAATTTTTTTTATAGTTATACCAATTATAATTGGAGGATTTGGAAATTG
ATTAATTCCTTTAATATTAGGAGCCCCAGATATAGCTTTCCCCCGAATAAATAATATAAGATTTTGATTACTACCCCCTTCATTAACCTTATTAATT
TCTAGAAGAATTGTAGAAAATGGAGCAGGAACTGGATGAACAGTTTACCCCCCTTTATCATCTAATATTGCTCATAGAGGAAGTTCAGTTGATTTAG
CTATTTTTTCCTTACATTTAGCAGGAATTTCATCAATCTTAGGAGCTATTAATTTTATTACAACTATTATTAATATACGAATTAATCATATATCATT
CGATCAAATACCTCTTTTTGTATGAGCAGTAGGAATTACTGCTTTACTCTTATTATTATCATTACCTGTATTAGCTGGTGCTATTACTATATTATTA
ACAGATCGAAATCTTAATACTTCATTTTTTGATCCAGCAGGGGGTGGTGA----------------------

AM231426_W336_parvisi_Gre
ACACTATATTTTATTTTTGGTATTTGAGCAGGTATAGTAGGAACCTCTTTAAGATTATTAATTCGTACTGAATTAGGTAATCCTGGATCTTTAATTG
GAGATGATCAAATTTATAATACTATTGTAACAGCTCATGCTTTTATTATAATTTTTTTTATAGTTATACCAATTATAATTGGAGGATTTGGAAATTG
ATTAATTCCTTTAATATTAGGAGCCCCAGATATAGCTTTTCCTCGAATAAATAATATAAGATTTTGATTACTACCCCCCTCATTAACTTTATTAATT
TCTAGAAGAATTG????????????????????GGATGAACAGTTTATCCCCCTTTATCATCTAATATCGCTCATAGAGGTAGTTCAGTTGATTTAG
CTATTTTTTCTTTACATTTAGCAGGAATTTCATCAATCTTAGGGGCTATTAATTTTATTACAACTATTATTAATATACGAATTAACCATATATCATT
TGATCAAATACCTCTCTTTGTATGAGCAGTAGGAATTACTGCTTTACTTTTATTATTATCTTTACCTGTATTAGCTGGTGCTATTACTATATTATTA
ACAGATCGAAATCTTAATACTTCATTTTTTGATCYTGCAGGAGGTGGAGATCCTATTTTATATCAA------

AM231425_W333_angorae_Tur
ACACTATATTTTATTTTTGGTATTTGAGCAGGTATAGTAGGAACCTCTTTAAGATTATTAATTCGTACTGAATTAGGTAATCCTGGATCTTTAATTG
GGGATGATCAAATTTATAATACTATTGTAACAGCTCATGCTTTTATTATAATTTTTTTTATAGTTATACCAATTATAATTGGAGGATTTGGAAATTG
ATTAATTCCTTTAATATTAGGAGCCCCAGATATAGCTTTTCCTCGAATAAATAATATAAGATTTTGATTATTACCCCCCTCATTAACTTTATTAATT
TCTAGAAGAATTG????????????????????GGATGAACAGTTTATCCCCCTTTATCATCTAATATTTCTCATAGAGGTAGTTCAGTTGATTTAG
CTATTTTTTCTTTACATTTAGCAGGAATTTCATCAATCTTAGGAGCTATTAATTTTATTACAACTATTATTAATATACGAATTAATCATATATCATT
TGATCAAATACCCCTTTTTGTATGGGCAGTAGGAATTACTGCTTTACTTTTATTATTATCTTTACCTGTATTAGCTGGTGCTWTTACTATATTATTA
ACAGATCGAAATCTTAATACTTCATTTTTCGATCCCGCAGGAGGTGGGGATCCTATTTTATATCAA------

AM231424_W331_angorae_Tur_Ankara
ACACTATATTTTATTTTTGGTATTTGAGCAGGTATAGTAGGAACCTCTTTAAGATTATTAATTCGTACTGAATTAGGTAATCCTGGATCTTTAATTG
GAGATGATCAAATTTATAATACTATTGTAACAGCTCATGCTTTTATTATAATTTTTTTTATAGTTATACCAATTATAATTGGAGGATTTGGAAATTG
ATTAATTCCTTTAATATTAGGAGCCCCAGATATAGCTTTTCCTCGAATAAATAATATAAGATTTTGATTATTACCCCCCTCATTAACTTTATTAATT
TCTAGAAGAATTGTA??????????????????GGATGAACAGTTTATCCCCCTTTATCATCTAATATCGCTCATAGAGGTAGCTCAGTTGATTTAG

CTATTTTTTCTTTACATTTAGCAGGAATTTCATCAATCTTAGGAGCTATTAATTTTATTACAACTATTATTAATATACGAATTAACCATATATCATT
TGATCAAATACCTCTCTTTGTATGAGCAGTAGGAATTACCGCTTTACTTTTATTATTATCTTTACCTGTATTAGCTGGTGCTATTACTATATTATTA
ACAGATCGAAATCTTAATACTTCATTTTTTGATCCTGCAGGAGGTGGAGATCCTATTTTATATCAA------

AM231423_W329_gigantea_Uzb_Chatkal
ACACTATATTTTATTTTTGGTATTTGAGCAGGTATAGTAGGAACCTCTTTAAGATTATTAATTCGTACTGAATTAGGTAATCCTGGATCTTTAATTG
GAGATGATCAAATTTATAATACTATTGTAACAGCTCATGCTTTTATTATAATTTTTTTTATAGTTATACCAATTATAATTGGAGGATTTGGAAATTG
ATTAATTCCTTTAATATTAGGAGCCCCAGATATAGCTTTTCCTCGAATAAATAATATAAGATTTTGATTATTACCCCCTTCATTAACTTTATTAATT
TCTAGAAGAATTG????????????????????GGATGAACAGTTTATCCCCCTTTATCATCTAATATCGCTCATAGAGGTAGTTCAGTTGATTTAG
CTATTTTTTCTTTACATTTAGCAGGAATTTCATCAATCTTAGGAGCTATTAATTTTATTACAACTATTATTAATATACGAATTAATCATATATCATT
TGATCAAATACCTCTCTTTGTATGAGCAGTAGGAATTACTGCTTTACTTTTATTACTATCTTTACCTGTATTAGCTGGTGCTATTACTATATTATTA
ACAGATCGAAATCTTAATACTTCATTTTTTGATCCTGCAGGAGGTGGAGATCCTATTTTATATCAA------

AM231422_W330_orientalis_Zailijskiy
ACACTATATTTTATTTTTGGTATTTGAGCAGGTATAGTAGGAACCTCTTTAAGATTATTAATTCGTACTGAATTAGGTAATCCTGGATCTTTAATTG
GAGATGATCAAATTTATAATACTATTGTAACAGCTCATGCTTTTATTATAATTTTTTTTATAGTTATACCAATTATAATTGGAGGATTTGGAAATTG
ATTAATTCCTTTAATATTAGGGGCCCCAGATATAGCTTTTCCTCGAATAAATAATATAAGATTTTGATTATTACCCCCTTCATTAACTTTATTAATT
TCTAGAAGAATTGTAGAAAATGGAGCAGGAACAGGATGAACAGTTTATCCCCCTTTATCATCTAATATCGCTCATAGAGGTAGTTCAGTTGATTTAG
CTATTTTTTCTTTACATTTAGCGGGAATTTCATCAATCTTAGGAGCTATTAATTTTATTACAACTATTATTAATATACGAATTAATCATATATCATT
TGATCAAATACCTCTCTTTGTATGAGCAGTAGGAATTACTGCTTTACTTTTATTACTATCTTTACCTGTATTAGCTGGTGCTATTACTATATTATTA
ACAGATCGAAATCTTAATACTTCATTTTTTGATCCTGCAGGAGGTGGAGATCCTATTTTATATCAA------

AM231421_W292_ochracea_Tadj_Zeravsh_
ACACTATATTTTATTTTTGGTATTTGAGCAGGTATAGTAGGAACCTCTTTAAGATTATTAATTCGTACTGAATTAGGTAATCCTGGATCTTTAATTG
GAGATGATCAAATTTATAATACTATTGTAACAGCTCATGCTTTTATTATAATTTTTTTCATAGTTATACCAATTATAATTGGAGGATTTGGAAATTG
ATTAATTCCTTTAATATTAGGAGCCCCAGATATAGCTTTTCCTCGAATAAATAATATAAGATTTTGATTACTACCCCCTTCATTAACTTTATTAATT
TCCAGAAGAATTGTAGAAAATGGAGCAGGAACAGGATGAACAGTTTATCCCCCTTTATCATCTAATATCGCTCATAGAGGTAGTTCAGTTGATTTAG
CTATTTTTTCTTTACATTTAGCAGGAATTTCATCAATTTTAGGAGCTATTAATTTCATTACAACTATTATTAATATACGAATTAACCATATATCATT
TGATCAAATACCCCTCTTTGTATGAGCAGTAGGAATTACTGCTTTACTTTTATTATTATCTTTACCTGTATTAGCTGGTGCTATTACTATATTATTA
ACAGATCGAAATCTTAATACTTCATTTTTTGATCCTGCAGG??GTGGAGATCCTATTTTATATCAA------

AM231420_W280_farsica_Iran_Fars
ACACTATATTTTATTTTCGGTATTTGAGCAGGTATAGTAGGAACCTCTTTAAGATTATTAATTCGTACTGAATTAGGTAATCCTGGATCTTTAATTG
GAGATGATCAAATTTATAATACTATTGTAACAGCTCATGCTTTTATTATAATCTTTTTTATAGTTATACCAATTATAATTGGAGGATTTGGAAATTG
ATTAATTCCTTTAATATTAGGAGCTCCAGATATAGCTTTCCCCCGAATAAATAATATAAGATTTTGATTACTCCCCCCCTCATTAACTTTATTAATT
TCTAGAAGAATTGTAGAAAATGGAGCAGGAACAGGATGAACAGTTTACCCCCCTTTATCATCTAATATTGCCCACAGAGGTAGTTCAGTTGATTTAG
CTATTTTTTCTTTACATTTAGCAGGAATTTCATCAATTTTAGGAGCTATTAATTTTATTACAACTATTATTAATATACGAATTAATTATATATCATT
TGATCAAATACCTCTTTTTGTATGAGCAGTAGGAATTACAGCATTACTTTTATTATTATCTTTACCTGTATTAGCTGGTGCTATTACTATATTATTA
ACAGATCGAAATCTTAACACTTCATTTTTTGATCCTGCA????GTGGGGATCCTATTTTATATCAA------

AM231419_W311_pseudonubilosus_Iran_Urmia
ACACTATATTTTATTTTCGGTATTTGAGCGGGTATAGTAGGAACCTCTTTAAGATTATTAATTCGTACTGAATTAGGTAATCCTGGATCTTTAATTG
GAGATGATCAAATTTATAACACTATTGTAACAGCTCATGCTTTTATTATAATTTTTTTTATAGTTATACCAATTATAATTGGAGGATTTGGAAATTG
ATTAATTCCTTTAATATTAGGAGCTCCAGATATAGCTTTTCCTCGAATAAATAATATAAGATTTTGATTACTCCCCCCCTCATTAACTTTATTAATT
TCTAGAAGAATTGTAGAAAATGGAGCAGGAACAGGATGAACAATTTATCCCCCTTTATCATCTAATATTGCTCATAGAGGTAGTTCAGTTGATTTAG
CTATTTTTTCTTTACATTTAGCAGGAATTTCATCAATCTTAGGAGCTATTAATTTTATTACAACTATTATTAACATACGAATTAATCATATATCATT
TGATCAAATACCTCTTTTTGTATGAGCAGTAGGAATTACTGCATTACTTTTATTATTATCTTTACCTGTATTAGCTGGTGCTATTACTATATTATTA
ACAGATCGAAATCTTAATACTTCATTTTTTGATCCTGCAGGA?GTGGAGATCCTATTTTATATCAA------

AM231418_W335_sheljuzhkoi_Tur_Adana
ACACTATATTTTATTTTTGGTATTTGAGCAGGTATAGTAGGAACCTCTTTAAGATTATTAATTCGTACTGAATTAGGTAATCCTGGATCTTTAATTA
GAGATGATCAAATTTATAATACTATTGTAACAGCTCATGCTTTTATTATAATTTTTTTTATAGTTATACCAATTATAATTGGAGGATTTGGAAATTG
ATTAATTCCTTTAATATTAGGAGCCCCAGATATAGCTTTTCCTCGAATAAATAATATAAGATTTTGATTACTTCCCCCTTCATTAACTTTATTAATT
TCTAGAAGAATTG????????????????????GGATGAACAGTTTATCCCCCTTTATCATCTAATATTGCTCATAGAGGTAGTTCAGTTGATTTAG
CTATTTTTTCTTTACATTTAGCAGGAATTTCATCAATCTTAGGAGCTATTAATTTTATTACAACTATTATTAATATACGAATTAATAATATATCATT
TGATCAAATACCTCTTTTTGTATGAGCAGTAGGAATTACTGCATTACTTTTATTATTATCCCTACCTGTATTAGCTGGTGCTATTACTATATTATTA
ACAGATCGAAATCTTAATACTTCATTTTTTGATCCCGCAGGAGGTGGAGATCCTATTTTATATCAA------

AM231417_W78_parmenides_Fr
ACATTATATTTTATTTTTGGTATTTGAGCAGGTATAGTAGGAACCTCTTTAAGATTATTAATTCGTACTGAATTAGGTAATCCTGGATCTTTAATTG
GAGATGATCAAATTTATAATACTATTGTAACAGCTCATGCTTTTATTATAATTTTTTTTATAGTTATACCAATTATAATTGGAGGATTTGGAAATTG
ATTAATTCCTTTAATATTAGGAGCCCCAGATATAGCTTTCCCCCGAATAAATAATATAAGATTTTGATTACTACCCCCTTCATTAACCTTATTAATT
TCTAGAAGAATTGTAGAAAATGGAGCAGGAACTGGATGAACAGTTTACCCCCCTTTATCATCTAATATTGCTCATAGAGGAAGTTCAGTTGATTTAG
CTATTTTTTCCTTACATTTAGCAGGAATTTCATCAATCTTAGGAGCTATTAATTTTATTACAACTATTATTAATATACGAATTAATCATATATCATT
CGATCAAATACCTCTTTTTGTATGAGCAGTAGGAATTACTGCTTTACTCTTATTATTATCATTACCTGTATTAGCTGGTGCTATTACTATATTATTA
ACAGATCGAAATCTTAATACTTCATTTTTTGATCCAGCAGGGGGTGGTGATCCTATTTTATATCAA------

EU836682_h_1_32_Rus ---

GCTTTTCCTCGAATAAATAATATAAGATTTTGATTACTACCCCCCTCATTAACTTTATTAATTTCTAGAAGAATTGTAGAAAATGGAGCAGGAACAG
GATGAACAGTTTATCCCCCTTTATCATCTAATATCGCTCATAGAGGTAGTTCAGTTGATTTAGCTATTTTTTCTTTACATTTAGCAGGAATTTCATC
AATCTTAGGAGCTATTAATTTTATTACAACTATTATTAATATACGAATTAACCATATATCATTTGATCAAATACCTCTCTTTGTATGAGCAGTAGGA
ATTACTGCTTTACTTTTATTATTATCTTTACCTGTATTAGCTGGTGCTATTACTATATTATTAACAGATCGAAATCTTAATACTTCATTTTTTGATC
CTGCAGGAGGTGGGGATCCTATTTTATATCAACATTTA

EU836681_h_1_31_Rus ---

GCTTTTCCTCGAATAAATAATATAAGATTTTGATTACTACCCCCCTCATTAACTTTATTAATTTCTAGAAGAATTGTAGAAAATGGAGCAGGAACAG

GATGAACAATTTATCCCCCTTTATCATCTAATATCGCTCATAGAGGTAGTTCAGTTGATTTAGCTATTTTTTCTTTACATTTAGCAGGAATTTCATC
AATCTTAGGAGCTATTAATTTTATTACAACTATTATTAATATACGAATTAACCATATATCATTTGATCAAATACCTCTCTTTGTATGAGCAGTAGGA
ATTACTGCTTTACTTTTATTATTATCTTTACCTGTATTAGCTGGTGCTATTACTATATTATTAACAGATCGAAATCTTAATACTTCATTTTTTGATC
CTGCAGGAGGTGGGGATCCTATTTTATATCAACATTTA

EU836680_A_Bo_Bu_Cr_Cz_Hu_Sl_Li_Fi_Po_Ru --

GCTTTTCCTCGAATAAATAATATAAGATTTTGATTACTACCCCCCTCATTAACTTTATTAATTTCTAGAAGAATTGTAGAAAATGGAGCAGGAACAG
GATGAACAGTTTATCCCCCTTTATCATCTAATATCGCTCATAGAGGTAGTTCAGTTGATTTAGCTATTTTTTCTTTACATTTAGCAGGAATTTCATC
AATCTTAGGAGCTATTAATTTTATTACAACTATTATTAATATACGAATTAACCATATATCATTTGATCAAATACCTCTCTTTGTATGAGCAGTAGGA
ATTACTGCTTTACTTTTATTATTATCTTTACCTGTATTAGCTGGTGCTATTACTATATTATTAACAGATCGAAATCTTAATACTTCATTTTTTGATC
CTGCAGGAGGTGGAGATCCTATTTTATATCAACATTTA

EU836675_h_1_19_It --

GCTTTCCCTCGAATAAATAATATAAGATTTTGATTACTACCCCCCTCATTAACTTTATTAATTTCTAGAAGAATTGTAGAAAATGGAGCAGGAACAG
GATGAACAGTTTATCCCCCTTTATCATCTAATATTGCTCATAGAGGTAGTTCAGTTGATTTAGCTATTTTTTCTTTACATTTAGCAGGAATTTCATC
AATCTTAGGAGCTATTAATTTTATTACAACTATTATTAATATACGAATTAACCATATATCATTTGATCAAATACCTCTCTTTGTATGAGCAGTAGGA
ATTACTGCTTTACTTTTATTATTATCTTTACCTGTATTAGCTGGTGCTATTACTATATTATTAACAGATCGAAATCTTAATACTTCATTTTTTGATC
CTGCAGGAGGTGGAGATCCTATTTTATATCAACATTTA

EU836674_h_1_18_At_It --

GCTTTTCCTCGAATAAATAATATAAGATTTTGATTACTACCCCCCTCATTAACTTTATTAATTTCTAGAAGAATTGTAGAAAATGGAGCAGGAACAG
GATGAACAGTTTATCCCCCTTTATCATCTAATATTGCTCATAGAGGTAGTTCAGTTGATTTAGCTATTTTTTCTTTACATTTAGCAGGAATTTCATC
AATCTTAGGAGCTATTAATTTTATTACAACTATTATTAATATACGAATTAACCATATATCATTTGATCAAATACCTCTCTTTGTATGAGCAGTAGGA
ATTACTGCTTTACTTTTATTATTATCTTTACCTGTATTAGCTGGTGCTATTACTATATTATTAACAGATCGAAATCTTAATACTTCATTTTTTGATC
CTGCAGGAGGTGGAGATCCTATTTTATATCAACATTTA

EU836672_h_1_10_It --

GCTTTCCCTCGAATAAATAATATAAGATTTTGATTACTACCCCCCTCATTAACTTTATTAATTTCTAGAAGAATTGTAGAAAATGGGGCAGGAACAG
GATGAACAGTTTATCCCCCTTTATCATCTAATATCGCTCATAGAGGTAGTTCAGTTGATTTAGCTATTTTTTCTTTACATTTAGCAGGAATTTCATC
AATCTTAGGAGCTATTAATTTTATTACAACTATTATTAATATACGAATTAACCATATATCATTTGATCAAATACCTCTCTTTGTATGAGCAGTAGGA
ATTACTGCTTTACTTTTATTATTATCTTTACCTGTATTAGCTGGTGCTATTACTATATTATTAACAGATCGAAATCTTAATACTTCATTTTTTGATC
CTGCAGGAGGTGGAGATCCTATTTTATATCAACATTTA

EU836671_h_1_09_It --

GCTTTTCCTCGAATAAATAATATAAGATTTTGATTACTACCCCCCTCATTAACTTTATTAATTTCTAGAAGAATTGTAGAAAATGGGGCAGGAACAG
GATGAACAGTTTATCCCCCTTTATCATCTAATATCGCTCATAGAGGTAGTTCAGTTGATTTAGCTATTTTTTCTTTACATTTAGCAGGAATTTCATC
AATCTTAGGAGCTATTAATTTTATTACAACTATTATTAATATACGAATTAACCATATATCATTTGATCAAATACCTCTCTTTGTATGAGCAGTAGGA
ATTACTGCTTTACTTTTATTATTATCTTTACCTGTATTAGCTGGTGCTATTACTATATTATTAACAGATCGAAATCTTAATACTTCATTTTTTGATC
CTGCAGGAGGTGGAGATCCTATTTTATATCAACATTTA

EU836670_h_1_07_Gre --

GCTTTTCCTCGAATAAATAATATAAGATTTTGATTACTACCCCCCTCATTAACTTTATTAATTTCTAGAAGAATTGTAGAAAATGGAGCAGGAACAG
GATGAACAATTTATCCCCCTTTATCATCTAATATCGCTCATAGAGGTAGTTCAGTTGATTTAGCTATTTTTTCTTTACATTTAGCAGGAATTTCATC
AATCTTAGGGGCTATTAATTTTATTACAACTATTATTAATATACGAATTAACCATATATCATTTGATCAAATACCTCTCTTTGTATGAGCAGTAGGA
ATTACTGCTTTACTTTTATTATTATCTTTACCTGTATTAGCTGGTGCTATTACTATATTATTAACAGATCGAAATCTTAATACTTCATTTTTTGATC
CTGCAGGAGGTGGAGATCCTATTTTATATCAACATTTA

EU836669_h_1_06_Gre --

GCTTTCCCTCGAATAAATAATATAAGATTTTGATTACTACCCCCCTCATTAACTTTATTAATTTCTAGAAGAATTGTAGAAAATGGAGCAGGAACAG
GATGAACAGTTTATCCCCCTTTATCATCTAATATAGCTCATAGAGGTAGTTCAGTTGATTTAGCTATTTTTTCTTTACATTTAGCAGGAATTTCATC
AATCTTAGGAGCTATTAATTTTATTACAACTATTATTAATATACGAATTAACCATATATCATTTGATCAAATACCTCTCTTTGTATGAGCAGTAGGA
ATTACTGCTTTACTTTTATTATTATCTTTACCTGTATTAGCTGGTGCTATTACTATATTATTAACAGATCGAAATCTTAATACTTCATTTTTTGATC
CTGCAGGAGGTGGAGATCCTATTTTATATCAACATTTA

EU836668_h_1_05_Gre --

GCTTTCCCTCGAATAAATAATATAAGATTTTGATTACTACCCCCCTCATTAACTTTATTAATTTCTAGAAGAATTGTAGAAAATGGAGCAGGAACAG
GATGAACAGTTTATCCCCCTTTATCATCTAATATCGCTCATAGAGGTAGTTCAGTTGATTTAGCTATTTTTTCTTTACATTTAGCAGGAATTTCATC
AATCTTAGGGGCTATTAATTTTATTACAACTATTATTAATATACGAATTAACCATATATCATTTGATCAAATACCTCTCTTTGTATGAGCAGTAGGA
ATTACTGCTTTACTTTTATTATTATCTTTACCTGTATTAGCTGGTGCTATTACTATATTATTAACAGATCGAAATCTTAATACTTCATTTTTTGATC
CTGCAGGAGGTGGAGATCCTATTTTATATCAACATTTA

EU836667_h_1_04_Gre --


```
GCTTTCCCTCGAATAAATAATATAAGATTTTGATTACTACCCCCCTCATTAACTTTATTAATTTCTAGAAGAATTGTAGAAAATGGAGCAGGAACAG
GATGAACAGTTTATCCCCCTTTATCATCTAATATCGCTCATAGAGGTAGTTCAGTTGATTTAGCTATTTTTTCTTTACATTTAGCAGGAATTTCATC
AATCTTAGGGGCTATTAATTTTATTACAACTATTATTAATATACGAATTAACCATATATCATTTGATCAAATACCTCTCTTTGTATGAGCAGTAGGA
ATTACTGCTTTACTTTTATTATTATCTTTACCCGTATTAGCTGGTGCTATTACTATATTATTAACAGATCGAAATCTTAATACTTCATTTTTTGATC
CTGCAGGAGGTGGAGATCCTATTTTATATCAACATTTA

       EU836666_h_1_03_Mac                         ---------------------------------------------
-------------------------------------------------------------------------------------------------
--------------------------------------------------------------------------------------
GCTTTTCCTCGAATAAATAATATAAGATTTTGATTACTACCCCCCTCATTAACTTTATTAATTTCTAGAAGAATTGTAGAAAATGGAGCAGGAACAG
GATGAACAGTTTATCCCCCTTTATCATCTAATATCGCTCATAGAGGTAGTTCAGTTGATTTAGCTATTTTTTCTTTACATTTAGCGGAATTTCATC
AATCTTAGGGGCTATTAATTTTATTACAACTATTATTAATATACGAATTAACCATATATCATTTGATCAAATACCTCTCTTTGTATGAGCAGTAGGA
ATTACTGCTTTACTTTTATTATTATCTTTACCTGTATTAGCTGGTGCTATTACTATATTATTAACAGATCGAAATCTTAATACTTCATTTTTTGATC
CTGCAGGAGGTGGAGATCCTATTTTATATCAACATTTA

       EU836665_h_1_02_Gre_Mac                     ---------------------------------------------
-------------------------------------------------------------------------------------------------
--------------------------------------------------------------------------------------
GCTTTTCCTCGAATAAATAATATAAGATTTTGATTACTACCCCCCTCATTAACTTTATTAATTTCTAGAAGAATTGTAGAAAATGGAGCAGGAACAG
GATGAACAGTTTATCCCCCTTTATCATCTAATATCGCTCATAGAGGTAGTTCAGTTGATTTAGCTATTTTTTCTTTACATTTAGCAGGAATTTCATC
AATCTTAGGGGCTATTAATTTTATTACAACTATTATTAATATACGAATTAACCATATATCATTTGATCAAATACCTCTCTTTGTATGAGCAGTAGGA
ATTACTGCTTTACTTTTATTATTATCTTTACCTGTATTAGCTGGTGCTATTACTATATTATTAACAGATCGAAATCTTAATACTTCATTTTTTGATC
CTGCAGGAGGTGGAGATCCTATTTTATATCAACATTTA

       EU836664_h_2_06_At_It                       ---------------------------------------------
-------------------------------------------------------------------------------------------------
--------------------------------------------------------------------------------------
GCTTTTCCTCGAATAAATAATATAAGATTTTGATTGCTACCCCCCTCATTAACTTTATTAATTTCTAGAAGAATTGTAGAAAATGGAGCAGGAACAG
GATGAACAGTTTATCCCCCTTTATCATCTAATATCGCTCATAGAGGTAGTTCAGTTGATTTAGCTATTTTTTCTTTACATTTAGCAGGAATTTCATC
AATCTTAGGAGCTATTAATTTTATTACAACTATTATTAATATACGAATTAACCATATATCATTTGATCAAATACCTCTCTTTGTATGAGCAGTAGGA
ATTACTGCTTTACTTTTATTATTATCTTTACCTGTATTAGCTGGTGCTATTACTATATTATTAACAGATCGAAATCTTAATACTTCATTTTTTGATC
CTGCAGGAGGTGGAGATCCTATTTTATATCAACATTTA

       EU836663_h_2_05_It                          ---------------------------------------------
-------------------------------------------------------------------------------------------------
--------------------------------------------------------------------------------------
GCTTTCCCTCGAATAAATAATATAAGATTTTGATTGCTACCCCCCTCATTAACTTTATTAATTTCTAGAAGAATTGTAGAAAATGGAGCAGGAACAG
GATGAACAGTTTATCCCCCTTTATCATCTAATATCGCTCATAGAGGTAGTTCAGTTGATTTAGCTATTTTTTCTTTACATTTAGCAGGAATTTCATC
AATCTTAGGAGCTATTAATTTTATTACAACTATTATTAATATACGAATTAACCATATATCATTTGATCAAATACCTCTCTTTGTATGAGCAGTAGGA
ATTACTGCTTTACTTTTATTATTATCTTTACCTGTATTAGCTGGTGCTATTACTATATTATTAACAGATCGAAATCTTAATACTTCATTTTTTGATC
CTGCAGGAGGTGGAGATCCTATTTTATATCAACATTTA;


END;
begin mrbayes;
       set autoclose=yes nowarn=yes;
       partition ancstates = 2: 1, 2-655;
       set partition=ancstates;
       lset applyto=(2) rates=invgamma nst=6;
       unlink statefreq=(all) revmat=(all) shape=(all) pinvar=(all);
       prset ratepr=variable;
       constraint mnemosyne_clade2=LOWAM001_Kyrgyzstan_Alai_10 LOWAM004_Uzbekistan_Gissar_38
LOWAM007_Kyrgyzstan_TianShan24 LOWAM009_Kyrgyzstan_TianShan_6 LOWAM013_Turkey_Georgia_3
LOWAM014_Turkey_Ovitdagi_1 LOWAM015_Don_Borisovka_Saratov LOWAM018_Kyrgyzstan_Karamyk
LOWAM019_Kyrgyzstan_Karamyk LOWAM021_Russia_Saratov LOWAM023_Saratov_Luga_Vologda_4
LOWAM027_Kazakhstan_Karatau_2 LOWAM037_Russia_Pskovskaya LOWAM038_Kyrgyzstan_Tjuz_Ashu_3
LOWAM039_Kyrgyzstan_23 LOWAM044_Russia_Borisovra_3 LOWAM045_Tajikistan_Shakhristan_3
LOWAM067_Kazakhstan_Kaindy_1 LOWAM069_Russia_Teberda_2 LOWAM073_Kyrgyzstan_Takhtalyk1
LOWAM074_Kyrgyzstan_Naryntoo LOWAM086_Turkmenistan_3 LOWAM087_Uzbekistan_Sarchashma
LOWAM095_Kyrgyzstan_Sosnovka LOWAM096_Gissar_5 LOWAM098_Tajikistan_Khondiza1
LOWAM099_Uzbekistan_Kamchik_2 LOWAM100_Kyrgyzstan_Konduk LOWAM115_Azerbaijan_Nyus_Nyus
LOWAM116_Iran_Dizin_2 LOWAM122_Russia_Saratow_1 LOWAM136_Uzbekistan_Tamshush_1
LOWAM149_Kyrgyzstan_Songkel_1 LOWAM198_Turkey_Aladaglar_2 LOWAM213_Tajikistan_Tandukul_1
Parnassius_stubbendorfii_2005_LOWA_815 Parnassius_stubbendorfii_2005_LOWA_154
Parnassius_stubbendorfii_2005_LOWA_153 Parnassius_ariadne_ariadne Parnassius_glacialis_mikado
Parnassius_stubbendorfii_hoenei Parnassius_stubbendorfii_hoenei_AC20_16
Parnassius_stubbendorfii_koreanus Parnassius_ariadne_AC4_14 HQ004911_RV_07_C107_Romania
HQ004908_RV_07_D060_Romania HQ004907_RV_08_M274_Romania HQ004902_RV_08_M361_Romania
GU947642_Pmne_ITNEB01_Italy_Sicily EU093018_Ca995 EU093017_Ch892 EU093016_Tk889 EU093015_It878
EU093014_Hu875 EU093013_Sk872_Cz_Slova EU093011_Cz869 EU093010_Ru_Bosnia_Fi_Bu_Hu_Bel_Po_Rom_Ukr_
EU093009_Kz848 EU093007_Fi810 EU093004_Tu999 EU093000_Bu961 EU092998_Bu951 EU092997_Ru940
EU092996_Sp924_Fr EU092995_Sp923 EU092994_Sp921 EU092993_Sp917 EU092992_Sp916 EU092991_Fr913
EU092990_Fr911 EU092988_It639 EU092987_Fr528 EU092985_Fr525 EU092984_Fr522 EU092983_Ch765
EU092982_Pl653_Slova_Ukr EU092981_At626 EU092980_At622 EU092978_At618_At_Slo
EU092977_At606_At_Cz_D_Fi_Hu_Po_Slova EU092976_At602 EU092975_D757 EU092974_D756 EU092973_D744
EU092971_Hu697_Bu_Hu EU092969_Bu775 EU092968_Bu772 EU093003_D982 DQ407769_UP_100_F
AM231426_W336_parvisi_Gre AM231425_W333_angorae_Tur AM231424_W331_angorae_Tur_Ankara
AM231423_W329_gigantea_Uzb_Chatkal AM231422_W330_orientalis_Zailijskiy
AM231421_W292_ochracea_Tadj_Zeravsh_ AM231420_W280_farsica_Iran_Fars
AM231419_W311_pseudonubilosus_Iran_Urmia AM231418_W335_sheljuzhkoi_Tur_Adana
```

```
AM231417_W78_parmenides_Fr EU836682_h_1_32_Rus EU836681_h_1_31_Rus
EU836680_A_Bo_Bu_Cr_Cz_Hu_Sl_Li_Fi_Po_Ru EU836675_h_1_19_It EU836674_h_1_18_At_It
EU836672_h_1_10_It EU836671_h_1_09_It EU836670_h_1_07_Gre EU836669_h_1_06_Gre EU836668_h_1_05_Gre
EU836667_h_1_04_Gre EU836666_h_1_03_Mac EU836665_h_1_02_Gre_Mac EU836664_h_2_06_At_It
EU836663_h_2_05_It;
	prset topologypr=constraints(mnemosyne_clade2);
	report applyto=(1) ancstates=yes;
	mcmcp ngen= 100000 relburnin=yes burninfrac=0.25 printfreq=100  samplefreq=100 nchains=4
savebrlens=yes;
	mcmc;
	sump;
end;
```

Alignment and command block for reconstruction of the ancestral states in the clade (3) (*stubbendorfii+glacialis+hoenei*)]

```
#NEXUS

BEGIN DATA;
	DIMENSIONS  NTAX=125 NCHAR=655;
	format datatype=mixed(standard:1,DNA:2-655) interleave=yes gap=- missing=?;
	MATRIX
	Parnassius_orleans_AC10_5			0
	LOWAM001_Kyrgyzstan_Alai_10			1
	LOWAM004_Uzbekistan_Gissar_38			1
	LOWAM007_Kyrgyzstan_TianShan24			1
	LOWAM009_Kyrgyzstan_TianShan_6			1
	LOWAM013_Turkey_Georgia_3			1
	LOWAM014_Turkey_Ovitdagi_1			1
	LOWAM015_Don_Borisovka_Saratov			1
	LOWAM018_Kyrgyzstan_Karamyk			1
	LOWAM019_Kyrgyzstan_Karamyk			1
	LOWAM021_Russia_Saratov			1
	LOWAM023_Saratov_Luga_Vologda_4			1
	LOWAM027_Kazakhstan_Karatau_2			1
	LOWAM037_Russia_Pskovskaya			1
	LOWAM038_Kyrgyzstan_Tjuz_Ashu_3			1
	LOWAM039_Kyrgyzstan_23			1
	LOWAM044_Russia_Borisovra_3			1
	LOWAM045_Tajikistan_Shakhristan_3			1
	LOWAM067_Kazakhstan_Kaindy_1			1
	LOWAM069_Russia_Teberda_2			1
	LOWAM073_Kyrgyzstan_Takhtalyk1			1
	LOWAM074_Kyrgyzstan_Naryntoo			1
	LOWAM086_Turkmenistan_3			1
	LOWAM087_Uzbekistan_Sarchashma			1
	LOWAM095_Kyrgyzstan_Sosnovka			1
	LOWAM096_Gissar_5			1
	LOWAM098_Tajikistan_Khondiza1			1
	LOWAM099_Uzbekistan_Kamchik_2			1
	LOWAM100_Kyrgyzstan_Konduk			1
	LOWAM115_Azerbaijan_Nyus_Nyus			1
	LOWAM116_Iran_Dizin_2			1
	LOWAM122_Russia_Saratow_1			1
	LOWAM136_Uzbekistan_Tamshush_1			1
	LOWAM149_Kyrgyzstan_Songkel_1			1
	LOWAM198_Turkey_Aladaglar_2			1
	LOWAM213_Tajikistan_Tandukul_1			1
	Parnassius_clodius			0
	Parnassius_eversmanni_2005_LOWA_108			0
	Parnassius_eversmanni_2005_LOWA_107			0
	Parnassius_stubbendorfii_2005_LOWA_815			1
	Parnassius_stubbendorfii_2005_LOWA_154			1
	Parnassius_stubbendorfii_2005_LOWA_153			1
	Parnassius_nordmanni			0
	Parnassius_eversmanni			0
	Parnassius_eversmanni_felderi			0
	Parnassius_ariadne_ariadne			0
	Parnassius_glacialis_mikado			1
	Parnassius_stubbendorfii_hoenei			1
	Parnassius_stubbendorfii_hoenei_AC20_16			1
	Parnassius_stubbendorfii_koreanus			1
	Parnassius_nordmanni_AC20_5			0
```

```
        Parnassius_eversmanni_felderi_AC23_68        0
        Parnassius_eversmanni_eversmanni_AC1_14      0
        Parnassius_clodius_AC4_5                     0
        Parnassius_ariadne_AC4_14                    0
        HQ004911_RV_07_C107_Romania                  1
        HQ004908_RV_07_D060_Romania                  1
        HQ004907_RV_08_M274_Romania                  1
        HQ004902_RV_08_M361_Romania                  1
        GU947642_Pmne_ITNEB01_Italy_Sicily           1
        EU093018_Ca995                               1
        EU093017_Ch892                               1
        EU093016_Tk889                               1
        EU093015_It878                               1
        EU093014_Hu875                               1
        EU093013_Sk872_Cz_Slova                      1
        EU093011_Cz869                               1
        EU093010_Ru_Bosnia_Fi_Bu_Hu_Bel_Po_Rom_Ukr_  1
        EU093009_Kz848                               1
        EU093007_Fi810                               1
        EU093004_Tu999                               1
        EU093000_Bu961                               1
        EU092998_Bu951                               1
        EU092997_Ru940                               1
        EU092996_Sp924_Fr                            1
        EU092995_Sp923                               1
        EU092994_Sp921                               1
        EU092993_Sp917                               1
        EU092992_Sp916                               1
        EU092991_Fr913                               1
        EU092990_Fr911                               1
        EU092988_It639                               1
        EU092987_Fr528                               1
        EU092985_Fr525                               1
        EU092984_Fr522                               1
        EU092983_Ch765                               1
        EU092982_Pl653_Slova_Ukr                     1
        EU092981_At626                               1
        EU092980_At622                               1
        EU092978_At618_At_Slo                        1
        EU092977_At606_At_Cz_D_Fi_Hu_Po_Slova        1
        EU092976_At602                               1
        EU092975_D757                                1
        EU092974_D756                                1
        EU092973_D744                                1
        EU092971_Hu697_Bu_Hu                         1
        EU092969_Bu775                               1
        EU092968_Bu772                               1
        EU093003_D982                                1
        DQ407769_UP_100_F                            1
        AM231426_W336_parvisi_Gre                    1
        AM231425_W333_angorae_Tur                    1
        AM231424_W331_angorae_Tur_Ankara             1
        AM231423_W329_gigantea_Uzb_Chatkal           1
        AM231422_W330_orientalis_Zailijskiy          1
        AM231421_W292_ochracea_Tadj_Zeravsh_         1
        AM231420_W280_farsica_Iran_Fars              1
        AM231419_W311_pseudonubilosus_Iran_Urmia     1
        AM231418_W335_sheljuzhkoi_Tur_Adana          1
        AM231417_W78_parmenides_Fr                   1
        EU836682_h_1_32_Rus                          1
        EU836681_h_1_31_Rus                          1
        EU836680_A_Bo_Bu_Cr_Cz_Hu_Sl_Li_Fi_Po_Ru     1
        EU836675_h_1_19_It                           1
        EU836674_h_1_18_At_It                        1
        EU836672_h_1_10_It                           1
        EU836671_h_1_09_It                           1
        EU836670_h_1_07_Gre                          1
        EU836669_h_1_06_Gre                          1
        EU836668_h_1_05_Gre                          1
        EU836667_h_1_04_Gre                          1
        EU836666_h_1_03_Mac                          1
        EU836665_h_1_02_Gre_Mac                      1
        EU836664_h_2_06_At_It                        1
        EU836663_h_2_05_It                           1

Parnassius_orleans_AC10_5                    ------------------------
TGAGCAGGTATAATAGGAACCTCTTTAAGATTATTAATTCGTACTGAATTAGGTAATCCTGGATCTTTAATTGGAGATGATCAAATTTATAACACTA
TTGTAACAGCTCATGCTTTTATTATAATTTTTTTTATAGTTATACCAATTATAATTGGAGGATTTGGAAATTGATTAATTCCATTAATATTAGGAGC
CCCAGATATAGCTTTCCCCCGAATAAATAATATAAGATTTTGACTATTACCCCCCTCATTAACCTTATTAATTTCCAGAAGAATCGTAGAAAATGGA
GCAGGAACTGGATGAACAGTTTATCCCCCTTTATCCTCTAATATTGCCCATAGTGGAAGATCAGTTGATTTAGCTATCTTTTCTTTACATTTAGCTG
```

GAATTTCATCTATTTTAGGAGCTATTAATTTTATTACAACTATTATTAATATACGAATTAATCATATATCATTTGATCAAATACCCCTTTTCGTATG
AGCAGTAGGAATTACTGCTTTACTTTTATTATTATCTTTACCTGTTTTAGCAGGTGCTATTACTATACTTCTTACAGATCGAAATCTTAATACTTCA
TTTTTTGATCCAGCAGGAGGTGGAGATCCTATTTTATACCAACATTTA

LOWAM001_Kyrgyzstan_Alai_10
ACACTATATTTTATTTTTGGTATTTGAGCAGGTATAGTAGGAACCTCTTTAAGATTATTAATTCGTACTGAATTAGGTAATCCTGGATCTTTAATTG
GAGATGATCAAATTTATAATACTATTGTAACAGCTCATGCTTTTATTATAATTTTTTTCATAGTTATACCAATTATAATTGGAGGATTTGGAAATTG
ATTAATTCCTTTAATATTAGGAGCCCCAGATATAGCTTTTCCTCGAATAAATAATATAAGATTTTGATTACTACCCCCTTCATTAACTTTATTAATT
TCTAGAAGAATTGTAGAAAATGGAGCAGGAACAGGATGAACAGTTTATCCCCCTTTATCATCTAATATCGCTCATAGAGGTAGTTCAGTTGATTTAG
CTATTTTTTCTTTACATTTAGCAGGAATTTCATCAATTTTAGGAGCTATTAATTTCATTACAACTATTATTAATATACGAATTAACCATATATCATT
TGATCAAATACCCCTCTTTGTATGAGCAGTAGGAATTACTGCTTTACTTTTATTATTATCTTTACCTGTATTAGCTGGTGCTATTACTATATTATTA
ACAGATCGAAATCTTAATACTTCATTTTTTGATCCTGCAGGAGGTGGAGATCCTATTTTATATCAACATTTA

LOWAM004_Uzbekistan_Gissar_38
ACACTATATTTTATTTTTGGTATTTGAGCAGGTATAGTAGGAACCTCTTTAAGATTATTAATTCGTACTGAATTAGGTAATCCTGGATCTTTAATTG
GAGATGATCAAATTTATAATACTATTGTAACAGCTCATGCTTTTATTATAATTTTTTTCATAGTTATACCAATTATAATTGGAGGATTTGGAAATTG
ATTAATTCCTTTAATATTAGGAGCCCCAGATATAGCTTTTCCTCGAATAAATAATATAAGATTTTGATTACTACCCCCTTCATTAACTTTATTAATT
TCCAGAAGAATTGTAGAAAATGGAGCAGGAACAGGATGAACAGTTTATCCCCCTTTATCATCTAATATCGCTCATAGAGGTAGTTCAGTTGATTTAG
CTATTTTTTCTTTACATTTAGCAGGAATTTCATCAATTTTAGGAGCTATTAATTTCATTACAACTATTATTAATATACGAATTAACCATATATCATT
TGATCAAATACCCCTCTTTGTATGAGCAGTAGGAATTACTGCTTTACTTTTATTATTATCTTTACCTGTATTAGCTGGTGCTATTACTATATTATTA
ACAGATCGAAATCTTAATACTTCATTTTTTGATCCTGCAGGAGGTGGAGATCCTATTTTATATCAACATTTA

LOWAM007_Kyrgyzstan_TianShan24
ACACTATATTTTATTTTTGGTATTTGAGCAGGTATAGTAGGAACCTCTTTAAGATTATTAATTCGTACTGAATTAGGTAATCCTGGATCTTTAATTG
GAGATGATCAAATTTATAATACTATTGTAACAGCTCATGCTTTTATTATAATTTTTTTTATAGTTATACCAATTATAATTGGAGGATTTGGAAATTG
ATTAATTCCTTTAATATTAGGGGCCCCAGATATAGCTTTTCCTCGAATAAATAATATAAGATTTTGATTATTACCCCCTTCATTAACTTTATTAATT
TCTAGAAGAATTGTAGAAAATGGAGCAGGAACAGGATGAACAGTTTATCCCCCTTTATCATCTAATATCGCTCATAGAGGTAGTTCAGTTGATTTAG
CTATTTTTTCTTTACATTTAGCAGGAATTTCATCAATCTTAGGAGCTATTAATTTTATTACAACTATTATTAATATACGAATTAATCATATATCATT
TGATCAAATACCTCTCTTTGTATGAGCAGTAGGAATTACTGCTTTACTTTTATTACTATCTTTACCTGTATTAGCTGGTGCTATTACTATATTATTA
ACAGATCGAAATCTTAATACTTCATTTTTTGATCCTGCAGGAGGTGGAGATCCTATTTTATATCAACATTTA

LOWAM009_Kyrgyzstan_TianShan_6
ACACTATATTTTATTTTTGGTATTTGAGCAGGTATAGTAGGAACCTCTTTAAGATTATTAATTCGTACTGAATTAGGTAATCCTGGATCTTTGATTG
GAGATGATCAAATTTATAATACTATTGTAACAGCTCATGCTTTTATTATAATTTTTTTTATAGTTATACCAATTATAATTGGAGGATTTGGAAATTG
ATTAATTCCTTTAATATTAGGGGCCCCAGATATAGCTTTTCCTCGAATAAATAATATAAGATTTTGATTATTACCCCCTTCATTAACTTTATTAATT
TCTAGAAGAATTGTAGAAAATGGAGCAGGAACAGGATGAACAGTTTATCCCCCTTTATCATCTAATATCGCTCATAGAGGTAGTTCAGTTGATTTAG
CTATTTTTTCTTTACATTTAGCAGGAATTTCATCAATCTTAGGAGCTATTAATTTTATTACAACTATTATTAATATACGAATTAATCATATATCATT
TGATCAAATACCTCTCTTTGTATGAGCAGTAGGAATTACTGCTTTACTTTTATTACTATCTTTACCTGTATTAGCTGGTGCTATTACTATATTATTA
ACAGATCGAAATCTTAATACTTCATTTTTTGATCCTGCAGGAGGTGGAGATCCTATTTTATATCAACATTTA

LOWAM013_Turkey_Georgia_3
ACACTATATTTTATTTTTGGTATTTGAGCAGGTATAGTAGGAACCTCTTTAAGATTATTAATTCGTACTGAATTAGGTAATCCTGGATCTTTAATTG
GAGATGATCAAATTTATAATACTATTGTAACAGCTCATGCTTTTATTATAATTTTTTTTATAGTTATACCAATTATAATTGGAGGATTTGGAAATTG
ATTAATTCCTTTAATATTAGGAGCCCCAGATATAGCTTTTCCTCGAATAAATAATATAAGATTTTGATTACTGCCCCCCTCATTAACTTTATTAATT
TCTAGAAGAATTGTAGAAAATGGAGCAGGAACAGGATGAACAGTTTATCCCCCTTTATCATCTAATATCGCTCATAGAGGTAGTTCAGTTGATTTAG
CTATTTTTTCTTTACATTTAGCAGGAATTTCATCAATCTTAGGAGCTATTAATTTTATTACAACTATTATTAATATACGAATTAACCATATATCATT
TGATCAAATACCTCTCTTTGTATGAGCAGTAGGAATTACTGCTTTACTTTTATTATTATCTTTACCTGTATTAGCTGGTGCTATTACTATATTATTA
ACAGATCGAAATCTTAATACTTCATTTTTTGATCCTGCAGGAGGTGGAGATCCTATTTTATATCAACATTTA

LOWAM014_Turkey_Ovitdagi_1
ACACTATATTTTATTTTTGGTATTTGAGCAGGTATAGTAGGAACCTCTTTAAGATTATTAATTCGTACTGAATTAGGTAATCCTGGATCTTTAATTG
GAGATGATCAAATTTATAATACTATTGTAACAGCTCATGCTTTTATTATAATTTTTTTTATAGTTATACCAATTATAATTGGAGGATTTGGAAATTG
ATTAATTCCTTTAATATTAGGAGCCCCAGATATAGCTTTTCCTCGAATAAATAATATAAGATTTTGATTACTGCCCCCCTCATTAACTTTATTAATT
TCCAGAAGAATTGTAGAAAATGGAGCAGGAACAGGATGAACAGTTTATCCCCCTTTATCATCTAATATCGCTCATAGAGGTAGTTCAGTTGATTTAG
CTATTTTTTCTTTACATTTAGCAGGAATTTCATCAATCTTAGGAGCTATTAATTTTATTACAACTATTATTAATATACGAATTAACCATATATCATT
TGATCAAATACCTCTCTTTGTATGAGCAGTAGGAATTACTGCTTTACTTTTATTATTATCTTTACCTGTATTAGCTGGTGCTATTACTATATTATTA
ACAGATCGAAATCTTAATACTTCATTTTTTGATCCTGCAGGAGGTGGAGATCCTATTTTATATCAACATTTA

LOWAM015_Don_Borisovka_Saratov
ACACTATATTTTATTTTTGGTATTTGAGCAGGTATAGTAGGAACCTCTTTAAGATTATTAATTCGTACTGAATTAGGTAATCCTGGATCTTTAATTG
GAGATGATCAAATTTATAATACTATTGTAACAGCTCATGCTTTTATTATAATTTTTTTTATAGTTATACCAATTATAATTGGAGGATTTGGAAATTG
ATTAATTCCTTTAATATTAGGAGCCCCAGATATAGCTTTTCCTCGAATAAATAATATAAGATTTTGATTACTACCCCCCTCATTAACTTTATTAATT
TCTAGAAGAATTGTAGAAAATGGAGCAGGAACAGGATGAACAGTTTATCCCCCTTTATCATCTAATATCGCTCATAGAGGTAGTTCAGTTGATTTAG
CTATTTTTTCTTTACATTTAGCAGGAATTTCATCAATCTTAGGAGCTATTAATTTTATTACAACTATTATTAATATACGAATTAACCATATATCATT
TGATCAAATACCTCTCTTTGTATGAGCAGTAGGAATTACTGCTTTACTTTTATTATTATCTTTACCTGTATTAGCTGGTGCTATTACTATATTATTA
ACAGATCGAAATCTTAATACTTCATTTTTTGATCCTGCAGGAGGTGGGGATCCTATTTTATATCAACATTTA

LOWAM018_Kyrgyzstan_Karamyk
ACACTATATTTTATTTTTGGTATTTGAGCAGGTATAGTAGGAACCTCTTTAAGATTATTAATTCGTACTGAATTAGGTAATCCTGGATCTTTAATTG
GAGATGATCAAATTTATAATACTATTGTAACAGCTCATGCTTTTATTATAATTTTTTTTATAGTTATACCAATTATAATTGGAGGATTTGGAAATTG
ATTAATTCCTTTAATATTAGGAGCCCCAGATATAGCTTTTCCTCGAATAAATAATATAAGATTTTGATTACTACCCCCTTCATTAACTTTATTAATT
TCTAGAAGAATTGTAGAAAATGGAGCAGGAACAGGATGAACAGTTTATCCCCCTTTATCATCTAATATCGCTCATAGAGGTAGTTCAGTTGATTTAG
CTATTTTTTCTTTACATTTAGCAGGAATTTCATCAATTTTAGGAGCTATTAATTTCATTACAACTATTATTAATATACGAATTAACCATATATCATT
TGATCAAATACCCCTCTTTGTATGAGCAGTAGGAATTACTGCTTTACTTTTATTATTATCTTTACCTGTATTAGCTGGTGCTATTACTATATTATTA
ACAGATCGAAATCTTAATACTTCATTTTTTGATCCAGCAGGAGGTGGAGATCCTATTTTATATCAACATTTA

LOWAM019_Kyrgyzstan_Karamyk
ACACTATATTTTATTTTTGGTATTTGAGCAGGTATAGTAGGAACCTCTTTAAGATTATTAATTCGTACTGAATTAGGTAATCCTGGATCTTTAATTG
GAGATGATCAAATTTATAATACTATTGTAACAGCTCATGCTTTTATTATAATTTTTTTCATAGTTATACCAATTATAATTGGAGGATTTGGAAATTG
ATTAATTCCTTTAATATTAGGAGCCCCAGATATAGCTTTTCCTCGAATAAATAATATAAGATTTTGATTACTACCCCCTTCATTAACTTTATTAATT

TCTAGAAGAATTGTAGAAAATGGAGCAGGAACAGGATGAACA?TTTATCCCCCTTTATCATCTAATATCGCTCATAGAGGTAGTTCAGTTGATTTAG
CTATTTTTTCTTTACATTTAGCAGGAATTTCATCAATTTTAGGGGCTATTAATTTCATTACAACTATTATTAATATACGAATTAACCATATATCATT
TGATCAAATACCCCTCTTTGTATGAGCAGTAGGAATTACTGCTTTACTTTTATTATTATCTTTACCTGTATTAGCTGGTGCTATTACTATATTATTA
ACAGATCGAAATCTTAATACTTCATTTTTTGATCCTGCAGGAGGTGGAGATCCTATTTTATATCAACATTTA

LOWAM021_Russia_Saratov
ACACTATATTTTATTTTTGGTATTTGAGCAGGTATAGTAGGAACCTCTTTAAGATTATTAATTCGTACTGAATTAGGTAATCCTGGATCTTTAATTG
GAGATGATCAAATTTATAATACTATTGTAACAGCTCATGCTTTTATTATAATTTTTTTTATAGTTATACCAATTATAATTGGAGGATTTGGAAATTG
ATTAATTCCTTTAATATTAGGAGCCCCAGATATAGCTTTTCCTCGAATAAATAATATAAGATTTTGATTACTACCCCCCTCATTAACTTTATTAATT
TCTAGAAGAATTGTAGAAAATGGAGCAGGAACAGGATGAACAGTTTATCCCCCTTTATCATCTAATATCGCTCATAGAGGTAGTTCAGTTGATTTAG
CTATTTTTTCTTTACATTTAGCAGGAATTTCATCAATCTTAGGAGCTATTAATTTTATTACAACTATTATTAATATACGAATTAACCATATATCATT
TGATCAAATACCTCTCTTTGTATGAGCAGTAGGAATTACTGCTTTACTTTTATTATTATCTTTACCTGTATTAGCTGGTGCTATTACTATATTATTA
ACAGATCGAAATCTTAATAATTCATTTTTTGATCCTGCAGGAGGTGGGGATCCTATTTTATATCAACATTTA

LOWAM023_Saratov_Luga_Vologda_4
ACACTATATTTTATTTTTGGTATTTGAGCAGGTATAGTAGGAACCTCTTTAAGATTATTAATTCGTACTGAATTAGGTAATCCTGGATCTTTAATTG
GAGATGATCAAATTTATAATACTATTGTAACAGCTCATGCTTTTATTATAATTTTTTTTATAGTTATACCAATTATAATTGGAGGATTTGGAAATTG
ATTAATTCCTTTAATATTAGGAGCCCCAGATATAGCTTTTCCTCGAATAAATAATATAAGATTTTGATTACTACCCCCCTCATTAACTTTATTAATT
TCTAGAAGAATTGTAGAAAATGGAGCAGGAACAGGATGAACAGTTTATCCCCCTTTATCATCTAATATCGCTCATAGAGGTAGTTCAGTTGATTTAG
CTATTTTTTCTTTACATTTAGCAGGAATTTCATCAATCTTAGGAGCTATTAATTTTATTACAACTATTATTAATATACGAATTAACCATATATCATT
TGATCAAATACCTCTCTTTGTATGAGCAGTAGGAATTACTGCTTTACTTTTATTATTATCTTTACCTGTATTAGCTGGTGCTATTACTATATTATTA
ACAGATCGAAATCTTAATACTTCATTTTTTGATCCTGCAGGAGGTGGAGATCCTATTTTATATCAACATTTA

LOWAM027_Kazakhstan_Karatau_2
ACACTATATTTTATTTTTGGTATTTGAGCAGGTATAGTAGGAACCTCTTTAAGATTATTAATTCGTACTGAATTAGGTAATCCTGGATCTTTAATTG
GAGATGATCAAATTTATAATACTATTGTAACAGCTCATGCTTTTATTATAATTTTTTTTATAGTTATACCAATTATAATTGGAGGATTTGGAAATTG
ATTAATTCCTTTAATATTAGGGGCCCCAGATATAGCTTTTCCTCGAATAAATAATATAAGATTTTGATTATTACCCCCTTCATTAACTTTATTAATT
TCTAGAAGAATTGTAGAAAATGGAGCAGGAACAGGATGAACAGTTTATCCCCCTTTATCATCTAATATCGCTCATAGAGGTAGTTCAGTTGATTTAG
CTATTTTTTCTTTACATTTAGCAGGAATTTCATCAATCTTAGGAGCTATTAATTTTATTACAACTATTATTAATATACGAATTAATCATATATCATT
TGATCAAATACCTCTCTTTGTATGAGCAGTAGGAATTACTGCTTTACTTTTATTACTATCCTTACCTGTATTAGCTGGTGCTATTACTATATTATTA
ACAGATCGAAATCTTAATACTTCATTTTTTGATCCTGCAGGAGGTGGAGATCCTATTTTATATCAACATTTA

LOWAM037_Russia_Pskovskaya ---------
TTTATTTTTGGTATTTGAGCAGGTATAGTAGGAACCTCTTTAAGATTATTAATTCGTACTGAATTAGGTAATCCTGGATCTTTAATTGGAGATGATC
AAATTTATAATACTATTGTAACAGCTCATGCTTTTATTATAATTTTTTTTATAGTTATACCAATTATAATTGGAGGATTTGGAAATTGATTAATTCC
TTTAATATTAGGAGCCCCAGATATAGCTTTTCCTCGAATAAATAATATAAGATTTTGATTACTACCCCCCTCATTAACTTTATTAATTTCTAGAAGA
ATTGTAGAAAATGGAGCAGGAACAGGATGAACAGTTTATCCCCCTTTATCATCTAATATCGCTCATAGAGGTAGTTCAGTTGATTTAGCTATTTTTT
CTTTACATTTAGCAGGAATTTCATCAATCTTAGGAGCTATTAATTTTATTACAACTATTATTAATATACGAATTAACCATATATCATTTGATCAAAT
ACCTCTCTTTGTATGAGCAGTAGGAATTACTGCTTTACTTTTATTATTATCTTTACCTGTATTAGCTGGTGCTATTACTATATTATTAACAGATCGA
AATCTTAATACTTCATTTTTTGATCCTGCAGGAGGTGGAGATCCTATTTTATATCAACATTTA

LOWAM038_Kyrgyzstan_Tjuz_Ashu_3
ACACTATATTTTATTTTTGGTATTTGAGCAGGTATAGTAGGAACCTCTTTAAGATTATTAATTCGTACTGAATTAGGTAATCCTGGATCTTTAATTG
GAGATGATCAAATTTATAATACTATTGTAACAGCTCATGCTTTTATTATAATTTTTTTTATAGTTATACCAATTATAATTGGAGGATTTGGAAATTG
ATTAATTCCTTTAATATTAGGGGCCCCAGATATAGCTTTTCCTCGAATAAATAATATAAGATTTTGATTATTACCCCCTTCATTAACTTTATTAATT
TCTAGAAGAATTGTAGAAAATGGAGCAGGAACAGGATGAACAATTTATCCCCCTTTATCATCTAATATCGCTCATAGAGGTAGTTCAGTTGATTTAG
CTATTTTTTCTTTACATTTAGCGGGAATTTCATCAATCTTAGGAGCTATTAATTTTATTACAACTATTATTAATATACGAATTAATCATATATCATT
TGATCAAATACCTCTCTTTGTATGAGCAGTAGGAATTACTGCTTTACTTTTATTACTATCTTTACCTGTATTAGCTGGTGCTATTACTATATTATTA
ACAGATCGAAATCTTAATACTTCATTTTTTGATCCTGCAGGAGGTGGAGATCCTATTTTATATCAACATTTA

LOWAM039_Kyrgyzstan_23
ACACTATATTTTATTTTTGGTATTTGAGCAGGTATAGTAGGAACCTCTTTAAGATTATTAATTCGTACTGAATTAGGTAATCCTGGATCTTTAATTG
GAGATGATCAAATTTATAATACTATTGTAACAGCTCATGCTTTTATTATAATTTTTTTTATAGTTATACCAATTATAATTGGAGGATTTGGAAATTG
ATTAATTCCTTTAATATTAGGGGCCCCAGATATAGCTTTTCCTCGAATAAATAATATAAGATTTTGATTATTACCCCCTTCATTAACTTTATTAATT
TCTAGAAGAATTGTAGAAAATGGAGCAGGAACAGGATGAACAGTTTATCCCCCTTTATCATCTAATATCGCTCATAGAGGTAGTTCAGTTGATTTAG
CTATTTTTTCTTTACATTTAGCGGGAATTTCATCAATCTTAGGAGCTATTAATTTTATTACAACTATTATTAATATACGAATTAATCATATATCATT
TGATCAAATACCTCTCTTTGTATGAGCAGTAGGAATTACTGCTTTACTTTTATTACTATCTTTACCTGTATTAGCTGGTGCTATTACTATATTATTA
ACAGATCGAAATCTTAATACTTCATTTTTTGATCCTGCAGGAGGTGGAGATCCTATTTTATATCAACATTTA

LOWAM044_Russia_Borisovra_3
ACACTATATTTTATTTTTGGTATTTGAGCAGGTATAGTAGGAACCTCTTTAAGATTATTAATTCGTACTGAATTAGGTAATCCTGGATCTTTAATTG
GAGATGATCAAATTTATAATACTATTGTAACAGCTCATGCTTTTATTATAATTTTTTTTATAGTTATACCAATTATAATTGGAGGATTTGGAAATTG
ATTAATTCCCTTAATATTAGGAGCCCCAGATATAGCTTTTCCTCGAATAAATAATATAAGATTTTGATTACTACCCCCCTCATTAACTTTATTAATT
TCTAGAAGAATTGTAGAAAATGGAGCAGGAACAGGATGAACAGTTTATCCCCCTTTATCATCTAATATCGCTCATAGAGGTAGTTCAGTTGATTTAG
CTATTTTTTCTTTACATTTAGCAGGAATTTCATCAATCTTAGGAGCTATTAATTTTATTACAACTATTATTAATATACGAATTAACCATATATCATT
TGATCAAATACCTCTCTTTGTATGAGCAGTAGGAATTACTGCTTTACTTTTATTATTATCTTTACCTGTATTAGCTGGTGCTATTACTATATTATTA
ACAGATCGAAATCTTAATACTTCATTTTTTGATCCTGCAGGAGGTGGAGATCCTATTTTATATCAACATTTA

LOWAM045_Tajikistan_Shakhristan_3
ACACTATATTTTATTTTTGGTATTTGAGCAGGTATAGTAGGAACCTCTTTAAGATTATTAATTCGTACTGAATTAGGTAATCCTGGATCTTTAATTG
GAGATGATCAAATTTATAATACTATTGTAACAGCTCATGCTTTTATTATAATTTTTTTCATAGTTATACCAATTATAATTGGAGGATTTGGAAATTG
ATTAATTCCTTTAATATTAGGAGCCCCAGATATAGCTTTTCCTCGAATAAATAATATAAGATTTTGATTACTACCCCCTTCATTAACTTTATTAATT
TCCAGAAGAATTGTAGAAAATGGAGCAGGAACAGGATGAACAGTTTATCCCCCTTTATCATCTAATATTGCTCATAGAGGTAGTTCAGTTGATTTAG
CTATTTTTTCTTTACATTTAGCAGGAATTTCATCAATTTTAGGAGCTATTAATTTCATTACAACTATTATTAATATACGAATTAACCATATATCATT
TGATCAAATACCCCTCTTTGTATGAGCAGTAGGAATTACTGCTTTACTTTTATTATTATCTTTACCTGTATTAGCTGGTGCTATTACTATATTATTA
ACAGATCGAAATCTTAATACTTCATTTTTTGATCCTGCAGGAGGTGGAGATCCTATTTTATATCAACATTTA

LOWAM067_Kazakhstan_Kaindy_1
ACACTATATTTTATTTTTGGTATTTGAGCAGGTATAGTAGGAACCTCTTTAAGATTATTAATTCGTACTGAATTAGGTAATCCTGGATCTTTAATTG
GAGATGATCAAATTTATAATACTATTGTAACAGCTCATGCTTTTATTATAATTTTTTTTATAGTTATACCAATTATAATTGGAGGATTTGGAAATTG

ATTAATTCCTTTAATATTAGGGGCCCCAGATATAGCTTTTCCTCGAATAAATAATATAAGATTTTGATTATTACCCCCTTCATTAACTTTATTAATT
TCTAGAATAATTGTAGAAAATGGAGCAGGAACAGGATGAACAGTTTATCCCCCTTTATCATCTAATATCGCTCATAGAGGTAGTTCAGTTGATTTAG
CTATTTTTTCTTTACATTTAGCGGGAATTTCATCAATCTTAGGAGCTATTAATTTTATTACAACTATTATTAATATACGAATTAATCATATATCATT
TGATCAAATACCTCTCTTTGTATGAGCAGTAGGAATTACTGCTTTACTTTTATTACTATCTTTACCTGTATTAGCTGGTGCTATTACTATATTATTA
ACAGATCGAAATCTTAATACTTCATTTTTTGATCCTGCAGGAGGTGGAGATCCTATTTTATATCAACATTTA

LOWAM069_Russia_Teberda_2 ---------
TTTATTTTTGGTATTTGAGCAGGTATAGTAGGAACCTCTTTAAGATTATTAATTCGTACTGAATTAGGTAATCCTGGATCTTTAATTGGAGATGATC
AAATTTATAATACTATTGTAACAGCTCATGCTTTTATTATAATTTTTTTTATAGTTATACCAATTATAATTGGAGGATTTGGAAATTGATTAATTCC
TTTAATATTAGGAGCCCCAGATATAGCTTTTCCTCGAATAAATAATATAAGATTTTGATTACTGCCCCCCTCATTAACTTTATTAATTTCTAGAAGA
ATTGTAGAAAATGGAGCAGGAACAGGATGAACAGTTTATCCCCCTTTATCATCTAATATCGCTCATAGAGGTAGTTCAGTTGATTTAGCTATTTTTT
CTTTACATTTAGCAGGAATTTCATCAATCTTAGGAGCTATTAATTTTATTACAACTATTATTAATATACGAATTAATCATATATCATTTGATCAAAT
ACCTCTCTTTGTATGAGCAGTAGGAATTAC?GCTTTACTTTTATTATTATCTTTACCTGTATTAGCTGG?GCTATTACTATATTATTAACAGATCGA
AATCT?AATACTTCATTTTTTGATCCTGCAGGAGGAGGAGATCCTATTTTATATCAACATTTA

LOWAM073_Kyrgyzstan_Takhtalyk1
ACACTATATTTTATTTTTGGTATTTGAGCAGGTATAGTAGGAACCTCTTTAAGATTATTAATTCGTACTGAATTAGGTAATCCTGGATCTTTAATTG
GAGATGATCAAATTTATAATACTATTGTAACAGCTCATGCTTTTATTATAATTTTTTTTATAGTTATACCAATTATAATTGGAGGATTTGGAAATTG
ATTAATTCCTTTAATATTAGGGGCCCCAGATATAGCTTTTCCTCGAATAAATAATATAAGATTTTGATTATTACCCCCTTCATTAACTTTATTAATT
TCTAGAAGAATTGTAGAAAATGGAGCAGGAACAGGATGAACAGTTTATCCCCCTTTATCATCTAATATCGCTCATAGAGGTAGTTCAGTTGATTTAG
CTATTTTTTCTTTACATTTAGCGGGGATTTCATCAATCTTAGGAGCTATTAATTTTATTACAACTATTATTAATATACGAATTAATCATATATCATT
TGATCAAATACCTCTCTTTGTATGAGCAGTAGGAATTACTGCTTTACTTTTATTATTATCTTTACCTGTATTAGCTGGTGCTATTACTATATTATTA
ACAGATCGAAATCTTAATACTTCATTTTTTGATCCTGCAGGAGGTGGAGATCCTATTTTATATCAACATTTA

LOWAM074_Kyrgyzstan_Naryntoo
ACACTATATTTTATTTTTGGTATTTGAGCAGGTATAGTAGGAACCTCTTTAAGATTATTAATTCGTACTGAATTAGGTAATCCTGGATCTTTAATTG
GAGATGATCAAATTTATAATACTATTGTAACAGCTCATGCTTTTATTATAATTTTTTTTATAGTTATACCAATTATAATTGGAGGATTTGGAAATTG
ATTAATTCCTTTAATATTAGGGGCCCCAGATATAGCTTTTCCTCGAATAAATAATATAAGATTTTGATTATTACCCCCTTCATTAACTTTATTAATT
TCTAGAAGAATTGTAGAAAATGGAGCAGGAACAGGATGAACAGTTTATCCCCCTTTATCATCTAATATCGCTCATAGAGGTAGTTCAGTTGATTTAG
CTATTTTTTCTTTACATTTAGCGGGAATTTCATCAATCTTAGGAGCTATTAATTTTATTACAACTATTATTAATATACGAATTAATCATATATCATT
TGATCAAATACCTCTCTTTGTATGAGCAGTAGGAATTACTGCTTTACTTTTATTACTATCTTTACCTGTATTAGCTGGTGCTATTACTATATTATTA
ACAGATCGAAATCTTAATACTTCATTTTTTGATCCTGCAGGAGGTGGAGATCCTATTTTATATCAACATTTA

LOWAM086_Turkmenistan_3
ACATTATATTTTATTTTTGGTATTTGAGCAGGTATAGTAGGAACTTCCTTAAGATTATTAATTCGTACTGAATTAGGTAATCCTGGATCTTTAATTG
GAGATGATCAAATTTATAATACTATTGTAACAGCTCATGCTTTTATTATAATTTTTTTTATAGTTATACCAATTATAATTGGAGGATTTGGAAATTG
ATTAATTCCTTTAATATTAGGAGCCCCAGATATAGCTTTCCCCCGAATAAATAATATAAGATTTTGATTATTACCCCCCTCATTAACTTTATTAATT
TCTAGAAGAATTGTAGAAAATGGAGCAGGAACTGGATGAACAGTTTATCCCCCTTTATCATCTAATATTGCCCATAGAGGTAGTTCAGTTGATTTAG
CTATCTTTTCTTTACATTTAGCAGGAATTTCATCAATTTTAGGAGCTATTAATTTTATTACAACTATTATTAATATACGAATTAATCATATATCATT
TGATCAAATACCTCTTTTTGTATGAGCAGTAGGAATTACTGCTTTACTTTTATTACTATCTTTACCTGTATTAGCTGGTGCTATTACTATATTATTA
ACAGATCGAAATCTTAATACCTCATTTTTTGATCCAGCAGGAGGTGGAGATCCTATTTTATATCAACATTTA

LOWAM087_Uzbekistan_Sarchashma
ACACTATATTTTATTTTTGGTATTTGAGCAGGTATAGTAGGAACCTCTTTAAGATTATTAATTCGTACTGAATTAGGTAATCCTGGATCTTTAATTG
GAGATGATCAAATTTATAATACTATTGTAACAGCTCATGCTTTTATTATAATTTTTTTCATAGTTATACCAATTATAATTGGAGGATTTGGAAATTG
ATTAATTCCTTTAATATTAGGAGCCCCAGATATAGCTTTTCCTCGAATAAATAATATAAGATTTTGATTACTACCCCCTTCATTAACTTTATTAATT
TCCAGAAGAATTGTAGAAAATGGAGCAGGAACAGGATGAACAGTTTATCCCCCTTTATCATCTAATATCGCTCATAGAGGTAGTTCAGTTGATTTAG
CTATTTTTTCTTTACATTTAGCAGGAATTTCATCAATTTTAGGAGCTATTAATTTCATTACAACTATTATTAATATACGAATTAACCATATATCATT
TGATCAAATACCCCTCTTTGTATGAGCAGTAGGAATTACTGCTTTACTTTTATTATTATCTTTACCTGTATTAGCTGGTGCTATTACTATATTATTA
ACAGATCGAAATCTTAATACTTCATTTTTTGATCCTGCAGGAGGTGGAGATCCTATTTA??ATCAACATTTA

LOWAM095_Kyrgyzstan_Sosnovka
ACACTATATTTTATTTTTGGTATTTGAGCAGGTATAGTAGGAACCTCTTTAAGATTATTAATTCGTACTGAATTAGGTAATCCTGGATCTTTAATTG
GAGATGATCAAATTTATAATACTATTGTAACAGCTCATGCTTTTATTATAATTTTTTTTATAGTTATACCAATTATAATTGGAGGATTTGGAAATTG
ATTAATTCCTTTAATATTAGGGGCTCCAGATATAGCTTTTCCTCGAATAAATAATATAAGATTTTGATTATTACCCCCTTCATTAACTTTATTAATT
TCTAGAAGAATTGTAGAAAATGGAGCAGGAACAGGATGAACAGTTTATCCCCCTTTATCATCTAATATCGCTCATAGAGGTAGTTCAGTTGATTTAG
CTATTTTTTCTTTACATTTAGCGGGAATTTCATCAATCTTAGGAGCTATTAATTTTATTACAACTATTATTAATATACGAATTAATCATATATCATT
TGATCAAATACCTCTCTTTGTATGAGCAGTAGGAATTACTGCTTTACTTTTATTACTATCTTTACCTGTATTAGCTGGTGCTATTACTATATTATTA
ACAGATCGAAATCTTAATACTTCATTTTTTGATCCTGCAGGAGGTGGAGATCCTATTTTATATCAACATTTA

LOWAM096_Gissar_5
ACACTATATTTTATTTTTGGTATTTGAGCAGGTATAGTAGGAACCTCTTTAAGATTATTAATTCGTACTGAATTAGGTAATCCTGGATCTTTAATTG
GAGATGATCAAATTTATAATACTATTGTAACAGCTCATGCTTTTATTATAATTTTTTTCATAGTTATACCAATTATAATTGGAGGATTTGGAAATTG
ATTAATTCCTTTAATATTAGGAGCCCCAGATATAGCTTTTCCTCGAATAAATAATATAAGATTTTGATTACTACCCCCTTCATTAACTTTATTAATT
TCCAGAAGAATTGTAGAAAATGGAGCAGGAACAGGATGAACAGTTTATCCCCCTTTATCGTCTAATATCGCTCATAGAGGTAGTTCAGTTGATTTAG
CTATTTTTTCTTTACATTTAGCAGGAATTTCATCAATTTTAGGAGCTATTAATTTCATTACAACTATTATTAATATACGAATTAACCATATATCATT
TGATCAAATACCCCTCTTTGTATGAGCAGTAGGAATTACTGCTTTACTTTTATTATTATCTTTACCTGTATTAGCTGGTGCTATTACTATATTATTA
ACAGATCGAAATCTTAATACTTCATTTTTTGATCCTGCAGGAGGTGGAGATCCTATTTTATATCAACATTTA

LOWAM098_Tajikistan_Khondiza1
ACACTATATTTTATTTTTGGTATTTGAGCAGGTATAATAGGAACCTCTTTAAGATTATTAATTCGTACTGAATTAGGTAATCCTGGATCTTTAATTG
GAGATGATCAAATTTATAATACTATTGTAACAGCTCATGCTTTTATTATAATTTTTTTCATAGTTATACCAATTATAATTGGAGGATTTGGAAATTG
ATTAATTCCTTTAATATTAGGAGCCCCAGATATAGCTTTTCCTCGAATAAATAATATAAGATTTTGATTACTACCCCCTTCATTAACTTTATTAATT
TCCAGAAGAATTGTAGAAAATGGAGCAGGAACAGGATGAACAGTTTATCCCCCTTTATCGTCTAATATCGCTCATAGAGGTAGTTCAGTTGATTTAG
CTATTTTTTCTTTACATTTAGCAGGAATTTCATCAATTTTAGGAGCTATTAATTTCATTACAACTATTATTAATATACGAATTAACCATATATCATT
TGATCAAATACCCCTCTTTGTATGAGCAGTAGGAATTACTGCTTTACTTTTATTATTATCTTTACCTGTATTAGCTGGTGCTATTACTATATTATTA
ACAGATCGAAATCTTAATACTTCATTTTTTGATCCTGCAGGAGGTGGAGATCCTATTTTATATCAACATTTA

LOWAM099_Uzbekistan_Kamchik_2
ACACTATATTTTATTTTTGGTATTTGAGCAGGTATAGTAGGAACCTCTTTAAGATTATTAATTCGTACTGAATTAGGTAATCCTGGATCTTTAATTG

GAGATGATCAAATTTATAATACTATTGTAACAGCTCATGCTTTTATTATAATTTTTTTTATAGTTATACCAATTATAATTGGAGGATTTGGAAATTG
ATTAATTCCTTTAATATTAGGGGCCCCAGATATAGCTTTTCCTCGAATAAATAATATAAGATTTTGATTATTACCCCCTTCATTAACTTTATTAATT
TCTAGAAGAATTGTAGAAAATGGAGCAGGAACAGGATGAACAGTTTATCCCCCTTTATCATCTAATATCGCTCATAGAGGTAGTTCAGTTGATTTAG
CTATTTTTTCTTTACATTTAGCAGGAATTTCATCAATTTTAGGAGCTATTAATTTTATTACAACTATTATTAATATACGAATTAATCATATATCATT
TGATCAAATACCTCTCTTTGTATGAGCAGTAGGAATTACTGCTTTACTTTTATTACTATCTTTACCTGTATTAGCTGGTGCTATTACTATATTATTA
ACAGATCGAAATCTTAATACTTCATTTTTTGATCCTGCAGGAGGTGGAGATCCTATTTTATATCAACATTA

LOWAM100_Kyrgyzstan_Konduk
ACACTATATTTTATTTTTGGTATTTGAGCAGGTATAGTAGGAACCTCTTTAAGATTATTAATTCGTACTGAATTAGGTAATCCTGGATCTTTAATTG
GAGATGATCAAATTTATAATACTATTGTAACAGCTCATGCTTTTATTATAATTTTTTTTATAGTTATACCAATTATAATTGGAGGATTTGGAAATTG
ATTAATTCCTTTAATATTAGGGGCCCCAGATATAGCTTTTCCTCGAATAAATAATATAAGATTTTGATTATTACCCCCTTCATTAACTTTATTAATT
TCTAGAAGAATTGTAGAAAATGGAGCAGGAACAGGATGAACAGTTTATCCCCCTTTATCATCTAATATCGCTCATAGAGGTAGTTCAGTTGATTTAG
CTATTTTTTCTTTACATTTAGCGGGAATTTCATCAATCTTAGGAGCTATTAATTTTATTACAACTATTATTAATATACGAATTAATCATATATCATT
TGATCAAATACCTCTCTTTGTATGAGCAGTAGGAATTACTGCTTTACTTTTATTACTATCTTTACCTGTATTAGCTGGTGCTATTACTATATTATTA
ACAGATCGAAATCTTAATACTTCATTTTTTGATCCTGCAGGAGGTGGAGATCCTATTTTATATCAACATTA

LOWAM115_Azerbaijan_Nyus_Nyus
ACACTATATTTTATTTTCGGTATTTGAGCAGGTATAGTAGGAACCTCTTTAAGATTATTAATTCGTACTGAATTAGGTAATCCCGGATCTTTAATTG
GAGATGATCAAATTTATAACACTATTGTAACAGCTCATGCTTTTATTATAATTTTTTTTATAGTTATACCAATTATAATTGGAGGATTTGGAAATTG
ATTAATTCCTTTAATATTAGGAGCTCCAGATATAGCTTTTCCTCGAATAAATAATATAAGATTTTGATTACTCCCCCCCTCATTAACTTTATTAATT
TCTAGAAGAATTGTAGAAAATGGAGCAGGAACAGGATGAACAATTTATCCCCCTTTATCATCTAATATTGCTCATAGAGGTAGTTCAGTTGATTTAG
CTATTTTTTCTTTACATTTAGCAGGAATTTCATCAATCTTAGGAGCTATTAATTTTATTACAACTATTATTAACATACGAATTAATCATATATCATT
TGATCAAATACCCCTTTTTGTATGAGCAGTAGGAATTACTGCATTACTTTTATTATTATCTTTACCTGTATTAGCTGGTGCTATTACTATATTATTA
ACAGATCGAAATCTTAATACTTCATTTTTTGATCCTGCAGGAGGTGGAGATCCTATTTTATATCAACACTTA

LOWAM116_Iran_Dizin_2
ACACTATATTTTATTTTCGGTATTTGAGCAGGTATAGTAGGAACCTCTTTAAGATTATTAATTCGTACTGAATTAGGTAATCCTGGATCTTTAATTG
GAGATGATCAAATTTATAACACTATTGTAACAGCTCATGCTTTTATTATAATTTTTTTTATAGTTATACCAATTATAATTGGAGGATTTGGAAATTG
ATTAATTCCTTTAATATTAGGAGCTCCAGATATAGCTTTTCCTCGAATAAATAATATAAGATTTTGATTACTCCCCCCCTCATTAACTTTATTAATT
TCTAGAAGAATTGTAGAAAATGGAGCAGGAACAGGATGAACAATTTATCCCCCTTTATCATCTAATATTGCTCATAGAGGTAGTTCAGTTGATTTAG
CTATTTTTTCTTTACATTTAGCAGGAATTTCATCAATCTTAGGAGCTATTAATTTTATTACAACTATTATTAACATACGAATTAATCATATATCATT
TGATCAAATACCCCTTTTTGTATGAGCAGTAGGAATTACTGCATTACTTTTATTATTATCTTTACCTGTATTAGCTGGTGCTATTACTATATTATTA
ACAGATCGAAATCTTAATACTTCATTTTTTGATCCTGCAGGAGGTGGAGATCCTATTTTATATCAACACTTA

LOWAM122_Russia_Saratow_1
ACACTATATTTTATTTTTGGTATTTGAGCAGGTATAGTAGGAACCTCTTTAAGATTATTAATTCGTACTGAATTAGGTAATCCTGGATCTTTAATTG
GAGATGATCAAATTTATAACACTATTGTAACAGCTCATGCTTTTATTATAATTTTTTTTATAGTTATACCAATTATAATTGGAGGATTTGGAAATTG
ATTAATTCCTTTAATATTAGGAGCCCCAGATATAGCTTTTCCTCGAATAAATAATATAAGATTTTGATTACTACCCCCCTCATTAACTTTATTAATT
TCTAGAAGAATTGTAGAAAATGGAGCAGGAACAGGATGAACAGTTTATCCCCCTTTATCATCTAATATCGCTCATAGAGGTAGTTCAGTTGATTTAG
CTATTTTTTCTTTACATTTAGCAGGAATTTCATCAATCTTAGGAGCTATTAATTTTATTACAACTATTATTAATATACGAATTAACCATATATCATT
TGATCAAATACCTCTCTTTGTATGAGCAGTAGGAATTACTGCTTTACTTTTATTATTATCTTTACCTGTATTAGCTGGTGCTATTACTATATTATTA
ACAGATCGAAATCTTAATACTTCATTTTTTGATCCTGCAGGAGGTGGGGATCCTATTTTATATCAACATTA

LOWAM136_Uzbekistan_Tamshush_1
ACACTATATTTTATTTTTGGTATTTGAGCAGGTATAGTAGGAACCTCTTTAAGATTATTAATTCGTACTGAATTAGGTAATCCTGGATCTTTAATTG
GAGATGATCAAATTTATAATACTATTGTAACAGCTCATGCTTTTATTATAATTTTTTTCATAGTTATACCAATTATAATTGGAGGATTTGGAAATTG
ATTAATTCCTTTAATATTAGGAGCCCCAGATATAGCTTTTCCTCGAATAAATAATATAAGATTTTGATTACTACCCCCTTCATTAACTTTATTAATT
TCCAGAAGAATTGTAGAAAATGGAGCAGGAACAGGATGAACAGTTTATCCCCCTTTATCATCTAATATCGCTCATAGAGGTAGTTCAGTTGATTTAG
CTATTTTTTCTTTACATTTAGCAGGAATTTCATCAATTTTAGGAGCTATTAATTTCATTACAACTATTATTAATATACGAATTAACCATATATCATT
TGATCAAATACCCCTCTTTGTATGAGCAATAGGAATTACTGCTTTACTTTTATTATTATCTTTACCTGTATTAGCTGGTGCTATTACTATATTATTA
ACAGATCGAAATCTTAATACTTCATTTTTTGATCCTGCAGGAGGTGGAGATCCTATTTTATATCAACATTA

LOWAM149_Kyrgyzstan_Songkel_1
ACACTATATTTTATTTTTGGTATTTGAGCAGGTATAGTAGGAACCTCTTTAAGATTATTAATTCGTACTGAATTAGGTAATCCTGGATCTTTAATTG
GAGATGATCAAATTTATAATACTATTGTAACAGCTCATGCTTTTATTATAATTTTTTTTATAGTTATACCAATTATAATTGGAGGATTTGGAAATTG
ATTAATTCCTTTAATATTAGGGGCCCCAGATATAGCTTTTCCTCGAATAAATAATATAAGATTTTGATTATTACCCCCCTCATTAACTTTATTAATT
TCTAGAAGAATTGTAGAAAATGGAGCAGGAACAGGATGAACAGTTTATCCCCCTTTATCATCTAATATTGCTCATAGAGGTAGTTCAGTTGATTTAG
CTATTTTTTCTTTACATTTAGCGGGAATTTCATCAATCTTAGGAGCTATTAATTTTATTACAACTATTATTAATATACGAATTAATCATATATCATT
TGATCAAATACCTCTCTTTGTATGAGCAGTAGGAATTACTGCTTTACTTTTATTACTATCTTTACCTGTATTAGCTGGTGCTATTACTATATTATTA
ACAGATCGAAATCTTAATACTTCATTTTTTGATCCTGCAGGAGGTGGAGATCCTATTTTATATCAACATTA

LOWAM198_Turkey_Aladaglar_2
ACACTATATTTTATTTTTGGTATTTGAGCAGGTATAGTAGGAACCTCTTTAAGATTATTAATTCGTACTGAATTAGGTAATCCTGGATCTTTAATTA
GAGATGATCAAATTTATAATACTATTGTAACAGCTCATGCTTTTATTATAATTTTTTTTATAGTTATACCAATTATAATTGGAGGATTTGGAAATTG
ATTAATTCCTTTAATATTAGGAGCTCCAGATATAGCTTTTCCTCGAATAAATAATATAAGATTTTGATTACTTCCCCCTTCATTAACTTTATTAATT
TCTAGAAGAATTGTAGAAAATGGAGCAGGAACAGGATGAACAGTTTATCCCCCTTTATCATCTAATATTGCTCATAGAGGTAGTTCAGTTGATTTAG
CTATTTTTTCTTTACATTTAGCAGGAATTTCATCAATCTTAGGAGCTATTAATTTTATTACAACTATTATTAATATA--------------------
----

LOWAM213_Tajikistan_Tandukul_1
ACACTATATTTTATTTTTGGTATTTGAGCAGGTATAGTAGGAACCTCTTTAAGATTATTAATTCGTACTGAATTAGGTAATCCTGGATCTTTAATTG
GAGATGATCAAATTTATAATACTATTGTAACAGCTCATGCTTTTATTATAATTTTTTTCATAGTTATACCAATTATAATTGGAGGATTTGGAAATTG
ATTAATTCCTTTAATATTAGGAGCCCCAGATATAGCTTTTTCTCGAATAAATAATATAAGATTTTGATTACTACCCCCTTCATTAACTTTATTAATT
TCTAGAAGAATTGTAGAAAATGGAGCAGGAACAGGATGAACAGTTTATCCCCCTTTATTATCTAATATCGCTCATAGAGGTAGTTCAGTTGATTTAG
CTATTTTTTCTTTACATTTAGCAGGAATTTCATCAATTTTAGGAGCTATTAATTTCATTACAACTATTATTAATATACGAATTAACCATATATCATT
TGATCAAATACCCCTCTTTGTATGAGCAGTAGGAATTACTGCGTTACTTTTATTATTATCTTTACCTGTATTAGCTGGTGCTATTACTATATTATTA
ACAGATCGAAATCTTAATACTTCATTTTTTGATCCTGCAGGAGGTGGAGATCCTATTTTATATCAACATTA

Parnassius_clodius
ACATTATATTTTATTTTTGGTATTTGAGCAGGTATAGTAGGAACCTCTTTAAGATTATTAATTCGTACTGAATTAGGTAATCCTGGATCTTTAATTG
GAGATGATCAAATTTATAATACTATTGTAACAGCTCATGCTTTTATCATAATTTTTTTCATAGTTATACCAATTATAATTGGAGGATTTGGAAATTG
ATTAATTCCATTAATATTAGGAGCTCCAGATATAGCTTTTCCTCGAATAAATAATATAAGATTTTGATTATTACCCCCTTCATTAACTTTATTAATT
TCTAGAAGAATTGTACAAAATGGAGCAGGAACTGGATGAACAGTTTATCCCCCTTTATCATCTAATATTGCTCATAGAGGAAGATCAGTTGATTTAG
CTATTTTTTCTTTACATTTAGCTGGAATTTCATCTATCTTAGGAGCTATTAATTTTATTACAACTATTATTAATATACGAATTAATCATATATCATT
TGATCAAATACCCCTTTTTGTATGAGCAGTAGGAATTACCGCTTTACTTCTATTATTATCTTTACCTGTTTTAGCAGGTGCTATTACCATATTATTA
ACAGATCGAAATCTTAATACTTCATTTTTTGATCCAGCAGGAGGTGGAGATCCTATTTTATATCAACACTTA

Parnassius_eversmanni_2005_LOWA_108 ---------------------
ATTTGAGCAGGTATAGTAGGAACCTCTTTAAGATTATTAATTCGTTCTGAATTAGGTAATCCTGGATCTTTAATTGGAGATGATCAAATTTATAATA
CTATTGTAACAGCTCATGCTTTTATTATAATTTTTTTTATAGTTATACCAATTATAATTGGAGGATTTGGAAATTGATTAATTCCATTAATATTAGG
AGCTCCAGATATAGCTTTTCCTCGAATAAATAATATAAGATTTTGATTATTACCCCCCTCATTAACCTTACTAATTTCTAGAAGAATTGTAGAAAAT
GGAGCAGGAACTGGATGAACGGTTTATCCCCCTTTATCATCTAATATTGCTCATAGAGGAAGATCAGTTGATTTAGCTATCTTTTCTTTACATTTAG
CTGGAATTTCATCTATCTTAGGAGCTATTAATTTTATTACAACTATTATTAATATACGAATTAATCATATATCATTTGATCAAATACCCCTTTTTGT
ATGAGCAGTAGGAATTACTGCTTTACTTTTATTATTATCTTTACCTGTTTTAGCAGGTGCTATTACCATATTATTAACAGATCGAAATCTTAATACT
TCATTTTTTGACCCAGCAGGAGGTGGAGATCCTATTTTATATCAACATTTA

Parnassius_eversmanni_2005_LOWA_107 ---------------------
ATTTGAGCAGGTATAGTAGGAACCTCTTTAAGATTATTAATTCGTTCTGAATTAGGTAATCCTGGATCTTTAATTGGAGATGATCAAATTTATAATA
CTATTGTAACAGCTCATGCTTTTATTATAATTTTTTTTATAGTTATACCAATTATAATTGGAGGATTTGGAAATTGATTAATTCCATTAATATTAGG
AGCTCCAGATATAGCTTTTCCTCGAATAAATAATATAAGATTTTGATTATTACCCCCCTCATTAACCTTACTAATTTCTAGAAGAATTGTAGAAAAT
GGAGCAGGAACTGGATGAACGGTTTATCCCCCTTTATCATCTAATATTGCTCATAGAGGAAGATCAGTTGATTTAGCTATCTTTTCTTTACATTTAG
CTGGAATTTCATCTATCTTAGGAGCTATTAATTTTATTACAACTATTATTAATATACGAATTAATCATATATCATTTGATCAAATACCCCTTTTTGT
ATGAGCAGTAGGAATTACTGCTTTACTTTTATTATTATCTTTACCTGTTTTAGCAGGTGCTATTACCATATTATTAACAGATCGAAATCTTAATACT
TCATTTTTTGACCCAGCAGGAGGTGGAGATCCTATTTTATATCAACATTTA

Parnassius_stubbendorfii_2005_LOWA_815
ACATTATATTTTATTTTTGGTATTTGAGCAGGTATAGTAGGAACTTCTTTAAGATTATTAATTCGTACTGAATTAGGTAATCCTGGATCTTTAATTG
GAGATGATCAAATTTATAATACTATTGTAACAGCTCATGCTTTTATTATAATTTTTTTTATAGTTATACCAATTATAATTGGAGGATTTGGAAATTG
ATTAATTCCTTTAATATTAGGAGCCCCAGATATAGCTTTTCCTCGAATAAATAATATAAGATTTTGATTATTACCCCCTTCATTAACTTTACTAATT
TCCAGAAGAATTGTAGAAAATGGGGCAGGAACTGGATGAACAGTCTACCCTCCTTTATCATCTAATATTGCTCACGGAGGAAGATCTGTTGATTTAG
CTATTTTTTCCTTACATTTAGCGGGAATTTCATCTATTTTAGGAGCCATTAATTTTATTACAACTATTATTAATATACGAATTAATCATATATCATT
TGATCAAATACCTCTTTTTGTATGAGCAGTAGGAATTACTGCTTTACTTTTATTATTATCTCTACCTGTTTTAGCAGGTGCTATTACTATATTATTA
ACAGATCGAAATCTTAATACTTCATTTTTTGACCCAGCAGGAGGTGGAGATCCTATTTTATATCAACACTTA

Parnassius_stubbendorfii_2005_LOWA_154
ACATTATATTTTATTTTTGGTATTTGAGCAGGTATAGTAGGAACTTCTTTAAGATTATTAATTCGTACTGAATTAGGTAATCCTGGATCTTTAATTG
GAGATGATCAAATTTATAATACTATTGTAACAGCTCATGCTTTTATTATAATTTTTTTTATAGTTATACCAATTATAATTGGAGGATTTGGAAATTG
ATTAATTCCTTTAATATTAGGAGCCCCAGATATAGCTTTTCCTCGAATAAATAATATAAGATTTTGATTATTACCCCCTTCATTAACTTTACTAATT
TCCAGAAGAATTGTAGAAAATGGAGCAGGAACTGGATGAACAGTCTACCCTCCTTTATCATCTAATATTGCTCACGGAGGAAGATCTGTTGATTTAG
CTATTTTTTCCTTACATTTAGCGGGAATTTCATCTATTTTAGGAGCCATTAATTTTATTACAACTATTATTAATATACGAATTAATCATATATCATT
TGATCAAATACCTCTTTTTGTATGAGCAGTAGGAATTACTGCTTTACTTTTATTATTATCTCTACCTGTTTTAGCAGGTGCTATTACTATATTATTA
ACAGATCGAAATCTTAATACTTCATTTTTTGACCCAGCAGGAGGTGGAGATCCTATTTTATATCAACACTTA

Parnassius_stubbendorfii_2005_LOWA_153 -
CATTATATTTTATTTTTGGTATTTGAGCAGGTATAGTAGGAACTTCTTTAAGATTATTAATTCGTACTGAATTAGGTAATCCTGGATCTTTAATTGG
AGATGATCAAATTTATAATACTATTGTAACAGCTCATGCTTTTATTATAATTTTTTTTATAGTTATACCAATTATAATTGGAGGATTTGGAAATTGA
TTAATTCCTTTAATATTAGGAGCCCCAGATATAGCTTTTCCTCGAATAAATAATATAAGATTTTGATTATTACCCCCTTCATTAACTTTACTAATTT
CCAGAAGAATTGTAGAAAATGGAGCAGGAACTGGATGAACAGTCTACCCTCCTTTATCATCTAATATTGCTCACGGAGGAAGATCTGTTGATTTAGC
TATTTTTTCCTTACATTTAGCGGGAATTTCATCTATTTTAGGAGCCATTAATTTTATTACAACTATTATTAATATACGAATTAATCATATATCATTT
GATCAAATACCTCTTTTTGTATGAGCAGTAGGAATTACTGCTTTACTTTTATTATTATCTCTACCTGTTTTAGCAGGTGCTATTACTATATTATTAA
CAGATCGAAATCTTAATACTTCATTTTTTGACCCAGCAGGAGGTGGAGATCCTATTTTATATCAACACTTA

Parnassius_nordmanni
ACATTATATTTTATTTTTGGTATTTGAGCAGGTATAGTAGGAACTTCTTTAAGATTATTAATTCGTACTGAATTAGGTAATCCTGGATCTCTAATTG
GAGATGATCAAATTTACAATACTATCGTAACAGCTCATGCTTTTATTATAATTTTTTTCATAGTTATACCAATTATAATTGGAGGATTTGGAAATTG
ATTAATTCCATTAATATTAGGAGCTCCAGATATAGCTTTCCCCCGAATAAATAATATAAGATTTTGATTATTACCCCCCTCATTAACTCTATTAATT
TCTAGAAGAATTGTAGAAAATGGGGCAGGAACTGGATGAACAGTTTACCCCCCTTTATCATCTAATATTGCTCATAGAGGAAGATCAGTTGACTTAG
CTATTTTTTCTTTACATTTGGCTGGGATTTCTTCTATTTTAGGAGCTATTAATTTTATCACAACTATTGTTAATATACGAATTAACTATATATCATT
TGATCAAATACCTCTTTTCGTATGAGCAGTAGGAATTACTGCTTTACTTTTATTATTATCTTTACCTGTTTTAGCAGGTGCTATTACTATATTATTA
ACAGATCGAAATCTTAATACTTCATTTTTTGATCCAGCAGGAGG?GGAGACCCTATTCTATATCAA------

Parnassius_eversmanni
ACATTATATTTTATTTTTGGTATTTGAGCAGGTATAGTAGGAACCTCTTTAAGATTATTAATTCGTTCTGAATTAGGTAATCCTGGATCTTTAATTG
GAGATGATCAAATTTATAATACTATTGTAACAGCTCATGCTTTTATTATAATTTTTTTTATAGTTATACCAATTATAATTGGAGGATTTGGAAATTG
ATTAATTCCATTAATATTAGGAGCTCCAGATATAGCTTTTCCTCGAATAAATAATATAAGATTTTGATTATTACCCCCCTCATTAACCTTACTAATT
TCTAGAAGAATTGTAGAAAATGGAGCAGGAACTGGATGAACGGTTTATCCCCCTTTATCATCTAATATTGCTCATAGAGGAAGATCAGTTGATTTAG
CTATCTTTTCTTTACATTTAGCTGGAATTTCATCTATCTTAGGAGCTATTAATTTTATTACAACTATTATTAATATACGAATTAATCATATATCATT
TGATCAAATACCCCTTTTTGTATGAGCAGTAGGAATTACTGCTTTACTTTTATTATTATCTTTACCTGTTTTAGCAGGTGCTATTACCATATTATTA
ACAGATCGAAATCTTAATACTTCATTTTTTGACCCAGCAGGAGGTGGAGATCCTATTTTATATCAA------

Parnassius_eversmanni_felderi
ACATTATATTTTATTTTTGGTATTTGAGCAGGTATAGTAGGAACCTCTTTAAGATTATTAATTCGTTCTGAATTAGGTAATCCTGGATCTTTAATTG
GAGATGATCAAATTTATAATACTATTGTAACAGCTCATGCTTTTATTATAATTTTTTTTATAGTTATACCAATTATAATTGGAGGATTTGGAAATTG
ATTAATTCCATTAATATTAGGAGCTCCAGATATAGCTTTTCCTCGAATAAATAATATAAGATTTTGATTATTACCCCCCTCATTAACCTTACTAATT
TCTAGAAGAATTGTAGAAAATGGAGCAGGAACTGGATGAACGGTTTATCCCCCTTTATCATCTAATATTGCTCATAGAGGAAGATCAGTTGATTTAG
CTATCTTTTCTTTACA?TTAGCTGGAATTTCATCTATCTTAGGAGCTATTAATTTTATTACAACTATTATTAATATACGAATTAATCATATATCATT
TGATCAAATACCCCTTTTTGTATGAGCAGTAGGAATTACTGCTTTACTTTTATTATTATCTTTACCTGTTTTAGCAGGTGCTATTACCATATTATTA
ACAGATCGAAATCTTAATACTTCATTTTTTGACCCAGCAGGAGGTGGAGATCCTATTTTATATCAA------

Parnassius_ariadne_ariadne
ACATTATATTTTATTTTTGGTATTTGAGCAGGTATAGTAGGAACCTCTTTAAGATTATTAATTCGTACTGAATTAGGTAATCCTGGATCTTTAATTG
GAGATGATCAAATTTATAATACTATCGTAACAGCTCATGCTTTTATTATAATTTTTTTTATAGTTATACCAATTATAATTGGAGGATTTGGAAATTG
ATTAATTCCTTTAATATTAGGAGCTCCAGATATAGCCTTTCCTCGAATAAATAATATAAGATTTTGATTACTACCCCCCTCATTAACTTTATTAATC
TCTAGAAGAATTGTAGAAAATGGAGCAGGAACTGGATGAACAGTTTATCCCCCTTTATCATCTAATATTGCTCATAGAGGAAGTTCAGTTGATTTAG
CCATTTTCTCTTTACATTTAGCAGGAATTTCATCAATTTTAGGAGCTATTAATTTTATCACAACTATTATTAATATACGAATTAATCATATATCATT
TGATCAAATACCCCTTTTTGTTTGAGCAGTAGGAATTACTGCTTTACTATTATTATTATCTTTACCTGTATTAGCTGGTGCTATTACTATATTATTA
ACAGATCGAAATCTTAATACTTCATTTTTTGATCCAGCAGGA????GAGATCCTATTTTATATCAA------

Parnassius_glacialis_mikado
ACATTATATTTTATTTTTGGTATTTGAGCAGGTATAGTAGGAACCTCCTTAAGATTATTAATTCGTACTGAATTAGGTAATCCTGGATCTTTAATTG
GAGATGATCAAATTTATAATACTATTGTAACAGCTCATGCTTTTATTATAATTTTTTTTATAGTTATACCAATTATAATTGGAGGATTTGGAAATTG
ATTAATCCCTTTAATATTAGGAGCTCCAGATATAGCTTTCCCCCGAATAAATAATATAAGATTTTGATTATTACCCCCCTCATTAACTTTACTAATT
TCCAGAAGAATTGTAGAAAATGGAGCAGGAACAGGATGAACAGTTTATCCCCCATTATCCTCTAATATTGCCCACAGAGGAAGATCTGTTGATTTAG
CTATTTTTTCTTTACATTTAGCAGGAATTTCATCTATTCTAGGAGCTATTAATTTTATTACAACTATTATTAATATACGAATTAATCATATATCATT
TGATCAAATACCTCTCTTTGTTTGAGCAGTAGGAATTACTGCTTTACTTTTATTATTATCCCTACCTGTTTTAGCAGGTGCTATTACTATATTATTA
ACAGATCGAAATCTTAATACTTCTTTTTTTGACCCAGCAGGAGGTGGAGATCCAATTTTATATCAA------

Parnassius_stubbendorfii_hoenei
ACATTATATTTTATTTTTGGTATTTGAGCAGGAATAGTAGGAACCTCCCTAAGATTATTAATTCGTACTGAATTAGGTAATCCCGGATCTTTAATTG
GAGATGATCAAATTTACAATACTATTGTAACAGCTCATGCTTTCATTATAATTTTTTTTATAGTTATACCAATTATAATTGGAGGATTTGGAAATTG
ACTGATTCCTTTAATATTAGGAGCCCCAGATATAGCTTTCCCCCGAATAAATAATATAAGATTTTGATTACTACCCCCCTCATTAACTTTATTAATT
TCTAGAAGAATTGTAGAAAATGGAGCAGGAACTGGATGAACAGTCTATCCCCCTTTATCATCTAATATTGCCCATAGAGGAAGATCCGTTGATTTAG
CTATTTTTTCCTTACATTTAGCAGGAATTTCATCTATTTTAGGAGCCATTAATTTTATTACAACTATTATTAATATACGAATTAATCATATATCATT
TGATCAAATACCTCTTTTTGTATGAGCAGTAGGAATTACTGCTTTACTTTTATTATTATCTTTACCTGTTTTAGCAGGTGCTATTACTATATTATTA
ACAGATCGAAATCTTAATACTTCCTTTTTTGACCCAGCAGGAGGTGGAGATCCTATTTTATACCAA------

Parnassius_stubbendorfii_hoenei_AC20_16 ------------------------
TGAGCAGGAATAGTAGGAACCTCCCTAAGATTATTAATTCGTACTGAATTAGGTAATCCCGGATCTTTAATTGGAGATGATCAAATTTACAATACTA
TTGTAACAGCTCATGCTTTCATTATAATTTTTTTTATAGTTATACCAATTATAATTGGAGGATTTGGAAATTGACTGATTCCTTTAATATTAGGAGC
CCCAGATATAGCTTTCCCCCGAATAAATAATATAAGATTTTGATTACTACCCCCCTCATTAACTTTATTAATTTCTAGAAGAATTGTAGAAAATGGA
GCAGGAACTGGATGAACAGTCTATCCCCCTTTATCATCTAATATTGCCCATAGAGGAAGATCCGTTGATTTAGCTATTTTTTCCTTACATTTAGCAG
GAATTTCATCTATTTTAGGAGCCATTAATTTTATTACAACTATTATTAATATACGAATTAATCATATATCATTTGATCAAATACCTCTTTTTGTATG
AGCAGTAGGAATTACTGCTTTACTTTTATTATTATCTTTACCTGTTTTAGCAGGTGCTATTACTATATTATTAACAGATCGAAATCTTAATACTTCC
TTTTTTGACCCCGCAGGAGGTGGAGATCCTATTTTATACCAACATTTA

Parnassius_stubbendorfii_koreanus ------------------------
TGAGCAGGTATAGTAGGAACTTCTTTAAGATTATTAATTCGTACTGAATTAGGTAATCCTGGATCTTTAATTGGAGATGATCAAATTTATAATACTA
TTGTAACAGCTCATGCTTTTATTATAATTTTTTTTATAGTTATACCAATTATAATTGGAGGATTTGGAAATTGATTAATTCCTTTAATATTAGGAGC
CCCAGATATAGCTTTTCCTCGAATAAATAATATAAGATTTTGACTATTACCCCCCTCATTAACTTTACTAATTTCCAGAAGAATTGTAGAAAATGGA
GCAGGAACTGGATGAACAGTCTACCCTCCTTTATCATCTAATATTGCTCACGGAGGAAGATCTGTTGATTTAGCTATTTTTTCCTTACATTTAGCAG
GAATTTCATCTATTTTAGGAGCCATTAATTTTATTACAACTATTATTAATATACGAATTAATCATATATCATTTGATCAAATACCTCTTTTTGTATG
AGCAGTAGGAATTACTGCTTTACTTTTATTATTATCTCTACCTGTTTTAGCAGGTGCTATTACTATATTATTAACAGATCGAAATCTTAATACTTCA
TTTTTTGACCCAGCAGGAGGTGGAGATCCTATTTTATATCAACACTTA

Parnassius_nordmanni_AC20_5 ------------------------
TGAGCAGGTATAGTAGGAACTTCTTTAAGATTATTAATTCGTACTGAATTAGGTAATCCTGGATCTCTAATTGGAGATGATCAAATTTACAATACTA
TCGTAACAGCTCATGCTTTTATTATAATTTTTTTCATAGTTATACCAATTATAATTGGAGGATTTGGAAATTGATTAATTCCATTAATATTAGGAGC
TCCAGATATAGCTTTCCCCCGAATAAATAATATAAGATTTTGATTATTACCCCCCTCATTAACTCTATTAATTTCTAGAAGAATTGTAGAAAATGGG
GCAGGAACTGGATGAACAGTTTACCCCCCTTTATCATCTAATATTGCTCATAGAGGAAGATCAGTTGACTTAGCTATTTTTTCTTTACATTTGGCTG
GGATTTCTTCTATTTTAGGAGCTATTAATTTTATCACAACTATTGTTAATATACGAATTAACTATATATCATTTGATCAAATACCTCTTTTCGTATG
AGCAGTAGGAATTACTGCTTTACTTTTATTATTATCTTTACCTGTTTTAGCAGGTGCTATTACTATATTATTAACAGATCGAAATCTTAATACTTCA
TTTTTTGATCCAGCAGGAGGTGGAGACCCTATTCTATATCAACATTTA

Parnassius_eversmanni_felderi_AC23_68 ------------------------
TGAGCAGGTATAGTAGGAACCTCTTTAAGATTATTAATTCGTTCTGAATTAGGTAATCCTGGATCTTTAATTGGAGATGATCAAATTTATAATACTA
TTGTAACAGCTCATGCTTTTATTATAATTTTTTTTATAGTTATACCAATTATAATTGGAGGATTTGGAAATTGATTAATTCCATTAATATTAGGAGC
TCCAGATATAGCTTTTCCTCGAATAAATAATATAAGATTTTGATTATTACCCCCCTCATTAACCTTACTAATTTCTAGAAGAATTGTAGAAAATGGA
GCAGGAACTGGATGAACGGTTTATCCCCCTTTATCATCTAATATTGCTCATAGAGGAAGATCAGTTGATTTAGCTATCTTTTCTTTACATTTAGCTG
GAATTTCATCTATCTTAGGAGCTATTAATTTTATTACAACTATTATTAATATACGAATTAATCATATATCATTTGATCAAATACCCCTTTTTGTATG
AGCAGTAGGAATTACTGCTTTACTTTTATTATTATCTTTACCTGTTTTAGCAGGTGCTATTACCATATTATTAACAGATCGAAATCTTAATACTTCA
TTTTTTGACCCAGCAGGAGGTGGAGATCCTATTTTATTTCAACATTTA

Parnassius_eversmanni_eversmanni_AC1_14 ------------------------
TGATCAGGTATAGTAGGAACCTCTTTAAGATTATTAATTCGTTCTGAATTAGGTAATCCTGGATCTTTAATTGGAGATGATCAAATTTATAATACTA
TTGTAACAGCTCATGCTTTTATTATAATTTTTTTTATAGTTATACCAATTATAATTGGAGGATTTGGAAATTGATTAATTCCATTAATATTAGGAGC
TCCAGATATAGCTTTTCCTCGAATAAATAATATAAGATTTTGATTATTACCCCCCTCATTAACCTTACTAATTTCTAGAAGAATTGTAGAAAATGGA
GCAGGAACTGGATGAACGGTTTATCCCCCTTTATCATCTAATATTGCTCATAGAGGAAGATCAGTTGATTTAGCTATCTTTTCTTTACATTTAGCTG
GAATTTCATCTATCTTAGGAGCTATTAATTTTATTACAACTATTATTAATATACGAATTAATCATATATCATTTGATCAAATACCCCTTTTTGTATG
AGCAGTAGGAATTACTGCTTTACTTTTATTATTATCTTTACCTGTTTTAGCAGGTGCTATTACCATATTATTAACAGATCGAAATCTCAATACTTCA
TTTTTTGACCCAGCAGGAGGTGGAGATCCTATTTTATATCAACATTTA

Parnassius_clodius_AC4_5 ------------------------
TGAGCAGGAATATTAGGAACCTCTTTAAGATTATTAATTCGTACTGAATTAGGTAATCCTGGATCTTTAATTGGAGATGATCAAATTTATAATACTA
TTGTAACAGCTCATGCTTTTATCATAATTTTTTTCATAGTTATACCAATTATAATTGGAGGATTTGGAAATTGATTAATTCCATTAATATTAGGAGC
TCCAGATATAGCTTTTCCTCGAATAAATAATATAAGATTTTGATTATTACCCCCTTCATTAACTTTATTAATTTCTAGAAGAATTGTAGAAAATGGA
GCAGGAACTGGATGAACAGTTTATCCCCCTTTATCATCTAATATTGCTCATAGAGGAAGATCAGTTGATTTAGCTATTTTTTCTTTACATTTAGCTG
GAATTTCATCTATCTTAGGAGCTATTAATTTTATTACAACTATTATTAATATACGAATTAATCATATATCATTTGATCAAATACCCCTTTTTGTATG

AGCAGTAGGAATTACCGCTTTACTTCTATTATTATCTTTACCTGTTTTAGCAGGTGCTATTACCATATTATTAACAGATCGAAATCTTAATACTTCA
TTTTTTGATCCAGCAGGAGGTGGAGATCCTATTTTATATCAACACTTA

Parnassius_ariadne_AC4_14 ------------------------
TGAGCAGGTATAGTAGGAACCTCTTTAAGATTATTAATTCGTACTGAATTAGGTAATCCTGGATCTTTAATTGGAGATGATCAAATTTATAATACTA
TCGTAACAGCTCATGCTTTTATTATAATTTTTTTTATAGTTATACCAATTATAATTGGAGGATTTGGAAATTGATTAATTCCTTTAATATTAGGAGC
TCCAGATATAGCCTTTCCTCGAATAAATAATATAAGATTTTGATTACTACCCCCCTCATTAACTTTATTAATCTCTAGAAGAATTGTAGAAAATGGA
GCAGGAACTGGATGAACAGTTTATCCCCCTTTATCATCTAATATTGCTCATAGAGGAAGTTCAGTTGATTTAGCCATTTTCTCTTTACATTTAGCAG
GAATTTCATCAATTTTAGGAGCTATTAATTTTATCACAACTATTATTAATATACGAATTAATCATATATCATTTGATCAAATACCCCTTTTTGTTTG
AGCAGTAGGAATTACTGCTTTACTATTATTATTATCTTTACCTGTATTAGCTGGTGCTATTACTATATCATTAACAGATCGAAATCTTAATACTTCA
TTTTTTGATCCAGCAGGAGGTGGAGATCCTATTTTATATCAACACTTA

HQ004911_RV_07_C107_Romania
ACACTATATTTTATTTTTGGTATTTGAGCAGGTATAGTAGGAACCTCTTTAAGATTATTAATTCGTACTGAATTAGGTAATCCTGGATCTTTAATTG
GAGATGATCAAATTTATAATACTATTGTAACAGCTCATGCTTTTATTATAATTTTTTTTATAGTTATACCAATTATAATTGGAGGATTTGGAAATTG
ATTAATTCCTTTAATATTAGGAGCCCCAGATATAGCTTTTCCTCGAATAAATAATATAAGATTTTGATTACTACCCCCCTCATTAACTTTATTAATT
TCTAGAAGAATTGTAGAAAATGGAGCAGGAACAGGATGAACAGTTTATCCCCCTTTATCATCTAATATCGCTCATAGAGGTAGTTCAGTTGATTTAG
CTATTTTTTCTTTACATTTAGCAGGAATTTCATCAATCTTAGGAGCTATTAATTTTATTACAACTATTATTAATATACGAATTAACCATATATCATT
TGATCAAATACCTCTCTTTGTATGAGCAGTAGGAATTACTGCTTTACTTTTATTATTATCTTTACCTGTATTAGCTGGTGCTATTACTATATTATTA
ACAGATCGAAATCTTAATACTTCATTTTTTGATCCTGCAGGAGGTGGAGATCCTATTTTATATCAACATTTA

HQ004908_RV_07_D060_Romania
ACACTATATTTTATTTTTGGTATTTGAGCAGGTATAGTAGGAACCTCTTTAAGATTATTAATTCGTACTGAATTAGGTAATCCTGGATCTTTAATTG
GAGATGATCAAATTTATAATACTATTGTAACAGCTCATGCTTTTATTATAATTTTTTTTATAGTTATACCAATTATAATTGGAGGATTTGGAAATTG
ATTAATTCCTTTAATATTAGGAGCCCCAGATATAGCTTTTCCTCGAATAAATAATATAAGATTTTGATTACTACCCCCCTCATTAACTTTATTAATT
TCTAGAAGAATTGTAGAAAATGGAGCAGGAACAGGATGAACAGTTTATCCCCCTTTATCATCTAATATCGCTCATAGAGGTAGTTCAGTTGATTTAG
CTATTTTTTCTTTACATTTAGCAGGAATTTCATCAATCTTAGGAGCTATTAATTTTATTACAACTATTATTAATATACGAATTAACCATATATCATT
TGATCAAATACCTCTCTTTGTATGAGCAGTAGGAATTACTGCTTTACTTTTATTATTATCTCTACCTGTATTAGCTGGTGCTATTACTATATTATTA
ACAGATCGAAATCTTAATACTTCATTTTTTGATCCTGCAGGAGGTGGAGATCCTATTTTATATCAACATTTA

HQ004907_RV_08_M274_Romania
ACACTATATTTTATTTTTGGTATTTGAGCAGGTATAGTAGGAACCTCTTTAAGATTATTAATTCGTACTGAATTAGGTAATCCTGGATCTTTAATTG
GAGATGATCAAATTTATAATACTATTGTAACAGCTCATGCTTTTATTATAATTTTTTTTATAGTTATACCAATTATAATTGGAGGATTTGGAAATTG
ATTAATTCCTTTAATATTAGGAGCCCCAGATATAGCTTTTCCTCGAATAAATAATATAAGATTTTGATTACTACCCCCCTCATTAACTTTATTAATT
TCTAGAAGAATTGTAGAAAATGGAGCAGGAACAGGATGAACAGTTTATCCCCCTTTATCATCTAATATCGCTCATAGAGGTAGTTCAGTTGATTTAG
CTATTTTTTCTTTACATTTAGCAGGAATTTCATCAATCTTAGGAGCTATTAATTTTATTACAACTATTATTAATATACGAATTAACCATATATCATT
TGATCAAATACCTCTATTTGTATGAGCAGTAGGAATTACTGCTTTACTTTTATTATTATCTTTACCTGTATTAGCTGGTGCTATTACTATATTATTA
ACAGATCGAAATCTTAATACTTCATTTTTTGATCCTGCAGGAGGTGGAGATCCTATTTTATATCAACATTTA

HQ004902_RV_08_M361_Romania
ACACTATATTTTATTTTTGGTATTTGAGCAGGTATAGTAGGAACCTCTTTAAGATTATTAATTCGTACTGAATTAGGTAATCCTGGATCTTTAATTG
GAGATGATCAAATTTATAATACTATTGTAACAGCTCATGCTTTTATTATAATTTTTTTTATAGTTATACCAATTATAATTGGAGGATTTGGAAATTG
ATTAATTCCCTTAATATTAGGAGCCCCAGATATAGCTTTTCCTCGAATAAATAATATAAGATTTTGATTACTACCCCCCTCATTAACTTTATTAATT
TCTAGAAGAATTGTAGAAAATGGAGCAGGAACAGGATGAACAGTTTATCCCCCTTTATCATCTAATATCGCTCATAGAGGTAGTTCAGTTGATTTAG
CTATTTTTTCTTTACATTTAGCAGGAATTTCATCAATCTTAGGAGCTATTAATTTTATTACAACTATTATTAATATACGAATTAACCATATATCATT
TGATCAAATACCTCTCTTTGTATGAGCAGTAGGAATTACTGCTTTACTTTTATTATTATCTTTACCTGTATTAGCTGGTGCTATTACTATATTATTA
ACAGATCGAAATCTTAATACTTCATTTTTTGATCCTGCAGGAGGTGGAGATCCTATTTTATATCAACATTTA

GU947642_Pmne_ITNEB01_Italy_Sicily --

CCCCTTCATTAACCTTATTAATTTCTAGAAGAATTGTAGAAAATGGAGCAGGAACTGGATGAACAGTTTACCCCCCTTTATCATCTAATATTGCTCA
TAGAGGAAGTTCAGTTGATTTAGCTATTTTTTCCTTACATTTAGCGGGAATTTCATCAATCTTAGGAGCTATTAATTTTATTACAACTATTATTAAT
ATACGAATTAATCATATATCATTCGATCAAATACCTCTTTTTGTATGAGCAGTAGGAATTACTGCTTTACTTTTATTATTATCTTTACCTGTATTAG
CTGGTGCTATTACTATATTATTAACAGATCGAAATCTTAATACTTCATTTTTTGATCCCGCAGGAGGTGGTGATCCTATTTTATACCAACATTTA

EU093018_Ca995 --

TTTAATATTAGGAGCCCCAGATATAGCTTTTCCTCGAATAAATAATATAAGATTTTGATTACTGCCCCCCTCATTAACTTTATTAATTTCTAGAAGA
ATTGTAGAAAATGGAGCAGGAACAGGATGAACAGTTTATCCCCCTTTATCATCTAATATCGCTCATAGAGGTAGTTCAGTTGATTTAGCTATTTTTT
CTTTACATTTAGCAGGAATTTCATCAATCTTAGGAGCTATTAATTTTATTACAACTATTATTAATATACGAATTAACCATATATCATTTGATCAAAT
ACCTCTCTTTGTATGAGCAGTAGGAATTACTGCTTTACTTTTATTATTATCTTTACCTGTATTAGCTGGTGCTATTACTATATTATTAACAGATCGA
AATCTTAATACTTCATTTTTTGATCCTGCAGGAGGTGGAGATCCTATTTTATATCAACATTTA

EU093017_Ch892 --

TTTAATATTAGGAGCCCCAGATATAGCTTTCCCCCGAATAAATAATATAAGATTTTGATTACTACCCCCTTCATTAACCTTATTAATTTCTAGAAGA
ATTGTAGAAAATGGAGCAGGAACTGGATGAACAGTTTACCCCCCTTTATCATCTAATATTGCTCATAGAGGAAGTTCAGTTGATTTAGCTATTTTTT
CCCTACATTTAGCGGGAATTTCATCAATCTTAGGAGCTATTAATTTTATTACAACTATTATCAATATACGAATTAATCATATATCATTCGATCAAAT
ACCTCTTTTTGTATGAGCAGTAGGAATTACTGCTTTACTTTTATTATTATCCTTACCTGTATTAGCTGGTGCTATTACTATATTATTAACAGATCGA
AATCTTAATACTTCATTTTTTGATCCAGCAGGAGGTGGTGATCCTATTTTATATCAACATTTA

EU093016_Tk889 --

TTTAATATTAGGAGCCCCAGATATAGCTTTTCCTCGAATAAATAATATAAGATTTTGATTACTACCCCCCTCATTAACTTTATTAATTTCTAGAAGA
ATTGTAGAAAATGGAGCAGGAACAGGATGAACAGTTTATCCCCCTTTATCATCTAATATTGCTCATAGAATTAGTTCAGTTGATTTAGCTATTTTTT

CTTTACATTTAGCAGGAATTTCATCAATCTTAGGAGCTATTAATTTTATTACAACTATTATTAATATACGAATTAATCATTTATCATTTGATCAAAT
ACCCCTCTTTGTATGAGCAGTAGGAATTACTGCTTTACTTTTATTATTATCTTTACCTGTATTAGCTGGTGCTATTACTATATTATTAACAGATCGA
AATCTTAATACTTCATTTTTTGATCCTGCAGGAGGTGGAGATCCTATTTTATATCAACATTTA

EU093015_It878 --

TTTAATATTAGGAGCCCCAGATATAGCTTTCCCCCGAATAAATAATATAAGATTTTGATTACTACCCCCTTCATTAACCTTATTAATTTCTAGAAGA
ATTGTAGAAAATGGAGCAGGAACTGGATGAACAGTTTACCCCCCTTTATCATCTAATATTGCTCATAGAGGAAGTTCAGTTGATTTAGCTATTTTTT
CCTTACATTTAGCGGGAATTTCATCAATCTTAGGAGCTATTAATTTTATTACAACTATTATTAATATACGAATTAATCATATATCATTCGATCAAAT
ACCTCTTTTGTATGAGCAGTAGGAATTACTGCTTTACTTTTATTATTATCTTTACCTGTATTAGCTGGTGCTATTACTATATTATTAACAGATCGA
AATCTTAATACTTCATTTTTTGATCCAGCAGGAGGTGGTGATCCTATTTTATACCAACATTTA

EU093014_Hu875 --

TTTAATATTAGGAGCCCCAGATATAGCTTTTCCTCGAATAAATAATATAAGATTTTGATTACTACCCCCCTCATTAACTTTATTAATTTCTAGAAGA
ATTGTAGAAAATGGAGCAGGAACAGGATGAACAGTTTATCCCCCTTTATCATCTAATATCGCTCATAGAGGTAGTTCAGTTGATTTAGCTATTTTTT
CTTTACATTTAGCGGGAATTTCATCAATCTTAGGAGCTATTAATTTTATTACAACTATTATTAATATACGAATTAACCATATATCATTTGATCAAAT
ACCTCTCTTTGTATGAGCAGTAGGAATTACTGCTTTACTTTTATTATTATCTTTACCTGTATTAGCTGGTGCTATTACTATATTATTAACAGATCGA
AATCTTAATACTTCATTTTTTGATCCTGCAGGAGGTGGAGATCCTATTTTATATCAACATTTA

EU093013_Sk872_Cz_Slova --

TTTAATATTAGGAGCCCCAGATATAGCTTTTCCTCGAATAAATAATATAAGATTTTGATTACTACCCCCCTCATTAACTTTATTAATTTCTAGAAGA
ATTGTAGAAAATGGAGCAGGAACAGGATGAACAGTTTATCCCCCTTTATCATCTAATATCGCTCATAGAGGTAGTTCAGTTGATTTAGCTATTTTTT
CTTTACATTTGGCAGGAATTTCATCAATCTTAGGAGCTATTAATTTTATTACAACTATTATTAATATACGAATTAATCATATATCATTTGATCAAAT
ACCTCTCTTTGTATGAGCAGTAGGAATTACTGCTTTACTTTTATTATTATCTTTACCTGTATTAGCTGGTGCTATTACTATATTATTAACAGATCGA
AATCTTAATACTTCATTTTTTGATCCTGCAGGAGGTGGAGATCCTATTTTATATCAACATTTA

EU093011_Cz869 --

TTTAATATTAGGAGCCCCAGATATAGCTTTTCCTCGAATAAATAATATAAGATTTTGATTACTACCCCCCTCATTAACTTTATTAATTTCTAGAAGA
ATTGTAGAAAATGGAGCAGGAACAGGATGAACAGTTTATCCCCCTTTATCATCTAATATTGCTCATAGAGGTAGTTCAGTTGATTTAGCTATTTTTT
CTTTACATTTAGCAGGAATTTCATCAATCTTAGGAGCTATTAATTTTATTACAACTATTATTAATATACGAATTAACCATATATCATTTGATCAAAT
ACCTCTCTTTGTATGAGCAGTAGGAATTACTGCTTTACTTTTATTATTATCTTTACCTGTATTAGCTGGTGTTATTACTATATTATTAACAGATCGA
AATCTTAATACTTCATTTTTTGATCCTGCAGGAGGTGGAGATCCTATTTTATATCAACATTTA

EU093010_Ru_Bosnia_Fi_Bu_Hu_Bel_Po_Rom_Ukr_ --

TTTAATATTAGGAGCCCCAGATATAGCTTTTCCTCGAATAAATAATATAAGATTTTGATTACTACCCCCCTCATTAACTTTATTAATTTCTAGAAGA
ATTGTAGAAAATGGAGCAGGAACAGGATGAACAGTTTATCCCCCTTTATCATCTAATATCGCTCATAGAGGTAGTTCAGTTGATTTAGCTATTTTTT
CTTTACATTTAGCAGGAATTTCATCAATCTTAGGAGCTATTAATTTTATTACAACTATTATTAATATACGAATTAACCATATATCATTTGATCAAAT
ACCTCTCTTTGTATGAGCAGTAGGAATTACTGCTTTACTTTTATTATTATCTTTACCTGTATTAGCTGGTGCTATTACTATATTATTAACAGATCGA
AATCTTAATACTTCATTTTTTGATCCTGCAGGAGGTGGAGATCCTATTTTATATCAACATTTA

EU093009_Kz848 --

TTTAATATTAGGGGCCCCAGATATAGCTTTTCCTCGAATAAATAATATAAGATTTTGATTATTACCCCCTTCATTAACTTTATTAATTTCTAGAAGA
ATTGTAGAAAATGGAGCAGGAACAGGATGAACAGTTTATCCCCCTTTATCATCTAATATCGCTCATAGAGGTAGTTCAGTTGATTTAGCTATTTTTT
CTTTACATTTAGCAGGAATTTCATCAATCTTAGGAGCTATTAATTTTATTACAACTATTATTAATATACGAATTAATCATATATCATTTGATCAAAT
ACCTCTCTTTGTATGAGCAGTAGGAATTACTGCTTTACTTTTATTACTATCTTTACCTGTATTAGCTGGTGCTATTACTATATTATTAACAGATCGA
AATCTTAATACTTCATTTTTTGATCCTGCAGGAGGTGGAGATCCTATTTTATATCAACATTTA

EU093007_Fi810 --

TTTAATATTAGGAGCCCCAGATATAGCCTTTCCTCGAATAAATAATATAAGATTTTGATTACTACCCCCCTCATTAACTTTATTAATTTCTAGAAGA
ATTGTAGAAAATGGAGCAGGAACAGGATGAACAGTTTATCCCCCTTTATCATCTAATATCGCTCATAGAGGTAGTTCAGTTGATTTAGCTATTTTTT
CTTTACATTTAGCAGGAATTTCATCAATCTTAGGAGCTATTAATTTTATTACAACTATTATTAATATACGAATTAACCATATATCATTTGATCAAAT
ACCTCTCTTTGTATGAGCAGTAGGAATTACTGCTTTACTTTTATTATTATCTTTACCTGTATTAGCTGGTGCTATTACTATATTATTAACAGATCGA
AATCTTAATACTTCATTTTTTGATCCTGCAGGAGGTGGAGATCCTATTTTATATCAACATTTA

EU093004_Tu999 --

TTTAATATTAGGAGCTCCAGATATAGCTTTTCCTCGAATAAATAACATAAGATTTTGATTACTCCCCCCTTCATTAACTTTATTAATTTCTAGAAGA
ATTGTAGAAAATGGAGCAGGAACAGGATGAACAGTTTATCCCCCTTTATCATCTAATATTGCTCATAGAGGTAGTTCAGTTGATTTAGCTATTTTTT
CTTTACATTTAGCAGGAATTTCATCAATCTTAGGAGCTATTAATTTTATTACAACTATTATTAATATACGAATTAATAATATATCATTTGATCAAAT
ACCTCTTTTGTATGAGCAGTAGGAATTACTGCATTACTTTTATTATTATCTTTACCTGTATTAGCTGGTGCTATTACTATATTATTAACAGATCGA
AATCTTAATACTTCATTTTTTGATCCCGCAGGAGGTGGAGATCCTATTTTATATCAACACTTA

EU093000_Bu961 --

TTTAATATTAGGAGCCCCAGATATAGCTTTTCCTCGAATAAATAATATAAGATTTTGATTACTACCCCCCTCATTAACTTTATTAATTTCTAGAAGA

ATTGTAGAAAATGGAGCAGGAACAGGATGAACAGTTTATCCCCCTTTATCATCTAATATCGCTCATAGAGGTAGTTCAGTTGATTTAGCTATTTTTT
CTTTACATTTAGCAGGAATTTCATCAATCTTAGGAGCTATTAATTTTATTACAACTATTATTAATATACGAATTAATCATATATCATTTGATCAAAT
ACCTCTCTTTGTATGAGCAGTAGGAATTACTGCTTTACTTTTATTATTATCTTTACCTGTATTAGCTGGTGCTATTACTATATTATTAACAGATCGA
AATCTTAATACTTCATTTTTTGATCCTGCAGGAGGTGGAGATCCTATTTTATATCAACATTTA

EU092998_Bu951 --

TTTAATATTAGGAGCCCCAGATATAGCTTTTCCTCGAATAAATAATATAAGATTTTGATTACTACCCCCCTCATTAACTTTATTAATTTCTAGAAGA
ATTGTAGAAAATGGAGCAGGAACAGGATGAACAGTTTATCCCCCTTTATCATCTAATATCGCTCATAGTGGTAGTTCAGTTGATTTAGCTATTTTTT
CTTTACATTTAGCAGGAATTTCATCAATCTTAGGAGCTATTAATTTTATTACAACTATTATTAATATACGAATTAACCATATATCATTTGATCAAAT
ACCTCTCTTTGTATGAGCAGTAGGAATTACTGCTTTACTTTTATTATTATCTTTACCTGTATTAGCTGGTGCTATTACTATATTATTAACAGATCGA
AATCTTAATACTTCATTTTTTGATCCTGCAGGAGGTGGAGATCCTATTTTATATCAACATTTA

EU092997_Ru940 --

TTTAATATTAGGAGCCCCAGATATAGCTTTTCCTCGAATAAATAATATAAGATTTTGATTACTACCCCCCTCATTAACTTTATTAATTTCTAGAAGA
ATTGTAGAAAATGGAGCAGGAACAGGATGAACAGTTTATCCCCCTTTATCATCTAATATCGCTCATAGAGGTAGTTCAGTTGATTTAGCTATTTTTT
CTTTACATTTAGCAGGAATTTCATCAATCTTAGGAGCTATTAATTTTATTACAACTATTATTAATATACGAATTAACCATATATCATTTGATCAAAT
ACCTCTCTTTGTATGAGCAGTAGGAATTACTGCTTTACTTTTATTATTATCTCTACCTGTATTAGCTGGTGCTATTACTATATTATTAACAGATCGA
AATCTTAATACTTCATTTTTTGATCCTGCAGGAGGTGGGGATCCTATTTTATATCAACATTTA

EU092996_Sp924_Fr --

TTTAATATTAGGAGCCCCAGATATAGCTTTCCCCCGAATAAATAATATAAGATTTTGATTACTACCCCCTTCATTAACCTTATTAATTTCTAGAAGA
ATTGTAGAAAATGGAGCAGGAACTGGATGAACAGTTTACCCCCCTTTATCATCCAATATTGCTCATAGAGGAAGTTCAGTTGATTTAGCTATTTTTT
CCCTACATTTAGCAGGAATTTCATCAATCTTAGGAGCTATTAATTTTATTACAACTATTATTAATATACGAATTAATCATATATCATTCGATCAAAT
ACCTCTTTTTGTGTGAGCAGTAGGAATTACTGCTTTACTTTTATTATTATCCTTACCTGTATTAGCTGGTGCTATTACTATATTATTAACAGATCGA
AATCTTAATACTTCATTTTTTGATCCAGCAGGAGGTGGTGATCCTATTTTATATCAACATTTA

EU092995_Sp923 --

TTTAATATTAGGAGCCCCAGATATAGCTTTCCCCCGAATAAATAATATAAGATTTTGATTACTACCCCCTTCATTAACCTTATTAATTTCTAGAAGA
ATTGTAGAAAATGGAGCAGGAACTGGATGAACAGTTTATCCCCCTTTATCATCCAATATTGCTCATAGAGGAAGTTCAGTTGATTTAGCTATTTTTT
CCCTACATTTAGCGGGAATTTCATCAATCTTAGGAGCTATTAATTTTATTACAACTATTATTAATATACGAATTAATCATATATCATTCGATCAAAT
ACCTCTTTTTGTGTGAGCAGTAGGAATTACTGCTTTACTTTTATTATTATCCTTACCTGTATTAGCTGGTGCTATTACTATATTATTAACGGATCGA
AATCTTAATACTTCATTTTTTGATCCAGCAGGAGGTGGTGATCCTATTTTATATCAACATTTA

EU092994_Sp921 --

TTTAATATTAGGAGCCCCAGATATAGCTTTCCCCCGAATAAATAATATAAGATTTTGATTACTACCCCCTTCATTAACCTTATTAATTTCTAGAAGA
ATTGTAGAAAATGGAGCAGGAACTGGATGAACAGTTTACCCCCCTTTATCATCCAATATTGCTCATAGAGGAAGTTCAGTTGATTTAGCTATTTTTT
CCCTACATTTAGCAGGAATTTCATCAATCTTAGGAGCTATTAATTTTATTACAACTATTATTAATATACGAATTAATCATATATCATTCGATCAAAT
ACCTCTTTTTGTGTGAGCAGTAGGAATTACTGCTTTACTTTTATTATTATCCTTACCTGTATTAGCTGGTGCTATTACTATATTATTAACGGATCGA
AATCTTAATACTTCATTTTTTGATCCAGCAGGAGGTGGTGATCCTATTTTATATCAACATTTA

EU092993_Sp917 --

TTTAATATTAGGAGCCCCAGATATAGCTTTCCCCCGAATAAATAATATAAGATTTTGATTACTACCCCCTTCATTAACCTTATTAATTTCTAGAAGA
ATTGTAGAAAATGGAGCAGGAACTGGATGAACAGTTTATCCCCCTTTATCATCCAATATTGCTCATAGAGGAAGTTCAGTTGATTTAGCTATTTTTT
CCCTACATTTAGCAGGAATTTCATCAATCTTAGGAGCTATTAATTTTATTACAACTATTATTAATATACGAATTAATCATATATCATTCGATCAAAT
ACCTCTTTTTGTGTGAGCAGTAGGAATTACTGCTTTACTTTTATTATTATCCTTACCTGTATTAGCTGGTGCTATTACTATATTATTAACAGATCGA
AATCTTAATACTTCATTTTTTGATCCAGCAGGAGGTGGTGATCCTATTTTATATCAACATTTA

EU092992_Sp916 --

TTTAATATTAGGAGCCCCAGATATAGCTTTCCCCCGAATAAATAATATAAGATTTTGATTACTACCCCCTTCATTAACCTTATTAATTTCTAGAAGA
ATTGTAGAAAATGGAGCAGGAACTGGATGAACAGTTTACCCCCCTTTATCATCCAATATTGCTCATAGAGGAAGTTCAGTTGATTTAGCTATTTTTT
CCCTACATTTAGCAGGAATTTCATCAATCTTAGGAGCTATTAATTTTATTACAACTATTATTAATATACGAATTAATCATATATCATTCGATCAAAT
ACCTCTTTTTGTGTGAGCAGTAGGAATTACTGCTTTACTTTTATTATTATCCTTACCTGTATTAGCTGGTGCTATTACTATATTATTAACAGATCGA
AATATTAATACTTCATTTTTTGATCCAGCAGGAGGTGGTGATCCTATTTTATATCAACATTTA

EU092991_Fr913 --

TTTAATATTAGGAGCCCCAGATATAGCTTTCCCCCGAATAAATAATATAAGATTTTGATTACTACCCCCTTCATTAACCTTATTAATTTCTAGAAGA
ATTGTAGAAAATGGAGCAGGAACTGGATGAACAATTTACCCCCCTTTATCATCCAATATTGCTCATAGAGGAAGTTCAGTTGATTTAGCTATTTTTT
CCCTACATTTAGCAGGAATTTCATCAATCTTAGGAGCTATTAATTTTATTACAACTATTATTAATATACGAATTAATCATATATCATTCGATCAAAT
ACCTCTTTTTGTGTGAGCAGTAGGAATTACTGCTTTACTTTTATTATTATCCTTACCTGTATTAGCTGGTGCTATTACTATATTATTAACAGATCGA
AATCTTAATACTTCATTTTTTGATCCAGCAGGAGGTGGTGATCCTATTTTATATCAACATTTA

EU092990_Fr911 --

TTTAATATTAGGAGCCCCAGATATAGCTTTCCCCCGAATAAATAATATAAGATTTTGATTACTACCCCCTTCATTAACCTTATTAATTTCTAGAAGA
ATTGTAGAAAATGGAGCAGGAACTGGATGAACAGTTTACCCCCCTTTATCATCTAATATTGCTCATAGAGGAAGTTCAGTTGATTTAGCTATTTTTT
CCTTACATTTAGCAGGAATTTCATCAATCTTAGGAGCTATTAATTTTATTACAACTATTATTAATATACGAATTAATCATATATCATTCGATCAAAT
ACCTCTTTTTGTATGAGCAGTAGGAATTACTGCTTTACTTTTATTATTATCATTACCTGTATTAGCTGGTGCTATTACTATATTATTAACAGATCGA
AATCTTAATACTTCATTTTTTGATCCAGCAGGGGGTGGTGATCCTATTTTATATCAACATTA

EU092988_It639 --

TTTAATATTAGGAGCCCCAGATATAGCTTTCCCCCGAATAAATAATATAAGATTTTGATTACTACCCCCTTCATTAACCTTATTAATTTCTAGAAGA
ATTGTAGAAAATGGAGCAGGAACTGGATGAACAGTTTACCCCCCTTTATCATCTAATATTGCTCATAGAGGAAGTTCAGTTGATTTAGCTATTTTTT
CCCTACATTTAGCGGGAATTTCATCAATCTTAGGAGCTATTAATTTTATTACAACTATTATTAATATACGAATTAATCATATATCATTTGATCAAAT
ACCTCTTTTTGTATGAGCAGTAGGAATTACTGCTTTACTTTTATTATTATCCTTACCTGTATTAGCTGGTGCTATTACTATATTATTAACAGATCGA
AATCTTAATACTTCATTTTTTGATCCAGCAGGAGGTGGTGATCCTATTTTATATCAACATTA

EU092987_Fr528 --

TTTAATATTAGGAGCCCCAGATATAGCTTTCCCCCGAATAAATAATATAAGATTTTGATTACTACCCCCTTCATTAACCTTATTAATTTCTAGAAGA
ATTGTAGAAAATGGAGCAGGAACTGGATGAACAGTTTACCCCCCTTTATCATCTAATATTGCTCATAGAGGAAGTTCAGTTGATTTAGCTATTTTTT
CCCTACATTTAGCGGGAATTTCATCAATCTTAGGAGCTATTAATTTTATTACAACTATTATTAATATACGAATTAATCATATATCATTCGATCAAAT
ACCTCTTTTTGTATGAGCAGTAGGAATTACTGCTTTACTTTTATTATTATCCTTACCTGTATTAGCTGGTGCTATTACTATATTATTAACAGATCGA
AATCTTAATACTTCATTTTTTGATCCAGCAGGAGGTGGTGATCCTATTTTATATCAACATTA

EU092985_Fr525 --

TTTAATATTAGGAGCCCCAGATATAGCTTTCCCCCGAATAAATAATATAAGATTTTGATTACTACCCCCTTCATTAACCTTATTAATTTCTAGAAGA
ATTGTAGAAAATGGAGCAGGAACTGGATGAACAGTTTACCCCCCTTTATCATCTAATATTGCTCATAGAGGAAGTTCAGTTGATTTAGCTATTTTTT
CCTTACATTTAGCAGGAATTTCATCAATCTTAGGAGCTATTAATTTTATTACAACTATTATTAATATACGAATTAATCATATATCATTCGATCAAAT
ACCTCTTTTTGTATGAGCAGTAGGAATTACTGCTTTACTTTTATTATTATCATTACCTGTATTAGCTGGTGCTATTACTATATTATTAACAGATCGA
AATCTTAATACTTCATTTTTTGATCCAGCAGGAGGTGGTGATCCTATTTTATATCAACATTA

EU092984_Fr522 --

TTTAATATTAGGAGCCCCAGATATAGCTTTCCCCCGAATAAATAATATAAGATTTTGATTACTACCCCCTTCATTAACCTTATTAATTTCTAGAAGA
ATTGTAGAAAATGGAGCAGGAACTGGATGAACAGTTTACCCCCCTTTATCATCTAATATTGCTCATAGAGGAAGTTCAGTTGATTTAGCTATTTTTT
CCTTACATTTAGCAGGAATTTCATCAATCTTAGGAGCTATTAATTTTATTACAACTATTATTAATATACGAATTAATCATATATCATTCGATCAAAT
ACCTCTTTTTGTATGAGCAGTAGGAATTACTGCTTTACTCTTATTATTATCATTACCTGTATTAGCTGGTGCTATTACTATATTATTAACAGATCGA
AATCTTAATACTTCATTTTTTGATCCAGCAGGGGGTGGTGATCCTATTTTATATCAACATTA

EU092983_Ch765 --

TTTAATATTAGGAGCCCCAGATATAGCTTTCCCCCGAATAAATAATATAAGATTTTGATTACTACCCCCTTCATTAACCTTATTAATTTCTAGAAGA
ATTGTAGAAAATGGAGCAGGAACTGGATGAACAGTTTACCCCCCTTTATCATCTAATATTGCTCATAGAGGAAGTTCAGTTGATTTAGCTATTTTTT
CCCTACATTTAGCAGGAATTTCATCAATCTTAGGAGCTATTAATTTTATTACAACTATTATTAATATACGAATTAATCATATATCATTCGATCAAAT
ACCTCTTTTTGTATGAGCAGTAGGAATTACTGCTTTACTTTTATTATTATCCTTACCTGTATTAGCTGGTGCTATTACTATATTATTAACAGATCGA
AATCTTAATACTTCATTTTTTGATCCAGCAGGAGGTGGTGATCCTATTTTATATCAACATTA

EU092982_Pl653_Slova_Ukr --

TTTAATATTAGGAGCCCCAGATATAGCTTTTCCTCGAATAAATAATATAAGATTTTGATTACTACCCCCCTCATTAACTTTATTAATTTCTAGAAGA
ATTGTAGAAAATGGAGCAGGAACAGGATGAACAATTTATCCCCCTTTATCATCTAATATCGCTCATAGAGGTAGTTCAGTTGATTTAGCTATTTTTT
CTTTACATTTAGCAGGAATTTCATCAATCTTAGGAGCTATTAATTTTATTACAACTATTATTAATATACGAATTAACCATATATCATTTGATCAAAT
ACCTCTCTTTGTATGAGCAGTAGGAATTACTGCTTTACTTTTATTATTATCTTTACCTGTATTAGCTGGTGCTATTACTATATTATTAACAGATCGA
AATCTTAATACTTCATTTTTTGATCCTGCAGGAGGTGGAGATCCTATTTTATATCAACATTA

EU092981_At626 --

TTTAATATTAGGAGCCCCAGATATAGCTTTTCCTCGAATAAATAATATAAGATTTTGATTACTACCCCCCTCATTAACTTTATTAATTTCTAGAAGA
ATTGTAGAAAATGGAGCAGGAACAGGATGAACAGTTTATCCCCCTTTATCATCTAATATCGCTCATAGAGGTAGTTCAGTTGATTTAGCTATTTTTT
CTTTACATTTAGCAGGAATTTCATCAATCTTAGGAGCTATTAATTTTATTACAACTATTATTAATATACGAATTAACCATATATCATTTGATCAAAT
ACCTCTCTTTGTATGAGCAGTAGGAATTACTGCTTTACTTTTATTATTATCTTTACCTGTGTTAGCTGGTGCTATTACTATATTATTAACAGATCGA
AATCTTAATACTTCATTTTTTGATCCTGCAGGAGGTGGAGATCCTATTTTATATCAACATTA

EU092980_At622 --

TTTAATATTAGGAGCCCCAGATATAGCTTTTCCTCGAATAAATAATATAAGATTTTGATTACTACCCCCCTCATTAACTTTATTAATTTCTAGAAGA
ATTGTAGAAAATGGAGCAGGAACAGGATGAACAGTTTATCCCCCTTTATCATCTAATATTGCTCATAGAGGTAGTTCAGTTGATTTAGCTATTTTTT
CTTTACATTTAGCAGGAATTTCATCAATCTTAGGAGCTATTAATTTTATTACAACTATTATTAATATACGAATTAACCATATATCATTTGATCAAAT
ACCTCTCTTTGTATGAGCAGTAGGAATTACTGCTTTACTTTTATTACTATCTTTACCTGTATTAGCTGGTGCTATTACTATATTATTAACAGATCGA
AATCTTAATACTTCATTTTTTGATCCTGCAGGAGGTGGAGATCCTATTTTATATCAACATTA

EU092978_At618_At_Slo --

TTTAATATTAGGAGCCCCAGATATAGCTTTTCCTCGAATAAATAATATAAGATTTTGATTACTACCCCCCTCATTAACTTTATTAATTTCTAGAAGA
ATTGTAGAAAATGGGGCAGGAACAGGATGAACAGTTTATCCCCCTTTATCATCTAATATCGCTCATAGAGGTAGTTCAGTTGATTTAGCTATTTTTT
CTTTACATTTAGCAGGAATTTCATCAATCTTAGGAGCTATTAATTTTATTACAACTATTATTAATATACGAATTAACCATATATCATTTGATCAAAT
ACCTCTCTTTGTATGAGCAGTAGGAATTACTGCTTTACTTTTATTATTATCTTTACCTGTATTAGCTGGTGCTATTACTATATTATTAACAGATCGA
AATCTTAATACTTCATTTTTTGATCCTGCAGGAGGTGGAGATCCTATTTTATATCAACATTTA

EU092977_At606_At_Cz_D_Fi_Hu_Po_Slova --

TTTAATATTAGGAGCCCCAGATATAGCTTTTCCTCGAATAAATAATATAAGATTTTGATTACTACCCCCCTCATTAACTTTATTAATTTCTAGAAGA
ATTGTAGAAAATGGAGCAGGAACAGGATGAACAGTTTATCCCCCTTTATCATCTAATATTGCTCATAGAGGTAGTTCAGTTGATTTAGCTATTTTTT
CTTTACATTTAGCAGGAATTTCATCAATCTTAGGAGCTATTAATTTTATTACAACTATTATTAATATACGAATTAACCATATATCATTTGATCAAAT
ACCTCTCTTTGTATGAGCAGTAGGAATTACTGCTTTACTTTTATTATTATCTTTACCTGTATTAGCTGGTGCTATTACTATATTATTAACAGATCGA
AATCTTAATACTTCATTTTTTGATCCTGCAGGAGGTGGAGATCCTATTTTATATCAACATTTA

EU092976_At602 --

TTTAATATTAGGAGCCCCAGATATAGCTTTTCCTCGAATAAATAATATAAGATTTTGATTACTACCCCCCTCATTAACTTTATTAATTTCTAGAAGA
ATTGTAGAAAATGGAGCAGGAACAGGATGAACAGTTTATCCCCCTTTATCATCTAATATTGCTCATAGAGGTAGTTCAGTTGATTTAGCTATTTTTT
CTTTACATTTAGTAGGAATTTCATCAATCTTAGGAGCTATTAATTTTATTACAACTATTATTAATATACGAATTAACCATATATCATTTGATCAAAT
ACCTCTCTTTGTATGAGCAGTAGGAATTACTGCTTTACTTTTATTATTATCTTTACCTGTATTAGCTGGTGCTATTACTATATTATTAACAGATCGA
AATCTTAATACTTCATTTTTTGATCCTGCAGGAGGTGGAGATCCTATTTTATATCAACATTTA

EU092975_D757 --

TTTAATATTAGGAGCCCCAGATATAGCTTTTCCTCGAATAAATAATATAAGATTTTGATTACTACCCCCCTCATTAACTTTATTAATTTCTAGAAGA
ATTGTAGAAAATGGAGCAGGAACAGGATGAACAGTTTATCTTCCTTTATCATCTAATATTGCTCATAGAGGTAGTTCAGTTGATTTAGCTATTTTTT
CTTTACATTTAGCAGGAATTTCATCAATCTTAGGAGCTATTAATTTTATTACAACTATTATTAATATACGAATTAACCATATATCATTTGATCAAAT
ACCTCTCTTTGTATGAGCAGTAGGAATTACTGCTTTACTTTTATTATTATCTTTACCTGTATTAGCTGGTGCTATTACTATATTATTAACAGATCGA
AATCTTAATACTTCATTTTTTGATCCTGCAGGAGGTGGAGATCCTATTTTATATCAACATTTA

EU092974_D756 --

TTTAATATTAGGAGCCCCAGATATAGCTTTTCCTCGAATAAATAATATAAGATTTTGATTACTACCCCCCTCATTAACTTTATTAATTTCTAGAAGA
ATTGTAGAAAATGGAGCAGGAACAGGATGAACAATTTATCCCCCTTTATCATCTAATATTGCTCATAGAGGTAGTTCAGTTGATTTAGCTATTTTTT
CTTTACATTTAGCAGGAATTTCATCAATCTTAGGAGCTATTAATTTTATTACAACTATTATTAATATACGAATTAACCATATATCATTTGATCAAAT
ACCTCTCTTTGTATGAGCAGTAGGAATTACTGCTTTACTTTTATTATTATCTTTACCTGTATTAGCTGGTGCTATTACTATATTATTAACAGATCGA
AATCTTAATACTTCATTTTTTGATCCTGCAGGAGGTGGAGATCCTATTTTATATCAACATTTA

EU092973_D744 --

TTTAATATTAGGAGCCCCAGATATAGCTTTTCCTCGAATAAATAATATAAGATTTTGATTACTACCCCCCTCATTAACTTTATTAATTTCTAGAAGA
ATTGTAGAAAATGGAGCAGGAACAGGATGAACGGTTTATCCCCCTTTATCATCTAATATTGCTCATAGAGGTAGTTCAGTTGATTTAGCTATTTTTT
CTTTACATTTAGCAGGAATTTCATCAATCTTAGGAGCTATTAATTTTATTACAACTATTATTAATATACGAATTAACCATATATCATTTGATCAAAT
ACCTCTCTTTGTATGAGCAGTAGGAATTACTGCTTTACTTTTATTATTATCTTTACCTGTATTAGCTGGTGCTATTACTATATTATTAACAGATCGA
AATCTTAATACTTCATTTTTTGATCCTGCAGGAGGTGGAGATCCTATTTTATATCAACATTTA

EU092971_Hu697_Bu_Hu --

TTTAATATTAGGAGCCCCAGATATAGCTTTTCCTCGAATAAATAATATAAGATTTTGATTACTACCCCCCTCATTAACTTTATTAATTTCTAGAAGA
ATTGTAGAAAATGGAGCAGGAACAGGATGAACAGTTTATCCCCCTTTATCATCTAATATCGCTCATAGAGGTAGTTCAGTTGATTTAGCTATTTTTT
CTTTACATTTAGCAGGAATTTCATCAATCTTAGGAGCTATTAATTTTATTACAACTATTATTAATATACGAATTAACCATATATCATTTGATCAAAT
ACCTCTCTTTGTATGAGCAGTAGGAATTACTGCTTTACTTTTATTATTATCTTTACCTGTATTAGCTGGTGCTATTACTATATTATTAACAGATCGA
AATCTTAATACTTCATTTTTTGATCCTGCAGGAGGTGGAGATCCTATTTTATATCAACATTTA

EU092969_Bu775 --

TTTAATATTAGGAGCCCCAGATATAGCTTTTCCTCGAATAAATAATATAAGATTTTGATTACTACCCCCCTCATTAACTTTATTAATTTCTAGAAGA
ATTGTAGAAAATGGAGCAGGAACAGGATGAACAATTTATCCCCCTTTATCATCTAATATCGCTCACAGAGGTAGTTCAGTTGATTTAGCTATTTTTT
CTTTACATTTAGCAGGAATTTCATCAATCTTAGGAGCTATTAATTTTATTACAACTATTATTAATATACGAATTAACCATATATCATTTGATCAAAT
ACCTCTCTTTGTATGAGCAGTAGGAATTACTGCTTTACTTTTATTATTATCTTTACCTGTATTAGCTGGTGCTATTACTATATTATTAACAGATCGA
AATCTTAATACTTCATTTTTTGATCCTGCAGGAGGTGGAGATCCTATTTTATATCAACATTTA

EU092968_Bu772 --

TTTAATATTAGGAGCCCCAGATATAGCTTTTCCTCGAATAAATAATATAAGATTTTGATTACTACCCCCCTCATTAACTTTATTAATTTCTAGAAGA
ATTGTAGAAAATGGAGCAGGAACAGGATGAACAGTTTATCCCCCTTTATCATCTAATATCGCTCATAGAGGTAGTTCAGTTGATTTAGCTATTTTTT
CTTTACATTTAGCAGGAATTTCATCAATCTTAGGAGCTATTAATTTTATTACAACTATTATTAATATACGAATTAACCATATATCATTTGATCAAAT
ACCTCTCTTTGTATGAGCAGTAGGAATTACTGCTTTACTTTTATTATTATCTCTACCTGTATTAGCTGGTGCTATTACTATATTATTAACAGATCGA
AATCTTAATACTTCATTTTTTGATCCTGCAGGAGGTGGAGATCCTATTTTATATCAACATTTA

EU093003_D982 --

TTTAATATTAGGAGCCCCAGATATAGCTTTTCCTCGAATAAATAATATAAGATTTTGATTACTACCCCCCTCATTAACTTTATTAATTTCTAGAAGA
ATTGTAGAAAATGGAGCAGGAACAGGATGAACAGTTTATCCCCCTTTATCATCTAATATTGCTCATAGAGGTAGTTCAGTTGATTTAGCTATTTTTT
CTTTACATTTAGCAGGAATTTCATCAATCTTAGGAGCTATTAATTTTATTACAACTATTATTAATATACGAATTAACCATATATCATTTGATCAAAT
ACCTCTCTTTGTATGGGCAGTAGGAATTACTGCTTTACTTTTATTATTATCTTTACCTGTATTAGCTGGTGCTATTACTATATTATTAACAGATCGA
AATCTTAATACTTCATTTTTTGATCCTGCAGGAGGTGGAGATCCTATTTTATATCAACATTA

DQ407769_UP_100_F
ACATTATATTTTATTTTTGGTATTTGAGCAGGTATAGTAGGAACCTCTTTAAGATTATTAATTCGTACTGAATTAGGTAATCCTGGATCTTTAATTG
GAGATGATCAAATTTATAACACTATTGTAACAGCTCATGCTTTTATTATAATTTTTTTTATAGTTATACCAATTATAATTGGAGGATTTGGAAATTG
ATTAATTCCTTTAATATTAGGAGCCCCAGATATAGCTTTCCCCCGAATAAATAATATAAGATTTTGATTACTACCCCCTTCATTAACCTTATTAATT
TCTAGAAGAATTGTAGAAAATGGAGCAGGAACTGGATGAACAGTTTACCCCCCTTTATCATCTAATATTGCTCATAGAGGAAGTTCAGTTGATTTAG
CTATTTTTTCCTTACATTTAGCAGGAATTTCATCAATCTTAGGAGCTATTAATTTTATTACAACTATTATTAATATACGAATTAATCATATATCATT
CGATCAAATACCTCTTTTTGTATGAGCAGTAGGAATTACTGCTTTACTCTTATTATTATCATTACCTGTATTAGCTGGTGCTATTACTATATTATTA
ACAGATCGAAATCTTAATACTTCATTTTTTGATCCAGCAGGGGGTGGTGA----------------------

AM231426_W336_parvisi_Gre
ACACTATATTTTATTTTTGGTATTTGAGCAGGTATAGTAGGAACCTCTTTAAGATTATTAATTCGTACTGAATTAGGTAATCCTGGATCTTTAATTG
GAGATGATCAAATTTATAATACTATTGTAACAGCTCATGCTTTTATTATAATTTTTTTTATAGTTATACCAATTATAATTGGAGGATTTGGAAATTG
ATTAATTCCTTTAATATTAGGAGCCCCAGATATAGCTTTTCCTCGAATAAATAATATAAGATTTTGATTACTACCCCCCTCATTAACTTTATTAATT
TCTAGAAGAATTG????????????????????GGATGAACAGTTTATCCCCCTTTATCATCTAATATCGCTCATAGAGGTAGTTCAGTTGATTTAG
CTATTTTTTCTTTACATTTAGCAGGAATTTCATCAATCTTAGGGGCTATTAATTTTATTACAACTATTATTAATATACGAATTAACCATATATCATT
TGATCAAATACCTCTCTTTGTATGAGCAGTAGGAATTACTGCTTTACTTTTATTATTATCTTTACCTGTATTAGCTGGTGCTATTACTATATTATTA
ACAGATCGAAATCTTAATACTTCATTTTTTGATCYTGCAGGAGGTGGAGATCCTATTTTATATCAA------

AM231425_W333_angorae_Tur
ACACTATATTTTATTTTTGGTATTTGAGCAGGTATAGTAGGAACCTCTTTAAGATTATTAATTCGTACTGAATTAGGTAATCCTGGATCTTTAATTG
GGGATGATCAAATTTATAATACTATTGTAACAGCTCATGCTTTTATTATAATTTTTTTTATAGTTATACCAATTATAATTGGAGGATTTGGAAATTG
ATTAATTCCTTTAATATTAGGAGCCCCAGATATAGCTTTTCCTCGAATAAATAATATAAGATTTTGATTATTACCCCCCTCATTAACTTTATTAATT
TCTAGAAGAATTG????????????????????GGATGAACAGTTTATCCCCCTTTATCATCTAATATTTCTCATAGAGGTAGTTCAGTTGATTTAG
CTATTTTTTCTTTACATTTAGCAGGAATTTCATCAATCTTAGGAGCTATTAATTTTATTACAACTATTATTAATATACGAATTAATCATATATCATT
TGATCAAATACCCCTTTTTGTATGGGCAGTAGGAATTACTGCTTTACTTTTATTATTATCTTTACCTGTATTAGCTGGTGCTWTTACTATATTATTA
ACAGATCGAAATCTTAATACTTCATTTTTCGATCCCGCAGGAGGTGGGGATCCTATTTTATATCAA------

AM231424_W331_angorae_Tur_Ankara
ACACTATATTTTATTTTTGGTATTTGAGCAGGTATAGTAGGAACCTCTTTAAGATTATTAATTCGTACTGAATTAGGTAATCCTGGATCTTTAATTG
GAGATGATCAAATTTATAATACTATTGTAACAGCTCATGCTTTTATTATAATTTTTTTTATAGTTATACCAATTATAATTGGAGGATTTGGAAATTG
ATTAATTCCTTTAATATTAGGAGCCCCAGATATAGCTTTTCCTCGAATAAATAATATAAGATTTTGATTATTACCCCCCTCATTAACTTTATTAATT
TCTAGAAGAATTGTA??????????????????GGATGAACAGTTTATCCCCCTTTATCATCTAATATCGCTCATAGAGGTAGCTCAGTTGATTTAG
CTATTTTTTCTTTACATTTAGCAGGAATTTCATCAATCTTAGGAGCTATTAATTTTATTACAACTATTATTAATATACGAATTAACCATATATCATT
TGATCAAATACCTCTCTTTGTATGAGCAGTAGGAATTACCGCTTTACTTTTATTATTATCTTTACCTGTATTAGCTGGTGCTATTACTATATTATTA
ACAGATCGAAATCTTAATACTTCATTTTTTGATCCTGCAGGAGGTGGAGATCCTATTTTATATCAA------

AM231423_W329_gigantea_Uzb_Chatkal
ACACTATATTTTATTTTTGGTATTTGAGCAGGTATAGTAGGAACCTCTTTAAGATTATTAATTCGTACTGAATTAGGTAATCCTGGATCTTTAATTG
GAGATGATCAAATTTATAATACTATTGTAACAGCTCATGCTTTTATTATAATTTTTTTTATAGTTATACCAATTATAATTGGAGGATTTGGAAATTG
ATTAATTCCTTTAATATTAGGAGCCCCAGATATAGCTTTTCCTCGAATAAATAATATAAGATTTTGATTATTACCCCCTTCATTAACTTTATTAATT
TCTAGAAGAATTG????????????????????GGATGAACAGTTTATCCCCCTTTATCATCTAATATCGCTCATAGAGGTAGTTCAGTTGATTTAG
CTATTTTTTCTTTACATTTAGCAGGAATTTCATCAATCTTAGGAGCTATTAATTTTATTACAACTATTATTAATATACGAATTAATCATATATCATT
TGATCAAATACCTCTCTTTGTATGAGCAGTAGGAATTACTGCTTTACTTTTATTACTATCTTTACCTGTATTAGCTGGTGCTATTACTATATTATTA
ACAGATCGAAATCTTAATACTTCATTTTTTGATCCTGCAGGAGGTGGAGATCCTATTTTATATCAA------

AM231422_W330_orientalis_Zailijskiy
ACACTATATTTTATTTTTGGTATTTGAGCAGGTATAGTAGGAACCTCTTTAAGATTATTAATTCGTACTGAATTAGGTAATCCTGGATCTTTAATTG
GAGATGATCAAATTTATAATACTATTGTAACAGCTCATGCTTTTATTATAATTTTTTTTATAGTTATACCAATTATAATTGGAGGATTTGGAAATTG
ATTAATTCCTTTAATATTAGGGGCCCCAGATATAGCTTTTCCTCGAATAAATAATATAAGATTTTGATTATTACCCCCTTCATTAACTTTATTAATT
TCTAGAAGAATTGTAGAAAATGGAGCAGGAACAGGATGAACAGTTTATCCCCCTTTATCATCTAATATCGCTCATAGAGGTAGTTCAGTTGATTTAG
CTATTTTTTCTTTACATTTAGCGGGAATTTCATCAATCTTAGGAGCTATTAATTTTATTACAACTATTATTAATATACGAATTAATCATATATCATT
TGATCAAATACCTCTCTTTGTATGAGCAGTAGGAATTACTGCTTTACTTTTATTACTATCTTTACCTGTATTAGCTGGTGCTATTACTATATTATTA
ACAGATCGAAATCTTAATACTTCATTTTTTGATCCTGCAGGAGGTGGAGATCCTATTTTATATCAA------

AM231421_W292_ochracea_Tadj_Zeravsh_
ACACTATATTTTATTTTTGGTATTTGAGCAGGTATAGTAGGAACCTCTTTAAGATTATTAATTCGTACTGAATTAGGTAATCCTGGATCTTTAATTG
GAGATGATCAAATTTATAATACTATTGTAACAGCTCATGCTTTTATTATAATTTTTTTCATAGTTATACCAATTATAATTGGAGGATTTGGAAATTG
ATTAATTCCTTTAATATTAGGAGCCCCAGATATAGCTTTTCCTCGAATAAATAATATAAGATTTTGATTACTACCCCCTTCATTAACTTTATTAATT
TCCAGAAGAATTGTAGAAAATGGAGCAGGAACAGGATGAACAGTTTATCCCCCTTTATCATCTAATATCGCTCATAGAGGTAGTTCAGTTGATTTAG
CTATTTTTTCTTTACATTTAGCAGGAATTTCATCAATTTTAGGAGCTATTAATTTCATTACAACTATTATTAATATACGAATTAACCATATATCATT
TGATCAAATACCCCTCTTTGTATGAGCAGTAGGAATTACTGCTTTACTTTTATTATTATCTTTACCTGTATTAGCTGGTGCTATTACTATATTATTA
ACAGATCGAAATCTTAATACTTCATTTTTTGATCCTGCAGG??GTGGAGATCCTATTTTATATCAA------

AM231420_W280_farsica_Iran_Fars
ACACTATATTTTATTTTCGGTATTTGAGCAGGTATAGTAGGAACCTCTTTAAGATTATTAATTCGTACTGAATTAGGTAATCCTGGATCTTTAATTG
GAGATGATCAAATTTATAATACTATTGTAACAGCTCATGCTTTTATTATAATCTTTTTTATAGTTATACCAATTATAATTGGAGGATTTGGAAATTG
ATTAATTCCTTTAATATTAGGAGCTCCAGATATAGCTTTCCCCCGAATAAATAATATAAGATTTTGATTACTCCCCCCCTCATTAACTTTATTAATT
TCTAGAAGAATTGTAGAAAATGGAGCAGGAACAGGATGAACAGTTTACCCCCCTTTATCATCTAATATTGCCCACAGAGGTAGTTCAGTTGATTTAG
CTATTTTTTCTTTACATTTAGCAGGAATTTCATCAATTTTAGGAGCTATTAATTTTATTACAACTATTATTAATATACGAATTAATTATATATCATT
TGATCAAATACCTCTTTTTGTATGAGCAGTAGGAATTACAGCATTACTTTTATTATTATCTTTACCTGTATTAGCTGGTGCTATTACTATATTATTA
ACAGATCGAAATCTTAACACTTCATTTTTTGATCCTGCA????GTGGGGATCCTATTTTATATCAA------

AM231419_W311_pseudonubilosus_Iran_Urmia
ACACTATATTTTATTTTCGGTATTTGAGCGGGTATAGTAGGAACCTCTTTAAGATTATTAATTCGTACTGAATTAGGTAATCCTGGATCTTTAATTG
GAGATGATCAAATTTATAACACTATTGTAACAGCTCATGCTTTTATTATAATTTTTTTTATAGTTATACCAATTATAATTGGAGGATTTGGAAATTG
ATTAATTCCTTTAATATTAGGAGCTCCAGATATAGCTTTTCCTCGAATAAATAATATAAGATTTTGATTACTCCCCCCCTCATTAACTTTATTAATT
TCTAGAAGAATTGTAGAAAATGGAGCAGGAACAGGATGAACAATTTATCCCCCTTTATCATCTAATATTGCTCATAGAGGTAGTTCAGTTGATTTAG
CTATTTTTTCTTTACATTTAGCAGGAATTTCATCAATCTTAGGAGCTATTAATTTTATTACAACTATTATTAACATACGAATTAATCATATATCATT
TGATCAAATACCTCTTTTTGTATGAGCAGTAGGAATTACTGCATTACTTTTATTATTATCTTTACCTGTATTAGCTGGTGCTATTACTATATTATTA
ACAGATCGAAATCTTAATACTTCATTTTTTGATCCTGCAGGA?GTGGAGATCCTATTTTATATCAA------

AM231418_W335_sheljuzhkoi_Tur_Adana
ACACTATATTTTATTTTTGGTATTTGAGCAGGTATAGTAGGAACCTCTTTAAGATTATTAATTCGTACTGAATTAGGTAATCCTGGATCTTTAATTA
GAGATGATCAAATTTATAATACTATTGTAACAGCTCATGCTTTTATTATAATTTTTTTTATAGTTATACCAATTATAATTGGAGGATTTGGAAATTG
ATTAATTCCTTTAATATTAGGAGCCCCAGATATAGCTTTTCCTCGAATAAATAATATAAGATTTTGATTACTTCCCCCTTCATTAACTTTATTAATT
TCTAGAAGAATTG????????????????????GGATGAACAGTTTATCCCCCTTTATCATCTAATATTGCTCATAGAGGTAGTTCAGTTGATTTAG
CTATTTTTTCTTTACATTTAGCAGGAATTTCATCAATCTTAGGAGCTATTAATTTTATTACAACTATTATTAATATACGAATTAATAATATATCATT
TGATCAAATACCTCTTTTTGTATGAGCAGTAGGAATTACTGCATTACTTTTATTATTATCCCTACCTGTATTAGCTGGTGCTATTACTATATTATTA
ACAGATCGAAATCTTAATACTTCATTTTTTGATCCCGCAGGAGGTGGAGATCCTATTTTATATCAA------

AM231417_W78_parmenides_Fr
ACATTATATTTTATTTTTGGTATTTGAGCAGGTATAGTAGGAACCTCTTTAAGATTATTAATTCGTACTGAATTAGGTAATCCTGGATCTTTAATTG
GAGATGATCAAATTTATAATACTATTGTAACAGCTCATGCTTTTATTATAATTTTTTTTATAGTTATACCAATTATAATTGGAGGATTTGGAAATTG
ATTAATTCCTTTAATATTAGGAGCCCCAGATATAGCTTTCCCCCGAATAAATAATATAAGATTTTGATTACTACCCCCTTCATTAACCTTATTAATT
TCTAGAAGAATTGTAGAAAATGGAGCAGGAACTGGATGAACAGTTTACCCCCCTTTATCATCTAATATTGCTCATAGAGGAAGTTCAGTTGATTTAG
CTATTTTTTCCTTACATTTAGCAGGAATTTCATCAATCTTAGGAGCTATTAATTTTATTACAACTATTATTAATATACGAATTAATCATATATCATT
CGATCAAATACCTCTTTTTGTATGAGCAGTAGGAATTACTGCTTTACTCTTATTATTATCATTACCTGTATTAGCTGGTGCTATTACTATATTATTA
ACAGATCGAAATCTTAATACTTCATTTTTTGATCCAGCAGGGGGTGGTGATCCTATTTTATATCAA------

EU836682_h_1_32_Rus --

GCTTTTCCTCGAATAAATAATATAAGATTTTGATTACTACCCCCCTCATTAACTTTATTAATTTCTAGAAGAATTGTAGAAAATGGAGCAGGAACAG
GATGAACAGTTTATCCCCCTTTATCATCTAATATCGCTCATAGAGGTAGTTCAGTTGATTTAGCTATTTTTTCTTTACATTTAGCAGGAATTTCATC
AATCTTAGGAGCTATTAATTTTATTACAACTATTATTAATATACGAATTAACCATATATCATTTGATCAAATACCTCTCTTTGTATGAGCAGTAGGA
ATTACTGCTTTACTTTTATTATTATCTTTACCTGTATTAGCTGGTGCTATTACTATATTATTAACAGATCGAAATCTTAATACTTCATTTTTTGATC
CTGCAGGAGGTGGGGATCCTATTTTATATCAACATTTA

EU836681_h_1_31_Rus --

GCTTTTCCTCGAATAAATAATATAAGATTTTGATTACTACCCCCCTCATTAACTTTATTAATTTCTAGAAGAATTGTAGAAAATGGAGCAGGAACAG
GATGAACAATTTATCCCCCTTTATCATCTAATATCGCTCATAGAGGTAGTTCAGTTGATTTAGCTATTTTTTCTTTACATTTAGCAGGAATTTCATC
AATCTTAGGAGCTATTAATTTTATTACAACTATTATTAATATACGAATTAACCATATATCATTTGATCAAATACCTCTCTTTGTATGAGCAGTAGGA
ATTACTGCTTTACTTTTATTATTATCTTTACCTGTATTAGCTGGTGCTATTACTATATTATTAACAGATCGAAATCTTAATACTTCATTTTTTGATC
CTGCAGGAGGTGGGGATCCTATTTTATATCAACATTTA

EU836680_A_Bo_Bu_Cr_Cz_Hu_Sl_Li_Fi_Po_Ru --

GCTTTTCCTCGAATAAATAATATAAGATTTTGATTACTACCCCCCTCATTAACTTTATTAATTTCTAGAAGAATTGTAGAAAATGGAGCAGGAACAG
GATGAACAGTTTATCCCCCTTTATCATCTAATATCGCTCATAGAGGTAGTTCAGTTGATTTAGCTATTTTTTCTTTACATTTAGCAGGAATTTCATC
AATCTTAGGAGCTATTAATTTTATTACAACTATTATTAATATACGAATTAACCATATATCATTTGATCAAATACCTCTCTTTGTATGAGCAGTAGGA
ATTACTGCTTTACTTTTATTATTATCTTTACCTGTATTAGCTGGTGCTATTACTATATTATTAACAGATCGAAATCTTAATACTTCATTTTTTGATC
CTGCAGGAGGTGGAGATCCTATTTTATATCAACATTTA

EU836675_h_1_19_It --

GCTTTCCCTCGAATAAATAATATAAGATTTTGATTACTACCCCCCTCATTAACTTTATTAATTTCTAGAAGAATTGTAGAAAATGGAGCAGGAACAG
GATGAACAGTTTATCCCCCTTTATCATCTAATATTGCTCATAGAGGTAGTTCAGTTGATTTAGCTATTTTTTCTTTACATTTAGCAGGAATTTCATC
AATCTTAGGAGCTATTAATTTTATTACAACTATTATTAATATACGAATTAACCATATATCATTTGATCAAATACCTCTCTTTGTATGAGCAGTAGGA
ATTACTGCTTTACTTTTATTATTATCTTTACCTGTATTAGCTGGTGCTATTACTATATTATTAACAGATCGAAATCTTAATACTTCATTTTTTGATC
CTGCAGGAGGTGGAGATCCTATTTTATATCAACATTTA

EU836674_h_1_18_At_It --

GCTTTTCCTCGAATAAATAATATAAGATTTTGATTACTACCCCCCTCATTAACTTTATTAATTTCTAGAAGAATTGTAGAAAATGGAGCAGGAACAG
GATGAACAGTTTATCCCCCTTTATCATCTAATATTGCTCATAGAGGTAGTTCAGTTGATTTAGCTATTTTTTCTTTACATTTAGCAGGAATTTCATC
AATCTTAGGAGCTATTAATTTTATTACAACTATTATTAATATACGAATTAACCATATATCATTTGATCAAATACCTCTCTTTGTATGAGCAGTAGGA
ATTACTGCTTTACTTTTATTATTATCTTTACCTGTATTAGCTGGTGCTATTACTATATTATTAACAGATCGAAATCTTAATACTTCATTTTTTGATC
CTGCAGGAGGTGGAGATCCTATTTTATATCAACATTTA

EU836672_h_1_10_It --

GCTTTCCCTCGAATAAATAATATAAGATTTTGATTACTACCCCCCTCATTAACTTTATTAATTTCTAGAAGAATTGTAGAAAATGGGGCAGGAACAG
GATGAACAGTTTATCCCCCTTTATCATCTAATATCGCTCATAGAGGTAGTTCAGTTGATTTAGCTATTTTTTCTTTACATTTAGCAGGAATTTCATC
AATCTTAGGAGCTATTAATTTTATTACAACTATTATTAATATACGAATTAACCATATATCATTTGATCAAATACCTCTCTTTGTATGAGCAGTAGGA

ATTACTGCTTTACTTTTATTATTATCTTTACCTGTATTAGCTGGTGCTATTACTATATTATTAACAGATCGAAATCTTAATACTTCATTTTTTGATC
CTGCAGGAGGTGGAGATCCTATTTTATATCAACATTTA

EU836671_h_1_09_It --

GCTTTTCCTCGAATAAATAATATAAGATTTTGATTACTACCCCCCTCATTAACTTTATTAATTTCTAGAAGAATTGTAGAAAATGGGGCAGGAACAG
GATGAACAGTTTATCCCCCTTTATCATCTAATATCGCTCATAGAGGTAGTTCAGTTGATTTAGCTATTTTTTCTTTACATTTAGCAGGAATTTCATC
AATCTTAGGAGCTATTAATTTTATTACAACTATTATTAATATACGAATTAACCATATATCATTTGATCAAATACCTCTCTTTGTATGAGCAGTAGGA
ATTACTGCTTTACTTTTATTATTATCTTTACCTGTATTAGCTGGTGCTATTACTATATTATTAACAGATCGAAATCTTAATACTTCATTTTTTGATC
CTGCAGGAGGTGGAGATCCTATTTTATATCAACATTTA

EU836670_h_1_07_Gre --

GCTTTTCCTCGAATAAATAATATAAGATTTTGATTACTACCCCCCTCATTAACTTTATTAATTTCTAGAAGAATTGTAGAAAATGGAGCAGGAACAG
GATGAACAATTTATCCCCCTTTATCATCTAATATCGCTCATAGAGGTAGTTCAGTTGATTTAGCTATTTTTTCTTTACATTTAGCAGGAATTTCATC
AATCTTAGGGGCTATTAATTTTATTACAACTATTATTAATATACGAATTAACCATATATCATTTGATCAAATACCTCTCTTTGTATGAGCAGTAGGA
ATTACTGCTTTACTTTTATTATTATCTTTACCTGTATTAGCTGGTGCTATTACTATATTATTAACAGATCGAAATCTTAATACTTCATTTTTTGATC
CTGCAGGAGGTGGAGATCCTATTTTATATCAACATTTA

EU836669_h_1_06_Gre --

GCTTTCCCTCGAATAAATAATATAAGATTTTGATTACTACCCCCCTCATTAACTTTATTAATTTCTAGAAGAATTGTAGAAAATGGAGCAGGAACAG
GATGAACAGTTTATCCCCCTTTATCATCTAATATAGCTCATAGAGGTAGTTCAGTTGATTTAGCTATTTTTTCTTTACATTTAGCAGGAATTTCATC
AATCTTAGGAGCTATTAATTTTATTACAACTATTATTAATATACGAATTAACCATATATCATTTGATCAAATACCTCTCTTTGTATGAGCAGTAGGA
ATTACTGCTTTACTTTTATTATTATCTTTACCTGTATTAGCTGGTGCTATTACTATATTATTAACAGATCGAAATCTTAATACTTCATTTTTTGATC
CTGCAGGAGGTGGAGATCCTATTTTATATCAACATTTA

EU836668_h_1_05_Gre --

GCTTTCCCTCGAATAAATAATATAAGATTTTGATTACTACCCCCCTCATTAACTTTATTAATTTCTAGAAGAATTGTAGAAAATGGAGCAGGAACAG
GATGAACAGTTTATCCCCCTTTATCATCTAATATCGCTCATAGAGGTAGTTCAGTTGATTTAGCTATTTTTTCTTTACATTTAGCAGGAATTTCATC
AATCTTAGGGGCTATTAATTTTATTACAACTATTATTAATATACGAATTAACCATATATCATTTGATCAAATACCTCTCTTTGTATGAGCAGTAGGA
ATTACTGCTTTACTTTTATTATTATCTTTACCTGTATTAGCTGGTGCTATTACTATATTATTAACAGATCGAAATCTTAATACTTCATTTTTTGATC
CTGCAGGAGGTGGAGATCCTATTTTATATCAACATTTA

EU836667_h_1_04_Gre --

GCTTTCCCTCGAATAAATAATATAAGATTTTGATTACTACCCCCCTCATTAACTTTATTAATTTCTAGAAGAATTGTAGAAAATGGAGCAGGAACAG
GATGAACAGTTTATCCCCCTTTATCATCTAATATCGCTCATAGAGGTAGTTCAGTTGATTTAGCTATTTTTTCTTTACATTTAGCAGGAATTTCATC
AATCTTAGGGGCTATTAATTTTATTACAACTATTATTAATATACGAATTAACCATATATCATTTGATCAAATACCTCTCTTTGTATGAGCAGTAGGA
ATTACTGCTTTACTTTTATTATTATCTTTACCCGTATTAGCTGGTGCTATTACTATATTATTAACAGATCGAAATCTTAATACTTCATTTTTTGATC
CTGCAGGAGGTGGAGATCCTATTTTATATCAACATTTA

EU836666_h_1_03_Mac --

GCTTTTCCTCGAATAAATAATATAAGATTTTGATTACTACCCCCCTCATTAACTTTATTAATTTCTAGAAGAATTGTAGAAAATGGAGCAGGAACAG
GATGAACAGTTTATCCCCCTTTATCATCTAATATCGCTCATAGAGGTAGTTCAGTTGATTTAGCTATTTTTTCTTTACATTTAGCGGGAATTTCATC
AATCTTAGGGGCTATTAATTTTATTACAACTATTATTAATATACGAATTAACCATATATCATTTGATCAAATACCTCTCTTTGTATGAGCAGTAGGA
ATTACTGCTTTACTTTTATTATTATCTTTACCTGTATTAGCTGGTGCTATTACTATATTATTAACAGATCGAAATCTTAATACTTCATTTTTTGATC
CTGCAGGAGGTGGAGATCCTATTTTATATCAACATTTA

EU836665_h_1_02_Gre_Mac --

GCTTTTCCTCGAATAAATAATATAAGATTTTGATTACTACCCCCCTCATTAACTTTATTAATTTCTAGAAGAATTGTAGAAAATGGAGCAGGAACAG
GATGAACAGTTTATCCCCCTTTATCATCTAATATCGCTCATAGAGGTAGTTCAGTTGATTTAGCTATTTTTTCTTTACATTTAGCAGGAATTTCATC
AATCTTAGGGGCTATTAATTTTATTACAACTATTATTAATATACGAATTAACCATATATCATTTGATCAAATACCTCTCTTTGTATGAGCAGTAGGA
ATTACTGCTTTACTTTTATTATTATCTTTACCTGTATTAGCTGGTGCTATTACTATATTATTAACAGATCGAAATCTTAATACTTCATTTTTTGATC
CTGCAGGAGGTGGAGATCCTATTTTATATCAACATTTA

EU836664_h_2_06_At_It --

GCTTTTCCTCGAATAAATAATATAAGATTTTGATTGCTACCCCCCTCATTAACTTTATTAATTTCTAGAAGAATTGTAGAAAATGGAGCAGGAACAG
GATGAACAGTTTATCCCCCTTTATCATCTAATATCGCTCATAGAGGTAGTTCAGTTGATTTAGCTATTTTTTCTTTACATTTAGCAGGAATTTCATC
AATCTTAGGAGCTATTAATTTTATTACAACTATTATTAATATACGAATTAACCATATATCATTTGATCAAATACCTCTCTTTGTATGAGCAGTAGGA
ATTACTGCTTTACTTTTATTATTATCTTTACCTGTATTAGCTGGTGCTATTACTATATTATTAACAGATCGAAATCTTAATACTTCATTTTTTGATC
CTGCAGGAGGTGGAGATCCTATTTTATATCAACATTTA

EU836663_h_2_05_It --

GCTTTCCCTCGAATAAATAATATAAGATTTTGATTGCTACCCCCCTCATTAACTTTATTAATTTCTAGAAGAATTGTAGAAAATGGAGCAGGAACAG
GATGAACAGTTTATCCCCCTTTATCATCTAATATCGCTCATAGAGGTAGTTCAGTTGATTTAGCTATTTTTTCTTTACATTTAGCAGGAATTTCATC

```
AATCTTAGGAGCTATTAATTTTATTACAACTATTATTAATATACGAATTAACCATATATCATTTGATCAAATACCTCTCTTTGTATGAGCAGTAGGA
ATTACTGCTTTACTTTTATTATTATCTTTACCTGTATTAGCTGGTGCTATTACTATATTATTAACAGATCGAAATCTTAATACTTCATTTTTTGATC
CTGCAGGAGGTGGAGATCCTATTTTATATCAACATTTA;


END;
begin mrbayes;
	set autoclose=yes nowarn=yes;
	partition ancstates = 2: 1, 2-655;
	set partition=ancstates;
	lset applyto=(2) rates=invgamma nst=6;
	unlink statefreq=(all) revmat=(all) shape=(all) pinvar=(all);
	prset ratepr=variable;
	constraint stubbendorfii_clade= Parnassius_stubbendorfii_2005_LOWA_815
Parnassius_stubbendorfii_2005_LOWA_154 Parnassius_stubbendorfii_2005_LOWA_153
Parnassius_glacialis_mikado Parnassius_stubbendorfii_hoenei
Parnassius_stubbendorfii_hoenei_AC20_16 Parnassius_stubbendorfii_koreanus;
	prset topologypr=constraints(stubbendorfii_clade);
	report applyto=(1) ancstates=yes;
	mcmcp ngen= 100000 relburnin=yes burninfrac=0.25 printfreq=100  samplefreq=100 nchains=4
savebrlens=yes;
	mcmc;
	sump;
end;
```
